# Lysosomal regulation of cholesterol homeostasis in tuberous sclerosis complex is mediated *via* NPC1 and LDL-R

**DOI:** 10.18632/oncotarget.17485

**Published:** 2017-04-27

**Authors:** Harilaos Filippakis, Nicola Alesi, Barbara Ogorek, Julie Nijmeh, Damir Khabibullin, Catherine Gutierrez, Alexander J. Valvezan, James Cunningham, Carmen Priolo, Elizabeth P. Henske

**Affiliations:** ^1^ Division of Pulmonary and Critical Care Medicine, Brigham and Women's Hospital, Harvard Medical School, Boston, Massachusetts, USA; ^2^ Department of Genetics and Complex Diseases, Harvard School of Public Health, Boston, Massachusetts, USA; ^3^ Department of Medicine, Division of Hematology, Brigham and Women's Hospital, Harvard Medical School, Boston, Massachusetts, USA

**Keywords:** TSC, mTORC1, chloroquine, cholesterol, lysosome

## Abstract

Tuberous sclerosis complex (TSC) is a multisystem disease associated with hyperactive mTORC1. The impact of TSC1/2 deficiency on lysosome-mediated processes is not fully understood. We report here that inhibition of lysosomal function using chloroquine (CQ) upregulates cholesterol homeostasis genes in TSC2-deficient cells. This TSC2-dependent transcriptional signature is associated with increased accumulation and intracellular levels of both total cholesterol and cholesterol esters. Unexpectedly, engaging this CQ-induced cholesterol uptake pathway together with inhibition of *de novo* cholesterol synthesis allows survival of TSC2-deficient, but not TSC2-expressing cells. The underlying mechanism of TSC2-deficient cell survival is dependent on exogenous cholesterol uptake via LDL-R, and endosomal trafficking mediated by Vps34. Simultaneous inhibition of lysosomal and endosomal trafficking inhibits uptake of esterified cholesterol and cell growth in TSC2-deficient, but not TSC2-expressing cells, highlighting the TSC-dependent lysosome-mediated regulation of cholesterol homeostasis and pointing toward the translational potential of these pathways for the therapy of TSC.

## INTRODUCTION

Tuberous Sclerosis Complex (TSC) is a multisystem disease caused by mutations in either the *TSC1* or *TSC2* gene [1]. TSC is a phenotypically and pathologically diverse disease with clinical manifestations that impact the central nervous system (seizures, autism, cognitive impairment) as well as hamartomatous tumors that involve multiple organ systems (brain, skin, kidney, heart and lung) and gender-specific phenotypes (lymphangioleiomyomatosis) [2]. The TSC proteins function as part of a multicomponent complex to regulate the mechanistic target of rapamycin complex 1 (mTORC1) by directly inhibiting the small GTPase, Rheb [3]. Loss of TSC1/2 leads to constitutive mTORC1 activation, resulting in increased protein translation and a profound effect on cellular metabolism *via* several inter-connected mechanisms, including glucose and glutamine utilization, nucleotide synthesis, and lipid synthesis [4–6]. mTORC1 activates the sterol regulatory element-binding proteins (SREBP1 and SREBP2), which in turn enhance expression of genes regulating *de novo* synthesis of fatty acids and sterols [7, 8]. In addition to stimulating biosynthetic processes, mTORC1 inhibits the catabolic process of autophagy *via* phosphorylation of ULK1 and lysosomal biogenesis through phosphorylation of the TFEB transcription factor [9–11].

The lysosome plays a critical role in sustaining cellular homeostasis. Lysosomes are characterized by a highly acidic lumen containing acid hydrolases. Although they were initially described as inert organelles, recent discoveries have revealed that lysosomal biogenesis and function are subject to tight transcriptional regulation [12]. Lysosomes are involved in degrading extracellular material delivered through the endocytic pathway, as well as intracellular components and organelles transported to the lysosomes *via* autophagy [13–15]. Chloroquine (CQ) is a lysosomal/autophagy inhibitor that exerts its effects through its lysosomotropic function. Chloroquine crosses the lysosomal membrane and becomes protonated resulting in its accumulation within the organelle. Continuous sequestration of CQ in the lysosome increases the pH of the organelle, thereby inactivating lysosomal enzymes [16]. CQ has been shown to suppress the growth of TSC2-deficient cells both *in vitro* and *in vivo*, and to induce the pentose phosphate pathway in TSC2-deficient cells, enabling selective therapeutic targeting of TSC2-deficient cells using the combination of CQ and pentose phosphate pathways inhibitors [17, 18].

Lysosomal degradation of exogenously-supplied nutrients involves their transport *via* Vps34 (vacuolar protein sorting 34)-regulated early and late endosomes. Vps34 (PI3KC3) is a lipid kinase with dual functions in autophagy initiation and vesicular trafficking. The kinase activity of Vps34 is crucial for the formation of endocytic membranes by phosphorylating the 3`-hydroxy position of the phosphatidylinositol ring to generate phosphatidylinositol 3-phosphate (PI(3)P) [19, 20].

Here we report that lysosomal inhibition of TSC2-deficient cells enhances the uptake and processing of cholesterol. Remarkably, this allows TSC2-deficient cells but not TSC2-expressing cells to survive in the presence of an HMGCR inhibitor, simvastatin. Importantly, CQ-dependent regulation of cholesterol homeostasis sensitizes TSC2-deficient cells to endosomal trafficking inhibition by concomitant inhibition of lysosomal function and Vps34. These results reveal a novel consequence of mTORC1-dependent metabolic reprogramming that impacts cholesterol homeostasis and has therapeutic implications for the elimination of TSC2-deficient tumor cells.

## RESULTS

### Chloroquine induces a transcriptional metabolic signature in mTORC1-hyperactive cells

**Figure 1:**
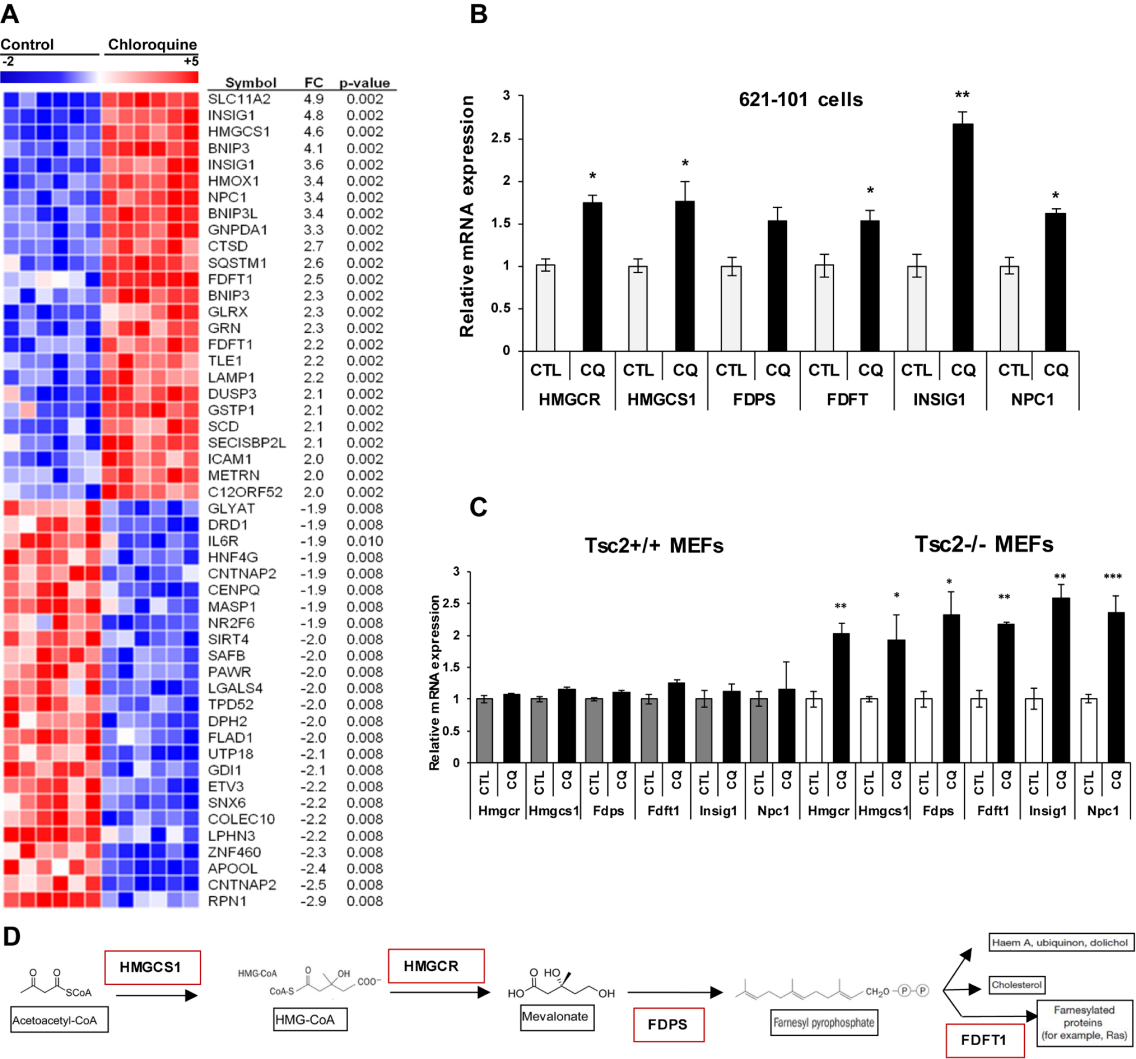
Chloroquine treatment upregulates mevalonate pathway genes in TSC2-deficient cells **A**. Transcriptional profiling (978 genes) of human kidney angiomyolipoma-derived cells (621-101) treated with CQ (5uM) for 24 hours (six biological replicates) using L-1000 platform. Supervised clustering of the top 20 up and downregulated genes compared to untreated 621-101 cells. **B**. Validation of upregulated mevalonate pathway genes by qRT-PCR after 24 hours CQ treatment of 621-101 cells. **C**. Expression levels of mevalonate pathway genes in Tsc2^+/+^ and Tsc2^−/−^ mouse embryonic fibroblasts (MEFs). Bar graph represents means ±SD of values relative to their vehicle control (H_2_0). One sample *t*-test was used to compare mRNA expression levels between treated and untreated conditions; *p < 0.05, **p < 0.01. See also Supplementary Table 1. **D**. Concise schematic of the mevalonate pathway with key genes highlighted in red.

To determine the effects of lysosomal inhibition on the transcriptome of mTORC1-hyperactive cells, we treated TSC2-deficient human kidney-derived angiomyolipoma cells (621-101), TSC2-empty vector angiomyolipoma cells (621-102), and TSC2-add back angiomyolipoma cells (621-103) with 5μM of CQ for 24 hours. CQ, a lysosomotropic agent inhibits lysosomal function by accumulating in the lysosome and increasing the pH of this highly acidic organelle. L1000 gene expression profiling was carried out to assess gene expression changes. L1000 is a high-throughput genomics platform that uses a bead-based Luminex method to measure 978 landmark genes [21–23]. The chloroquine-induced “landmark” gene expression signature showed upregulation of mevalonate pathway genes in both 621-101 and empty vector-expressing 621-102 cells but not in TSC2-add back 621-103 cells (Figure 1A and Supplementary Table 1). More specifically, multiple genes involved in cholesterol synthesis, uptake and processing were significantly induced upon CQ treatment, including Insulin-induced gene 1 (*INSIG1*; 4.8-fold), 3-Hydroxy-3-Methylglutaryl-CoA Synthase 1 (*HMGCS1*; 4.6-fold), Farnesyl Diphosphate Transferase 1 (*FDFT1*; 2.5-fold), Niemann Pick Disease Type 1 (*NPC1*; 3.4-fold) and 3-Hydroxy-3-Methylglutaryl-CoA Reductase (*HMGCR*; 1.7-fold), (Figure 1A and 1D). Quantitative real-time PCR carried out on cells treated with CQ confirmed the upregulated genes, both in 621-101 cells and in Tsc2^−/−^ mouse embryonic fibroblasts (MEFs) (Figure 1B and 1C respectively).

### Chloroquine induces accumulation of cholesterol esters in the lysosomes of TSC2-deficient cells

**Figure 2:**
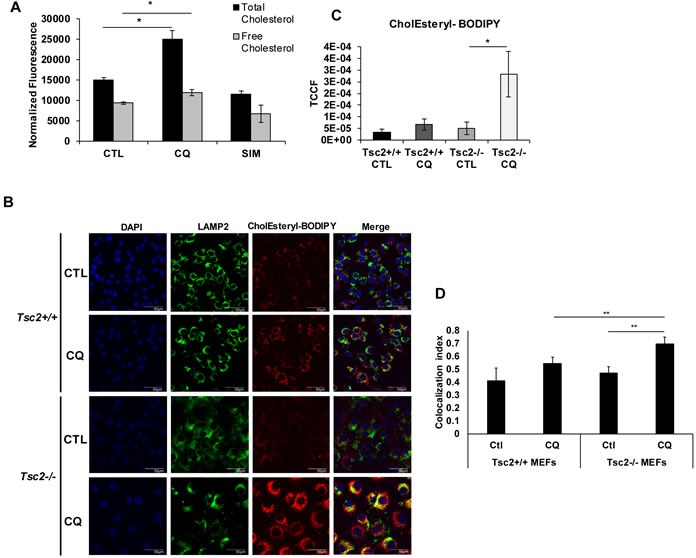
Chloroquine treatment increases esterified cholesterol levels and lysosomal accumulation in Tsc2^−/−^ cells **A**. Chloroquine increases total cholesterol levels in TSC2-deficient cells. Total and free cholesterol levels measured in TSC2-deficient cells treated with CQ (5uM), simvastatin (10uM) for 16 hrs. Values were normalized to total protein. Student's *t*-test was used. Bar graph represents means ±SD of values. **B**. Exogenously supplied CholEsteryl-BODIPY (1ug/ml; 4 hours) accumulates in distinctive puncta in Tsc2^−/−^ cells pretreated with CQ (5uM) for 16 hours. The puncta colocalize with the lysosomal membrane marker LAMP2A in CQ-pretreated Tsc2^−/−^ cells but not in Tsc2^+/+^ cells. **C**. Quantification of CholEsteryl-BODIPY in vehicle treated (H2O) and CQ-treated cells depicted in panel B (TCCF; total corrected cell fluorescence). Image acquisition was carried out using identical exposure settings. Student's *t*-test was carried out. **D**. Colocalization index of CholEsteryl-BODIPY and *Lamp2A* in vehicle treated (H2O) and CQ-treated cells depicted in panel B. The colocalization signal for each condition was measured from four representative fields, n≥15 cells. The colocalization index represents the Pearson's coefficient (zero is no colocalization and one means perfect colocalization).

To understand the metabolic consequences of CQ on genes involved in cholesterol homeostasis, we measured free and total cholesterol in Tsc2^−/−^ MEFs treated for 16 hours with vehicle control (DMSO), CQ (5 uM) or simvastatin (10 uM). Simvastatin is an inhibitor of HMGCR, the rate-limiting enzyme in the metabolic pathway responsible for endogenous cholesterol production. CQ increased total cholesterol by 66% (*p* < 0.05), while free cholesterol was slightly increased (Figure 2A), suggesting that CQ may increase cholesterol esters uptake and accumulation in the lysosomes. Simvastatin decreased the levels of total and free cholesterol in Tsc2^−/−^ MEFs as expected. Exogenous esterified cholesterol is internalized by LDLR and is processed at the late endosomes and lysosomes. Clearance of free cholesterol from the lysosome requires the interaction of lipoproteins with NPC1/2 proteins, which facilitate transport of free cholesterol to the cytoplasm.

Next, to investigate how cholesterol uptake and trafficking are affected by lysosomal inhibition, we treated Tsc2-deficient MEFs with CQ (5μM) or vehicle control (H_2_O) for 16 hours and performed fluorescent esterified cholesterol uptake assays. We used CholEsteryl-BODIPY (1ug/ml), a fluorescent cholesterol analogue labeled at the fatty acid ester and internalized by LDLR, to treat cells and incubated for 4 hours. At baseline, CholEsteryl-BODIPY supplementation of Tsc2^+/+^ MEFs showed subtle accumulation in cells treated with CQ when compared to untreated cells (Figure 2B, upper eight panels). In contrast, exogenously supplied CholEsteryl-BODIPY strikingly accumulated in distinct punctate vesicles in Tsc2-deficient cells treated with CQ (Figure 2B, lower eight panels). Exogenously supplied esterified cholesterol levels were significantly increased in CQ-treated Tsc2^−/−^ MEFs compared to untreated and Tsc2^+/+^ MEFs as quantified in Figure 2C. The CholEsteryl-BODIPY puncta observed after CQ treatment strongly colocalized with Lamp2A only in Tsc2^−/−^ MEFs suggesting that exogenous cholesterol esters accumulate in lysosomes, potentially as a consequence of CQ-induced lysosomal dysfunction (colocalization index 0.69 in CQ-treated *vs* 0.49 untreated Tsc2^−/−^ MEFs), (Figure 2B and 2D). CholEsteryl-BODIPY colocalization with Lamp2A in Tsc2^+/+^ MEFs was only slightly increased following CQ treatment (colocalization index 0.54 in CQ-treated *vs* 0.41 in untreated Tsc2^+/+^ MEFs), (Figure 2D).

### Chloroquine treatment protects Tsc2-deficient cells from Simvastatin induced death

**Figure 3:**
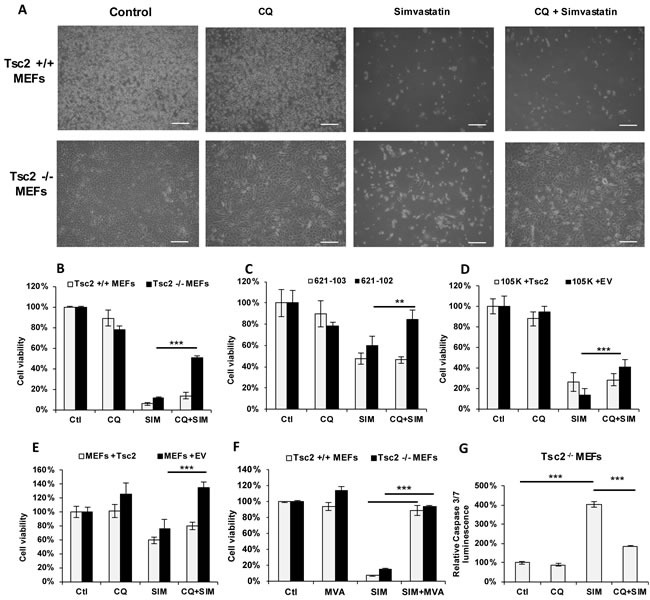
Chloroquine rescues Tsc2^−/−^ cells from simvastatin-induced cell death **A**. Phase contrast microscopy images of Tsc2^+/+^ and Tsc2^−/−^ MEFs treated with vehicle control (DMSO), CQ (5uM), Simvastatin (10uM) or the combination for 72 hrs (scale bars 100um). Cell viability (as measured by crystal violet staining) of **B**. Tsc2^+/+^ and Tsc2^−/−^ MEFs, **C**. human kidney-derived angiomyolipoma with TSC-2 add-back cells (621-103) or with empty vector (621-102) cells, **D**. mouse kidney cystadenoma 105K cells with Tsc2 expressing or empty vector (EV), and **E**. Tsc2^−/−^ MEFs with Tsc2 re-expressing cells or with EV, treated with: vehicle control (DMSO), CQ (5uM), simvastatin (10uM) or the combination for 72 hrs. Results represent average of three independent experiments ±SD of values. Two-sample *t*-test was carried out between simvastatin and CQ combination treatment and simvastatin treatment alone. **F**. Cell viability of Tsc2^+/+^ and Tsc2^−/−^ MEFs treated with vehicle control (DMSO), simvastatin (10uM), Mevalonic acid (MVA) (500uM) or the combination for 72 hrs, as measured by crystal violet staining. **G**. Caspase 3 and caspase 7 levels in Tsc2^−/−^MEFs treated with CQ, simvastatin or the combination for 24 hrs. Results are representative of three independent experiments. Two-sample *t*-test was carried out between simvastatin and vehicle control and between simvastatin alone and combination of CQ and simvastatin (*n* = 3). **p < 0.01, ***p < 0.001. See also Supplementary Figure S1.

Our earlier observations showed that both total and esterified cholesterol were increased by CQ, and that cholesterol esters accumulate in the lysosomes of Tsc2^−/−^ MEFs treated with CQ. To determine the role of newly synthesized cholesterol for their survival upon lysosomal inhibition, we monitored the proliferation of Tsc2^−/−^ and Tsc2^+/+^ MEFs after treatment with CQ (5 uM), simvastatin (10 uM, to inhibit *de novo* cholesterol synthesis), or combination of the two drugs for 3 days. CQ treatment as a single agent had minimal effects on the proliferation of Tsc2^−/−^ and Tsc2^+/+^ MEFs, while simvastatin significantly reduced viability of both cell lines at 72 hours. Surprisingly, CQ rescued the viability of Tsc2^−/−^ MEFs by ~3.5 fold (*p* < 0.001) compared to simvastatin treatment alone, but had no significant effect on the viability of Tsc2^+/+^ MEFs (Figure 3A-3B). To confirm that this effect is TSC2-specific, we tested three other pairs of TSC2-expressing and TSC2-deficient cell lines (Figure 3C-3E). In all cases the survival of simvastatin-treated cells was rescued upon combination treatment with CQ in the TSC2-deficient, but not in the TSC2-expressing cells. To further confirm the specificity of the mevalonate pathway in the survival of cells treated with simvastatin and CQ, we measured the proliferation of Tsc2^−/−^ MEFs treated with simvastatin (10uM) and mevalonic acid (500uM) for 72 hours. Both Tsc2^+/+^ and Tsc2^−/−^ MEFs were rescued from simvastatin-induced cell death by mevalonic acid, as expected (Figure 3F).

To determine the cellular consequences of the combination of CQ and simvastatin, we monitored apoptosis by using a fluorescent assay for activation of caspase 3 and 7. Treatment of Tsc2-deficient MEFs with simvastatin increased caspase 3/7 activity, as expected, while co-treatment with CQ decreased caspase activity by ~ 50% (Figure 3G), consistent with the results observed in the cell viability experiments. These results point towards an mTORC1-dependent mechanism that allows TSC2-deficient cells to survive when both sources of cholesterol (exogenous and *de novo*) are inhibited.

### Expression of genes involved in regulation of cholesterol homeostasis is increased with the combination of CQ and simvastatin treatment in TSC2-null but not TSC2-expressing cells

**Figure 4:**
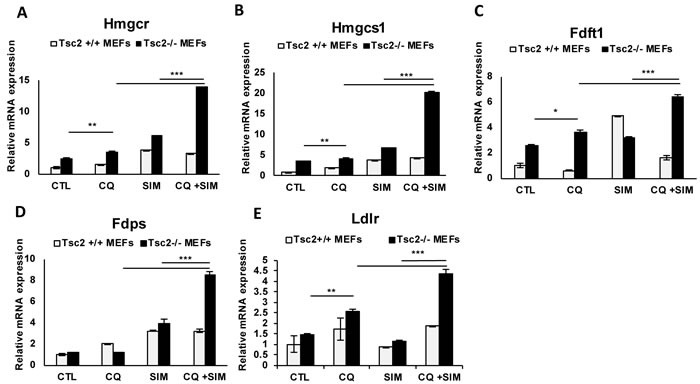
Chloroquine and simvastatin combination treatment upregulates cholesterol homeostasis genes in TSC-2 deficient cells **A**.-**E**. mRNA expression levels of *Hmgcr, Hmgcs1, Fdft1, Fdps* and *Ldlr* genes following treatment with CQ, simvastatin or combination for 24 hours. Bar graph represents means ±SD of values relative to Tsc2 ^+/+^ vehicle control (H_2_0). One sample *t*-test was used to compare mRNA expression levels between CQ and simvastatin combination and each treatment alone and between CQ-treatment and vehicle control. Bar graph represents means ±SD of values. * *p* < 0.05, **p < 0.01. ***p < 0.001.

To determine how the mevalonate pathway and the lipoprotein-specific receptor LDLR are affected by the combination of CQ and simvastatin, we treated Tsc2^+/+^ and Tsc2^−/−^ MEFs with CQ (5uM), simvastatin (10uM) or combination for 24 hours and quantified gene expression using qRT-PCR. Treatment with CQ increased the mRNA expression levels of *Hmgcr* and *Hmgcs1* as expected based on our prior results (Figure 1C), as well as *Fdft1* and *Ldlr* in Tsc2^−/−^ MEFs but not in Tsc2^+/+^ MEFs. Interestingly, combination treatment with CQ and simvastatin further increased expression of *Hmgcr, Hmgcs1*, *Fdft1, Fdps and Ldlr*, when compared to each treatment alone (Figure 4A-4E). These results indicate that Tsc2^−/−^ MEFs possess a higher capacity for enhancement of cholesterol homeostasis, including both *do novo* and exogenous cholesterol uptake upon lysosomal inhibition.

### SREBP2 mediates the pro-survival effects of CQ

**Figure 5:**
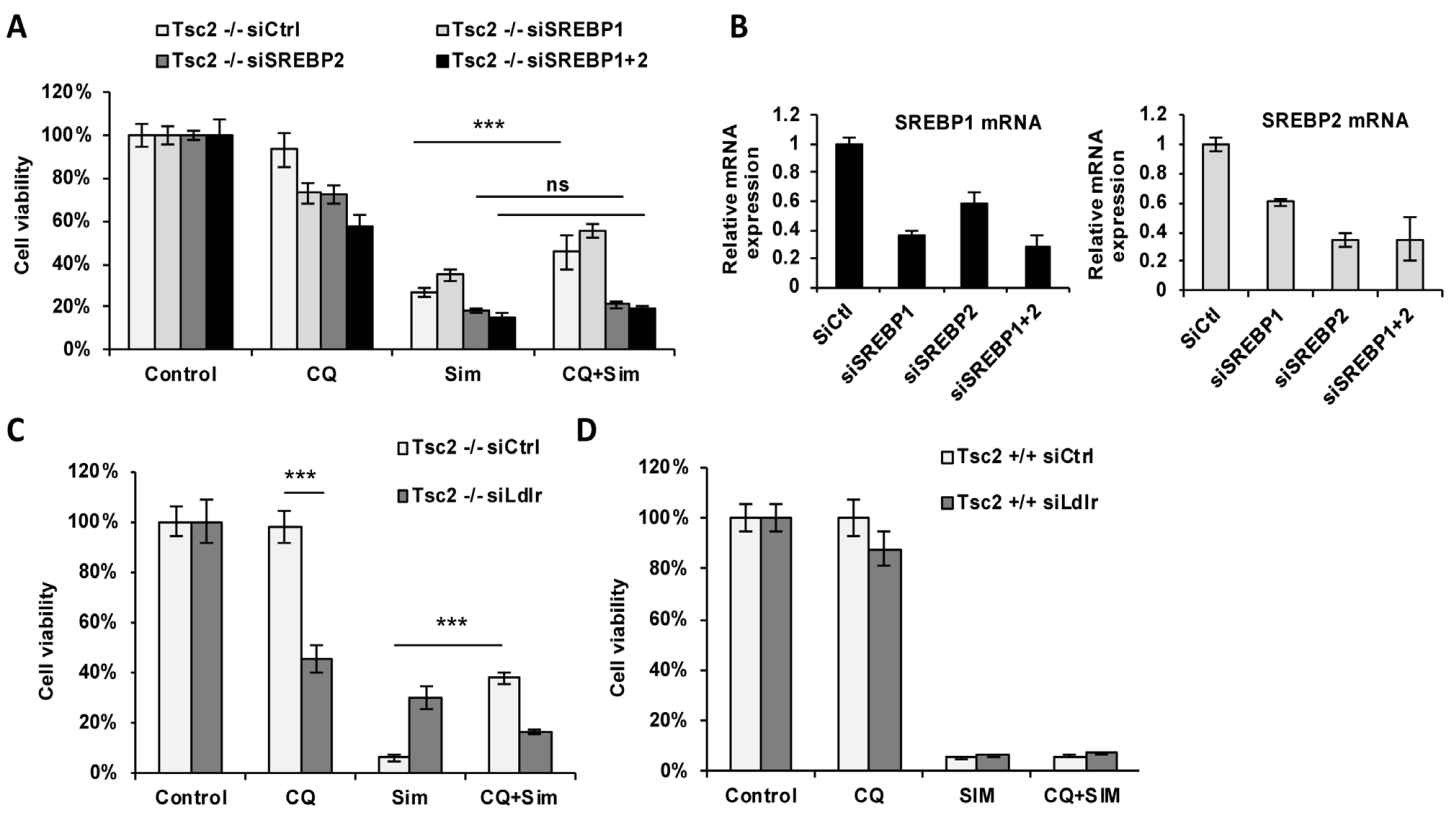
Exogenous lipoproteins mediate the cytoprotective role of CQ from simvastatin-induced cell death *via* SREBP2 and LDLR **A**. Cell viability of Tsc2^−/−^ MEFs transfected with siRNA against *SREBP1, SREBP2* or both and subsequently treated with vehicle control (DMSO), CQ (5uM), simvastatin (10uM) or the combination for 72 hours (crystal violet staining, 96 hrs post transfection). **B**. mRNA expression levels of *SREBP1* and *SREBP2* in each transfected cell line. Results represent experiment in panel A. **C**.-**D**. Cell viability of Tsc2^−/−^ and Tsc2^+/+^ MEFs following knockdown of *LDL-R* (24h) and treated with CQ (5uM), simvastatin (10uM) or the combination for 48 hours (crystal violet staining). Results are representative of three independent experiments. Bar graph represent means ±SD. Two-sample *t*-test was carried out between simvastatin and CQ combination and simvastatin treatment alone.

Since many of the identified CQ-dependent genes are transcriptionally regulated by Sterol Regulatory Element Binding Protein (SREBP), a master regulator of lipid and cholesterol homeostasis, we hypothesized that the cytoprotective effects of CQ are SREBP1/2-dependent. Therefore, we downregulated *SREBP1, SREBP2* or both in Tsc2^−/−^ MEFs and treated with CQ, simvastatin or the combination. Interestingly, downregulation of *SREBP2*, but not *SREBP1*, blocked the pro-survival effect of CQ in simvastatin-treated Tsc2^−/−^ MEFs following 72 hours of treatment (Figure 5A, 5B). These results suggest that CQ promotes cell survival of simvastatin-treated Tsc2^−/−^ MEFs by a mechanism involving SREBP2. We then hypothesized that the exogenous lipoprotein uptake is mediated by a SREBP2 transcriptional target, the lipoprotein receptor (LDLR). Genetic knockdown of *LDLR* using siRNA in Tsc2^−/−^ MEFs reversed their protection from simvastatin-induced cell death (Figure 5C-5E). Taken together, these results suggest that the CQ-mediated rescue of Tsc2^−/−^ MEFs from simvastatin induced cell death is mediated by exogenously supplied lipoproteins that enter the cell *via* SREBP2 and LDLR-dependent mechanisms.

### CQ-mediated rescue of TSC2-deficient cells depends on exogenous lipoproteins

**Figure 6:**
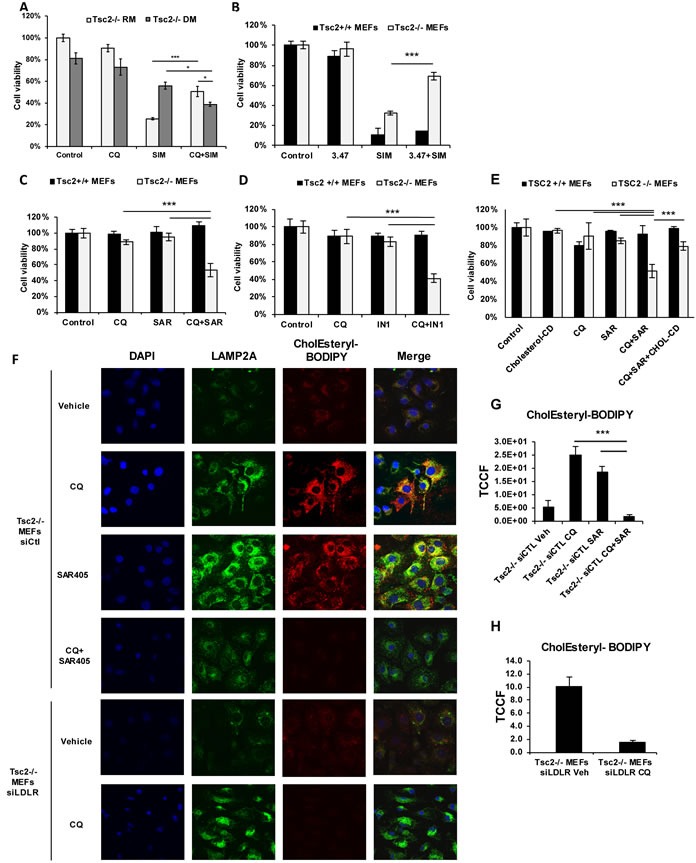
Survival of Tsc2^−/−^ MEFs depends on CQ-mediated uptake of exogenous cholesterol *via* the endosomal pathway **A**. Proliferation of Tsc2^+/+^ and Tsc2^−/−^ MEFs treated with vehicle control (DMSO), CQ (5uM), simvastatin (10uM) or the combination for 48 hrs. Cells were incubated with media supplemented with 10% FBS (RM) or 10% lipoprotein depleted FBS (DM). **B**. NPC1 inhibition (compound 3.47) protects Tsc2^−/−^ cells from simvastatin-induced cell death. Proliferation of Tsc2^+/+^ and Tsc2^−/−^ MEFs treated with vehicle control (DMSO), simvastatin (10uM), 3.47 (200nM), or the combination of simvastatin and AML or combination of simvastatin and compound 3.47 for 48 hrs (crystal violet staining). **C**. Lysosomal and endosomal inhibition selectively targets Tsc2^−/−^ MEFs. Proliferation of Tsc2^+/+^ and Tsc2^−/−^ MEFs treated with vehicle control (DMSO), CQ (5uM), Vps34 inhibitor (SAR405; 1uM) or the combination for 72 hours (crystal violet staining). **D**. Confirmation of selective growth inhibition using VPS34-IN1 inhibitor. Proliferation of Tsc2^+/+^ and Tsc2^−/−^ MEFs treated with vehicle control (DMSO), CQ (5uM), VPS34-IN1 (500nM) or the combination for 72 hours (crystal violet staining). Two-sample *t*-test was carried out between CQ and SAR405 and each drug alone. Results are representative of three independent experiments. Bar graph represent means ±SD. **E**. Growth inhibition of Tsc2^−/−^ MEFs by CQ and SAR405 is cholesterol dependent. Proliferation of Tsc2^+/+^ and Tsc2^−/−^ MEFs treated with vehicle control (DMSO), Cholesterol-Cyclodextrin (2ug/ml), CQ (5uM), Vps34 inhibitor (SAR405; 1uM) or the combination for 72 hours (crystal violet staining). **F**. Exogenously supplied CholEsteryl-BODIPY (1ug/ml; 4 hours) accumulates in distinctive puncta in siRNA-control *Tsc2−/−* MEFs pretreated with CQ (5uM) or SAR405 (1uM) for 16 hours. siRNA-Ldlr transfected *Tsc2^−/−^* MEF pretreated with CQ show no accumulation of exogenous cholesterol esters. **G**. Quantification of total corrected cell fluorescence (TCCF) of CholEsteryl-BODIPY in CQ, SAR405 and their combination. **H**. Quantification of total corrected cell fluorescence (TCCF) of CholEsteryl-BODIPY in siLDLR transfected Tsc2^−/−^ MEFs treated with CQ or vehicle control. Image acquisition was carried out using identical exposure settings. Two sample *t*-tests were used. ***p < 0.001. See also Supplementary Figure S2.

To prove that the pro-survival effects of CQ are dependent on exogenous lipoproteins, we seeded Tsc2^−/−^ MEFs in media supplemented with 10% lipoprotein-depleted serum (DM) or 10% regular serum (RM) and treated with CQ (5uM), simvastatin (10uM) or combination. Interestingly, cells grown in lipoprotein-depleted conditions showed resistance to simvastatin treatment, compared to cells grown in regular conditions. We hypothesize that this is the consequence of compensatory upregulation of *de novo* cholesterol synthesis in the lipoprotein-depleted setting. (Figure 6A). Importantly, the viability of Tsc2^−/−^ MEFs grown in lipoprotein depleted conditions was significantly decreased when treated with the combination of CQ and simvastatin, compared to cells grown in physiological serum conditions. Therefore, uptake of exogenous cholesterol mediates the survival of Tsc2^−/−^ MEFs when lysosomal function is inhibited.

### Inhibition of cholesterol trafficking from the lysosome phenocopies CQ-mediated effects

Since chloroquine impairs lysosomal function, we next asked whether inhibition of cholesterol transport from the lysosome to the cytoplasm would selectively inhibit proliferation of Tsc2^−/−^ MEFs under simvastatin-treatment conditions. We utilized a catalytic inhibitor of Niemann-Pick Disease Type 1 (NPC1), compound 3.47 (200nM), which prevents cholesterol egress from the lysosome [24, 25]. The combination of 3.47 with simvastatin (10uM) increased the survival of Tsc2^−/−^ but not Tsc2^+/+^ MEFs compared to simvastatin alone at 48 hrs (Figure 6B), phenocopying the results of CQ and simvastatin combination treatment. This suggests that cholesterol esters accumulating in the lysosome following NPC1 inhibition, allows Tsc2^−/−^ MEFs to survive simvastatin-induced cell death probably by a compensatory mechanism.

Since CQ inhibits autophagy, we investigated whether the CQ-mediated effects on TSC2-deficient cell viability are also autophagy dependent. Simvastatin had similar effects on the proliferation of Atg5^+/+^ and Atg5^−/−^ MEFs stably transduced with shRNA against *Tsc2* (Supplementary Figure S1), indicating that these effects are autophagy-independent. Taken together, these results demonstrate that TSC2-deficient cells activate mechanisms upstream of the lysosome to uptake and process lipoprotein cargo upon treatment with CQ, thereby enhancing cell survival.

### CQ and PI3KC3 inhibition selectively inhibits growth of Tsc2-deficient cells

We hypothesized that upon lysosomal inhibition and *de novo* cholesterol synthesis inhibition, Tsc2-deficient cells utilize exogenous cholesterol processed by acid lipase, which hydrolyses cholesterol esters and triglycerides in both endosomal and lysosomal compartments [26]. SAR405 and VPS34-IN1 are ATP-competitive inhibitors of Vps34, Phosphatidylinositol 3-Kinase Catalytic Subunit Type 3 (PI3KC3) [27, 28]. PI3KC3 is involved in the initiation and maturation of autophagosome formation, and thereby regulates endocytic trafficking and delivery of enzymes to lysosomes. Simultaneous treatment with CQ (5uM) and either SAR405 (1uM) or VPS34-IN1 (1uM) for 72 hrs resulted in selective and significant inhibition of growth of Tsc2^−/−^ MEFs (Figure 6C, 6D). To determine whether this effect occurs by inhibition of utilization of exogenous cholesterol, we treated Tsc2^+/+^ and Tsc2^−/−^ MEFs with CQ (5uM), SAR405 (1uM) and Cholesterol/Cyclodextrin (2ug/ml) for 48hrs. Interestingly, supplementation of Tsc2-deficient cells with cholesterol, rescued from CQ and SAR405-induced cell death (Figure 6E). In addition, esterified cholesterol accumulation was hindered when Tsc2^−/−^ MEFs were treated with the combination of CQ and SAR405 for 16 hours in Tsc2^−/−^ but not Tsc2^+/+^ MEFs (Figure 6F-6H and Supplementary Figure S2). Moreover, genetic knockdown of *LDLR* resulted in almost complete inhibition of CholEsteryl-BODIPY uptake in Tsc2^−/−^ MEFs, confirming that CholEsteryl-BODIPY is selectively taken up by LDLR (Figure 6H). These results indicate that endosomal cholesterol trafficking is involved in CQ-mediated survival of Tsc2^−/−^ cells, thereby facilitating survival when *de novo* synthesis and lysosomal processing of cholesterol are inhibited.

## DISCUSSION

Tuberous sclerosis complex is a multisystem disease in which inactivation of the *TSC1* or *TSC2* gene leads to mTORC1 hyperactivation. The impact of TSC1/2 deficiency on the lysosome is not completely understood. We report here that inhibition of lysosomal function by chloroquine results in a striking transcriptional signature in TSC2-deficient cells, with upregulation of genes involved in regulation of cholesterol homeostasis in TSC2-deficient cells treated with CQ but not in TSC2-expressing cells. This transcriptional signature is associated with increased accumulation and intracellular levels of both total cholesterol and cholesterol ester levels in Tsc2^−/−^ MEFs treated with CQ, whereas free cholesterol levels remained unchanged. Unexpectedly, engaging this CQ-mediated cholesterol uptake pathway together with inhibition of *de novo* cholesterol synthesis is cytoprotective in TSC2-deficient, but not TSC2-expressing cells, highlighting its physiologic importance. Most importantly, simultaneous inhibition of the lysosome and endosomal trafficking inhibits TSC2-deficient cell growth, but not growth of TSC2-expressing cells, suggesting that these reprogrammed metabolic pathways that are initiated by CQ have translational potential.

mTORC1 regulates multiple biosynthetic processes that support cell growth and proliferation and can promote the survival of TSC2-deficient cells under stress conditions [29, 30]. Lysosomal degradation of extracellular proteins is essential for survival and activation of mTORC1, in a mechanism independent of autophagy [31]. Our data indicate that the lysosome, but not autophagy, plays a critical role in titrating lipid uptake in mTORC1 hyperactive cells. mTORC1 is known to induce a transcriptional program to drive lipid synthesis through activation of SREBP1 and 2 [1, 32]. We found that the lysosome regulates the balance between *de novo* lipid synthesis, *via* SREBP2, and the uptake of exogenous lipids, *via* LDLR and endocytic trafficking. Therefore, both the biosynthesis and the uptake of cholesterol are increased when the lysosome is inhibited with chloroquine. The net result of lysosomal inhibition is an increase of total cholesterol and cholesterol ester levels and constant levels of free cholesterol in TSC2-deficient cells. Through an analogous mechanism, it was recently shown that mTORC1 signaling suppresses lysosomal catabolism of extracellular proteins favoring *de novo* synthesis and autophagic turnover of proteins [31].

We were surprised to find that simultaneous inhibition of *de novo* cholesterol synthesis by simvastatin and the lysosome with CQ resulted in a pro-survival phenotype that is specific to TSC2-deficient cells (Figure 3B). Both SREBP2 and LDLR are required for this CQ pro-survival phenotype, indicating a mechanism involving exogenous uptake of lipoproteins. mTORC1 is known to upregulate LDLR, which mediates uptake of circulating cholesterol-rich LDL from the plasma into the liver and peripheral tissues. LDLR gene expression is controlled by SREBP, while mTORC1 signaling is known to repress the expression of PCSK9, a negative regulator of LDLR protein levels [33, 34]. Taken together, these data support a critical role of the lysosome in regulating the mTORC1-dependent balance between lipid uptake and lipid synthesis.

Most mammalian cells acquire ~80% of their cholesterol by receptor-mediated endocytosis of plasma LDL [35]. Delivery of LDL to the lysosomes involves the endosomal trafficking of clathrin-coated LDL particles through early to late endosomes which eventually fuse with the lysosome to deliver the lipid cargo [36]. We found that cholesterol esters are significantly enhanced in TSC2-deficient cells treated with chloroquine, pointing toward a TSC2-dependent compensatory cholesterol processing mechanism (Figure 2A). Lysosomal egress of free cholesterol is regulated by NPC1, which resides at the lysosomal lumen [37, 38]. Consistent with the concept that NPC1 function is critical to the lysosome in TSC2-deficient cells, we found that NPC1-inhibition phenocopies the effects of CQ on cell proliferation (Figure 5B). This may be linked to our recent finding that cholesteryl esters are upregulated in TSC2-deficient cells, *via* an SREBP1/2 dependent mechanism [32].

Vps34 is a crucial component of endosome formation and successful cholesterol transport to the lysosome. Vps34 is a lipid kinase that recruits proteins containing phosphatidylinositol to intracellular membranes [39]. Vps34 has also been implicated in regulation of mTORC1 response to nutrient sensing by regulating autophagy activation [40, 41]. We hypothesized that Vps34-dependent delivery of exogenous cholesterol to the lysosome contributes to the unexpected enhanced survival of TSC2-deficient cells treated with CQ and simvastatin. Consistent with this hypothesis, combinational inhibition of Vps34 using SAR405 or VPS34-IN1 together with inhibition of the lysosome with CQ resulted in selective growth inhibition of TSC2-deficient cells (Figure 6C, 6D). Furthermore, add-back of cholesterol to CQ and SAR405-treated Tsc2-deficient cells restored their proliferation, showing that exogenously supplied cholesterol is crucial for the survival of Tsc2-deficient cells upon lysosomal inhibition. In addition to effects on proliferation, the combination of CQ and Vps34 inhibition resulted in striking inhibition of the LDLR-mediated accumulation of exogenous cholesterol esters in Tsc2^−/−^ MEFs (Figure 6F-6H). Pharmacologic inhibition of Vps34 has been shown to disrupt endosomal trafficking, and simultaneous inhibition of Vps34 and mTORC1 reduces the proliferation of renal tumor-derived cells [27]. Taken together, these results reveal a novel connection between cholesterol homeostasis, endosomes and lysosomes that is specific to cells with mTORC1 hyperactivation, with important implications for both the pathogenesis and therapy of TSC. Furthermore, these findings can be applied in xenograft animal models of TSC, in which combination treatment with CQ and SAR-405 is expected to reduce tumor burden.

**Figure 7:**
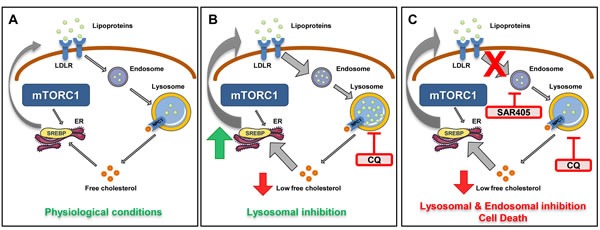
Model of lysosomal-mediated regulation of cholesterol homeostasis in mTORC1 hyperactive cells In physiological conditions, free cholesterol is derived from two main sources: LDLR-mediated uptake and subsequent endosomal/lysosomal processing and SREBP-mediated *de novo* synthesis. **A**. In TSC2-deficient cells with mTORC1 hyperactivation, increased cholesterol synthesis is regulated by the transcription factor SREBP. **B**. Lysosomal inhibition of TSC2-deficient cells with CQ activates compensatory mechanisms for maintaining cholesterol levels, involving both SREBP-mediated activation of the mevalonate pathway and uptake of exogenous lipoprotein uptake *via* LDLR. In TSC2-deficient (but not TSC2-expressing) cells co-treated with simvastatin, the uptake of exogenous lipoprotein via LDLR is sufficient to over-ride the impact of simvastatin and promote cell survival. **C**. In TSC2-deficient cells, but not TSC2-expressing cells, combination treatment with CQ and SAR405, a PI3KC3 inhibitor, blocks uptake of exogenous cholesterol esters and inhibits cell survival.

In summary, we demonstrate here that lysosomal inhibition by chloroquine increases exogenous cholesterol uptake and overcomes the pro-apoptotic effects of mevalonate pathway inhibition in TSC2-deficient cells. Exogenous cholesterol uptake becomes critical for survival of TSC2-deficient cells when *de novo* synthesis and lysosomal processing of cholesterol are inhibited, and the endosomal pathway plays a crucial role in regulating survival of TSC2-deficient cells. Importantly, the chloroquine-mediated pro-survival effects on TSC2-deficient cells are mediated by SREBP2 and LDLR, indicating a tightly coordinated mechanism that is activated when lysosomal function is inhibited to maintain cholesterol homeostasis (Figure 7). Remarkably, this mechanism is specific to TSC2-deficient cells, in which lipid metabolism is extensively reprogrammed. Our finding that simultaneous inhibition of the lysosome and endosomal trafficking inhibits the proliferation of TSC2-deficient cells provides a novel potential therapeutic avenue for the treatment of TSC and other diseases associated with mTORC1 hyperactivation, including lymphangioleiomyomatosis (LAM) and the majority of human malignancies.

## EXPERIMENTAL PROCEDURES

### Cell lines, culture conditions, drugs

621-101 cells were derived from an angiomyolipoma and carry bi-allelic inactivation of the TSC2 gene [42, 43]. The 621-102 cell line was generated by introduction of E6/E7 (pLXSN 16E6E7-neo) and human telomerase (pLXSN hTERT-hyg) into a primary culture of *TSC2* null human angiomyolipoma cells [44]. 621-103 was generated by stable transfection of TRI102 with wild-type *TSC2* (pcDNA3.1 TSC2-zeo) into 621-101 cells [42].

Tsc2^−/−^p53^−/−^ and Tsc2^+/+^p53^−/−^ mouse embryonic fibroblasts (MEFs) were provided by Dr. David Kwiatkowski (Brigham and Women's Hospital, Boston, MA).

To stably re-express TSC2 in 105K cells, full length human TSC2 in pLXIN-IRES-hygromycin vector or empty vector were introduced by retroviral infection [45]. Briefly, full length human TSC2 in pLXIN-IRES-hygromycin vector, or empty vector, were transfected into the PT67 retroviral packaging cell line (ATCC #CRL-12284) using lipofectamine 2000 according to manufacturer's instructions. 4 hrs later, cells were washed with PBS and fresh media was added. 48hrs later, virus-containing media was collected and filtered through a 0.45um PES filter. Virus-containing media was added to 105K cells with 4ug/mL polybrene and plates were spun at 600rcf for 45min before returning to the incubator. 6hrs later, fresh media was added to the plates in a 1:1 ratio with the virus-containing media. 24hrs later, cells were washed with PBS and fresh media was added with 100ug/mL hygromycin B. Single cell clones were grown in the presence of 100ug/mL hygromycin B and screened by western blot for TSC2 expression and serum/growth factor-independent mTORC1 activation.

Unless otherwise specified, cells were cultured in Dulbecco's modified Eagle medium with 4.5 g/l glucose supplemented with 10% FBS, 100 μg/mL penicillin and 100 μg/mL streptomycin. For cholesterol starvation experiments cells were cultured in DMEM with 4.5g/l glucose supplemented with 10% Lipoprotein-depleted FBS obtained from Kalen Biomedical (Montgomery Village, MD). All cells tested negative for mycoplasma contamination using MycoAlert (Lonza, Walkersville, MD) and were re-tested monthly. Chloroquine diphosphate salt, simvastatin and mevalonic acid were obtained from Sigma-Aldrich. SAR405 was obtained from ApexBio (Houston, TX) and VPS34-IN1 from Selleckchem (Houston, TX).

### L-1000 high throughput expression profiling

mRNA from cell lysates is amplified using ligation mediated amplification [21]. mRNA probes are annealed to reversely transcribed complementary deoxyribonucleic acid (cDNAs), created from the isolated mRNAs, and then ligated by a taq ligase. Probes are then amplified by PCR and hybridized to barcoded Luminex beads through the probes' gene specific barcode region. The hybridized beads are then detected and quantified using the Luminex technology. This technology utilizes laser beams to detect the identity of the differentially-dyed beads and to measure the density of the hybridized probes on each bead [23].

### mRNA expression analysis

Two micrograms of total RNA (RNeasy MicroKit; Qiagen Inc., Valencia, CA, USA) were retrotranscribed with the High-Capacity cDNA Reverse-transcription kit (Applied Biosystems, Grand Island, NY, USA). Forty nanograms of cDNA per reaction were run using TaqMan probes (Applied Biosystems) in an Applied Biosystems Instrument. Results were normalized to actin, which had stable expression in our experimental conditions, and analyzed using the Delta Delta Ct method. Final values were expressed as n-fold the calibrator.

### Cholesterol quantification

Cholesterol extraction and quantification was performed using Chloroform: Isopropanol: NP-40 (7:11:0.1) per the manufacturer's instructions (Abcam, Cambridge, UK). Cholesterol-Cholesteryl Ester quantification was performed by detecting total cholesterol (cholesterol and cholesteryl esters) in the presence of cholesterol esterase or free cholesterol in the absence of cholesterol esterase in the reaction. Quantification of fluorescent signal was performed using a plate reader (BioTek, Winooski, VT, USA). Cholesterol ester was determined by subtracting the value of free cholesterol from the total (cholesterol plus cholesteryl esters). Normalization was carried out for total protein.

### Fluorescent cholesteryl-ester uptake assay and immunofluorescent labeling

Cells were seeded on 4-chamber glass slides and grown in 1% FBS DMEM overnight. Treatment with CQ was carried out for 16 hours followed by addition of 1μg/ml CholEsteryl-BODIPY dissolved in DMSO for 4 hours. Cells were fixed using 4% PFA. Immunofluorescent labeling was performed using Lamp2A and Alexa-488 antibodies (Invitrogen, Carlsbad, CA, USA). To quantify CholEsteryl-BODIPY an outline was drawn around each cell (n≥15) and circularity, area and mean fluorescence were measured, along with several adjacent background readings. Total corrected cell fluorescence (TCCF) = integrated density - (area of selected cell × mean fluorescence of background readings) [46]. The colocalization signal for each condition was measured from four representative fields, n≥15 cells, using ImageJ. The colocalization index represents the Pearson's coefficient (zero is no colocalization and one means perfect colocalization).

### Confocal microscopy

Cells were seeded on 4-chamber tissue culture glass slides overnight. Cells were rinsed with PBS twice, fixed with 4% paraformaldehyde, permeabilized with 0.2% Saponin, blocked in 3% BSA/PBS for 1 h, incubated with primary antibody 1% BSA in PBS overnight at 4°C, and then incubated with secondary antibodies for 1 h. Images were captured with a FluoView FV-10i Olympus Laser Point Scanning Confocal Microscope using a 60x objective. Confocal filters (Excitation/Emission nm) used for microscopy imaging were: 358/461 (DAPI), 542/563 (CholEsteryl-BODIPY) and 490/525 (Alexa-Fluor488). Antibodies of LAMP2A and Alexa-Fluor488 where purchased from Invitrogen, Carlsbad, CA, USA).

### Cell proliferation assay (crystal violet staining)

Cells were plated in 96-well plates. After treatment for 24, 48, 72 or 96 hours, cells were fixed with 10% formalin for 10 minutes, stained with 0.05% crystal violet in distilled water for 30 minutes, washed two times, and air-dried. Crystal violet was solubilized with 100 ml of methanol, and read with a plate reader (OD 540; BioTek, Winooski, VT, USA).

### Caspase 3/7 measurement

Following treatments cells were subjected to Caspase 3/7 activities measurement with Caspase-Glo assay kit (Promega, Madison USA). Briefly, 1000 cells were plated on each well of a 96-well plate, and treated with the described compounds. 100 μl of Caspase-Glo reagent was added to each well, the content of well was gently mixed with a plate shaker at 300-500 rpm for 30 seconds. The plate was then incubated at room temperature for 2 hours. A plate-reading luminometer was used to measure luminescence with parameters of 1 minute lag time and 0.5 second/well, read time (BioTek, Winooski, VT, USA). The experiments were performed in triplicate.

### Small interfering RNA transfections

Small interfering RNA (siRNA) Smartpools targeting *SREBP1, SREBP2, LDLR* and nontargeting controls were obtained from Dharmacon (Lafayette, CO, USA) and used at 25 nM final concentration. Cells were transfected with Lipofectamine-RNAiMAX Reagent Invitrogen, Carlsbad, CA, USA) as previously described [31].

### Statistics

Statistical significance was determined using GraphPad Prism software. Differences were considered statistically significant at *p* < 0.05. Results are expressed as mean values ± SD of the indicated number of observations.

## SUPPLEMENTARY FIGURES AND TABLE




