# Supplementary material for: Lysosomal regulation of cholesterol homeostasis in tuberous sclerosis complex is mediated via NPC1 and LDL-R
Source: Oncotarget. 2017 Apr 27;8(24):38099–112. doi: 10.18632/oncotarget.17485 (PMC5503518; doi:10.18632/oncotarget.17485)
Supplement: Supplementary file 1 [file oncotarget-08-38099-s001.pdf]

Lysosomal regulation of cholesterol homeostasis in tuberous sclerosis complex is mediated *via* NPC1 and LDL-R

Supplementary Material

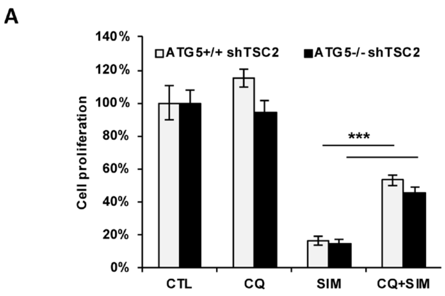

Supplementary figure 1. CQ-mediated TSC2-deficient cell survival from simvastatin-induced cell death is autophagy independent.

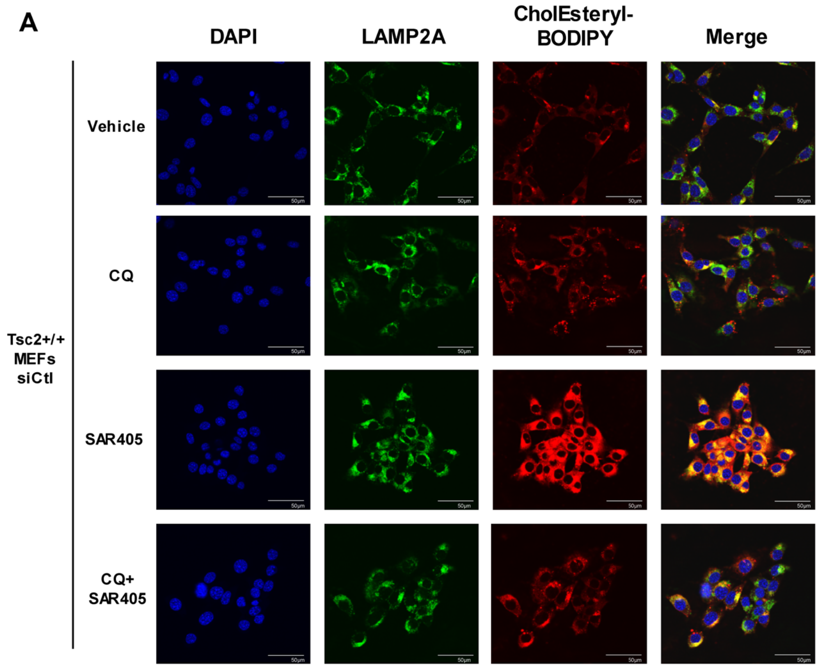

Supplementary figure 2. Combinational inhibition of lysosome and endosome in Tsc2+/+ MEFs has no effect on CholEsteryl-BODIPY accumulation.
