# Supplementary material for: Lysosomal regulation of cholesterol homeostasis in tuberous sclerosis complex is mediated via NPC1 and LDL-R
Source: Oncotarget. 2017 Apr 27;8(24):38099–112. doi: 10.18632/oncotarget.17485 (PMC5503518; doi:10.18632/oncotarget.17485)
Supplement: Supplementary file 2 [file oncotarget-08-38099-s002.docx]

| 621-101 |  |  |  |  |  |  | 5uM Chloroquine -24hrs |  |  |  |  |  | H2O treatment |  |  |  |  |  |
| --- | --- | --- | --- | --- | --- | --- | --- | --- | --- | --- | --- | --- | --- | --- | --- | --- | --- | --- |
| id | pr_gene_id | pr_gene_symbol | pr_gene_title | Fold Change(FC) | p_value | FDR(BH) | HEN004_621-101_XH_X1.L2_B21:E01 | HEN004_621-101_XH_X1.L2_B21:E03 | HEN004_621-101_XH_X1.L2_B21:E05 | HEN004_621-101_XH_X1.L2_B21:E07 | HEN004_621-101_XH_X1.L2_B21:E09 | HEN004_621-101_XH_X1.L2_B21:E11 | HEN004_621-101_XH_X1.L2_B21:A13 | HEN004_621-101_XH_X1.L2_B21:A15 | HEN004_621-101_XH_X1.L2_B21:A17 | HEN004_621-101_XH_X1.L2_B21:A19 | HEN004_621-101_XH_X1.L2_B21:A21 | HEN004_621-101_XH_X1.L2_B21:A23 |
| 203123_s_at | 4891 | SLC11A2 | solute carrier family 11 (proton-coupled divalent metal ion transporters), member 2 | 4.9451 | 0.001996 | 0.03683 | 8.109 | 7.994 | 8.1122 | 8.2638 | 8.1034 | 8.009 | 6.6032 | 6.5104 | 6.4795 | 6.4611 | 7.0095 | 6.5426 |
| 201626_at | 3638 | INSIG1 | insulin induced gene 1 | 4.8444 | 0.001996 | 0.03683 | 12.1105 | 11.8192 | 11.5278 | 11.5736 | 11.5736 | 11.2558 | 9.5248 | 9.1737 | 9.4687 | 9.1881 | 9.3686 | 9.5933 |
| 221750_at | 3157 | HMGCS1 | 3-hydroxy-3-methylglutaryl-CoA synthase 1 (soluble) | 4.605 | 0.001996 | 0.03683 | 12.1267 | 11.7221 | 11.3002 | 11.7111 | 11.4922 | 11.8527 | 9.6105 | 9.6925 | 9.3073 | 9.18 | 9.3502 | 9.5783 |
| 201849_at | 664 | BNIP3 | BCL2/adenovirus E1B 19kDa interacting protein 3 | 4.1327 | 0.001996 | 0.03683 | 11.7715 | 11.4593 | 11.8192 | 11.8922 | 11.7221 | 11.5736 | 9.7626 | 9.9764 | 9.1754 | 9.7119 | 9.5971 | 10.0829 |
| 203665_at | 3162 | HMOX1 | heme oxygenase (decycling) 1 | 3.4346 | 0.001996 | 0.03683 | 12.6305 | 12.7437 | 12.417 | 12.417 | 12.5912 | 12.4521 | 11.3906 | 11.5382 | 10.9837 | 11.4145 | 11.2169 | 10.9477 |
| 202679_at | 4864 | NPC1 | Niemann-Pick disease, type C1 | 3.4256 | 0.001996 | 0.03683 | 10.1435 | 10.1993 | 9.9902 | 9.9167 | 9.6968 | 10.0604 | 8.5757 | 8.7161 | 8.941 | 8.3903 | 8.8705 | 8.7049 |
| 221478_at | 665 | BNIP3L | BCL2/adenovirus E1B 19kDa interacting protein 3-like | 3.3861 | 0.001996 | 0.03683 | 8.714 | 8.8266 | 8.5681 | 8.6611 | 8.8416 | 8.5944 | 7.5755 | 7.7844 | 7.2245 | 7.3114 | 7.6635 | 7.708 |
| 202382_s_at | 10007 | GNPDA1 | glucosamine-6-phosphate deaminase 1 | 3.3434 | 0.001996 | 0.03683 | 10.8479 | 10.908 | 10.5491 | 10.6859 | 10.7601 | 10.6859 | 9.5703 | 9.809 | 9.3635 | 9.804 | 9.8461 | 9.7268 |
| 200766_at | 1509 | CTSD | cathepsin D | 2.6682 | 0.001996 | 0.03683 | 10.7254 | 11.0885 | 10.908 | 10.7601 | 11.0165 | 10.8176 | 10.2737 | 10.1926 | 9.9167 | 10.2247 | 10.1771 | 10.1993 |
| 201471_s_at | 8878 | SQSTM1 | sequestosome 1 | 2.5863 | 0.001996 | 0.03683 | 14.1383 | 14.3206 | 14.3206 | 14.4357 | 14.3206 | 14.3206 | 13.5329 | 13.4469 | 13.2745 | 13.5329 | 13.4075 | 13.9002 |
| 208647_at | 2222 | FDFT1 | farnesyl-diphosphate farnesyltransferase 1 | 2.4797 | 0.001996 | 0.03683 | 11.9303 | 11.8922 | 11.8403 | 11.7544 | 11.7544 | 11.7111 | 9.3953 | 10.5073 | 10.6859 | 10.7843 | 10.459 | 10.3126 |
| 206662_at | 2745 | GLRX | glutaredoxin (thioltransferase) | 2.281 | 0.001996 | 0.03683 | 12.4808 | 12.5394 | 12.3797 | 12.2657 | 12.246 | 12.1907 | 11.8922 | 11.8824 | 11.8922 | 11.7715 | 11.9623 | 11.7779 |
| 203221_at | 7088 | TLE1 | transducin-like enhancer of split 1 (E(sp1) homolog, Drosophila) | 2.1718 | 0.001996 | 0.03683 | 9.4948 | 9.7275 | 9.6129 | 9.631 | 9.9424 | 9.5801 | 8.8416 | 9.039 | 8.6032 | 8.941 | 8.941 | 9.153 |
| 201536_at | 1845 | DUSP3 | dual specificity phosphatase 3 | 2.1367 | 0.001996 | 0.03683 | 9.2979 | 9.5548 | 9.2744 | 8.9476 | 9.3913 | 9.4881 | 7.6827 | 7.852 | 7.509 | 7.479 | 7.8993 | 8.7568 |
| 202637_s_at | 3383 | ICAM1 | intercellular adhesion molecule 1 | 2.0427 | 0.001996 | 0.03683 | 7.863 | 7.5316 | 7.8568 | 7.7145 | 7.9728 | 8.1256 | 7.241 | 7.1751 | 6.8421 | 6.6394 | 6.868 | 7.0204 |
| 219051_x_at | 79006 | METRN | meteorin, glial cell differentiation regulator | 2.0417 | 0.001996 | 0.03683 | 7.4254 | 7.6563 | 7.3065 | 7.1638 | 7.5755 | 7.3828 | 6.7388 | 6.5842 | 6.0527 | 6.661 | 6.6478 | 6.5948 |
| 213702_x_at | 427 | ASAH1 | N-acylsphingosine amidohydrolase (acid ceramidase) 1 | 1.9782 | 0.001996 | 0.03683 | 10.2247 | 10.3719 | 10.2247 | 10.1993 | 10.1926 | 10.0527 | 9.5434 | 9.6788 | 9.3614 | 9.5434 | 9.9424 | 9.6788 |
| 200678_x_at | 2896 | GRN | granulin | 1.952 | 0.001996 | 0.03683 | 11.3122 | 11.3906 | 10.972 | 11.3254 | 11.5382 | 11.0671 | 10.1514 | 10.7254 | 10.5013 | 10.3354 | 10.6137 | 10.2876 |
| 212665_at | 25976 | TIPARP | TCDD-inducible poly(ADP-ribose) polymerase | 1.9472 | 0.001996 | 0.03683 | 9.1224 | 9.153 | 9.1181 | 9.0984 | 9.1448 | 9.0374 | 8.6465 | 8.9263 | 8.7093 | 8.9476 | 8.7752 | 8.6754 |
| 200886_s_at | 5223 | PGAM1 | phosphoglycerate mutase 1 (brain) | 1.9127 | 0.001996 | 0.03683 | 13.2745 | 13.5329 | 13.4075 | 13.5329 | 13.5329 | 13.4075 | 12.8415 | 13.1644 | 12.7014 | 12.7892 | 13.0802 | 12.8646 |
| 203068_at | 9903 | KLHL21 | kelch-like 21 (Drosophila) | 1.9015 | 0.001996 | 0.03683 | 6.102 | 6.1358 | 5.9813 | 6.0728 | 5.7482 | 6.2018 | 5.4729 | 5.5373 | 5.1167 | 5.3205 | 5.3097 | 5.6058 |
| 201968_s_at | 5236 | PGM1 | phosphoglucomutase 1 | 1.8667 | 0.001996 | 0.03683 | 10.0808 | 10.1486 | 9.8854 | 9.7678 | 10.1099 | 9.8461 | 9.0083 | 9.4533 | 8.7482 | 9.148 | 9.388 | 9.3223 |
| 218498_s_at | 30001 | ERO1L | ERO1-like (S. cerevisiae) | 1.8403 | 0.001996 | 0.03683 | 6.9347 | 6.7353 | 6.618 | 6.6738 | 6.9125 | 6.6394 | 6.0314 | 6.0363 | 5.8306 | 5.5606 | 5.9889 | 6.4201 |
| 202735_at | 10682 | EBP | emopamil binding protein (sterol isomerase) | 1.8102 | 0.001996 | 0.03683 | 9.3686 | 9.1224 | 8.941 | 9.3614 | 9.0829 | 9.2051 | 8.3278 | 8.2951 | 7.2999 | 8.2999 | 8.2669 | 8.326 |
| 202324_s_at | 64746 | ACBD3 | acyl-CoA binding domain containing 3 | 1.8065 | 0.001996 | 0.03683 | 9.2225 | 8.907 | 8.8314 | 9.0407 | 9.0276 | 9.0651 | 8.5389 | 8.6279 | 8.4038 | 8.4327 | 8.7387 | 8.5389 |
| 202604_x_at | 102 | ADAM10 | ADAM metallopeptidase domain 10 | 1.7772 | 0.005988 | 0.0805 | 9.6384 | 9.6671 | 9.4001 | 9.5248 | 9.6755 | 9.6523 | 9.1448 | 9.2245 | 8.8705 | 9.2264 | 9.2744 | 9.0602 |
| 201870_at | 10953 | TOMM34 | translocase of outer mitochondrial membrane 34 | 1.7743 | 0.001996 | 0.03683 | 11.5542 | 11.5542 | 11.6213 | 11.6793 | 11.7993 | 11.7544 | 11.1208 | 11.3002 | 10.5765 | 10.9837 | 11.2124 | 10.8361 |
| 202208_s_at | 10123 | ARL4C | ADP-ribosylation factor-like 4C | 1.767 | 0.001996 | 0.03683 | 8.3198 | 8.5534 | 8.3263 | 8.5475 | 8.5475 | 8.4583 | 8.1034 | 8.0653 | 8.2196 | 7.8309 | 7.9707 | 7.9685 |
| 207871_s_at | 7982 | ST7 | suppression of tumorigenicity 7 | 1.7404 | 0.001996 | 0.03683 | 5.5606 | 5.4828 | 5.5373 | 5.614 | 5.4539 | 5.4117 | 5.0832 | 5.2689 | 5.005 | 5.101 | 5.0889 | 5.3545 |
| 209092_s_at | 51031 | GLOD4 | glyoxalase domain containing 4 | 1.7325 | 0.001996 | 0.03683 | 9.3614 | 9.2487 | 9.3635 | 9.2225 | 9.148 | 9.4533 | 8.5177 | 8.9854 | 8.4919 | 8.8343 | 8.904 | 8.7786 |
| 201170_s_at | 8553 | BHLHE40 | basic helix-loop-helix family, member e40 | 1.7274 | 0.001996 | 0.03683 | 5.3719 | 5.5235 | 5.3205 | 5.3633 | 5.5856 | 5.4539 | 5.101 | 5.005 | 4.2725 | 4.6715 | 4.5291 | 4.7929 |
| 201000_at | 16 | AARS | alanyl-tRNA synthetase | 1.7112 | 0.001996 | 0.03683 | 9.4059 | 9.6721 | 9.6788 | 9.7275 | 9.7584 | 9.6968 | 9.0957 | 9.1881 | 9.0132 | 9.2134 | 9.4533 | 9.0922 |
| 200768_s_at | 4144 | MAT2A | methionine adenosyltransferase II, alpha | 1.6501 | 0.001996 | 0.03683 | 12.3797 | 12.417 | 12.5651 | 12.5073 | 12.5394 | 12.4808 | 12.2657 | 12.3115 | 12.1907 | 12.3315 | 12.2657 | 12.3115 |
| 203379_at | 6195 | RPS6KA1 | ribosomal protein S6 kinase, 90kDa, polypeptide 1 | 1.6032 | 0.001996 | 0.03683 | 8.0281 | 8.2368 | 8.1495 | 8.135 | 8.2329 | 8.3629 | 7.7844 | 7.9809 | 7.3678 | 7.716 | 7.5658 | 7.4527 |
| 219888_at | 6676 | SPAG4 | sperm associated antigen 4 | 1.6029 | 0.003992 | 0.06972 | 5.0889 | 5.1697 | 4.6715 | 5.1836 | 5.1697 | 4.9409 | 4.4625 | 4.231 | 4.0394 | 4.6498 | 4.4787 | 4.0809 |
| 202368_s_at | 9697 | TRAM2 | translocation associated membrane protein 2 | 1.5907 | 0.001996 | 0.03683 | 9.1423 | 8.941 | 8.7161 | 8.7482 | 8.8784 | 8.7387 | 8.1169 | 8.5903 | 7.9299 | 8.3263 | 8.3111 | 8.1992 |
| 201631_s_at | 8870 | IER3 | immediate early response 3 | 1.4848 | 0.007984 | 0.0805 | 11.3882 | 11.2169 | 11.3401 | 10.9837 | 11.7111 | 11.6464 | 10.4861 | 10.8639 | 10.1384 | 10.6074 | 10.7601 | 10.7157 |
| 202022_at | 230 | ALDOC | aldolase C, fructose-bisphosphate | 1.4846 | 0.001996 | 0.03683 | 4.9025 | 4.7827 | 4.7891 | 4.9025 | 4.8145 | 5.2415 | 4.2483 | 4.6432 | 4.337 | 4.5201 | 4.3685 | 4.4949 |
| 202683_s_at | 8731 | RNMT | RNA (guanine-7-) methyltransferase | 1.4812 | 0.001996 | 0.03683 | 8.3465 | 8.0363 | 8.1169 | 8.2196 | 8.0403 | 8.2527 | 7.2813 | 7.7998 | 7.7004 | 7.6047 | 7.3752 | 7.9299 |
| 218450_at | 50865 | HEBP1 | heme binding protein 1 | 1.4496 | 0.001996 | 0.03683 | 10.2689 | 10.1926 | 9.8804 | 9.9633 | 9.9764 | 9.8338 | 9.3727 | 9.2894 | 9.2928 | 9.0602 | 9.684 | 9.7119 |
| 202087_s_at | 1514 | CTSL1 | cathepsin L1 | 1.4438 | 0.005988 | 0.0805 | 13.7451 | 13.9002 | 13.7451 | 13.8177 | 13.9002 | 13.9002 | 13.3248 | 13.7451 | 13.4075 | 13.2128 | 13.3248 | 13.6034 |
| 202733_at | 8974 | P4HA2 | prolyl 4-hydroxylase, alpha polypeptide II | 1.3916 | 0.001996 | 0.03683 | 7.9848 | 9.0276 | 8.0829 | 8.1923 | 7.8894 | 7.8976 | 7.2291 | 7.4922 | 6.5715 | 7.2099 | 7.3089 | 6.9988 |
| 201700_at | 896 | CCND3 | cyclin D3 | 1.3718 | 0.001996 | 0.03683 | 9.2928 | 9.2225 | 9.2979 | 9.2559 | 9.2894 | 9.2559 | 9.1936 | 9.045 | 8.5475 | 8.8963 | 8.9323 | 8.9817 |
| 202555_s_at | 4638 | MYLK | myosin light chain kinase | 1.3696 | 0.01796 | 0.1156 | 7.5151 | 6.9657 | 7.4609 | 6.7148 | 7.5894 | 7.8309 | 6.718 | 6.6943 | 6.0689 | 6.4052 | 6.4004 | 6.3782 |
| 212281_s_at | 27346 | TMEM97 | transmembrane protein 97 | 1.3568 | 0.001996 | 0.03683 | 10.5704 | 10.2889 | 10.3818 | 10.1993 | 10.1666 | 10.2533 | 9.4001 | 9.9038 | 9.3223 | 9.6721 | 10.0849 | 9.8461 |
| 202756_s_at | 2817 | GPC1 | glypican 1 | 1.3464 | 0.007984 | 0.0805 | 7.437 | 7.3065 | 7.2921 | 7.4434 | 7.5461 | 7.5885 | 7.2722 | 6.9531 | 7.1323 | 7.1057 | 7.209 | 7.042 |
| 202246_s_at | 1019 | CDK4 | cyclin-dependent kinase 4 | 1.3078 | 0.001996 | 0.03683 | 12.1519 | 12.2657 | 12.2184 | 12.246 | 12.3315 | 12.2931 | 11.985 | 12.0869 | 11.5032 | 11.8741 | 12.0242 | 11.9142 |
| 202371_at | 79921 | TCEAL4 | transcription elongation factor A (SII)-like 4 | 1.3029 | 0.001996 | 0.03683 | 10.9477 | 10.9943 | 10.7843 | 11.1857 | 10.9278 | 10.9278 | 10.5353 | 10.8331 | 10.459 | 10.4861 | 10.6815 | 10.647 |
| 220091_at | 11182 | SLC2A6 | solute carrier family 2 (facilitated glucose transporter), member 6 | 1.2913 | 0.005988 | 0.0805 | 10.0527 | 9.8461 | 10.0565 | 9.8248 | 9.6882 | 9.5952 | 8.9839 | 8.3743 | 9.5221 | 8.9323 | 8.1893 | 9.4293 |
| 202784_s_at | 23530 | NNT | nicotinamide nucleotide transhydrogenase | 1.2831 | 0.001996 | 0.03683 | 7.3649 | 7.0159 | 6.9042 | 7.209 | 7.042 | 7.0908 | 6.7515 | 6.9458 | 6.3064 | 6.4262 | 6.63 | 6.5622 |
| 200807_s_at | 3329 | HSPD1 | heat shock 60kDa protein 1 (chaperonin) | 1.2643 | 0.005988 | 0.0805 | 10.7777 | 10.9582 | 10.8392 | 10.9182 | 11.0671 | 11.04 | 10.8479 | 10.6815 | 10.0808 | 10.1926 | 10.3719 | 10.365 |
| 204000_at | 10681 | GNB5 | guanine nucleotide binding protein (G protein), beta 5 | 1.264 | 0.001996 | 0.03683 | 6.887 | 7.2813 | 6.887 | 6.9731 | 7.5158 | 6.9731 | 6.3392 | 6.8533 | 6.2218 | 6.5498 | 6.0558 | 6.4548 |
| 203405_at | 8624 | PSMG1 | proteasome (prosome, macropain) assembly chaperone 1 | 1.2573 | 0.003992 | 0.06972 | 11.5656 | 11.669 | 11.8922 | 11.8527 | 11.6464 | 11.4384 | 11.1258 | 11.5736 | 11.0165 | 11.2899 | 11.1592 | 11.0282 |
| 218581_at | 63874 | ABHD4 | abhydrolase domain containing 4 | 1.2488 | 0.005988 | 0.0805 | 7.3419 | 7.5484 | 8.3257 | 6.988 | 6.8615 | 8.4785 | 6.6394 | 6.6264 | 6.1988 | 6.6943 | 6.3555 | 6.5208 |
| 221060_s_at | 7099 | TLR4 | toll-like receptor 4 | 1.2009 | 0.005988 | 0.0805 | 8.8432 | 8.7049 | 8.6032 | 8.4785 | 8.4038 | 8.6794 | 8.2102 | 8.1106 | 7.4434 | 7.8858 | 8.3045 | 8.2587 |
| 218034_at | 51024 | FIS1 | fission 1 (mitochondrial outer membrane) homolog (S. cerevisiae) | 1.1986 | 0.003992 | 0.06972 | 8.3545 | 8.2527 | 8.511 | 8.3545 | 8.5944 | 8.3263 | 8.2263 | 6.0689 | 6.4376 | 6.1558 | 8.4418 | 5.9556 |
| 201080_at | 8396 | PIP4K2B | phosphatidylinositol-5-phosphate 4-kinase, type II, beta | 1.1984 | 0.005988 | 0.0805 | 6.5793 | 6.7086 | 6.2451 | 6.3669 | 6.5715 | 6.4376 | 5.5688 | 6.1605 | 5.7865 | 5.4454 | 6.102 | 6.2451 |
| 202780_at | 5019 | OXCT1 | 3-oxoacid CoA transferase 1 | 1.1976 | 0.00998 | 0.08955 | 9.5971 | 9.9424 | 9.7678 | 9.9317 | 9.3768 | 9.684 | 9.2559 | 9.459 | 9.2787 | 9.3768 | 9.4293 | 9.3768 |
| 202500_at | 3300 | DNAJB2 | DnaJ (Hsp40) homolog, subfamily B, member 2 | 1.195 | 0.02395 | 0.1331 | 10.0145 | 8.7482 | 9.8902 | 8.4919 | 10.1251 | 9.7119 | 8.2527 | 8.124 | 8.6611 | 8.5534 | 8.5504 | 8.1223 |
| 212955_s_at | 5438 | POLR2I | polymerase (RNA) II (DNA directed) polypeptide I, 14.5kDa | 1.1912 | 0.01996 | 0.1243 | 8.7304 | 8.6465 | 8.6611 | 8.0155 | 8.907 | 8.5782 | 8.3257 | 7.706 | 8.0238 | 7.6669 | 8.0281 | 7.5703 |
| 40829_at | 23038 | WDTC1 | WD and tetratricopeptide repeats 1 | 1.1817 | 0.01996 | 0.1243 | 8.1495 | 8.5237 | 8.5534 | 8.5534 | 8.4914 | 8.6205 | 7.8985 | 8.4241 | 7.7616 | 7.8729 | 8.0444 | 8.0559 |
| 205376_at | 8821 | INPP4B | inositol polyphosphate-4-phosphatase, type II, 105kDa | 1.1684 | 0.001996 | 0.03683 | 6.3476 | 6.3361 | 6.2018 | 6.5887 | 6.4096 | 6.3383 | 6.0689 | 6.1169 | 5.8888 | 5.9772 | 6.224 | 6.1777 |
| 202180_s_at | 9961 | MVP | major vault protein | 1.1312 | 0.001996 | 0.03683 | 11.4145 | 11.7333 | 11.5382 | 11.3882 | 11.4384 | 11.8741 | 11.3254 | 11.247 | 10.7601 | 10.7777 | 11.0165 | 11.2365 |
| 202125_s_at | 66008 | TRAK2 | trafficking protein, kinesin binding 2 | 1.1073 | 0.00998 | 0.08955 | 7.5831 | 7.2099 | 6.8804 | 6.5498 | 6.5887 | 7.8751 | 6.2743 | 6.4322 | 5.9994 | 6.2451 | 6.2743 | 6.5793 |
| 201186_at | 4043 | LRPAP1 | low density lipoprotein receptor-related protein associated protein 1 | 1.1043 | 0.001996 | 0.03683 | 8.6545 | 8.8163 | 8.8039 | 8.941 | 8.7387 | 8.8761 | 8.609 | 8.27 | 8.2527 | 7.3551 | 7.8224 | 8.6173 |
| 200078_s_at | 533 | ATP6V0B | ATPase, H+ transporting, lysosomal 21kDa, V0 subunit b | 1.1034 | 0.02196 | 0.1294 | 7.3385 | 7.5831 | 7.3707 | 7.1996 | 7.5011 | 7.4236 | 6.8095 | 6.9763 | 6.6032 | 7.088 | 7.3956 | 6.8753 |
| 219082_at | 51005 | AMDHD2 | amidohydrolase domain containing 2 | 1.1008 | 0.01198 | 0.09843 | 7.356 | 7.437 | 7.1596 | 7.5348 | 7.6511 | 7.7268 | 7.197 | 6.8435 | 7.0126 | 7.0936 | 7.0974 | 7.2722 |
| 204812_at | 9183 | ZW10 | ZW10, kinetochore associated, homolog (Drosophila) | 1.0975 | 0.03393 | 0.1558 | 10.1959 | 9.4293 | 10.1849 | 9.9764 | 9.9633 | 8.9476 | 8.7027 | 8.6398 | 8.2368 | 8.5629 | 9.6384 | 8.4749 |
| 222125_s_at | 54681 | P4HTM | prolyl 4-hydroxylase, transmembrane (endoplasmic reticulum) | 1.0819 | 0.01597 | 0.11 | 9.2695 | 7.6505 | 7.2999 | 7.6047 | 9.0518 | 8.9293 | 6.887 | 7.3174 | 6.5559 | 6.8018 | 7.1221 | 7.3828 |
| 202716_at | 5770 | PTPN1 | protein tyrosine phosphatase, non-receptor type 1 | 1.0763 | 0.01597 | 0.11 | 9.684 | 9.9038 | 10.1028 | 9.8902 | 9.9918 | 9.9764 | 9.6206 | 9.1448 | 9.7401 | 9.6102 | 9.6523 | 9.5193 |
| 207620_s_at | 8573 | CASK | calcium/calmodulin-dependent serine protein kinase (MAGUK family) | 1.0702 | 0.01796 | 0.1156 | 7.5322 | 7.3752 | 6.8247 | 6.9125 | 7.1996 | 7.3678 | 6.3957 | 7.0936 | 6.4052 | 6.5559 | 6.6943 | 6.618 |
| 202117_at | 392 | ARHGAP1 | Rho GTPase activating protein 1 | 1.0697 | 0.00998 | 0.08955 | 9.4562 | 9.6788 | 9.6671 | 9.6634 | 9.7119 | 9.5676 | 9.1348 | 9.6105 | 9.2695 | 9.3848 | 9.2894 | 9.4116 |
| 201125_s_at | 3693 | ITGB5 | integrin, beta 5 | 1.0646 | 0.001996 | 0.03683 | 11.4922 | 11.7005 | 11.7277 | 11.4259 | 11.4675 | 11.5185 | 10.9582 | 11.2169 | 10.8176 | 11.1208 | 11.451 | 11.3906 |
| 212740_at | 30849 | PIK3R4 | phosphoinositide-3-kinase, regulatory subunit 4 | 1.0614 | 0.01397 | 0.1059 | 6.9988 | 7.1323 | 6.8095 | 7.075 | 7.0691 | 7.1782 | 6.7449 | 6.8533 | 6.3496 | 6.5208 | 6.8018 | 6.8515 |
| 217777_s_at | 51495 | PTPLAD1 | protein tyrosine phosphatase-like A domain containing 1 | 1.0497 | 0.02595 | 0.141 | 10.3917 | 10.633 | 10.5568 | 10.232 | 10.653 | 10.5491 | 10.2689 | 10.3354 | 10.1028 | 10.3293 | 10.1993 | 10.1099 |
| 201266_at | 7296 | TXNRD1 | thioredoxin reductase 1 | 1.0476 | 0.01397 | 0.1059 | 13.2128 | 13.2128 | 13.1644 | 13.3248 | 13.1644 | 13.2745 | 12.4808 | 13.3248 | 12.5073 | 12.8415 | 12.983 | 12.8415 |
| 202284_s_at | 1026 | CDKN1A | cyclin-dependent kinase inhibitor 1A (p21, Cip1) | 1.0377 | 0.001996 | 0.03683 | 9.6102 | 10.0808 | 9.6721 | 9.6206 | 9.5676 | 9.8248 | 9.4116 | 9.6384 | 9.0769 | 9.4001 | 9.0769 | 9.1423 |
| 212300_at | 200081 | TXLNA | taxilin alpha | 1.0164 | 0.007984 | 0.0805 | 11.7544 | 11.7993 | 11.7277 | 11.6252 | 11.8922 | 11.7715 | 11.2558 | 11.5857 | 11.0778 | 11.4259 | 11.7333 | 11.5185 |
| 202310_s_at | 1277 | COL1A1 | collagen, type I, alpha 1 | 1.009 | 0.01796 | 0.1156 | 12.7892 | 13.0802 | 12.7014 | 12.983 | 13.3248 | 13.2128 | 12.7892 | 12.7437 | 12.3115 | 12.5912 | 12.5394 | 12.6305 |
| 201078_at | 9375 | TM9SF2 | transmembrane 9 superfamily member 2 | 1.0009 | 0.02196 | 0.1294 | 8.5534 | 8.3092 | 8.5475 | 8.1992 | 8.6715 | 8.3545 | 7.7268 | 8.5264 | 7.5876 | 7.9258 | 7.994 | 7.3114 |
| 208711_s_at | 595 | CCND1 | cyclin D1 | 0.9957 | 0.02196 | 0.1294 | 5.4729 | 5.4036 | 5.1407 | 5.231 | 5.3633 | 5.4828 | 4.9889 | 4.7763 | 4.337 | 4.841 | 5.2953 | 5.0861 |
| 201453_x_at | 6009 | RHEB | Ras homolog enriched in brain | 0.9929 | 0.001996 | 0.03683 | 13.4469 | 13.4075 | 13.4469 | 13.4469 | 13.4075 | 13.6591 | 12.9116 | 13.2128 | 12.8646 | 13.4075 | 13.2745 | 13.2745 |
| 202761_s_at | 23224 | SYNE2 | spectrin repeat containing, nuclear envelope 2 | 0.9896 | 0.00998 | 0.08955 | 6.6264 | 6.6561 | 6.5918 | 6.6478 | 7.1241 | 6.2018 | 6.3669 | 6.3957 | 6.108 | 6.0903 | 5.9503 | 6.1777 |
| 208025_s_at | 8091 | HMGA2 | high mobility group AT-hook 2 | 0.9856 | 0.02395 | 0.1331 | 9.5221 | 9.4948 | 9.3686 | 9.6671 | 9.4881 | 9.4825 | 8.8432 | 9.631 | 9.0651 | 9.0957 | 9.24 | 8.907 |
| 204143_s_at | 55556 | ENOSF1 | enolase superfamily member 1 | 0.9729 | 0.01198 | 0.09843 | 5.2783 | 5.0402 | 5.5788 | 5.231 | 5.3313 | 5.3313 | 4.5201 | 5.3097 | 4.7256 | 4.6365 | 4.9592 | 4.9592 |
| 200617_at | 9761 | MLEC | malectin | 0.9717 | 0.02794 | 0.1457 | 11.9552 | 11.7111 | 12.015 | 11.9142 | 11.669 | 11.985 | 11.1393 | 11.9142 | 10.5295 | 10.737 | 11.4922 | 11.5278 |
| 200757_s_at | 813 | CALU | calumenin | 0.9663 | 0.02395 | 0.1331 | 12.1907 | 12.4808 | 12.3315 | 12.3655 | 12.1907 | 12.246 | 11.7544 | 12.3315 | 11.8824 | 11.985 | 12.0358 | 12.0358 |
| 202458_at | 11098 | PRSS23 | protease, serine, 23 | 0.9648 | 0.007984 | 0.0805 | 11.8922 | 11.8527 | 11.9303 | 12.015 | 11.8403 | 11.7993 | 11.5656 | 11.8403 | 11.3401 | 11.6252 | 11.7544 | 11.7333 |
| 201628_s_at | 10670 | RRAGA | Ras-related GTP binding A | 0.9644 | 0.01397 | 0.1059 | 9.3933 | 9.3589 | 9.1834 | 9.3502 | 9.3223 | 9.1348 | 8.9954 | 9.103 | 8.5346 | 8.9817 | 9.1505 | 9.0717 |
| 202675_at | 6390 | SDHB | succinate dehydrogenase complex, subunit B, iron sulfur (Ip) | 0.9633 | 0.00998 | 0.08955 | 12.5394 | 12.983 | 12.5912 | 12.8646 | 12.4521 | 12.6305 | 12.0242 | 12.4808 | 11.9552 | 12.417 | 12.4808 | 12.1519 |
| 217871_s_at | 4282 | MIF | macrophage migration inhibitory factor (glycosylation-inhibiting factor) | 0.9631 | 0.01597 | 0.11 | 13.962 | 14.1383 | 14.4357 | 14.2088 | 14.4357 | 14.4357 | 13.9002 | 14.1383 | 13.6034 | 13.9002 | 13.9002 | 14.0045 |
| 218847_at | 10644 | IGF2BP2 | insulin-like growth factor 2 mRNA binding protein 2 | 0.9622 | 0.01198 | 0.09843 | 8.3092 | 8.0884 | 7.8447 | 8.0487 | 8.2845 | 8.1545 | 7.863 | 8.009 | 7.168 | 7.6947 | 7.7268 | 7.632 |
| 212330_at | 7027 | TFDP1 | transcription factor Dp-1 | 0.9619 | 0.02994 | 0.1457 | 9.3223 | 9.8804 | 9.8644 | 9.5385 | 10.1486 | 9.9746 | 8.6631 | 9.0729 | 9.0922 | 9.459 | 9.6721 | 8.8963 |
| 203648_at | 9797 | TATDN2 | TatD DNase domain containing 2 | 0.9583 | 0.007984 | 0.0805 | 5.5822 | 5.663 | 5.5235 | 5.6525 | 5.8199 | 5.6828 | 4.9736 | 5.614 | 5.2031 | 5.1978 | 5.4828 | 5.4894 |
| 209213_at | 873 | CBR1 | carbonyl reductase 1 | 0.9392 | 0.04391 | 0.1812 | 9.9167 | 9.9746 | 9.804 | 9.9746 | 9.9902 | 9.8854 | 9.3333 | 9.6037 | 9.4224 | 9.4187 | 9.936 | 9.9273 |
| 220140_s_at | 29916 | SNX11 | sorting nexin 11 | 0.9319 | 0.01796 | 0.1156 | 6.1843 | 6.5405 | 5.7667 | 6.5104 | 6.1479 | 5.8163 | 5.9994 | 5.6421 | 5.2783 | 5.5738 | 5.7482 | 5.5542 |
| 208624_s_at | 1981 | EIF4G1 | eukaryotic translation initiation factor 4 gamma, 1 | 0.9218 | 0.01597 | 0.11 | 7.7356 | 7.8729 | 7.4643 | 7.4743 | 7.937 | 7.6219 | 7.3306 | 7.4922 | 6.8533 | 6.9657 | 7.5703 | 7.2156 |
| 213851_at | 375346 | TMEM110 | transmembrane protein 110 | 0.9182 | 0.04192 | 0.1767 | 6.7852 | 6.5842 | 7.028 | 7.042 | 6.9531 | 7.1423 | 6.5208 | 6.618 | 6.5426 | 6.8018 | 6.729 | 6.5948 |
| 212399_s_at | 9686 | VGLL4 | vestigial like 4 (Drosophila) | 0.9142 | 0.02196 | 0.1294 | 8.3629 | 8.1602 | 8.0653 | 8.1665 | 7.9031 | 8.1992 | 7.9171 | 7.7756 | 8.0444 | 7.7838 | 8.0238 | 7.7129 |
| 200981_x_at | 2778 | GNAS | GNAS complex locus | 0.9021 | 0.03194 | 0.1524 | 13.1644 | 13.2745 | 13.0802 | 13.2128 | 12.7892 | 12.8646 | 12.6305 | 13.0242 | 12.2931 | 12.5394 | 12.7664 | 12.7437 |
| 201709_s_at | 8508 | NIPSNAP1 | nipsnap homolog 1 (C. elegans) | 0.9008 | 0.05788 | 0.2089 | 8.6279 | 8.6367 | 8.609 | 8.5757 | 8.2999 | 8.7071 | 8.1893 | 8.3545 | 8.2903 | 8.0684 | 8.4056 | 8.5177 |
| 208050_s_at | 835 | CASP2 | caspase 2, apoptosis-related cysteine peptidase | 0.8996 | 0.01397 | 0.1059 | 5.922 | 6.4857 | 6.0979 | 6.2743 | 6.0868 | 6.102 | 6.0363 | 5.8835 | 5.6165 | 5.6058 | 6.0115 | 5.4347 |
| 202109_at | 23647 | ARFIP2 | ADP-ribosylation factor interacting protein 2 | 0.8979 | 0.02994 | 0.1457 | 10.1435 | 10.0766 | 9.8248 | 9.9317 | 9.8338 | 10.1099 | 9.4687 | 9.7678 | 9.3953 | 9.4319 | 9.2979 | 10.1251 |
| 204285_s_at | 5366 | PMAIP1 | phorbol-12-myristate-13-acetate-induced protein 1 | 0.894 | 0.02994 | 0.1457 | 9.631 | 9.9633 | 9.8338 | 9.4631 | 10.0183 | 10.0226 | 9.18 | 9.4319 | 8.9476 | 9.5676 | 9.6968 | 9.4224 |
| 40562_at | 2767 | GNA11 | guanine nucleotide binding protein (G protein), alpha 11 (Gq class) | 0.8752 | 0.005988 | 0.0805 | 9.0276 | 9.3202 | 9.2487 | 8.8986 | 9.3037 | 9.045 | 8.6336 | 8.7838 | 8.4241 | 7.156 | 8.402 | 9.0602 |
| 202825_at | 291 | SLC25A4 | solute carrier family 25 (mitochondrial carrier; adenine nucleotide translocator), member 4 | 0.8717 | 0.02395 | 0.1331 | 9.7268 | 9.5385 | 9.5703 | 9.5013 | 9.6206 | 9.3768 | 9.1881 | 9.5013 | 9.1881 | 9.24 | 9.1559 | 9.5434 |
| 202540_s_at | 3156 | HMGCR | 3-hydroxy-3-methylglutaryl-CoA reductase | 0.8678 | 0.02794 | 0.1457 | 8.9476 | 8.9937 | 7.852 | 9.0552 | 9.0083 | 8.4014 | 7.6486 | 8.1404 | 8.4749 | 8.0939 | 7.479 | 8.0238 |
| 202911_at | 2956 | MSH6 | mutS homolog 6 (E. coli) | 0.8557 | 0.01597 | 0.11 | 9.6721 | 9.8854 | 10.0808 | 10.435 | 10.0323 | 9.5035 | 9.1707 | 9.6671 | 9.4462 | 9.3602 | 9.4001 | 9.7678 |
| 201555_at | 4172 | MCM3 | minichromosome maintenance complex component 3 | 0.8547 | 0.01597 | 0.11 | 11.0778 | 11.1746 | 10.9278 | 11.2899 | 10.8361 | 10.908 | 10.8911 | 10.8826 | 10.5568 | 10.4155 | 10.737 | 10.8361 |
| 217478_s_at | 3108 | HLA-DMA | major histocompatibility complex, class II, DM alpha | 0.844 | 0.01796 | 0.1156 | 5.7377 | 6.1811 | 5.844 | 6.0689 | 6.0054 | 5.9889 | 5.5304 | 5.8494 | 5.5856 | 5.0492 | 5.5606 | 5.9059 |
| 209409_at | 2887 | GRB10 | growth factor receptor-bound protein 10 | 0.8435 | 0.02395 | 0.1331 | 7.0198 | 7.075 | 6.8515 | 7.1638 | 7.1423 | 6.7738 | 6.6628 | 6.7738 | 6.4376 | 6.7764 | 6.887 | 6.855 |
| 203367_at | 11072 | DUSP14 | dual specificity phosphatase 14 | 0.8431 | 0.01597 | 0.11 | 8.2444 | 8.1415 | 7.9809 | 7.994 | 8.0069 | 8.2553 | 7.8729 | 7.9217 | 7.4967 | 7.8136 | 8.0155 | 7.937 |
| 201899_s_at | 7319 | UBE2A | ubiquitin-conjugating enzyme E2A | 0.8241 | 0.0479 | 0.1889 | 12.1784 | 12.3115 | 12.2184 | 12.3115 | 12.1784 | 12.0613 | 11.9552 | 12.2931 | 11.7333 | 11.9552 | 11.985 | 11.985 |
| 208717_at | 5018 | OXA1L | oxidase (cytochrome c) assembly 1-like | 0.8239 | 0.05788 | 0.2089 | 12.246 | 12.1784 | 12.1267 | 12.0613 | 12.0242 | 12.1784 | 11.7221 | 12.1519 | 12.1105 | 11.6213 | 11.9142 | 11.8527 |
| 200972_at | 10099 | TSPAN3 | tetraspanin 3 | 0.8161 | 0.05788 | 0.2089 | 10.1251 | 10.1154 | 9.9558 | 10.1384 | 9.8994 | 10.212 | 9.4293 | 10.1514 | 9.7825 | 9.8248 | 9.7401 | 9.804 |
| 201795_at | 3930 | LBR | lamin B receptor | 0.8152 | 0.007984 | 0.0805 | 9.1048 | 9.153 | 9.0769 | 9.045 | 9.153 | 8.9817 | 9.0242 | 8.9532 | 8.5144 | 9.0099 | 8.904 | 8.8628 |
| 209588_at | 2048 | EPHB2 | EPH receptor B2 | 0.815 | 0.0499 | 0.1937 | 7.2468 | 7.1782 | 7.2378 | 7.2813 | 7.028 | 6.855 | 6.9531 | 7.1373 | 6.6336 | 6.855 | 6.8515 | 6.7367 |
| 209166_s_at | 4125 | MAN2B1 | mannosidase, alpha, class 2B, member 1 | 0.815 | 0.03792 | 0.1656 | 6.1674 | 6.4548 | 6.0338 | 6.3152 | 6.1169 | 6.3826 | 5.9438 | 5.8494 | 6.1605 | 5.922 | 6.1582 | 6.0054 |
| 200791_s_at | 8826 | IQGAP1 | IQ motif containing GTPase activating protein 1 | 0.8064 | 0.02595 | 0.141 | 9.7072 | 9.5193 | 9.4293 | 9.7541 | 9.5971 | 9.5336 | 9.3465 | 9.3465 | 9.5801 | 9.0242 | 9.3953 | 9.3848 |
| 218910_at | 55129 | ANO10 | anoctamin 10 | 0.7924 | 0.05988 | 0.213 | 7.4155 | 7.3439 | 7.3174 | 7.5894 | 7.1241 | 7.5658 | 7.156 | 7.5375 | 6.8106 | 6.9308 | 6.8753 | 6.9458 |
| 220094_s_at | 63933 | CCDC90A | coiled-coil domain containing 90A | 0.7913 | 0.001996 | 0.03683 | 7.9915 | 7.95 | 7.9728 | 8.1034 | 7.9457 | 7.9728 | 6.3476 | 7.8773 | 6.7706 | 7.7681 | 7.868 | 7.829 |
| 37152_at | 5467 | PPARD | peroxisome proliferator-activated receptor delta | 0.7908 | 0.02994 | 0.1457 | 6.618 | 6.4795 | 6.3555 | 6.7353 | 6.7698 | 6.6943 | 5.9854 | 6.3867 | 6.5498 | 5.9924 | 6.4611 | 6.3631 |
| 204297_at | 5289 | PIK3C3 | phosphoinositide-3-kinase, class 3 | 0.789 | 0.02196 | 0.1294 | 8.5757 | 8.6173 | 8.4824 | 8.8501 | 8.6189 | 8.7006 | 8.3407 | 8.5177 | 8.2824 | 8.4824 | 8.609 | 8.4583 |
| 202732_at | 11142 | PKIG | protein kinase (cAMP-dependent, catalytic) inhibitor gamma | 0.7885 | 0.06188 | 0.2169 | 10.8331 | 10.6708 | 10.6859 | 10.448 | 10.6105 | 10.7417 | 10.3066 | 10.5669 | 9.8902 | 10.2689 | 10.212 | 10.737 |
| 215093_at | 50814 | NSDHL | NAD(P) dependent steroid dehydrogenase-like | 0.7869 | 0.03792 | 0.1656 | 7.7844 | 9.804 | 9.6882 | 9.4948 | 9.5509 | 9.7963 | 8.5681 | 8.6205 | 8.3436 | 7.568 | 8.5177 | 8.7838 |
| 212462_at | 23522 | KAT6B | K(lysine) acetyltransferase 6B | 0.7767 | 0.04192 | 0.1767 | 6.7023 | 6.5529 | 6.3205 | 6.3933 | 6.5462 | 6.4485 | 6.4201 | 6.256 | 6.1743 | 6.3178 | 6.443 | 6.1605 |
| 202179_at | 642 | BLMH | bleomycin hydrolase | 0.776 | 0.02994 | 0.1457 | 7.5158 | 7.4625 | 7.3551 | 7.7356 | 7.513 | 7.3114 | 7.1751 | 7.3914 | 7.0955 | 6.9531 | 7.2869 | 7.437 |
| 203775_at | 10165 | SLC25A13 | solute carrier family 25, member 13 (citrin) | 0.7733 | 0.03792 | 0.1656 | 9.1448 | 9.0755 | 8.9854 | 9.3347 | 8.941 | 8.7665 | 8.7304 | 8.5757 | 8.4824 | 8.9824 | 8.9937 | 8.5475 |
| 209161_at | 9128 | PRPF4 | PRP4 pre-mRNA processing factor 4 homolog (yeast) | 0.7713 | 0.02794 | 0.1457 | 9.1545 | 8.8963 | 8.8761 | 9.0374 | 8.8705 | 9.1559 | 8.8039 | 8.901 | 8.3092 | 8.7161 | 8.9134 | 8.6896 |
| 207808_s_at | 5627 | PROS1 | protein S (alpha) | 0.7691 | 0.03992 | 0.1705 | 6.3631 | 6.988 | 6.492 | 7.0126 | 6.9896 | 7.0908 | 6.1811 | 6.718 | 6.2218 | 6.3593 | 5.9184 | 6.6796 |
| 204475_at | 4312 | MMP1 | matrix metallopeptidase 1 (interstitial collagenase) | 0.7615 | 0.03992 | 0.1705 | 14.2088 | 13.8177 | 13.9002 | 14.1383 | 13.8177 | 14.1383 | 13.4469 | 13.962 | 13.8177 | 13.8177 | 13.7451 | 13.4469 |
| 218375_at | 53343 | NUDT9 | nudix (nucleoside diphosphate linked moiety X)-type motif 9 | 0.7607 | 0.0519 | 0.1967 | 8.9527 | 9.0242 | 8.5986 | 8.3883 | 8.7727 | 8.8371 | 8.5534 | 8.587 | 8.3436 | 8.4647 | 8.6336 | 8.402 |
| 207847_s_at | 4582 | MUC1 | mucin 1, cell surface associated | 0.7576 | 0.02994 | 0.1457 | 4.4625 | 4.8366 | 4.6966 | 4.3527 | 4.6498 | 4.5382 | 4.3843 | 4.4285 | 4.1776 | 4.3527 | 4.3213 | 4.5382 |
| 202812_at | 2548 | GAA | glucosidase, alpha; acid | 0.7574 | 0.04192 | 0.1767 | 4.4285 | 4.9409 | 4.6203 | 4.4949 | 4.231 | 4.3213 | 3.6664 | 3.9934 | 3.7012 | 3.8013 | 4.0394 | 4.7929 |
| 202260_s_at | 6812 | STXBP1 | syntaxin binding protein 1 | 0.7548 | 0.03194 | 0.1524 | 5.5478 | 6.5685 | 6.5208 | 6.6647 | 6.3361 | 5.9503 | 5.4828 | 6.0728 | 5.2462 | 5.3633 | 5.8629 | 6.0054 |
| 201040_at | 2771 | GNAI2 | guanine nucleotide binding protein (G protein), alpha inhibiting activity polypeptide 2 | 0.7501 | 0.03393 | 0.1558 | 7.4967 | 8.1665 | 7.7599 | 7.6219 | 8.0069 | 7.5885 | 7.5418 | 7.3828 | 6.9731 | 7.168 | 7.6947 | 7.5073 |
| 201572_x_at | 1635 | DCTD | dCMP deaminase | 0.7411 | 0.03792 | 0.1656 | 10.1514 | 10.1384 | 9.9273 | 10.0766 | 10.0527 | 10.1993 | 9.7929 | 10.0145 | 9.4293 | 10.042 | 9.9918 | 9.8248 |
| 206015_s_at | 22887 | FOXJ3 | forkhead box J3 | 0.7374 | 0.03194 | 0.1524 | 9.6788 | 9.5676 | 9.2709 | 9.3768 | 9.4948 | 9.6102 | 7.0456 | 9.5336 | 8.7151 | 9.2225 | 7.852 | 9.5062 |
| 202550_s_at | 9217 | VAPB | VAMP (vesicle-associated membrane protein)-associated protein B and C | 0.7366 | 0.06786 | 0.2329 | 7.1885 | 7.0813 | 7.2043 | 7.1373 | 7.3802 | 7.0355 | 6.8717 | 6.794 | 6.7353 | 6.8804 | 7.3028 | 7.0095 |
| 202880_s_at | 9267 | CYTH1 | cytohesin 1 | 0.7361 | 0.04391 | 0.1812 | 4.1776 | 4.8366 | 4.2967 | 4.2967 | 4.6041 | 4.4625 | 4.3527 | 4.1776 | 4.1498 | 4.2043 | 4.231 | 4.1498 |
| 218050_at | 51569 | UFM1 | ubiquitin-fold modifier 1 | 0.7332 | 0.09581 | 0.2901 | 8.1602 | 8.5064 | 8.3872 | 8.2999 | 8.4038 | 8.2903 | 7.0631 | 8.3465 | 7.2291 | 6.9458 | 8.4443 | 8.3407 |
| 201811_x_at | 9467 | SH3BP5 | SH3-domain binding protein 5 (BTK-associated) | 0.7301 | 0.09182 | 0.2824 | 9.1936 | 8.5807 | 9.0325 | 8.9134 | 8.9674 | 8.9937 | 8.4418 | 8.6578 | 8.6505 | 8.5091 | 9.0374 | 8.5807 |
| 207196_s_at | 10318 | TNIP1 | TNFAIP3 interacting protein 1 | 0.7291 | 0.0499 | 0.1937 | 7.1596 | 6.3361 | 7.0936 | 6.3593 | 6.3739 | 6.3064 | 5.7815 | 6.1358 | 5.5688 | 5.9503 | 6.7706 | 5.8083 |
| 217956_s_at | 58478 | ENOPH1 | enolase-phosphatase 1 | 0.7289 | 0.03393 | 0.1558 | 11.04 | 11.2734 | 10.9582 | 11.2124 | 11.0534 | 11.2726 | 10.7843 | 11.0165 | 10.5704 | 10.7988 | 11.0563 | 11.0993 |
| 200698_at | 11014 | KDELR2 | KDEL (Lys-Asp-Glu-Leu) endoplasmic reticulum protein retention receptor 2 | 0.7272 | 0.0479 | 0.1889 | 8.2587 | 7.989 | 7.937 | 8.0684 | 8.0281 | 8.1327 | 7.5894 | 8.0884 | 7.0661 | 7.4474 | 7.8447 | 8.1122 |
| 205504_at | 695 | BTK | Bruton agammaglobulinemia tyrosine kinase | 0.7269 | 0.0499 | 0.1937 | 3.924 | 4.1637 | 3.7012 | 3.2386 | 3.8792 | 3.5043 | 3.5043 | 3.4175 | 3.2386 | 3.2386 | 3.5043 | 3.5043 |
| 208763_s_at | 1831 | TSC22D3 | TSC22 domain family, member 3 | 0.7264 | 0.03992 | 0.1705 | 6.8106 | 6.8292 | 6.7764 | 7.1423 | 6.618 | 6.5065 | 6.492 | 6.5208 | 6.7164 | 6.0639 | 6.4292 | 6.5622 |
| 218316_at | 26520 | TIMM9 | translocase of inner mitochondrial membrane 9 homolog (yeast) | 0.7248 | 0.05988 | 0.213 | 12.983 | 13.0242 | 13.2128 | 13.0242 | 13.0802 | 12.983 | 12.4521 | 13.2745 | 12.3315 | 12.7014 | 12.5912 | 13.0242 |
| 217867_x_at | 25825 | BACE2 | beta-site APP-cleaving enzyme 2 | 0.7216 | 0.05788 | 0.2089 | 8.0653 | 8.1923 | 8.7598 | 8.1754 | 7.9869 | 8.8963 | 8.3743 | 7.4155 | 7.5461 | 7.5507 | 8.2366 | 7.4434 |
| 207163_s_at | 207 | AKT1 | v-akt murine thymoma viral oncogene homolog 1 | 0.7074 | 0.04391 | 0.1812 | 9.0485 | 9.18 | 9.0325 | 9.2384 | 9.3361 | 9.103 | 8.9175 | 9.1448 | 8.8181 | 8.8085 | 9.0242 | 9.1101 |
| 202809_s_at | 65123 | INTS3 | integrator complex subunit 3 | 0.7004 | 0.03393 | 0.1558 | 7.6653 | 7.7942 | 7.816 | 7.706 | 7.9869 | 7.706 | 7.798 | 7.5894 | 7.1801 | 7.6669 | 7.2722 | 7.6511 |
| 210337_s_at | 47 | ACLY | ATP citrate lyase | 0.6967 | 0.005988 | 0.0805 | 10.7601 | 14.8479 | 10.5073 | 10.5209 | 10.5209 | 10.7777 | 9.9558 | 10.2247 | 9.7541 | 9.6671 | 10.1126 | 10.0766 |
| 212644_s_at | 93487 | MAPK1IP1L | mitogen-activated protein kinase 1 interacting protein 1-like | 0.6904 | 0.06188 | 0.2169 | 8.2196 | 8.1893 | 8.3743 | 8.4362 | 9.3635 | 9.6206 | 7.7004 | 8.1404 | 8.587 | 8.1415 | 7.9479 | 7.7522 |
| 200666_s_at | 3337 | DNAJB1 | DnaJ (Hsp40) homolog, subfamily B, member 1 | 0.6727 | 0.07585 | 0.2506 | 9.8499 | 9.618 | 9.7541 | 10.0183 | 9.8644 | 9.9918 | 9.3614 | 9.809 | 9.415 | 9.4724 | 9.8248 | 9.7541 |
| 201121_s_at | 10857 | PGRMC1 | progesterone receptor membrane component 1 | 0.6718 | 0.05389 | 0.2012 | 12.8646 | 12.5651 | 13.0242 | 12.5912 | 12.9116 | 12.3797 | 12.5394 | 12.7014 | 11.9303 | 12.1105 | 12.2931 | 12.5651 |
| 209615_s_at | 5058 | PAK1 | p21 protein (Cdc42/Rac)-activated kinase 1 | 0.6696 | 0.0479 | 0.1889 | 6.8421 | 6.9531 | 6.9495 | 7.1084 | 6.9531 | 6.9657 | 6.9387 | 6.8095 | 6.4201 | 6.8428 | 6.9258 | 6.7227 |
| 221539_at | 1978 | EIF4EBP1 | eukaryotic translation initiation factor 4E binding protein 1 | 0.6686 | 0.07784 | 0.2546 | 6.0937 | 7.7634 | 7.4643 | 7.654 | 8.1393 | 6.9009 | 5.7914 | 5.9994 | 5.6525 | 7.267 | 5.6765 | 7.4155 |
| 208424_s_at | 57019 | CIAPIN1 | cytokine induced apoptosis inhibitor 1 | 0.6632 | 0.06387 | 0.2207 | 9.6882 | 9.5971 | 10.2689 | 9.6523 | 9.4611 | 9.4001 | 9.2444 | 9.2787 | 9.1722 | 9.6105 | 9.5548 | 9.24 |
| 201847_at | 3988 | LIPA | lipase A, lysosomal acid, cholesterol esterase | 0.6622 | 0.06387 | 0.2207 | 11.5857 | 11.3401 | 11.2514 | 10.879 | 10.737 | 11.3906 | 10.468 | 11.0282 | 10.8826 | 10.6708 | 10.9182 | 11.04 |
| 202651_at | 9926 | LPGAT1 | lysophosphatidylglycerol acyltransferase 1 | 0.661 | 0.0519 | 0.1967 | 9.18 | 8.9476 | 9.045 | 9.1181 | 9.3465 | 9.5035 | 8.8266 | 9.1707 | 8.6205 | 8.7568 | 8.941 | 9.148 |
| 201393_s_at | 3482 | IGF2R | insulin-like growth factor 2 receptor | 0.6607 | 0.05788 | 0.2089 | 10.6859 | 10.3006 | 10.4304 | 10.3671 | 10.4155 | 10.4396 | 10.3293 | 10.653 | 9.9764 | 9.9633 | 10.1486 | 9.936 |
| 209408_at | 11004 | KIF2C | kinesin family member 2C | 0.66 | 0.09381 | 0.2858 | 4.3843 | 4.9592 | 4.6203 | 4.5854 | 4.9664 | 5.1552 | 4.0809 | 4.741 | 3.8792 | 4.0394 | 4.6432 | 4.6365 |
| 202862_at | 2184 | FAH | fumarylacetoacetate hydrolase (fumarylacetoacetase) | 0.6595 | 0.06986 | 0.2389 | 8.2903 | 8.4221 | 8.3465 | 8.4075 | 8.6827 | 8.5389 | 8.2903 | 8.511 | 8.1256 | 8.347 | 8.1122 | 7.9457 |
| 221531_at | 80349 | WDR61 | WD repeat domain 61 | 0.6529 | 0.04591 | 0.1871 | 8.0487 | 9.2264 | 8.0653 | 8.5629 | 8.2519 | 8.6414 | 8.2102 | 7.9604 | 7.7458 | 8.27 | 8.1782 | 7.8184 |
| 204695_at | 993 | CDC25A | cell division cycle 25 homolog A (S. pombe) | 0.6526 | 0.0998 | 0.3003 | 8.3903 | 8.8131 | 8.8416 | 9.1048 | 8.6279 | 8.8416 | 8.6398 | 8.8163 | 7.8894 | 7.7998 | 8.714 | 8.0306 |
| 201460_at | 9261 | MAPKAPK2 | mitogen-activated protein kinase-activated protein kinase 2 | 0.6521 | 0.01397 | 0.1059 | 9.9038 | 10.1251 | 9.9764 | 12.6305 | 10.2864 | 10.0145 | 9.6634 | 9.5801 | 8.9824 | 9.2695 | 9.7859 | 10.042 |
| 213720_s_at | 6597 | SMARCA4 | SWI/SNF related, matrix associated, actin dependent regulator of chromatin, subfamily a, member 4 | 0.6499 | 0.07186 | 0.2423 | 7.1241 | 6.3908 | 6.6032 | 6.8203 | 7.4055 | 7.1996 | 6.3339 | 6.6115 | 6.2218 | 6.2681 | 6.8435 | 6.7353 |
| 205451_at | 4303 | FOXO4 | forkhead box O4 | 0.6459 | 0.08982 | 0.2771 | 6.0689 | 5.8835 | 5.9994 | 6.2589 | 6.1874 | 5.922 | 5.4961 | 6.3438 | 5.5187 | 5.1697 | 5.4454 | 6.0689 |
| 206070_s_at | 2042 | EPHA3 | EPH receptor A3 | 0.6396 | 0.05389 | 0.2012 | 6.6679 | 6.2618 | 7.7268 | 6.5948 | 8.3263 | 8.0444 | 6.3108 | 6.3739 | 5.844 | 6.3704 | 6.04 | 7.3551 |
| 202963_at | 5993 | RFX5 | regulatory factor X, 5 (influences HLA class II expression) | 0.6354 | 0.05788 | 0.2089 | 7.6754 | 7.8257 | 6.7203 | 8.0259 | 6.794 | 6.7449 | 6.4322 | 6.4485 | 6.1479 | 7.1373 | 6.3361 | 7.2434 |
| 212591_at | 23029 | RBM34 | RNA binding motif protein 34 | 0.6288 | 0.1916 | 0.4389 | 11.2365 | 11.3882 | 11.2734 | 11.1258 | 11.3122 | 11.2726 | 10.972 | 11.2365 | 10.7843 | 11.2734 | 11.2899 | 10.908 |
| 208634_s_at | 23499 | MACF1 | microtubule-actin crosslinking factor 1 | 0.6268 | 0.005988 | 0.0805 | 11.1857 | 10.7417 | 10.8826 | 10.6659 | 10.6105 | 10.8911 | 10.1993 | 10.7456 | 6.0162 | 9.9764 | 10.1384 | 10.459 |
| 219573_at | 55604 | LRRC16A | leucine rich repeat containing 16A | 0.6268 | 0.02994 | 0.1457 | 5.7815 | 6.0338 | 8.82 | 6.2451 | 6.2197 | 5.934 | 5.922 | 5.4828 | 5.4036 | 5.8234 | 5.8123 | 5.2594 |
| 201074_at | 6599 | SMARCC1 | SWI/SNF related, matrix associated, actin dependent regulator of chromatin, subfamily c, member 1 | 0.6192 | 0.1038 | 0.3095 | 9.3502 | 9.3953 | 9.4187 | 9.1745 | 9.4486 | 9.2264 | 8.7752 | 9.1224 | 9.0552 | 9.153 | 9.5703 | 8.9263 |
| 201397_at | 26227 | PHGDH | phosphoglycerate dehydrogenase | 0.6182 | 0.1178 | 0.3387 | 10.0183 | 10.6815 | 10.737 | 10.8479 | 10.647 | 10.5815 | 10.7254 | 10.4304 | 9.7514 | 10.1028 | 10.0685 | 10.1028 |
| 207993_s_at | 11261 | CHP | calcium binding protein P22 | 0.6154 | 0.1118 | 0.3244 | 10.4535 | 10.8808 | 10.7641 | 10.5765 | 10.6708 | 10.8479 | 10.2889 | 10.8176 | 9.9746 | 10.1251 | 10.468 | 10.7003 |
| 212124_at | 57178 | ZMIZ1 | zinc finger, MIZ-type containing 1 | 0.6153 | 0.1098 | 0.3215 | 10.5353 | 9.7825 | 10.1993 | 10.3126 | 10.7417 | 9.8902 | 10.1384 | 9.5469 | 9.3202 | 9.6384 | 9.0888 | 10.468 |
| 218619_s_at | 6839 | SUV39H1 | suppressor of variegation 3-9 homolog 1 (Drosophila) | 0.6139 | 0.08383 | 0.2645 | 8.1256 | 8.2999 | 8.402 | 8.3263 | 8.3227 | 8.0294 | 8.2102 | 8.0155 | 8.1034 | 7.8224 | 8.1958 | 8.109 |
| 204828_at | 5883 | RAD9A | RAD9 homolog A (S. pombe) | 0.6132 | 0.03393 | 0.1558 | 4.9025 | 5.3457 | 5.3457 | 5.2953 | 7.6283 | 5.3804 | 4.741 | 4.7616 | 4.7891 | 4.741 | 4.9889 | 5.3545 |
| 211729_x_at | 644 | BLVRA | biliverdin reductase A | 0.6091 | 0.0998 | 0.3003 | 10.468 | 10.4396 | 10.468 | 10.5491 | 10.5669 | 10.1771 | 10.2533 | 10.3126 | 10.0604 | 10.2384 | 10.3126 | 10.4803 |
| 202187_s_at | 5525 | PPP2R5A | protein phosphatase 2, regulatory subunit B', alpha | 0.6076 | 0.08982 | 0.2771 | 9.4562 | 8.5303 | 9.3333 | 9.7859 | 9.4001 | 9.0099 | 9.0651 | 8.9824 | 8.941 | 8.4418 | 8.9954 | 8.5629 |
| 202009_at | 11344 | TWF2 | twinfilin, actin-binding protein, homolog 2 (Drosophila) | 0.6069 | 0.07186 | 0.2423 | 7.3802 | 7.6412 | 7.4718 | 7.863 | 7.6989 | 7.6467 | 7.509 | 7.5569 | 7.0955 | 7.5831 | 6.8804 | 7.4112 |
| 201011_at | 6184 | RPN1 | ribophorin I | 0.6054 | 0.08184 | 0.2599 | 13.8177 | 14.2088 | 14.2088 | 14.0469 | 14.0469 | 13.962 | 13.8177 | 14.0469 | 13.3248 | 13.962 | 13.5329 | 14.0045 |
| 206562_s_at | 1452 | CSNK1A1 | casein kinase 1, alpha 1 | 0.6049 | 0.1078 | 0.3175 | 8.6794 | 9.0374 | 8.4293 | 9.0166 | 9.2051 | 9.0166 | 8.7117 | 8.7482 | 8.4583 | 8.511 | 8.82 | 8.5757 |
| 203188_at | 11041 | B3GNT1 | UDP-GlcNAc:betaGal beta-1,3-N-acetylglucosaminyltransferase 1 | 0.6027 | 0.1517 | 0.3925 | 8.2824 | 8.3407 | 8.1754 | 8.0829 | 8.4548 | 8.3198 | 7.6653 | 8.3958 | 7.3429 | 7.915 | 8.2587 | 8.1665 |
| 212833_at | 91137 | SLC25A46 | solute carrier family 25, member 46 | 0.5941 | 0.1597 | 0.4035 | 9.7825 | 9.7963 | 9.684 | 9.7514 | 9.8403 | 9.6634 | 9.5062 | 9.7894 | 9.0374 | 9.2559 | 9.8572 | 9.684 |
| 202696_at | 9943 | OXSR1 | oxidative-stress responsive 1 | 0.5839 | 0.08184 | 0.2599 | 7.5099 | 7.9171 | 7.4967 | 8.6173 | 8.4093 | 7.4661 | 7.3884 | 7.6101 | 7.1221 | 7.3854 | 7.5322 | 7.7989 |
| 203067_at | 8050 | PDHX | pyruvate dehydrogenase complex, component X | 0.5651 | 0.1098 | 0.3215 | 9.4224 | 9.3848 | 9.4486 | 9.5056 | 9.631 | 9.6788 | 9.415 | 9.4293 | 9.1834 | 9.4881 | 9.4462 | 9.3347 |
| 215116_s_at | 1759 | DNM1 | dynamin 1 | 0.5642 | 0.1617 | 0.4054 | 5.2118 | 5.1287 | 5.3719 | 5.3313 | 5.5139 | 5.3097 | 5.338 | 4.9592 | 5.2205 | 5.0402 | 4.9736 | 5.3097 |
| 210640_s_at | 2852 | GPER | G protein-coupled estrogen receptor 1 | 0.5603 | 0.07585 | 0.2506 | 6.2531 | 6.5178 | 6.1227 | 6.0979 | 6.1674 | 10.6417 | 5.8083 | 6.0589 | 5.9373 | 5.7865 | 5.9718 | 5.7626 |
| 202174_s_at | 5108 | PCM1 | pericentriolar material 1 | 0.5598 | 0.1297 | 0.3595 | 6.3957 | 6.2681 | 5.934 | 6.4096 | 6.2018 | 6.5178 | 5.8629 | 6.4795 | 5.6982 | 6.0639 | 6.1582 | 5.8234 |
| 218995_s_at | 1906 | EDN1 | endothelin 1 | 0.5591 | 0.1477 | 0.3894 | 8.6896 | 8.2824 | 8.7702 | 8.8424 | 8.6965 | 8.8628 | 8.4241 | 8.3294 | 8.3958 | 8.3111 | 8.7786 | 8.5886 |
| 203342_at | 10245 | TIMM17B | translocase of inner mitochondrial membrane 17 homolog B (yeast) | 0.5562 | 0.07784 | 0.2546 | 14.3206 | 11.9623 | 12.0358 | 11.9303 | 12.0869 | 11.5542 | 11.6232 | 11.6793 | 11.2734 | 11.7779 | 11.6252 | 11.8824 |
| 201761_at | 10797 | MTHFD2 | methylenetetrahydrofolate dehydrogenase (NADP+ dependent) 2, methenyltetrahydrofolate cyclohydrolase | 0.5497 | 0.1497 | 0.3894 | 11.9142 | 12.0358 | 12.0613 | 11.8192 | 11.5857 | 11.8922 | 11.3122 | 11.8527 | 11.5382 | 11.7221 | 11.8922 | 11.7111 |
| 202949_s_at | 2274 | FHL2 | four and a half LIM domains 2 | 0.5466 | 0.1277 | 0.357 | 12.9116 | 12.9116 | 12.7892 | 12.5651 | 13.2745 | 12.8415 | 12.5073 | 13.0802 | 12.3655 | 12.3797 | 12.6305 | 12.7014 |
| 217758_s_at | 56889 | TM9SF3 | transmembrane 9 superfamily member 3 | 0.5462 | 0.2016 | 0.4512 | 9.7626 | 9.6523 | 9.3202 | 9.5193 | 9.7072 | 9.2384 | 9.498 | 9.7275 | 8.7752 | 8.4056 | 9.6788 | 7.1782 |
| 209435_s_at | 9181 | ARHGEF2 | Rho/Rac guanine nucleotide exchange factor (GEF) 2 | 0.5453 | 0.1058 | 0.3145 | 8.5211 | 9.153 | 8.6965 | 9.1224 | 8.901 | 9.3614 | 8.7117 | 9.2709 | 8.1754 | 8.3629 | 8.3198 | 8.6032 |
| 217168_s_at | 9709 | HERPUD1 | homocysteine-inducible, endoplasmic reticulum stress-inducible, ubiquitin-like domain member 1 | 0.5429 | 0.1337 | 0.3643 | 8.3743 | 8.3872 | 8.587 | 8.1893 | 8.6061 | 8.4241 | 8.4014 | 8.0403 | 7.7893 | 8.2824 | 6.8292 | 8.6336 |
| 203050_at | 7158 | TP53BP1 | tumor protein p53 binding protein 1 | 0.5416 | 0.1617 | 0.4054 | 8.5346 | 8.6652 | 8.1602 | 8.2903 | 8.3883 | 8.4056 | 7.9457 | 8.4056 | 7.9299 | 8.347 | 8.3465 | 8.206 |
| 204662_at | 9738 | CCP110 | centriolar coiled coil protein 110kDa | 0.5409 | 0.1697 | 0.4145 | 7.706 | 7.9604 | 7.4186 | 11.0671 | 10.3354 | 7.5793 | 7.2549 | 7.8935 | 7.5658 | 7.7416 | 7.9335 | 7.6989 |
| 217934_x_at | 10273 | STUB1 | STIP1 homology and U-box containing protein 1, E3 ubiquitin protein ligase | 0.5405 | 0.1717 | 0.4145 | 11.8824 | 12.0869 | 11.8741 | 12.0242 | 12.015 | 11.9142 | 11.8403 | 11.9552 | 11.6252 | 11.5542 | 12.1105 | 11.7544 |
| 217989_at | 51170 | HSD17B11 | hydroxysteroid (17-beta) dehydrogenase 11 | 0.5389 | 0.1737 | 0.4163 | 11.1997 | 11.1258 | 11.1393 | 11.2558 | 10.972 | 11.0993 | 10.6659 | 11.1997 | 10.4832 | 11.0282 | 10.9335 | 11.2169 |
| 218852_at | 55012 | PPP2R3C | protein phosphatase 2, regulatory subunit B'', gamma | 0.5381 | 0.1497 | 0.3894 | 9.3848 | 9.0755 | 9.5062 | 9.6384 | 9.3686 | 9.459 | 8.9323 | 9.2051 | 8.8371 | 9.1707 | 9.5062 | 9.3589 |
| 214259_s_at | 8574 | AKR7A2 | aldo-keto reductase family 7, member A2 (aflatoxin aldehyde reductase) | 0.538 | 0.001996 | 0.03683 | 10.5568 | 10.6859 | 10.7157 | 10.7843 | 10.7157 | 10.4803 | 10.3917 | 10.6417 | 9.9273 | 10.5568 | 10.5491 | 7.2378 |
| 203007_x_at | 10434 | LYPLA1 | lysophospholipase I | 0.5372 | 0.2116 | 0.4629 | 9.2093 | 9.3333 | 9.0407 | 9.3913 | 9.0374 | 9.3589 | 6.4074 | 9.3255 | 5.3418 | 9.1348 | 9.3635 | 9.4438 |
| 202224_at | 1398 | CRK | v-crk sarcoma virus CT10 oncogene homolog (avian) | 0.536 | 0.03792 | 0.1656 | 8.1223 | 8.4548 | 8.4093 | 8.2553 | 8.5681 | 7.95 | 8.1122 | 8.0487 | 8.0487 | 6.1558 | 7.9171 | 8.2102 |
| 201719_s_at | 2037 | EPB41L2 | erythrocyte membrane protein band 4.1-like 2 | 0.5348 | 0.1277 | 0.357 | 9.5221 | 9.7626 | 9.5013 | 9.7119 | 9.7275 | 9.3686 | 9.5193 | 9.644 | 9.1048 | 9.3361 | 9.3288 | 9.5336 |
| 201729_s_at | 9703 | KIAA0100 | KIAA0100 | 0.5342 | 0.09381 | 0.2858 | 6.4201 | 6.4548 | 6.1358 | 10.6137 | 6.1169 | 6.2812 | 5.694 | 6.3152 | 5.4539 | 5.6828 | 5.9889 | 6.1558 |
| 203105_s_at | 10059 | DNM1L | dynamin 1-like | 0.5315 | 0.2475 | 0.5084 | 9.9273 | 9.8338 | 9.9424 | 9.7963 | 9.8141 | 9.9633 | 9.9746 | 10.1666 | 4.1186 | 10.365 | 10.3006 | 3.9717 |
| 201032_at | 10904 | BLCAP | bladder cancer associated protein | 0.5289 | 0.1637 | 0.4073 | 11.3254 | 11.0885 | 11.0165 | 11.2718 | 10.7777 | 11.1114 | 10.5209 | 11.2558 | 9.8499 | 10.7157 | 11.04 | 10.9943 |
| 216836_s_at | 2064 | ERBB2 | v-erb-b2 erythroblastic leukemia viral oncogene homolog 2, neuro/glioblastoma derived oncogene homolog (avian) | 0.5281 | 0.1198 | 0.3435 | 7.3114 | 7.6144 | 7.5073 | 7.6101 | 7.9529 | 7.9685 | 7.2549 | 7.913 | 6.9209 | 7.4967 | 7.3707 | 7.1029 |
| 202749_at | 7485 | WRB | tryptophan rich basic protein | 0.5267 | 0.1337 | 0.3643 | 7.2921 | 6.9125 | 6.8435 | 6.887 | 7.0192 | 6.8475 | 6.9209 | 6.7515 | 6.492 | 6.6264 | 7.0585 | 6.7713 |
| 207805_s_at | 5715 | PSMD9 | proteasome (prosome, macropain) 26S subunit, non-ATPase, 9 | 0.5257 | 0.08184 | 0.2599 | 9.1754 | 9.3361 | 9.3361 | 9.5703 | 9.4825 | 9.3361 | 9.1075 | 9.3202 | 9.2051 | 9.3137 | 9.2559 | 9.3288 |
| 200081_s_at | 6194 | RPS6 | ribosomal protein S6 | 0.5217 | 0.1557 | 0.3965 | 14.5815 | 14.6935 | 14.6935 | 14.6935 | 14.5815 | 14.5815 | 14.2088 | 14.5815 | 14.4357 | 14.4357 | 14.6935 | 14.6935 |
| 203726_s_at | 3909 | LAMA3 | laminin, alpha 3 | 0.519 | 0.1557 | 0.3965 | 4.4949 | 4.4625 | 4.1498 | 4.6841 | 4.3843 | 3.8267 | 4.1776 | 4.0809 | 3.8267 | 4.4114 | 4.0809 | 3.6316 |
| 207181_s_at | 840 | CASP7 | caspase 7, apoptosis-related cysteine peptidase | 0.5152 | 0.2255 | 0.4837 | 8.5986 | 8.5389 | 7.0813 | 8.0719 | 8.4231 | 8.3898 | 8.109 | 8.0444 | 7.5316 | 6.8247 | 8.1062 | 6.7698 |
| 201548_s_at | 10765 | KDM5B | lysine (K)-specific demethylase 5B | 0.5086 | 0.1118 | 0.3244 | 6.2156 | 6.102 | 6.0115 | 6.1479 | 6.4857 | 5.7223 | 5.5304 | 5.8083 | 5.7626 | 5.6421 | 5.7924 | 6.4292 |
| 203512_at | 27095 | TRAPPC3 | trafficking protein particle complex 3 | 0.5052 | 0.01996 | 0.1243 | 11.5382 | 11.5382 | 11.3634 | 11.1746 | 11.3791 | 11.3122 | 10.737 | 6.4485 | 10.908 | 11.1857 | 11.1746 | 11.5857 |
| 218001_at | 51116 | MRPS2 | mitochondrial ribosomal protein S2 | 0.5048 | 0.1497 | 0.3894 | 9.8403 | 9.2368 | 10.1384 | 9.0651 | 9.1048 | 8.82 | 8.9522 | 8.9522 | 9.4116 | 8.9527 | 8.8424 | 8.941 |
| 203725_at | 1647 | GADD45A | growth arrest and DNA-damage-inducible, alpha | 0.5031 | 0.1317 | 0.3629 | 11.295 | 11.3002 | 11.5656 | 11.1393 | 11.5278 | 11.4384 | 11.3401 | 11.2718 | 11.1393 | 11.3791 | 11.161 | 11.1857 |
| 204039_at | 1050 | CEBPA | CCAAT/enhancer binding protein (C/EBP), alpha | 0.5029 | 0.2056 | 0.4591 | 3.0296 | 3.0296 | 3.0296 | 3.0296 | 4.0394 | 3.2386 | 3.0296 | 3.0296 | 3.0296 | 3.0296 | 3.0296 | 3.0296 |
| 201875_s_at | 9019 | MPZL1 | myelin protein zero-like 1 | 0.4972 | 0.1597 | 0.4035 | 10.647 | 10.5324 | 10.5353 | 10.5669 | 10.1514 | 9.8994 | 10.4209 | 10.232 | 9.5221 | 9.9038 | 10.2889 | 10.1154 |
| 201727_s_at | 1994 | ELAVL1 | ELAV (embryonic lethal, abnormal vision, Drosophila)-like 1 (Hu antigen R) | 0.4963 | 0.1756 | 0.419 | 6.4951 | 8.8761 | 6.2197 | 6.1358 | 6.599 | 6.224 | 6.1199 | 6.3867 | 6.2218 | 5.7707 | 6.2066 | 6.0979 |
| 218399_s_at | 55038 | CDCA4 | cell division cycle associated 4 | 0.4938 | 0.1617 | 0.4054 | 6.4857 | 6.6679 | 6.1916 | 6.1169 | 6.6264 | 6.2681 | 6.2197 | 6.5065 | 5.9556 | 6.1358 | 6.3064 | 5.7815 |
| 201756_at | 6118 | RPA2 | replication protein A2, 32kDa | 0.4937 | 0.1297 | 0.3595 | 10.5295 | 9.6384 | 9.5434 | 9.9038 | 10.7254 | 10.2247 | 9.7268 | 10.212 | 9.8854 | 9.7626 | 9.2051 | 9.1048 |
| 208945_s_at | 8678 | BECN1 | beclin 1, autophagy related | 0.4925 | 0.1417 | 0.3797 | 8.7752 | 8.7006 | 8.5834 | 8.7702 | 8.7161 | 8.5681 | 8.4362 | 8.714 | 7.915 | 8.7752 | 8.5303 | 8.5681 |
| 218086_at | 56654 | NPDC1 | neural proliferation, differentiation and control, 1 | 0.4923 | 0.1497 | 0.3894 | 7.8329 | 8.351 | 8.0719 | 8.4443 | 8.4231 | 8.3257 | 8.1415 | 7.9685 | 7.8388 | 8.3407 | 6.3555 | 7.9829 |
| 201589_at | 8243 | SMC1A | structural maintenance of chromosomes 1A | 0.4916 | 0.01397 | 0.1059 | 10.5669 | 10.7621 | 10.7601 | 10.7157 | 10.5704 | 10.65 | 10.5568 | 10.5815 | 10.633 | 10.5353 | 10.4803 | 8.5303 |
| 221046_s_at | 29083 | GTPBP8 | GTP-binding protein 8 (putative) | 0.4914 | 0.2116 | 0.4629 | 9.6671 | 9.8141 | 9.9038 | 9.8572 | 9.9424 | 9.9038 | 9.6882 | 9.8248 | 9.4881 | 9.631 | 9.8854 | 9.8338 |
| 213190_at | 91949 | COG7 | component of oligomeric golgi complex 7 | 0.4884 | 0.1417 | 0.3797 | 11.4675 | 11.3791 | 11.7779 | 11.3906 | 11.7333 | 11.7005 | 11.5108 | 11.5185 | 11.3002 | 11.3906 | 11.5656 | 11.2558 |
| 210153_s_at | 4200 | ME2 | malic enzyme 2, NAD(+)-dependent, mitochondrial | 0.4851 | 0.1517 | 0.3925 | 8.5264 | 8.9821 | 8.855 | 9.2281 | 9.1559 | 8.9532 | 8.5629 | 8.7752 | 8.5389 | 8.7883 | 8.9476 | 8.8807 |
| 202100_at | 5899 | RALB | v-ral simian leukemia viral oncogene homolog B (ras related; GTP binding protein) | 0.4824 | 0.1397 | 0.3775 | 9.6634 | 9.3502 | 9.459 | 9.2264 | 9.2627 | 9.18 | 8.9817 | 9.4724 | 8.9522 | 9.1559 | 9.2281 | 9.2702 |
| 201913_s_at | 80347 | COASY | CoA synthase | 0.4816 | 0.1477 | 0.3894 | 6.9763 | 6.9655 | 6.3908 | 6.3339 | 6.9694 | 6.6032 | 6.6115 | 7.0204 | 6.1199 | 6.1139 | 6.256 | 6.2018 |
| 217900_at | 55699 | IARS2 | isoleucyl-tRNA synthetase 2, mitochondrial | 0.4803 | 0.01397 | 0.1059 | 11.6464 | 11.1857 | 11.2899 | 11.2365 | 11.3634 | 11.3634 | 11.0671 | 11.3188 | 11.1746 | 11.0563 | 11.2734 | 8.1393 |
| 208898_at | 51382 | ATP6V1D | ATPase, H+ transporting, lysosomal 34kDa, V1 subunit D | 0.4802 | 0.2555 | 0.5131 | 11.2169 | 11.2124 | 11.1857 | 11.0054 | 11.1857 | 10.9943 | 10.7417 | 11.161 | 11.04 | 10.647 | 11.1258 | 11.1393 |
| 201361_at | 79073 | TMEM109 | transmembrane protein 109 | 0.4773 | 0.1337 | 0.3643 | 8.9674 | 9.0428 | 8.8501 | 8.9817 | 8.9854 | 9.1101 | 8.9263 | 8.907 | 8.9323 | 8.855 | 8.9175 | 9.0091 |
| 217911_s_at | 9531 | BAG3 | BCL2-associated athanogene 3 | 0.4717 | 0.1477 | 0.3894 | 11.4259 | 11.4145 | 11.3882 | 11.3002 | 11.2558 | 11.3791 | 11.2365 | 11.4025 | 10.7003 | 11.2417 | 11.3254 | 11.3401 |
| 210788_s_at | 51635 | DHRS7 | dehydrogenase/reductase (SDR family) member 7 | 0.4692 | 0.1497 | 0.3894 | 10.4209 | 10.5734 | 10.653 | 10.4209 | 10.5073 | 10.7456 | 10.6074 | 10.3818 | 9.7963 | 10.3818 | 10.4396 | 10.5353 |
| 201498_at | 7874 | USP7 | ubiquitin specific peptidase 7 (herpes virus-associated) | 0.467 | 0.2515 | 0.5092 | 6.1958 | 5.9356 | 12.6867 | 6.0162 | 5.9924 | 5.7482 | 5.5606 | 6.0314 | 5.2953 | 5.6982 | 5.7124 | 5.8782 |
| 201608_s_at | 11137 | PWP1 | PWP1 homolog (S. cerevisiae) | 0.4657 | 0.1756 | 0.419 | 10.7003 | 10.7157 | 10.4803 | 11.3791 | 11.8192 | 10.7843 | 10.9406 | 10.459 | 10.212 | 11.0778 | 10.2737 | 10.5013 |
| 202996_at | 57804 | POLD4 | polymerase (DNA-directed), delta 4 | 0.4645 | 0.1796 | 0.4254 | 8.4221 | 8.4418 | 8.3102 | 8.4075 | 8.4418 | 7.9299 | 8.0444 | 8.1893 | 8.1106 | 8.2903 | 8.0747 | 8.347 |
| 204458_at | 23659 | PLA2G15 | phospholipase A2, group XV | 0.4629 | 0.2475 | 0.5084 | 7.9217 | 8.206 | 8.2824 | 7.7499 | 8.0487 | 8.2638 | 7.5507 | 7.5099 | 6.992 | 8.109 | 8.0606 | 8.2999 |
| 209040_s_at | 5696 | PSMB8 | proteasome (prosome, macropain) subunit, beta type, 8 (large multifunctional peptidase 7) | 0.4613 | 0.03992 | 0.1705 | 10.4155 | 10.647 | 10.647 | 10.653 | 10.3719 | 10.633 | 10.448 | 10.6859 | 10.042 | 10.3671 | 10.4304 | 5.7223 |
| 207334_s_at | 7048 | TGFBR2 | transforming growth factor, beta receptor II (70/80kDa) | 0.4554 | 0.3234 | 0.5739 | 5.9556 | 5.9503 | 5.9924 | 5.6292 | 9.8461 | 5.663 | 5.9924 | 5.8234 | 5.5373 | 5.2953 | 5.7482 | 5.4347 |
| 217995_at | 58472 | SQRDL | sulfide quinone reductase-like (yeast) | 0.4548 | 0.2375 | 0.4995 | 7.4767 | 7.707 | 7.708 | 7.8329 | 7.706 | 7.2502 | 7.5158 | 7.2747 | 7.6219 | 7.5322 | 7.5158 | 7.1241 |
| 201819_at | 949 | SCARB1 | scavenger receptor class B, member 1 | 0.4542 | 0.1876 | 0.4338 | 6.7388 | 7.3028 | 7.1323 | 7.4625 | 7.3226 | 7.0192 | 6.855 | 6.3438 | 7.632 | 6.4262 | 6.6115 | 7.0908 |
| 203288_at | 9710 | KIAA0355 | KIAA0355 | 0.454 | 0.2735 | 0.5303 | 5.3313 | 5.251 | 5.2031 | 5.338 | 5.4828 | 5.0889 | 5.3457 | 4.7891 | 5.1925 | 5.5373 | 4.7891 | 4.7616 |
| 204999_s_at | 22809 | ATF5 | activating transcription factor 5 | 0.4509 | 0.3134 | 0.5655 | 10.042 | 9.7514 | 8.3475 | 9.7268 | 9.804 | 9.2841 | 9.4631 | 9.2325 | 9.3545 | 7.9069 | 9.3037 | 8.2329 |
| 209179_s_at | 79143 | MBOAT7 | membrane bound O-acyltransferase domain containing 7 | 0.4493 | 0.2754 | 0.5303 | 10.3006 | 10.4861 | 10.6815 | 10.365 | 10.5815 | 10.5765 | 7.9685 | 10.0527 | 11.669 | 11.3254 | 8.4293 | 9.0374 |
| 204531_s_at | 672 | BRCA1 | breast cancer 1, early onset | 0.4484 | 0.2794 | 0.5317 | 7.8568 | 7.6989 | 7.7314 | 7.3914 | 7.8091 | 7.6719 | 7.5418 | 7.6283 | 7.172 | 7.8309 | 7.6283 | 7.2386 |
| 200887_s_at | 6772 | STAT1 | signal transducer and activator of transcription 1, 91kDa | 0.4472 | 0.1856 | 0.4322 | 10.1028 | 9.3037 | 9.2264 | 9.9424 | 9.3333 | 9.2134 | 9.804 | 8.7387 | 9.5971 | 9.0467 | 8.8424 | 8.8314 |
| 214447_at | 2113 | ETS1 | v-ets erythroblastosis virus E26 oncogene homolog 1 (avian) | 0.4466 | 0.1856 | 0.4322 | 7.6906 | 7.479 | 8.4698 | 7.4661 | 8.0069 | 8.0719 | 6.9308 | 7.373 | 8.4647 | 6.8922 | 7.7129 | 7.172 |
| 203315_at | 8440 | NCK2 | NCK adaptor protein 2 | 0.4463 | 0.2016 | 0.4512 | 5.7482 | 6.1605 | 5.8199 | 5.8408 | 5.8199 | 5.8408 | 5.6525 | 6.1255 | 5.1767 | 5.4117 | 5.5235 | 5.9924 |
| 221514_at | 10813 | UTP14A | UTP14, U3 small nucleolar ribonucleoprotein, homolog A (yeast) | 0.4459 | 0.1657 | 0.4081 | 7.937 | 8.4443 | 8.4924 | 8.6398 | 8.7006 | 7.7599 | 7.6047 | 8.439 | 7.9848 | 7.716 | 8.5237 | 7.5108 |
| 210151_s_at | 8444 | DYRK3 | dual-specificity tyrosine-(Y)-phosphorylation regulated kinase 3 | 0.4458 | 0.2515 | 0.5092 | 7.7268 | 7.4609 | 7.6144 | 7.5703 | 7.5831 | 7.4112 | 7.5158 | 7.2468 | 7.4112 | 7.568 | 7.2999 | 7.6219 |
| 203291_at | 4850 | CNOT4 | CCR4-NOT transcription complex, subunit 4 | 0.4404 | 0.2395 | 0.4995 | 7.1166 | 7.3854 | 6.9495 | 7.0813 | 6.7367 | 7.1002 | 7.0974 | 6.9657 | 6.618 | 7.0033 | 6.9258 | 6.7086 |
| 202153_s_at | 23636 | NUP62 | nucleoporin 62kDa | 0.4374 | 0.2116 | 0.4629 | 8.587 | 8.4241 | 8.206 | 8.7883 | 8.5757 | 8.829 | 8.2366 | 8.3872 | 7.8091 | 7.915 | 8.6205 | 8.8085 |
| 202705_at | 9133 | CCNB2 | cyclin B2 | 0.4364 | 0.5848 | 0.7905 | 11.6213 | 11.5656 | 11.6252 | 11.7221 | 11.6213 | 11.4675 | 12.015 | 7.7416 | 12.0242 | 12.0242 | 7.5158 | 12.1105 |
| 217752_s_at | 55748 | CNDP2 | CNDP dipeptidase 2 (metallopeptidase M20 family) | 0.436 | 0.2555 | 0.5131 | 12.0613 | 12.1267 | 11.9142 | 12.0869 | 12.0485 | 11.4145 | 11.2718 | 11.9623 | 11.5656 | 11.8192 | 11.9303 | 11.7221 |
| 200670_at | 7494 | XBP1 | X-box binding protein 1 | 0.434 | 0.2515 | 0.5092 | 9.1012 | 9.4438 | 9.2281 | 9.1745 | 8.8233 | 9.1707 | 9.4224 | 9.0888 | 8.8266 | 8.6754 | 8.6173 | 9.0091 |
| 201611_s_at | 23463 | ICMT | isoprenylcysteine carboxyl methyltransferase | 0.4333 | 0.2096 | 0.4627 | 9.8293 | 9.7119 | 9.936 | 9.4438 | 9.5248 | 9.7678 | 9.2368 | 9.153 | 8.9263 | 9.1075 | 10.3293 | 9.6412 |
| 201827_at | 6603 | SMARCD2 | SWI/SNF related, matrix associated, actin dependent regulator of chromatin, subfamily d, member 2 | 0.4332 | 0.2275 | 0.4859 | 7.0813 | 7.1801 | 7.2245 | 7.1241 | 7.2434 | 7.3114 | 7.0846 | 7.0813 | 6.8975 | 6.9387 | 7.2747 | 7.2619 |
| 221779_at | 85377 | MICALL1 | MICAL-like 1 | 0.4324 | 0.2395 | 0.4995 | 6.4672 | 6.5008 | 6.1777 | 6.4376 | 6.4231 | 6.3205 | 6.2546 | 6.4074 | 5.9924 | 6.2114 | 6.5405 | 6.0868 |
| 202423_at | 7994 | KAT6A | K(lysine) acetyltransferase 6A | 0.4318 | 0.2395 | 0.4995 | 7.5461 | 7.3649 | 7.5971 | 7.3439 | 7.4617 | 7.6827 | 7.6283 | 7.4474 | 7.3174 | 6.9655 | 7.479 | 7.209 |
| 201561_s_at | 22883 | CLSTN1 | calsyntenin 1 | 0.4281 | 0.3174 | 0.5674 | 8.6448 | 8.5944 | 8.4221 | 7.9604 | 8.5144 | 8.587 | 7.5322 | 8.0281 | 7.7356 | 8.6631 | 8.5834 | 8.347 |
| 212694_s_at | 5096 | PCCB | propionyl CoA carboxylase, beta polypeptide | 0.4268 | 0.2016 | 0.4512 | 9.5801 | 10.1063 | 9.9918 | 10.5568 | 9.9424 | 10.6137 | 9.0428 | 10.1099 | 9.1936 | 10.4209 | 9.6105 | 10.0145 |
| 200752_s_at | 823 | CAPN1 | calpain 1, (mu/I) large subunit | 0.4251 | 0.1896 | 0.4364 | 5.0557 | 5.0635 | 4.9736 | 5.005 | 4.4625 | 5.0832 | 4.5947 | 4.4285 | 4.4456 | 4.4625 | 5.4539 | 4.6498 |
| 212115_at | 90861 | HN1L | hematological and neurological expressed 1-like | 0.4233 | 0.2096 | 0.4627 | 9.0888 | 8.9954 | 8.8807 | 9.148 | 8.8163 | 8.8432 | 8.7482 | 9.0099 | 8.8131 | 8.5852 | 9.0276 | 8.8181 |
| 209576_at | 2770 | GNAI1 | guanine nucleotide binding protein (G protein), alpha inhibiting activity polypeptide 1 | 0.4228 | 0.507 | 0.7389 | 11.3906 | 5.5139 | 5.4828 | 5.5788 | 5.694 | 5.6241 | 5.73 | 5.3804 | 5.3205 | 5.5738 | 5.4036 | 5.4729 |
| 218046_s_at | 51021 | MRPS16 | mitochondrial ribosomal protein S16 | 0.4226 | 0.2455 | 0.5076 | 10.6815 | 10.7843 | 10.7003 | 10.9582 | 11.1746 | 10.708 | 10.5669 | 10.8479 | 10.2447 | 10.9131 | 10.8331 | 10.4209 |
| 202394_s_at | 55324 | ABCF3 | ATP-binding cassette, sub-family F (GCN20), member 3 | 0.4214 | 0.1856 | 0.4322 | 7.7681 | 8.0774 | 7.2813 | 7.479 | 7.2999 | 7.3114 | 6.9531 | 7.6684 | 7.3174 | 7.4661 | 7.028 | 7.2921 |
| 209163_at | 1534 | CYB561 | cytochrome b-561 | 0.4202 | 0.2016 | 0.4512 | 8.0155 | 8.3686 | 7.9732 | 8.5237 | 8.8039 | 8.5177 | 8.1709 | 8.3475 | 7.973 | 8.1709 | 7.6684 | 8.3894 |
| 205202_at | 5110 | PCMT1 | protein-L-isoaspartate (D-aspartate) O-methyltransferase | 0.4192 | 0.4032 | 0.6464 | 11.5278 | 11.5278 | 11.5185 | 11.3634 | 11.6793 | 11.4922 | 11.1592 | 11.4922 | 11.1258 | 11.5278 | 11.5542 | 11.5032 |
| 205562_at | 10557 | RPP38 | ribonuclease P/MRP 38kDa subunit | 0.4164 | 0.4092 | 0.6518 | 7.3226 | 12.1907 | 7.3956 | 7.6754 | 7.3752 | 7.0491 | 6.8975 | 7.5793 | 6.9655 | 7.1166 | 7.4967 | 7.3047 |
| 200614_at | 1213 | CLTC | clathrin, heavy chain (Hc) | 0.4146 | 0.1976 | 0.4484 | 11.7111 | 11.4593 | 11.0563 | 11.4675 | 11.1997 | 11.5656 | 11.1746 | 11.5599 | 10.879 | 11.0993 | 11.1857 | 11.3791 |
| 214274_s_at | 30 | ACAA1 | acetyl-CoA acyltransferase 1 | 0.4135 | 0.3892 | 0.6376 | 5.9772 | 5.922 | 9.0888 | 5.7761 | 5.9149 | 5.5187 | 6.1358 | 5.6189 | 5.6982 | 5.6982 | 5.5688 | 5.6292 |
| 203336_s_at | 9270 | ITGB1BP1 | integrin beta 1 binding protein 1 | 0.4098 | 0.0479 | 0.1889 | 10.5013 | 10.7988 | 10.633 | 10.6417 | 10.6815 | 10.5353 | 4.5382 | 10.5704 | 10.3354 | 10.6417 | 10.7988 | 10.5669 |
| 202507_s_at | 6616 | SNAP25 | synaptosomal-associated protein, 25kDa | 0.4087 | 0.3693 | 0.6173 | 5.1407 | 10.1063 | 5.1925 | 5.4779 | 5.8083 | 5.6241 | 5.7554 | 4.8724 | 5.0832 | 5.8629 | 5.2205 | 4.6966 |
| 217398_x_at | 2597 | GAPDH | glyceraldehyde-3-phosphate dehydrogenase | 0.4082 | 0.001996 | 0.03683 | 15 | 15 | 15 | 15 | 15 | 15 | 15 | 15 | 14.6375 | 15 | 15 | 15 |
| 201984_s_at | 1956 | EGFR | epidermal growth factor receptor | 0.4065 | 0.2495 | 0.5084 | 6.7164 | 6.992 | 7.4501 | 6.7852 | 7.6047 | 7.437 | 7.241 | 7.1751 | 6.7023 | 5.8782 | 7.3226 | 6.3178 |
| 201503_at | 10146 | G3BP1 | GTPase activating protein (SH3 domain) binding protein 1 | 0.4045 | 0.3253 | 0.5764 | 10.0226 | 9.9764 | 9.7514 | 10.2876 | 10.2247 | 10.0808 | 9.6153 | 10.3293 | 9.3768 | 8.7304 | 10.0808 | 10.2533 |
| 204089_x_at | 4216 | MAP3K4 | mitogen-activated protein kinase kinase kinase 4 | 0.4008 | 0.2176 | 0.4718 | 7.0491 | 6.718 | 6.7086 | 6.6336 | 6.661 | 6.8106 | 6.492 | 6.8837 | 6.1811 | 6.3064 | 6.5104 | 7.042 |
| 204729_s_at | 6804 | STX1A | syntaxin 1A (brain) | 0.3984 | 0.2395 | 0.4995 | 8.8416 | 9.5013 | 8.714 | 9.3288 | 9.3202 | 8.6336 | 8.609 | 8.4221 | 8.2519 | 9.331 | 9.1181 | 8.7387 |
| 200602_at | 351 | APP | amyloid beta (A4) precursor protein | 0.3981 | 0.2495 | 0.5084 | 14.8479 | 14.4357 | 14.8479 | 14.8479 | 14.8479 | 14.8479 | 14.5815 | 14.8479 | 14.8479 | 14.5815 | 14.5815 | 14.4357 |
| 208728_s_at | 998 | CDC42 | cell division cycle 42 (GTP binding protein, 25kDa) | 0.3934 | 0.2495 | 0.5084 | 11.247 | 11.3122 | 11.5542 | 11.5542 | 11.7779 | 11.5857 | 11.5108 | 11.3634 | 8.0684 | 11.4675 | 11.6213 | 11.2718 |
| 202830_s_at | 2542 | SLC37A4 | solute carrier family 37 (glucose-6-phosphate transporter), member 4 | 0.3909 | 0.2355 | 0.4995 | 5.7223 | 5.3097 | 5.4117 | 5.5606 | 5.2783 | 5.5688 | 5.5139 | 5.3955 | 4.8322 | 5.251 | 5.3457 | 5.5139 |
| 203135_at | 6908 | TBP | TATA box binding protein | 0.3902 | 0.2375 | 0.4995 | 8.609 | 8.4583 | 8.8432 | 8.5807 | 8.8784 | 8.7151 | 8.3872 | 8.5389 | 8.0383 | 8.5852 | 8.7304 | 8.7883 |
| 203456_at | 11230 | PRAF2 | PRA1 domain family, member 2 | 0.39 | 0.2555 | 0.5131 | 9.24 | 9.4187 | 9.2841 | 9.2134 | 9.1386 | 9.0485 | 8.5903 | 9.4486 | 8.8314 | 8.904 | 9.4881 | 8.9476 |
| 207419_s_at | 5880 | RAC2 | ras-related C3 botulinum toxin substrate 2 (rho family, small GTP binding protein Rac2) | 0.3895 | 0.2794 | 0.5317 | 9.3933 | 9.4687 | 9.5056 | 8.9367 | 9.7401 | 8.7151 | 9.8141 | 8.941 | 8.2638 | 8.5283 | 9.5013 | 8.2874 |
| 201178_at | 25793 | FBXO7 | F-box protein 7 | 0.3875 | 0.3473 | 0.5949 | 7.4112 | 7.0491 | 6.9896 | 7.1373 | 6.9125 | 7.0064 | 6.7148 | 7.2378 | 6.34 | 7.3707 | 7.1323 | 6.0979 |
| 56197_at | 57048 | PLSCR3 | phospholipid scramblase 3 | 0.3867 | 0.479 | 0.7205 | 7.3707 | 7.17 | 7.0585 | 7.1885 | 9.4611 | 7.4527 | 7.1843 | 7.209 | 7.1801 | 7.0813 | 7.4661 | 7.1524 |
| 214435_x_at | 5898 | RALA | v-ral simian leukemia viral oncogene homolog A (ras related) | 0.3866 | 0.2415 | 0.5015 | 9.7894 | 9.936 | 9.8432 | 9.8338 | 9.4224 | 9.8804 | 9.5548 | 9.8461 | 9.4775 | 9.5783 | 9.5783 | 9.8644 |
| 219184_x_at | 29928 | TIMM22 | translocase of inner mitochondrial membrane 22 homolog (yeast) | 0.3866 | 0.2914 | 0.546 | 8.5144 | 8.4767 | 8.1958 | 8.7978 | 8.6431 | 8.5211 | 8.3903 | 8.3257 | 8.1169 | 8.6465 | 8.5504 | 8.1782 |
| 212403_at | 89910 | UBE3B | ubiquitin protein ligase E3B | 0.3859 | 0.2695 | 0.5281 | 7.7599 | 8.4293 | 8.2771 | 8.5303 | 8.3629 | 7.6101 | 8.4293 | 7.994 | 7.3065 | 7.5507 | 7.9529 | 7.95 |
| 219352_at | 55008 | HERC6 | hect domain and RLD 6 | 0.3857 | 0.2615 | 0.5198 | 8.7482 | 8.6827 | 7.4833 | 8.6545 | 8.9824 | 7.5056 | 7.8091 | 7.9171 | 8.1992 | 8.3782 | 7.7545 | 7.8894 |
| 211747_s_at | 23658 | LSM5 | LSM5 homolog, U6 small nuclear RNA associated (S. cerevisiae) | 0.3856 | 0.3353 | 0.5815 | 13.0242 | 13.1644 | 12.983 | 13.0802 | 12.7014 | 13.1644 | 13.0802 | 12.9116 | 12.5651 | 12.983 | 12.8646 | 12.9116 |
| 200929_at | 10972 | TMED10 | transmembrane emp24-like trafficking protein 10 (yeast) | 0.3844 | 0.4691 | 0.7134 | 11.6252 | 11.6464 | 11.7005 | 11.4977 | 11.5032 | 11.5382 | 10.7601 | 11.7779 | 11.5736 | 10.7417 | 11.5736 | 11.7715 |
| 204252_at | 1017 | CDK2 | cyclin-dependent kinase 2 | 0.3831 | 0.3014 | 0.5572 | 9.4059 | 9.7678 | 9.7894 | 9.6882 | 9.6104 | 9.7275 | 9.8644 | 9.1181 | 9.5056 | 9.5933 | 9.459 | 9.5676 |
| 201462_at | 9805 | SCRN1 | secernin 1 | 0.3826 | 0.2475 | 0.5084 | 8.9323 | 9.2979 | 8.4914 | 9.1101 | 8.8131 | 8.7071 | 8.901 | 8.7006 | 8.8039 | 8.7387 | 8.7049 | 8.6173 |
| 202630_at | 10513 | APPBP2 | amyloid beta precursor protein (cytoplasmic tail) binding protein 2 | 0.38 | 0.2415 | 0.5015 | 6.5529 | 5.9059 | 5.9503 | 6.5498 | 5.922 | 6.5178 | 5.5478 | 5.9373 | 5.928 | 6.1255 | 6.2066 | 6.2812 |
| 220127_s_at | 54850 | FBXL12 | F-box and leucine-rich repeat protein 12 | 0.3743 | 0.3852 | 0.6354 | 7.7599 | 7.7599 | 7.829 | 8.8233 | 7.6684 | 7.7545 | 7.6517 | 7.6358 | 7.8388 | 7.8544 | 7.8184 | 7.4617 |
| 214086_s_at | 10038 | PARP2 | poly (ADP-ribose) polymerase 2 | 0.3721 | 0.3533 | 0.603 | 9.3635 | 9.3913 | 9.4438 | 9.2928 | 9.1909 | 9.3037 | 9.2444 | 9.3347 | 8.9134 | 9.4631 | 9.3137 | 9.0769 |
| 218743_at | 79643 | CHMP6 | charged multivesicular body protein 6 | 0.372 | 0.2435 | 0.5046 | 5.5139 | 5.2594 | 5.2594 | 5.5235 | 5.3097 | 4.841 | 5.2415 | 5.1287 | 5.0635 | 5.3205 | 5.1167 | 5.0402 |
| 204849_at | 10732 | TCFL5 | transcription factor-like 5 (basic helix-loop-helix) | 0.371 | 0.2615 | 0.5198 | 5.0889 | 5.0889 | 4.7616 | 4.7462 | 4.5854 | 4.6365 | 4.8366 | 4.7515 | 4.6498 | 4.7103 | 4.6841 | 4.6041 |
| 204757_s_at | 9854 | C2CD2L | C2CD2-like | 0.3709 | 0.3333 | 0.5801 | 5.5822 | 5.9059 | 6.0709 | 5.9745 | 6.3476 | 6.2805 | 5.251 | 5.7815 | 5.9503 | 5.9356 | 6.0709 | 5.9059 |
| 203574_at | 4783 | NFIL3 | nuclear factor, interleukin 3 regulated | 0.3689 | 0.3054 | 0.5625 | 7.1524 | 7.4501 | 7.5322 | 7.5073 | 6.8515 | 7.0691 | 7.4625 | 7.4609 | 6.4262 | 6.6738 | 6.7367 | 7.2099 |
| 201270_x_at | 23386 | NUDCD3 | NudC domain containing 3 | 0.3674 | 0.2814 | 0.5334 | 9.0741 | 9.3073 | 8.9293 | 8.206 | 9.6671 | 8.206 | 7.5876 | 9.1348 | 8.5037 | 8.6754 | 8.7665 | 8.2527 |
| 121_at | 7849 | PAX8 | paired box 8 | 0.3653 | 0.3094 | 0.5635 | 5.8083 | 6.0209 | 5.7482 | 5.3804 | 6.6394 | 5.8408 | 5.6525 | 5.8083 | 5.928 | 5.4894 | 5.4828 | 5.7707 |
| 201380_at | 10491 | CRTAP | cartilage associated protein | 0.365 | 0.3313 | 0.5797 | 8.4038 | 8.2999 | 8.2519 | 8.4647 | 7.6467 | 8.5091 | 7.2895 | 8.2369 | 7.716 | 8.1196 | 8.5389 | 8.0281 |
| 203130_s_at | 3800 | KIF5C | kinesin family member 5C | 0.3644 | 0.6028 | 0.8032 | 5.5373 | 11.2558 | 5.4497 | 5.7377 | 5.338 | 5.2783 | 5.8548 | 5.663 | 5.4729 | 4.997 | 5.6189 | 5.0557 |
| 203409_at | 1643 | DDB2 | damage-specific DNA binding protein 2, 48kDa | 0.3631 | 0.4251 | 0.6696 | 7.3065 | 7.1944 | 9.1881 | 7.3114 | 7.1944 | 7.1782 | 7.2095 | 7.267 | 6.661 | 7.1944 | 7.5375 | 7.1423 |
| 202076_at | 329 | BIRC2 | baculoviral IAP repeat containing 2 | 0.3628 | 0.2874 | 0.5427 | 9.5469 | 8.6611 | 9.631 | 9.7072 | 9.644 | 9.6721 | 9.459 | 9.1834 | 9.1722 | 9.0166 | 9.0518 | 9.5971 |
| 218845_at | 56940 | DUSP22 | dual specificity phosphatase 22 | 0.3609 | 0.2295 | 0.4891 | 7.2099 | 7.6906 | 9.1707 | 9.3913 | 7.7268 | 9.2928 | 8.4548 | 8.9323 | 7.1801 | 7.3028 | 7.3385 | 7.6047 |
| 218113_at | 23670 | TMEM2 | transmembrane protein 2 | 0.3587 | 0.3413 | 0.5898 | 8.3782 | 8.0487 | 8.0487 | 8.1327 | 7.798 | 8.0684 | 7.716 | 8.2102 | 7.3028 | 7.973 | 8.1415 | 8.009 |
| 208309_s_at | 10892 | MALT1 | mucosa associated lymphoid tissue lymphoma translocation gene 1 | 0.3556 | 0.3333 | 0.5801 | 7.17 | 6.8106 | 7.2747 | 7.2502 | 6.7148 | 6.3593 | 6.618 | 6.7148 | 5.9718 | 5.5139 | 6.8203 | 7.6122 |
| 207199_at | 7015 | TERT | telomerase reverse transcriptase | 0.3551 | 0.3293 | 0.5783 | 9.6105 | 10.6137 | 10.3695 | 10.3006 | 10.8082 | 10.737 | 10.5295 | 10.5013 | 9.6206 | 9.9167 | 10.1926 | 9.9918 |
| 202349_at | 1861 | TOR1A | torsin family 1, member A (torsin A) | 0.3534 | 0.4671 | 0.7115 | 8.2348 | 8.2102 | 8.1852 | 8.3155 | 8.2553 | 8.2845 | 8.4056 | 8.4443 | 8.3257 | 8.0423 | 8.2797 | 5.101 |
| 204489_s_at | 960 | CD44 | CD44 molecule (Indian blood group) | 0.3509 | 0.491 | 0.7276 | 13.6591 | 13.6034 | 13.6591 | 13.962 | 13.7451 | 13.7451 | 13.6034 | 4.0394 | 14.0469 | 13.6591 | 14.0469 | 14.2088 |
| 203127_s_at | 9517 | SPTLC2 | serine palmitoyltransferase, long chain base subunit 2 | 0.3476 | 0.3074 | 0.563 | 4.7967 | 4.882 | 4.741 | 4.769 | 5.2552 | 4.8917 | 4.5947 | 4.8322 | 4.7515 | 4.2483 | 4.7967 | 5.1167 |
| 202417_at | 9817 | KEAP1 | kelch-like ECH-associated protein 1 | 0.3464 | 0.3433 | 0.5922 | 9.6523 | 9.5703 | 9.4187 | 9.5971 | 9.4687 | 9.5062 | 9.2709 | 9.5676 | 9.0276 | 9.3686 | 9.5056 | 9.7626 |
| 219016_at | 60493 | FASTKD5 | FAST kinase domains 5 | 0.3451 | 0.2934 | 0.5476 | 9.4825 | 8.6032 | 8.941 | 8.609 | 9.2928 | 9.2702 | 8.0487 | 9.4825 | 8.009 | 8.1602 | 9.0957 | 9.24 |
| 204517_at | 5480 | PPIC | peptidylprolyl isomerase C (cyclophilin C) | 0.3441 | 0.2735 | 0.5303 | 8.5144 | 8.4647 | 8.4785 | 8.7598 | 8.4014 | 8.3743 | 8.1602 | 8.6431 | 7.863 | 8.3545 | 8.4014 | 8.6611 |
| 202927_at | 5300 | PIN1 | peptidylprolyl cis/trans isomerase, NIMA-interacting 1 | 0.3419 | 0.4371 | 0.6829 | 10.9582 | 10.8639 | 10.6659 | 10.9278 | 11.1114 | 11.0534 | 10.647 | 10.9278 | 9.8644 | 10.9477 | 10.8826 | 11.0778 |
| 200966_x_at | 226 | ALDOA | aldolase A, fructose-bisphosphate | 0.3419 | 0.3014 | 0.5572 | 7.7314 | 8.109 | 6.1479 | 7.9194 | 6.887 | 6.6943 | 6.7738 | 7.3828 | 6.6943 | 6.8922 | 7.028 | 6.4672 |
| 209095_at | 1738 | DLD | dihydrolipoamide dehydrogenase | 0.3408 | 0.3653 | 0.6138 | 11.0534 | 10.9182 | 10.7777 | 10.6815 | 10.9335 | 10.708 | 10.8176 | 10.8392 | 10.5209 | 10.5209 | 10.908 | 10.8176 |
| 200697_at | 3098 | HK1 | hexokinase 1 | 0.3407 | 0.3154 | 0.567 | 7.1996 | 7.2468 | 8.3545 | 7.2245 | 7.3028 | 7.3402 | 6.7353 | 6.8975 | 6.3669 | 7.6251 | 7.3089 | 7.7268 |
| 204093_at | 902 | CCNH | cyclin H | 0.3399 | 0.3134 | 0.5655 | 9.5056 | 9.3686 | 9.5801 | 9.2744 | 9.3953 | 9.6105 | 9.3727 | 9.5783 | 9.1012 | 9.4438 | 9.2368 | 9.3953 |
| 202220_at | 22889 | KIAA0907 | KIAA0907 | 0.3303 | 0.3054 | 0.5625 | 8.8761 | 9.1448 | 8.7568 | 8.9263 | 8.7568 | 9.0717 | 8.6631 | 8.9175 | 8.8181 | 8.6431 | 9.0099 | 8.8761 |
| 205161_s_at | 8800 | PEX11A | peroxisomal biogenesis factor 11 alpha | 0.3295 | 0.6307 | 0.8127 | 4.9736 | 4.7035 | 4.9889 | 4.6841 | 8.8343 | 4.9216 | 4.9592 | 4.5201 | 5.4036 | 4.882 | 4.6841 | 4.841 |
| 212458_at | 200734 | SPRED2 | sprouty-related, EVH1 domain containing 2 | 0.3289 | 0.2814 | 0.5334 | 9.8902 | 10.8911 | 10.879 | 9.9902 | 9.8499 | 10.879 | 9.6412 | 10.7417 | 9.7894 | 9.8804 | 10.5669 | 9.7894 |
| 203408_s_at | 6304 | SATB1 | SATB homeobox 1 | 0.3288 | 0.2894 | 0.5443 | 4.6365 | 5.5916 | 5.2953 | 4.4114 | 4.1776 | 5.3955 | 4.7616 | 5.4117 | 4.231 | 4.5618 | 4.1186 | 4.337 |
| 202431_s_at | 4609 | MYC | v-myc myelocytomatosis viral oncogene homolog (avian) | 0.3279 | 0.3074 | 0.563 | 9.3202 | 9.4187 | 9.3255 | 9.18 | 9.1754 | 9.4116 | 8.941 | 9.5703 | 8.7631 | 8.8085 | 9.0717 | 9.6882 |
| 201416_at | 6659 | SOX4 | SRY (sex determining region Y)-box 4 | 0.3275 | 0.3473 | 0.5949 | 6.6647 | 7.1241 | 6.5147 | 6.661 | 6.5065 | 5.7815 | 6.3516 | 6.5405 | 6.1558 | 6.5126 | 6.0709 | 6.3933 |
| 201762_s_at | 5721 | PSME2 | proteasome (prosome, macropain) activator subunit 2 (PA28 beta) | 0.3273 | 0.2914 | 0.546 | 12.3315 | 12.8646 | 12.5073 | 12.7892 | 12.417 | 12.5651 | 12.3115 | 12.4521 | 12.4521 | 12.246 | 12.6867 | 12.5912 |
| 201746_at | 7157 | TP53 | tumor protein p53 | 0.3269 | 0.3074 | 0.563 | 7.9707 | 8.1169 | 8.1393 | 7.7681 | 8.2126 | 7.3914 | 7.3089 | 8.2026 | 7.3429 | 7.2245 | 8.135 | 7.9069 |
| 200060_s_at | 10921 | RNPS1 | RNA binding protein S1, serine-rich domain | 0.3268 | 0.2934 | 0.5476 | 12.7014 | 12.015 | 12.3115 | 12.5394 | 12.6867 | 12.6867 | 12.417 | 12.5073 | 12.246 | 12.0869 | 12.3115 | 12.5073 |
| 218292_s_at | 51422 | PRKAG2 | protein kinase, AMP-activated, gamma 2 non-catalytic subunit | 0.3252 | 0.4032 | 0.6464 | 8.4038 | 8.5389 | 8.4014 | 8.1393 | 8.7304 | 8.347 | 8.511 | 8.2951 | 7.9529 | 8.2329 | 8.3782 | 8.4241 |
| 205498_at | 2690 | GHR | growth hormone receptor | 0.3217 | 0.3273 | 0.5779 | 5.2118 | 5.694 | 5.424 | 5.8408 | 6.1358 | 5.9889 | 5.4347 | 5.5856 | 5.6797 | 5.663 | 5.5139 | 5.5688 |
| 209567_at | 23212 | RRS1 | RRS1 ribosome biogenesis regulator homolog (S. cerevisiae) | 0.319 | 0.6806 | 0.8363 | 6.9209 | 7.0955 | 6.8975 | 7.0198 | 11.1258 | 7.0126 | 7.0355 | 7.3419 | 6.868 | 7.0192 | 7.3802 | 6.6679 |
| 205398_s_at | 4088 | SMAD3 | SMAD family member 3 | 0.3167 | 0.3653 | 0.6138 | 8.1393 | 8.2329 | 7.9604 | 7.1084 | 8.4785 | 8.2102 | 8.1393 | 8.3257 | 7.088 | 7.4186 | 7.1423 | 8.0719 |
| 203787_at | 23635 | SSBP2 | single-stranded DNA binding protein 2 | 0.3158 | 0.6727 | 0.8296 | 6.7353 | 6.6943 | 6.794 | 6.6943 | 11.1592 | 6.6264 | 6.8357 | 6.9657 | 7.0204 | 6.9763 | 6.1358 | 6.6943 |
| 205895_s_at | 9221 | NOLC1 | nucleolar and coiled-body phosphoprotein 1 | 0.3138 | 0.4491 | 0.695 | 9.1181 | 9.2281 | 9.24 | 9.1436 | 9.3288 | 9.1203 | 9.1075 | 9.4881 | 8.5986 | 8.402 | 9.5193 | 9.0485 |
| 202942_at | 2109 | ETFB | electron-transfer-flavoprotein, beta polypeptide | 0.3113 | 0.3273 | 0.5779 | 8.9817 | 9.0166 | 9.0099 | 8.9824 | 8.6189 | 8.6715 | 8.3198 | 9.3686 | 8.3475 | 8.7752 | 8.587 | 8.8181 |
| 216321_s_at | 2908 | NR3C1 | nuclear receptor subfamily 3, group C, member 1 (glucocorticoid receptor) | 0.3087 | 0.6208 | 0.8095 | 6.1479 | 5.4961 | 5.9149 | 5.928 | 5.5235 | 10.4861 | 5.9059 | 6.1874 | 5.7924 | 5.6189 | 5.7482 | 6.2197 |
| 218780_at | 29911 | HOOK2 | hook homolog 2 (Drosophila) | 0.3084 | 0.7006 | 0.8461 | 4.8724 | 4.6365 | 4.4625 | 4.4625 | 8.1665 | 4.5947 | 4.6498 | 4.5382 | 4.7616 | 4.6966 | 4.7616 | 4.8724 |
| 210986_s_at | 7168 | TPM1 | tropomyosin 1 (alpha) | 0.3081 | 0.4132 | 0.656 | 13.3248 | 13.3248 | 13.3248 | 13.2745 | 13.4469 | 13.3248 | 13.0242 | 13.6034 | 12.8415 | 13.2745 | 13.4469 | 13.2128 |
| 202148_s_at | 5831 | PYCR1 | pyrroline-5-carboxylate reductase 1 | 0.3061 | 0.3313 | 0.5797 | 8.1545 | 7.8976 | 8.0238 | 8.3465 | 8.3407 | 7.9457 | 7.6122 | 8.5629 | 7.2156 | 8.4749 | 7.6635 | 7.8568 |
| 204420_at | 8061 | FOSL1 | FOS-like antigen 1 | 0.2992 | 0.4012 | 0.6453 | 10.9278 | 10.7456 | 10.9943 | 10.972 | 11.0993 | 11.1746 | 10.9837 | 11.0993 | 10.5073 | 10.7601 | 11.0534 | 10.8479 |
| 205039_s_at | 10320 | IKZF1 | IKAROS family zinc finger 1 (Ikaros) | 0.2968 | 0.7086 | 0.8461 | 3.568 | 3.7758 | 3.4175 | 4.0809 | 11.9303 | 3.8792 | 4.1186 | 4.1498 | 3.7758 | 4.2967 | 3.9934 | 4.0394 |
| 215000_s_at | 9637 | FEZ2 | fasciculation and elongation protein zeta 2 (zygin II) | 0.2944 | 0.3792 | 0.6286 | 7.632 | 7.2921 | 6.34 | 7.4568 | 7.2921 | 5.5916 | 7.2999 | 6.1874 | 6.7706 | 5.6292 | 7.1638 | 5.9438 |
| 219461_at | 56924 | PAK6 | p21 protein (Cdc42/Rac)-activated kinase 6 | 0.2932 | 0.3453 | 0.5935 | 3.7012 | 4.2967 | 4.3685 | 4.1498 | 4.1186 | 3.9717 | 3.7758 | 3.924 | 4.3843 | 3.924 | 3.924 | 3.8792 |
| 202726_at | 3978 | LIG1 | ligase I, DNA, ATP-dependent | 0.2893 | 0.3772 | 0.6275 | 5.4961 | 5.9924 | 6.3383 | 6.0868 | 5.9718 | 6.4672 | 5.6765 | 6.2531 | 5.2594 | 5.9924 | 5.8377 | 6.1255 |
| 219742_at | 80758 | PRR7 | proline rich 7 (synaptic) | 0.2882 | 0.4072 | 0.6496 | 5.4117 | 6.3476 | 5.8083 | 5.5916 | 6.102 | 6.0868 | 5.6828 | 5.7377 | 5.5976 | 6.0115 | 5.934 | 5.3804 |
| 201825_s_at | 51097 | SCCPDH | saccharopine dehydrogenase (putative) | 0.2876 | 0.477 | 0.7205 | 11.451 | 11.5736 | 11.1592 | 11.669 | 11.3906 | 11.6213 | 11.5857 | 11.6464 | 10.7641 | 11.669 | 10.9278 | 11.2734 |
| 211071_s_at | 10962 | MLLT11 | myeloid/lymphoid or mixed-lineage leukemia (trithorax homolog, Drosophila); translocated to, 11 | 0.2875 | 0.3333 | 0.5801 | 8.2999 | 8.27 | 8.0653 | 8.4221 | 8.1923 | 8.4221 | 8.1256 | 8.2329 | 8.1327 | 8.0155 | 8.1545 | 8.4924 |
| 209260_at | 2810 | SFN | stratifin | 0.287 | 0.4611 | 0.7068 | 3.7758 | 3.924 | 3.9717 | 4.0394 | 3.2386 | 4.0151 | 3.2386 | 3.6316 | 3.924 | 3.6316 | 3.7758 | 3.8267 |
| 219390_at | 55033 | FKBP14 | FK506 binding protein 14, 22 kDa | 0.2868 | 0.4232 | 0.6675 | 6.8247 | 6.7607 | 6.6943 | 6.7698 | 6.6647 | 6.6738 | 6.794 | 6.9308 | 6.3205 | 6.6115 | 6.7515 | 6.4888 |
| 201234_at | 3611 | ILK | integrin-linked kinase | 0.2841 | 0.5868 | 0.7905 | 9.9764 | 9.9167 | 9.8141 | 10.0226 | 9.9746 | 9.3913 | 9.7275 | 9.8644 | 9.0083 | 9.7514 | 9.9746 | 9.7825 |
| 201260_s_at | 6856 | SYPL1 | synaptophysin-like 1 | 0.279 | 0.6108 | 0.8072 | 11.8298 | 11.7715 | 11.7111 | 11.7779 | 11.6252 | 11.9303 | 11.9623 | 11.985 | 11.3906 | 10.5686 | 11.8527 | 11.7993 |
| 221562_s_at | 23410 | SIRT3 | sirtuin 3 | 0.2785 | 0.7086 | 0.8461 | 3.8267 | 3.6316 | 3.7758 | 3.6316 | 10.8868 | 3.4175 | 4.231 | 3.7758 | 4.0809 | 3.9717 | 3.9934 | 3.924 |
| 203884_s_at | 22841 | RAB11FIP2 | RAB11 family interacting protein 2 (class I) | 0.2778 | 0.4371 | 0.6829 | 7.0585 | 6.9308 | 7.028 | 7.1885 | 7.1423 | 6.9387 | 7.1423 | 7.075 | 6.743 | 7.1166 | 6.988 | 6.7449 |
| 201098_at | 9276 | COPB2 | coatomer protein complex, subunit beta 2 (beta prime) | 0.2767 | 0.3752 | 0.6252 | 11.6793 | 11.5032 | 11.4675 | 11.247 | 11.1393 | 11.3002 | 11.1997 | 11.6252 | 10.9182 | 10.879 | 11.1393 | 11.6793 |
| 206364_at | 9928 | KIF14 | kinesin family member 14 | 0.2742 | 0.481 | 0.7205 | 7.4767 | 7.8184 | 7.5703 | 7.2595 | 7.9299 | 7.4922 | 7.829 | 7.654 | 6.9308 | 7.437 | 7.6358 | 7.075 |
| 217933_s_at | 51056 | LAP3 | leucine aminopeptidase 3 | 0.2739 | 0.4371 | 0.6829 | 12.0358 | 12.1105 | 11.6793 | 11.985 | 11.7715 | 11.9552 | 11.9303 | 11.7333 | 12.0613 | 11.8824 | 11.8192 | 11.5542 |
| 217789_at | 58533 | SNX6 | sorting nexin 6 | 0.271 | 0.521 | 0.7466 | 9.2559 | 9.1737 | 9.2134 | 9.3037 | 9.0957 | 8.904 | 8.6965 | 9.103 | 9.1423 | 9.2726 | 9.1505 | 9.0276 |
| 203167_at | 7077 | TIMP2 | TIMP metallopeptidase inhibitor 2 | 0.2662 | 0.503 | 0.7375 | 9.1881 | 9.6634 | 9.4687 | 9.2894 | 9.7894 | 9.5952 | 9.3502 | 9.618 | 9.5933 | 9.3465 | 8.6794 | 9.4881 |
| 203727_at | 6499 | SKIV2L | superkiller viralicidic activity 2-like (S. cerevisiae) | 0.2632 | 0.4012 | 0.6453 | 8.0363 | 8.4919 | 7.9457 | 8.27 | 8.1122 | 7.9604 | 7.9604 | 8.4924 | 7.3802 | 7.9685 | 8.0747 | 8.0444 |
| 207842_s_at | 22794 | CASC3 | cancer susceptibility candidate 3 | 0.2611 | 0.3952 | 0.641 | 8.1992 | 7.8773 | 8.0155 | 8.0829 | 7.7545 | 8.0939 | 7.7942 | 8.135 | 7.915 | 7.7335 | 7.9479 | 8.0155 |
| 203046_s_at | 8914 | TIMELESS | timeless homolog (Drosophila) | 0.2608 | 0.7046 | 0.8461 | 9.7541 | 9.5056 | 9.7275 | 9.6788 | 9.5336 | 9.4306 | 9.7401 | 8.181 | 9.4948 | 9.4825 | 9.8994 | 9.6523 |
| 203696_s_at | 5982 | RFC2 | replication factor C (activator 1) 2, 40kDa | 0.2575 | 0.477 | 0.7205 | 7.6122 | 6.9387 | 6.9657 | 6.9458 | 7.4693 | 7.7314 | 7.0456 | 6.9657 | 7.2386 | 7.3956 | 7.4112 | 6.5208 |
| 209774_x_at | 2920 | CXCL2 | chemokine (C-X-C motif) ligand 2 | 0.2573 | 0.6387 | 0.8155 | 4.9889 | 5.3633 | 5.0557 | 5.0557 | 5.1925 | 9.0741 | 5.3955 | 5.5738 | 4.9592 | 5.338 | 5.5856 | 4.9409 |
| 202119_s_at | 8895 | CPNE3 | copine III | 0.2573 | 0.5609 | 0.7737 | 12.2657 | 12.0242 | 12.1519 | 12.1186 | 11.985 | 12.0869 | 11.4259 | 12.1784 | 11.985 | 11.9142 | 12.246 | 12.246 |
| 202788_at | 7867 | MAPKAPK3 | mitogen-activated protein kinase-activated protein kinase 3 | 0.2554 | 0.3872 | 0.6354 | 6.8717 | 6.5745 | 6.5104 | 6.4951 | 7.4218 | 7.5158 | 6.63 | 6.7023 | 7.209 | 6.2805 | 6.6943 | 6.7086 |
| 204042_at | 10810 | WASF3 | WAS protein family, member 3 | 0.2535 | 0.4571 | 0.704 | 7.1423 | 6.9209 | 6.5842 | 6.9655 | 7.0631 | 6.8203 | 6.9888 | 6.7449 | 6.3739 | 6.3476 | 7.2434 | 6.9531 |
| 221567_at | 8996 | NOL3 | nucleolar protein 3 (apoptosis repressor with CARD domain) | 0.2532 | 0.3214 | 0.5714 | 8.5629 | 9.0099 | 11.2124 | 11.161 | 9.045 | 8.8898 | 9.1423 | 9.2928 | 9.6788 | 9.2281 | 9.1048 | 9.2911 |
| 200799_at | 3303 | HSPA1A | heat shock 70kDa protein 1A | 0.2525 | 0.7485 | 0.8653 | 9.9902 | 10.3126 | 9.9633 | 9.8994 | 10.1154 | 9.8403 | 10.4803 | 10.4803 | 9.7626 | 7.3777 | 9.8403 | 10.15 |
| 203171_s_at | 23378 | RRP8 | ribosomal RNA processing 8, methyltransferase, homolog (yeast) | 0.2514 | 0.4451 | 0.691 | 7.0813 | 7.0159 | 6.9531 | 6.9531 | 6.8203 | 7.4434 | 6.7738 | 7.1166 | 6.9458 | 6.9209 | 7.1751 | 6.7515 |
| 202945_at | 2356 | FPGS | folylpolyglutamate synthase | 0.2496 | 0.4391 | 0.6849 | 7.3956 | 7.9685 | 7.5073 | 7.6867 | 7.7416 | 7.3551 | 7.5658 | 7.3028 | 6.9531 | 7.2595 | 7.913 | 7.7756 |
| 211715_s_at | 622 | BDH1 | 3-hydroxybutyrate dehydrogenase, type 1 | 0.2479 | 0.4271 | 0.6716 | 6.5793 | 6.6647 | 7.075 | 6.3782 | 6.2649 | 6.4672 | 5.8377 | 7.2841 | 5.73 | 6.4935 | 5.9184 | 6.8155 |
| 213995_at | 27109 | ATP5S | ATP synthase, H+ transporting, mitochondrial Fo complex, subunit s (factor B) | 0.2464 | 0.4351 | 0.6829 | 6.8717 | 6.8421 | 7.9299 | 8.402 | 8.181 | 8.0383 | 8.0238 | 6.9308 | 6.6115 | 6.7227 | 8.0335 | 7.9217 |
| 203582_s_at | 5867 | RAB4A | RAB4A, member RAS oncogene family | 0.2463 | 0.3872 | 0.6354 | 8.135 | 7.6791 | 7.9069 | 9.415 | 8.0559 | 9.3848 | 8.6794 | 7.937 | 7.1423 | 7.8976 | 7.7634 | 9.0242 |
| 201036_s_at | 3033 | HADH | hydroxyacyl-CoA dehydrogenase | 0.2435 | 0.479 | 0.7205 | 8.6398 | 8.6431 | 8.6827 | 7.5755 | 7.3551 | 8.4914 | 8.3092 | 7.3114 | 7.7545 | 8.6336 | 8.498 | 7.042 |
| 221482_s_at | 10776 | ARPP19 | cAMP-regulated phosphoprotein, 19kDa | 0.242 | 0.4711 | 0.7154 | 8.4241 | 8.3894 | 8.1665 | 8.2366 | 8.5389 | 8.2519 | 8.2553 | 8.5504 | 7.9993 | 8.1122 | 8.2553 | 8.3545 |
| 200989_at | 3091 | HIF1A | hypoxia inducible factor 1, alpha subunit (basic helix-loop-helix transcription factor) | 0.2408 | 0.483 | 0.7212 | 14.6935 | 14.5815 | 14.5815 | 14.5815 | 14.6935 | 14.6935 | 14.3206 | 14.6935 | 14.3206 | 14.3206 | 14.8479 | 14.8479 |
| 213324_at | 6714 | SRC | v-src sarcoma (Schmidt-Ruppin A-2) viral oncogene homolog (avian) | 0.2408 | 0.3932 | 0.6388 | 3.8792 | 3.9717 | 4.0273 | 7.1782 | 3.7012 | 8.1754 | 4.1498 | 3.7012 | 6.5385 | 4.0809 | 3.8267 | 4.2483 |
| 203303_at | 6990 | DYNLT3 | dynein, light chain, Tctex-type 3 | 0.2393 | 0.4571 | 0.704 | 7.7458 | 7.8257 | 7.816 | 8.51 | 7.7634 | 7.7145 | 8.3465 | 7.7756 | 7.9644 | 7.4527 | 7.3174 | 7.5375 |
| 208687_x_at | 3312 | HSPA8 | heat shock 70kDa protein 8 | 0.2388 | 0.7844 | 0.8879 | 10.6074 | 10.468 | 11.1208 | 10.8392 | 10.908 | 10.8826 | 10.5013 | 11.1592 | 8.1223 | 10.8392 | 10.972 | 11.2124 |
| 218249_at | 64429 | ZDHHC6 | zinc finger, DHHC-type containing 6 | 0.2383 | 0.493 | 0.7295 | 8.4093 | 4.2397 | 8.1852 | 8.3257 | 8.2999 | 8.402 | 3.9717 | 8.3894 | 7.7458 | 8.1908 | 3.6316 | 8.3092 |
| 205190_at | 5357 | PLS1 | plastin 1 | 0.2382 | 0.479 | 0.7205 | 7.8823 | 7.9031 | 8.1062 | 8.0259 | 7.8588 | 8.0294 | 7.6563 | 8.124 | 7.707 | 7.989 | 7.9335 | 7.994 |
| 204014_at | 1846 | DUSP4 | dual specificity phosphatase 4 | 0.2369 | 0.507 | 0.7389 | 8.2149 | 8.0684 | 8.6173 | 8.0559 | 7.8773 | 8.6465 | 8.2845 | 7.9299 | 7.3551 | 8.2523 | 8.1256 | 8.511 |
| 201412_at | 26020 | LRP10 | low density lipoprotein receptor-related protein 10 | 0.2366 | 0.519 | 0.7453 | 10.6137 | 10.8331 | 10.4396 | 10.249 | 11.2899 | 10.5568 | 10.6708 | 10.7003 | 10.1993 | 10.2889 | 10.6417 | 10.653 |
| 218902_at | 4851 | NOTCH1 | notch 1 | 0.235 | 0.6547 | 0.8188 | 11.3634 | 6.8435 | 7.1423 | 7.2099 | 7.1111 | 7.2869 | 7.3174 | 7.0974 | 7.3997 | 7.3114 | 7.4501 | 7.6653 |
| 218064_s_at | 26993 | AKAP8L | A kinase (PRKA) anchor protein 8-like | 0.2347 | 0.4212 | 0.6654 | 6.2346 | 6.9763 | 6.5918 | 7.1885 | 8.1574 | 6.4201 | 6.2812 | 7.8447 | 6.0363 | 6.7515 | 6.4148 | 6.3476 |
| 218474_s_at | 54442 | KCTD5 | potassium channel tetramerisation domain containing 5 | 0.2301 | 0.509 | 0.7397 | 6.5685 | 6.4672 | 6.3339 | 7.5237 | 6.4458 | 6.7086 | 6.5065 | 6.5147 | 6.2531 | 6.4826 | 6.6679 | 6.7764 |
| 209045_at | 7511 | XPNPEP1 | X-prolyl aminopeptidase (aminopeptidase P) 1, soluble | 0.2301 | 0.515 | 0.7453 | 8.4785 | 8.7786 | 8.4443 | 7.3997 | 8.3092 | 7.1111 | 8.2797 | 8.2196 | 8.2149 | 7.1423 | 7.6505 | 7.4236 |
| 201695_s_at | 4860 | PNP | purine nucleoside phosphorylase | 0.2294 | 0.489 | 0.7257 | 11.161 | 11.2718 | 11.1258 | 11.5278 | 11.7005 | 11.161 | 11.0778 | 11.1746 | 10.6659 | 11.2718 | 11.669 | 11.3122 |
| 201243_s_at | 481 | ATP1B1 | ATPase, Na+/K+ transporting, beta 1 polypeptide | 0.2258 | 0.5429 | 0.764 | 11.7005 | 11.8824 | 11.5032 | 11.5656 | 11.2124 | 11.3882 | 11.6793 | 11.7005 | 10.7777 | 11.1746 | 11.5382 | 11.5736 |
| 208644_at | 142 | PARP1 | poly (ADP-ribose) polymerase 1 | 0.2241 | 0.5868 | 0.7905 | 9.5336 | 9.7401 | 9.5676 | 9.5867 | 9.5193 | 9.5783 | 9.8248 | 9.5933 | 9.1559 | 9.3953 | 9.4319 | 9.6968 |
| 201959_s_at | 23077 | MYCBP2 | MYC binding protein 2 | 0.2221 | 0.4631 | 0.7076 | 7.2999 | 7.2468 | 7.1241 | 7.0661 | 7.513 | 7.1782 | 7.0585 | 7.3649 | 7.1002 | 7.072 | 7.0661 | 7.3569 |
| 208398_s_at | 9519 | TBPL1 | TBP-like 1 | 0.2198 | 0.5549 | 0.772 | 8.7665 | 8.8424 | 8.6336 | 8.8424 | 9.24 | 8.9854 | 8.9839 | 9.0166 | 8.582 | 8.7665 | 8.8501 | 8.5886 |
| 202413_s_at | 7398 | USP1 | ubiquitin specific peptidase 1 | 0.2191 | 0.501 | 0.7357 | 7.8091 | 7.3802 | 7.4254 | 7.2378 | 7.6511 | 7.042 | 6.9042 | 7.4661 | 7.6616 | 7.2502 | 7.4155 | 7.1323 |
| 213191_at | 148022 | TICAM1 | toll-like receptor adaptor molecule 1 | 0.2184 | 0.481 | 0.7205 | 5.663 | 6.1811 | 5.8969 | 5.7482 | 5.5606 | 5.9503 | 5.2868 | 5.6292 | 5.8629 | 5.7482 | 5.8782 | 5.9772 |
| 213233_s_at | 55958 | KLHL9 | kelch-like 9 (Drosophila) | 0.217 | 0.4671 | 0.7115 | 7.568 | 7.3174 | 7.3429 | 7.4967 | 9.2979 | 8.7304 | 7.7314 | 7.5056 | 7.6563 | 7.5885 | 7.4236 | 8.3294 |
| 221549_at | 83743 | GRWD1 | glutamate-rich WD repeat containing 1 | 0.2162 | 0.499 | 0.7339 | 6.9731 | 7.2382 | 6.868 | 7.3752 | 6.7449 | 7.2099 | 7.072 | 6.729 | 6.9084 | 7.2386 | 7.0159 | 6.9042 |
| 201334_s_at | 23365 | ARHGEF12 | Rho guanine nucleotide exchange factor (GEF) 12 | 0.2156 | 0.497 | 0.732 | 7.0988 | 7.1111 | 6.7367 | 7.0974 | 6.3593 | 6.9531 | 6.3826 | 7.0355 | 6.7227 | 6.4935 | 6.9747 | 6.9988 |
| 201579_at | 2195 | FAT1 | FAT tumor suppressor homolog 1 (Drosophila) | 0.2121 | 0.5369 | 0.7577 | 9.1348 | 9.0651 | 8.7883 | 9.1881 | 9.3073 | 9.0957 | 8.8898 | 9.1559 | 8.7631 | 9.1075 | 8.9522 | 9.2559 |
| 212689_s_at | 55818 | KDM3A | lysine (K)-specific demethylase 3A | 0.2116 | 0.8104 | 0.9017 | 8.9263 | 8.7978 | 8.7978 | 8.6715 | 5.6421 | 9.1936 | 8.0363 | 8.3782 | 8.5264 | 8.5681 | 4.5854 | 8.2736 |
| 201323_at | 10969 | EBNA1BP2 | EBNA1 binding protein 2 | 0.2114 | 0.481 | 0.7205 | 12.6867 | 12.2931 | 12.4808 | 12.4808 | 12.3797 | 12.1519 | 12.3655 | 12.5912 | 12.1784 | 12.3115 | 12.417 | 12.1784 |
| 209337_at | 11168 | PSIP1 | PC4 and SFRS1 interacting protein 1 | 0.2054 | 0.5609 | 0.7737 | 9.8854 | 9.5783 | 6.6585 | 10.1514 | 9.7268 | 9.7401 | 6.3108 | 9.9633 | 9.5676 | 9.8994 | 9.7859 | 6.4074 |
| 218245_at | 25987 | TSKU | tsukushi small leucine rich proteoglycan homolog (Xenopus laevis) | 0.2048 | 0.507 | 0.7389 | 5.9924 | 5.9718 | 5.8199 | 6.0558 | 6.1811 | 6.1811 | 5.8163 | 6.0209 | 5.7865 | 5.8377 | 6.2812 | 6.0558 |
| 219492_at | 26511 | CHIC2 | cysteine-rich hydrophobic domain 2 | 0.2041 | 0.6008 | 0.8016 | 7.9732 | 7.7356 | 7.7657 | 7.7268 | 8.2329 | 4.6715 | 7.708 | 5.5738 | 7.4434 | 7.716 | 8.0069 | 4.1186 |
| 212143_s_at | 3486 | IGFBP3 | insulin-like growth factor binding protein 3 | 0.2039 | 0.9321 | 0.9637 | 8.9937 | 8.8705 | 8.9293 | 8.9527 | 9.1707 | 9.0276 | 6.9657 | 9.3614 | 8.7568 | 9.1754 | 9.3361 | 9.1012 |
| 219281_at | 4482 | MSRA | methionine sulfoxide reductase A | 0.2034 | 0.5389 | 0.7595 | 8.0069 | 8.1545 | 7.8773 | 8.009 | 7.2245 | 7.1914 | 8.3782 | 7.2291 | 6.9655 | 8.0423 | 7.0936 | 7.5507 |
| 202006_at | 5782 | PTPN12 | protein tyrosine phosphatase, non-receptor type 12 | 0.2025 | 0.519 | 0.7453 | 9.7514 | 9.4486 | 9.3255 | 9.4705 | 9.684 | 9.4631 | 9.0374 | 9.7626 | 9.0922 | 9.3037 | 9.7963 | 9.5548 |
| 206099_at | 5583 | PRKCH | protein kinase C, eta | 0.1967 | 0.6287 | 0.8112 | 4.8322 | 4.7967 | 4.5382 | 4.9889 | 4.7256 | 4.841 | 4.6966 | 4.6715 | 4.9216 | 4.9134 | 4.7103 | 4.4114 |
| 201833_at | 3066 | HDAC2 | histone deacetylase 2 | 0.1965 | 0.5269 | 0.7502 | 10.0766 | 10.0604 | 9.8994 | 10.212 | 9.8854 | 9.9902 | 9.8403 | 10.0145 | 9.6129 | 9.936 | 10.042 | 10.2737 |
| 208950_s_at | 501 | ALDH7A1 | aldehyde dehydrogenase 7 family, member A1 | 0.1957 | 0.505 | 0.7389 | 7.1801 | 6.9458 | 7.1423 | 7.9771 | 7.1751 | 6.5559 | 6.5948 | 6.8753 | 7.798 | 7.156 | 6.855 | 6.6264 |
| 202887_s_at | 54541 | DDIT4 | DNA-damage-inducible transcript 4 | 0.1933 | 0.6148 | 0.8092 | 7.0355 | 7.7268 | 7.7832 | 8.2824 | 8.1169 | 7.9299 | 7.8447 | 6.7388 | 8.3294 | 8.0653 | 7.1111 | 7.5894 |
| 206571_s_at | 9448 | MAP4K4 | mitogen-activated protein kinase kinase kinase kinase 4 | 0.1913 | 0.6208 | 0.8095 | 8.7161 | 8.6205 | 8.2348 | 8.5852 | 8.6336 | 8.2348 | 8.582 | 8.4749 | 7.8184 | 8.3198 | 8.8131 | 8.3903 |
| 204514_at | 1802 | DPH2 | DPH2 homolog (S. cerevisiae) | 0.1906 | 0.5469 | 0.7652 | 7.0988 | 7.088 | 7.1782 | 7.0661 | 6.5104 | 7.241 | 6.5685 | 7.3551 | 6.5887 | 7.072 | 6.9655 | 6.988 |
| 204610_s_at | 11007 | CCDC85B | coiled-coil domain containing 85B | 0.1896 | 0.517 | 0.7453 | 6.5529 | 7.3707 | 7.088 | 7.3385 | 6.599 | 6.8292 | 6.5529 | 6.8421 | 6.3496 | 6.4096 | 7.1751 | 7.5154 |
| 200843_s_at | 2058 | EPRS | glutamyl-prolyl-tRNA synthetase | 0.1854 | 0.517 | 0.7453 | 10.879 | 10.5568 | 10.7254 | 10.737 | 10.4304 | 10.7641 | 10.5491 | 10.879 | 10.448 | 10.3719 | 10.7777 | 10.6708 |
| 209682_at | 868 | CBLB | Cas-Br-M (murine) ecotropic retroviral transforming sequence b | 0.1854 | 0.5449 | 0.7646 | 7.6047 | 7.3419 | 7.8858 | 7.3419 | 7.2549 | 7.8588 | 7.1751 | 7.1596 | 7.1423 | 8.1196 | 7.3854 | 7.5755 |
| 204808_s_at | 10329 | TMEM5 | transmembrane protein 5 | 0.1853 | 0.5808 | 0.789 | 10.2533 | 10.459 | 9.6102 | 9.8804 | 9.7963 | 9.631 | 10.3066 | 9.4631 | 9.1224 | 9.5062 | 10.653 | 9.5469 |
| 203616_at | 5423 | POLB | polymerase (DNA directed), beta | 0.1837 | 0.6886 | 0.8397 | 6.599 | 6.7764 | 6.7449 | 6.8753 | 7.0095 | 7.1323 | 6.0558 | 6.8837 | 6.8753 | 6.741 | 6.8018 | 7.1596 |
| 200711_s_at | 6500 | SKP1 | S-phase kinase-associated protein 1 | 0.1826 | 0.495 | 0.7302 | 7.5322 | 7.7129 | 7.5971 | 7.5461 | 7.2869 | 7.8823 | 7.2378 | 7.8578 | 7.0813 | 7.509 | 7.3956 | 7.8976 |
| 209662_at | 1070 | CETN3 | centrin, EF-hand protein, 3 | 0.1793 | 0.6547 | 0.8188 | 8.3407 | 8.3263 | 8.3102 | 8.4548 | 8.1754 | 8.443 | 8.3278 | 8.4293 | 8.1404 | 7.8447 | 8.4914 | 8.4418 |
| 210115_at | 116832 | RPL39L | ribosomal protein L39-like | 0.1781 | 0.5469 | 0.7652 | 6.3383 | 6.3108 | 6.9943 | 7.4186 | 6.34 | 7.168 | 6.2018 | 6.4201 | 5.4961 | 6.9531 | 7.3609 | 6.9042 |
| 202778_s_at | 7750 | ZMYM2 | zinc finger, MYM-type 2 | 0.1772 | 0.5589 | 0.7731 | 6.7515 | 6.5008 | 6.3476 | 6.5842 | 6.8975 | 6.5793 | 6.5745 | 6.9531 | 6.0903 | 6.5745 | 6.5842 | 6.3782 |
| 202598_at | 6284 | S100A13 | S100 calcium binding protein A13 | 0.175 | 0.5788 | 0.7874 | 12.0869 | 12.3655 | 12.3655 | 12.7014 | 12.5073 | 12.1267 | 12.1907 | 12.3797 | 12.1267 | 12.4808 | 12.3315 | 12.2657 |
| 208936_x_at | 3964 | LGALS8 | lectin, galactoside-binding, soluble, 8 | 0.1737 | 0.5449 | 0.7646 | 8.0559 | 8.3782 | 8.2527 | 8.7071 | 7.5375 | 8.0559 | 7.8136 | 8.6794 | 7.8894 | 7.7335 | 7.9707 | 8.135 |
| 209029_at | 50813 | COPS7A | COP9 constitutive photomorphogenic homolog subunit 7A (Arabidopsis) | 0.1732 | 0.6208 | 0.8095 | 10.7393 | 11.0165 | 11.0993 | 11.0054 | 10.879 | 12.015 | 11.1857 | 11.1208 | 11.0563 | 10.8911 | 11.0778 | 10.7988 |
| 209189_at | 2353 | FOS | FBJ murine osteosarcoma viral oncogene homolog | 0.1719 | 0.5968 | 0.7985 | 7.0111 | 6.7713 | 6.7203 | 6.9209 | 6.4935 | 6.0639 | 7.197 | 6.0903 | 6.9084 | 6.8428 | 5.9994 | 6.0438 |
| 218108_at | 55148 | UBR7 | ubiquitin protein ligase E3 component n-recognin 7 (putative) | 0.1718 | 0.5589 | 0.7731 | 9.8994 | 9.9558 | 8.893 | 9.0276 | 8.7978 | 10.0766 | 9.0518 | 10.0604 | 8.6173 | 9.7275 | 8.9532 | 9.0741 |
| 219711_at | 54807 | ZNF586 | zinc finger protein 586 | 0.1706 | 0.7086 | 0.8461 | 5.0402 | 5.2953 | 5.3955 | 5.4779 | 11.3002 | 4.841 | 5.9772 | 5.2953 | 6.3339 | 5.6765 | 5.5976 | 5.5373 |
| 209191_at | 84617 | TUBB6 | tubulin, beta 6 | 0.1696 | 0.6267 | 0.8108 | 12.7437 | 5.5916 | 12.7437 | 5.5478 | 12.6305 | 12.5073 | 5.73 | 5.5542 | 12.4808 | 5.0861 | 12.7664 | 12.4521 |
| 212054_x_at | 23061 | TBC1D9B | TBC1 domain family, member 9B (with GRAM domain) | 0.1693 | 0.5669 | 0.7797 | 6.6943 | 6.8533 | 7.1885 | 7.5237 | 6.6738 | 6.7698 | 7.3439 | 6.6561 | 6.3957 | 6.2589 | 7.6517 | 6.4795 |
| 217901_at | 1829 | DSG2 | desmoglein 2 | 0.1675 | 0.6487 | 0.8184 | 8.2999 | 8.6279 | 8.2348 | 8.4824 | 8.2638 | 8.3782 | 9.3288 | 8.1665 | 8.0684 | 8.0047 | 8.0684 | 7.9728 |
| 202388_at | 5997 | RGS2 | regulator of G-protein signaling 2, 24kDa | 0.1674 | 0.5828 | 0.7905 | 6.6943 | 7.913 | 7.7844 | 6.7388 | 6.6738 | 8.0653 | 8.0155 | 7.8091 | 6.2805 | 6.9042 | 6.5918 | 6.9042 |
| 204761_at | 9712 | USP6NL | USP6 N-terminal like | 0.1671 | 0.6846 | 0.8391 | 7.0691 | 12.0613 | 7.7356 | 7.0585 | 7.2099 | 7.2245 | 7.5108 | 7.8329 | 7.5971 | 7.5108 | 7.0781 | 8.4056 |
| 209253_at | 10174 | SORBS3 | sorbin and SH3 domain containing 3 | 0.1669 | 0.6088 | 0.8072 | 4.2483 | 8.5475 | 4.2483 | 4.231 | 7.1801 | 4.231 | 4.4456 | 4.3527 | 4.4949 | 4.3213 | 7.6122 | 4.2043 |
| 201693_s_at | 1958 | EGR1 | early growth response 1 | 0.1667 | 0.5749 | 0.7863 | 6.6478 | 6.7086 | 5.7377 | 6.3782 | 6.0709 | 6.2618 | 6.0903 | 6.6647 | 5.8306 | 5.9924 | 6.5498 | 5.9718 |
| 201469_s_at | 6464 | SHC1 | SHC (Src homology 2 domain containing) transforming protein 1 | 0.1662 | 0.6427 | 0.8163 | 9.2281 | 9.5062 | 9.4825 | 9.3913 | 11.0282 | 9.0769 | 10.1251 | 8.7702 | 9.6634 | 9.6634 | 9.18 | 9.1181 |
| 212815_at | 10973 | ASCC3 | activating signal cointegrator 1 complex subunit 3 | 0.1649 | 0.5549 | 0.772 | 7.8483 | 7.4434 | 7.5658 | 7.852 | 7.6635 | 7.513 | 7.2156 | 8.0719 | 7.2619 | 7.5158 | 7.5461 | 7.7844 |
| 207416_s_at | 4775 | NFATC3 | nuclear factor of activated T-cells, cytoplasmic, calcineurin-dependent 3 | 0.1646 | 0.6727 | 0.8296 | 6.0798 | 6.1558 | 10.9477 | 5.8888 | 6.1674 | 5.8969 | 6.4795 | 6.2649 | 6.6796 | 6.661 | 6.3908 | 6.5208 |
| 219222_at | 64080 | RBKS | ribokinase | 0.1637 | 0.5788 | 0.7874 | 5.4497 | 5.3313 | 5.0447 | 5.5916 | 4.9409 | 4.9736 | 5.2594 | 5.251 | 5.0557 | 5.1287 | 5.2205 | 5.0557 |
| 211783_s_at | 9112 | MTA1 | metastasis associated 1 | 0.163 | 0.6487 | 0.8184 | 7.6122 | 8.2771 | 8.1338 | 8.7769 | 8.0939 | 7.6906 | 8.1223 | 7.6505 | 7.9809 | 7.6517 | 8.4362 | 8.0363 |
| 205379_at | 874 | CBR3 | carbonyl reductase 3 | 0.1621 | 0.6267 | 0.8108 | 8.4583 | 8.609 | 8.4418 | 8.3407 | 8.2903 | 8.5834 | 8.5237 | 8.3257 | 8.1893 | 8.4014 | 8.6431 | 8.3629 |
| 201997_s_at | 23013 | SPEN | spen homolog, transcriptional regulator (Drosophila) | 0.1617 | 0.6367 | 0.8151 | 7.4661 | 7.5158 | 7.2595 | 7.437 | 8.7093 | 7.2607 | 8.3587 | 7.4743 | 7.0192 | 7.2434 | 7.5831 | 6.9347 |
| 217761_at | 55256 | ADI1 | acireductone dioxygenase 1 | 0.1607 | 0.9721 | 0.9852 | 9.0717 | 8.8501 | 8.7434 | 8.7006 | 8.7665 | 8.4548 | 8.9674 | 6.9042 | 8.6684 | 8.7117 | 9.2702 | 9.045 |
| 218175_at | 80212 | CCDC92 | coiled-coil domain containing 92 | 0.16 | 0.7166 | 0.8511 | 7.2468 | 7.2382 | 7.0126 | 6.8475 | 7.3089 | 7.0608 | 6.8804 | 7.0095 | 6.4611 | 7.209 | 7.2099 | 7.4474 |
| 201591_s_at | 11188 | NISCH | nischarin | 0.1582 | 0.5948 | 0.7969 | 6.3631 | 7.0033 | 6.6869 | 6.9387 | 6.9125 | 6.7353 | 6.5426 | 6.9943 | 6.2618 | 6.3361 | 7.2291 | 6.6943 |
| 202954_at | 11065 | UBE2C | ubiquitin-conjugating enzyme E2C | 0.1571 | 0.5768 | 0.7868 | 12.0242 | 12.1519 | 12.2931 | 12.3315 | 12.3115 | 12.5912 | 12.3315 | 12.5651 | 11.9142 | 12.1907 | 12.1907 | 12.1267 |
| 203038_at | 5796 | PTPRK | protein tyrosine phosphatase, receptor type, K | 0.1552 | 0.8483 | 0.929 | 10.4535 | 10.5491 | 10.7417 | 10.7777 | 10.3917 | 11.0165 | 11.0165 | 10.9477 | 6.4672 | 11.1393 | 10.879 | 11.4922 |
| 217931_at | 10695 | CNPY3 | canopy 3 homolog (zebrafish) | 0.1551 | 0.7046 | 0.8461 | 6.8515 | 6.8095 | 7.4402 | 7.1638 | 12.5651 | 7.9171 | 7.5831 | 7.3419 | 8.4418 | 7.479 | 7.6122 | 7.863 |
| 204748_at | 5743 | PTGS2 | prostaglandin-endoperoxide synthase 2 (prostaglandin G/H synthase and cyclooxygenase) | 0.1534 | 0.6527 | 0.8184 | 4.0602 | 4.0151 | 3.8267 | 3.7758 | 3.6316 | 3.6316 | 3.8267 | 3.5043 | 4.2725 | 3.5043 | 3.7012 | 3.7012 |
| 204087_s_at | 8884 | SLC5A6 | solute carrier family 5 (sodium-dependent vitamin transporter), member 6 | 0.1532 | 0.6208 | 0.8095 | 7.632 | 7.1596 | 7.6505 | 8.7304 | 8.6061 | 7.1423 | 7.0126 | 7.6467 | 8.2329 | 7.3956 | 8.5091 | 6.887 |
| 202011_at | 7082 | TJP1 | tight junction protein 1 (zona occludens 1) | 0.1523 | 0.6607 | 0.821 | 7.0813 | 6.6869 | 7.0064 | 7.3828 | 7.0033 | 7.278 | 6.8717 | 6.9125 | 6.992 | 6.4672 | 7.6563 | 6.9657 |
| 205463_s_at | 5154 | PDGFA | platelet-derived growth factor alpha polypeptide | 0.1523 | 0.6527 | 0.8184 | 9.0957 | 8.4362 | 9.2928 | 8.5237 | 9.415 | 8.5534 | 8.0559 | 8.5389 | 8.6684 | 9.0602 | 8.9674 | 9.24 |
| 210058_at | 5603 | MAPK13 | mitogen-activated protein kinase 13 | 0.1514 | 0.6667 | 0.8264 | 7.6467 | 7.209 | 7.5108 | 7.5151 | 7.8204 | 7.1914 | 8.8963 | 7.1029 | 6.6647 | 6.8106 | 7.5108 | 6.9347 |
| 209364_at | 572 | BAD | BCL2-associated agonist of cell death | 0.1498 | 0.6527 | 0.8184 | 7.4568 | 7.8894 | 7.8091 | 7.8588 | 8.8343 | 7.7386 | 8.1327 | 7.479 | 7.6144 | 7.6122 | 7.706 | 8.3198 |
| 203931_s_at | 6182 | MRPL12 | mitochondrial ribosomal protein L12 | 0.146 | 0.5948 | 0.7969 | 10.7843 | 11.5857 | 11.4145 | 11.451 | 11.4384 | 11.8403 | 10.923 | 11.3882 | 10.8911 | 11.3634 | 11.4259 | 11.8922 |
| 201494_at | 5547 | PRCP | prolylcarboxypeptidase (angiotensinase C) | 0.1453 | 0.8124 | 0.9028 | 8.2444 | 8.4075 | 8.3629 | 8.1415 | 8.1992 | 8.2999 | 8.2369 | 8.3407 | 8.4362 | 8.206 | 8.2999 | 7.8823 |
| 202329_at | 1445 | CSK | c-src tyrosine kinase | 0.1416 | 0.6527 | 0.8184 | 7.2869 | 7.5073 | 7.2245 | 7.4877 | 7.829 | 7.509 | 6.9943 | 7.2099 | 6.8421 | 8.5475 | 7.1801 | 7.3707 |
| 203138_at | 8520 | HAT1 | histone acetyltransferase 1 | 0.1412 | 0.6786 | 0.8349 | 10.0849 | 9.8403 | 9.5469 | 9.1348 | 9.3502 | 9.4486 | 9.8878 | 9.18 | 9.9918 | 9.3635 | 9.4462 | 8.8898 |
| 202990_at | 5836 | PYGL | phosphorylase, glycogen, liver | 0.1407 | 0.7046 | 0.8461 | 9.2787 | 9.2894 | 9.7401 | 9.8141 | 9.8248 | 9.6671 | 9.5801 | 9.4187 | 9.388 | 8.8343 | 9.9273 | 9.9103 |
| 204826_at | 899 | CCNF | cyclin F | 0.1402 | 0.6886 | 0.8397 | 7.0198 | 7.042 | 6.8106 | 6.8615 | 9.0651 | 6.6561 | 6.9655 | 7.1801 | 6.1255 | 7.2747 | 7.5894 | 7.1423 |
| 203132_at | 5925 | RB1 | retinoblastoma 1 | 0.1361 | 0.7305 | 0.8546 | 7.6616 | 7.6653 | 7.2245 | 7.7599 | 7.5614 | 7.3678 | 7.7129 | 7.2595 | 8.1106 | 7.6505 | 7.088 | 6.8804 |
| 209507_at | 6119 | RPA3 | replication protein A3, 14kDa | 0.1358 | 0.6507 | 0.8184 | 8.5834 | 7.716 | 7.716 | 8.4418 | 7.4661 | 7.6144 | 8.4824 | 8.2845 | 7.7998 | 7.042 | 7.3439 | 7.7599 |
| 221230_s_at | 51742 | ARID4B | AT rich interactive domain 4B (RBP1-like) | 0.1328 | 0.7006 | 0.8461 | 7.7458 | 7.5484 | 8.2149 | 7.3649 | 7.9194 | 7.6047 | 7.3439 | 7.4254 | 7.7268 | 7.1944 | 8.1223 | 8.0403 |
| 202318_s_at | 26054 | SENP6 | SUMO1/sentrin specific peptidase 6 | 0.1306 | 0.6607 | 0.821 | 8.9134 | 8.7838 | 8.9102 | 8.9527 | 8.5264 | 8.9175 | 8.609 | 8.8343 | 8.7786 | 8.7598 | 8.6279 | 9.1224 |
| 202794_at | 3628 | INPP1 | inositol polyphosphate-1-phosphatase | 0.1302 | 0.6687 | 0.8278 | 9.2894 | 9.0485 | 9.1101 | 8.8807 | 8.9323 | 8.8039 | 8.9937 | 8.6715 | 8.6505 | 8.9175 | 8.9824 | 9.4775 |
| 203627_at | 3480 | IGF1R | insulin-like growth factor 1 receptor | 0.1225 | 0.6347 | 0.8147 | 9.0099 | 8.7093 | 8.4698 | 7.6505 | 7.4617 | 8.3872 | 8.2999 | 8.7786 | 7.3854 | 8.3092 | 7.6947 | 8.4014 |
| 205691_at | 9143 | SYNGR3 | synaptogyrin 3 | 0.119 | 0.7126 | 0.8488 | 4.1186 | 3.4175 | 3.6316 | 3.8267 | 3.7758 | 4.1776 | 3.6664 | 3.2386 | 3.9934 | 3.4175 | 4.1498 | 4.0151 |
| 201314_at | 10494 | STK25 | serine/threonine kinase 25 | 0.1181 | 0.7146 | 0.8502 | 9.6968 | 10.0183 | 10.159 | 10.4861 | 10.212 | 10.1028 | 9.8804 | 10.1251 | 9.804 | 10.1154 | 10.0527 | 10.3719 |
| 202518_at | 9275 | BCL7B | B-cell CLL/lymphoma 7B | 0.1158 | 0.7066 | 0.8461 | 6.5208 | 6.5887 | 6.4096 | 6.2018 | 6.2812 | 6.5462 | 5.8377 | 6.6365 | 6.256 | 6.1777 | 6.4733 | 6.8155 |
| 202332_at | 1454 | CSNK1E | casein kinase 1, epsilon | 0.1147 | 0.7685 | 0.8811 | 9.4631 | 9.459 | 9.3589 | 9.3137 | 9.6104 | 9.415 | 9.5336 | 9.0242 | 9.4631 | 9.4948 | 9.1224 | 9.7275 |
| 221552_at | 57406 | ABHD6 | abhydrolase domain containing 6 | 0.1128 | 0.6128 | 0.8088 | 10.5491 | 10.5013 | 6.868 | 7.0095 | 7.0126 | 7.0204 | 7.4155 | 10.7988 | 7.7129 | 7.0192 | 6.7706 | 6.9763 |
| 203640_at | 10150 | MBNL2 | muscleblind-like 2 (Drosophila) | 0.1079 | 0.8942 | 0.9529 | 9.5013 | 9.8499 | 9.153 | 8.8898 | 9.5801 | 9.7268 | 9.3635 | 9.7963 | 7.95 | 9.5548 | 9.6882 | 9.6634 |
| 212723_at | 23210 | JMJD6 | jumonji domain containing 6 | 0.1072 | 0.7265 | 0.854 | 11.9623 | 11.8741 | 11.5736 | 11.5185 | 11.8741 | 11.7779 | 11.2899 | 12.015 | 11.3254 | 11.7058 | 11.9552 | 11.9623 |
| 201855_s_at | 23300 | ATMIN | ATM interactor | 0.1067 | 0.7505 | 0.8666 | 7.4693 | 7.3114 | 7.0974 | 7.3047 | 6.7353 | 6.9209 | 7.0192 | 7.4218 | 6.492 | 6.988 | 7.1885 | 7.3402 |
| 212189_s_at | 25839 | COG4 | component of oligomeric golgi complex 4 | 0.1064 | 0.7186 | 0.8511 | 7.2386 | 7.9809 | 7.6989 | 8.0363 | 7.1524 | 7.6719 | 7.6122 | 7.267 | 7.3752 | 7.6251 | 7.8568 | 7.6719 |
| 203117_s_at | 9924 | PAN2 | PAN2 poly(A) specific ribonuclease subunit homolog (S. cerevisiae) | 0.1012 | 0.7445 | 0.8637 | 3.9717 | 4.5382 | 3.5043 | 3.8792 | 5.2205 | 4.5947 | 3.9016 | 3.8267 | 3.4175 | 3.7012 | 5.0557 | 5.005 |
| 204131_s_at | 2309 | FOXO3 | forkhead box O3 | 0.1006 | 0.7345 | 0.8562 | 7.7942 | 7.0631 | 6.7698 | 7.6989 | 7.5011 | 7.241 | 6.5887 | 6.8292 | 7.2468 | 7.5375 | 7.6947 | 7.6563 |
| 208847_s_at | 128 | ADH5 | alcohol dehydrogenase 5 (class III), chi polypeptide | 0.09974 | 0.7665 | 0.8798 | 10.5209 | 10.5734 | 10.5013 | 10.6074 | 10.7641 | 10.459 | 10.0766 | 10.972 | 9.9424 | 10.7254 | 10.7157 | 10.6837 |
| 218679_s_at | 51160 | VPS28 | vacuolar protein sorting 28 homolog (S. cerevisiae) | 0.09916 | 0.7186 | 0.8511 | 6.5084 | 7.707 | 6.3826 | 6.5104 | 7.3429 | 7.1241 | 6.1605 | 7.6635 | 7.3385 | 7.5507 | 6.0314 | 6.0338 |
| 203848_at | 10270 | AKAP8 | A kinase (PRKA) anchor protein 8 | 0.09895 | 0.7086 | 0.8461 | 7.6505 | 7.5348 | 7.3649 | 7.9529 | 8.0306 | 7.7961 | 7.3707 | 7.5151 | 7.2099 | 7.8588 | 8.1958 | 7.8136 |
| 212871_at | 8550 | MAPKAPK5 | mitogen-activated protein kinase-activated protein kinase 5 | 0.09831 | 0.7066 | 0.8461 | 8.0444 | 7.1885 | 7.3114 | 7.8894 | 7.3429 | 8.4824 | 6.9125 | 7.6754 | 7.4877 | 7.3569 | 7.9604 | 8.2824 |
| 201200_at | 8804 | CREG1 | cellular repressor of E1A-stimulated genes 1 | 0.09818 | 0.7246 | 0.8537 | 9.2709 | 10.653 | 9.6384 | 10.7417 | 10.2689 | 10.468 | 9.4825 | 10.3006 | 10.7254 | 9.6482 | 10.448 | 9.7963 |
| 200915_x_at | 3895 | KTN1 | kinectin 1 (kinesin receptor) | 0.09682 | 0.7665 | 0.8798 | 11.1114 | 11.0534 | 10.6417 | 10.3818 | 10.5324 | 10.4304 | 10.7157 | 10.7843 | 10.9278 | 10.459 | 10.3917 | 10.5704 |
| 204756_at | 5607 | MAP2K5 | mitogen-activated protein kinase kinase 5 | 0.09329 | 0.7585 | 0.8737 | 7.3028 | 7.356 | 7.1166 | 7.1002 | 7.3649 | 7.5484 | 7.2747 | 7.2245 | 7.4434 | 7.1373 | 7.0159 | 7.4967 |
| 203192_at | 10058 | ABCB6 | ATP-binding cassette, sub-family B (MDR/TAP), member 6 | 0.09217 | 0.6966 | 0.8453 | 7.6906 | 7.9217 | 7.6101 | 8.3782 | 7.2386 | 8.0487 | 7.4527 | 7.373 | 7.4693 | 7.8773 | 8.1393 | 8.1602 |
| 202123_s_at | 25 | ABL1 | c-abl oncogene 1, non-receptor tyrosine kinase | 0.09102 | 0.7485 | 0.8653 | 7.5755 | 8.8807 | 7.8976 | 8.6652 | 8.7727 | 8.1196 | 7.5158 | 9.24 | 8.2771 | 7.6122 | 8.8761 | 7.706 |
| 204825_at | 9833 | MELK | maternal embryonic leucine zipper kinase | 0.09008 | 0.7705 | 0.8823 | 11.2558 | 11.1114 | 11.3906 | 10.8911 | 10.8639 | 12.3115 | 10.879 | 11.0778 | 10.6815 | 12.1267 | 11.3002 | 11.1997 |
| 214710_s_at | 891 | CCNB1 | cyclin B1 | 0.08871 | 0.7305 | 0.8546 | 12.8415 | 12.5073 | 8.2553 | 12.2931 | 12.4808 | 12.417 | 12.983 | 12.7892 | 8.7027 | 13.0802 | 12.9116 | 8.1545 |
| 204484_at | 5287 | PIK3C2B | phosphoinositide-3-kinase, class 2, beta polypeptide | 0.08582 | 0.7964 | 0.8942 | 5.7924 | 5.9772 | 5.338 | 4.3843 | 5.6765 | 4.0602 | 4.4949 | 5.7626 | 4.4625 | 5.9637 | 5.424 | 4.337 |
| 208690_s_at | 9124 | PDLIM1 | PDZ and LIM domain 1 | 0.08347 | 0.7345 | 0.8562 | 8.941 | 7.7942 | 8.2999 | 8.1602 | 9.1909 | 7.9848 | 8.0774 | 8.0306 | 8.5757 | 8.5629 | 7.6754 | 8.941 |
| 212973_at | 22934 | RPIA | ribose 5-phosphate isomerase A | 0.08197 | 0.9102 | 0.9541 | 10.1959 | 10.365 | 10.232 | 10.5073 | 10.468 | 10.2689 | 10.5073 | 10.5568 | 9.6721 | 10.1993 | 10.5815 | 10.2876 |
| 209608_s_at | 39 | ACAT2 | acetyl-CoA acetyltransferase 2 | 0.08172 | 0.8303 | 0.9166 | 10.8826 | 11.161 | 10.9182 | 5.8199 | 10.9477 | 10.8639 | 9.6968 | 10.0323 | 10.1926 | 9.7541 | 9.8338 | 9.9633 |
| 209860_s_at | 310 | ANXA7 | annexin A7 | 0.07899 | 0.7884 | 0.8883 | 10.0604 | 9.8644 | 10.1849 | 10.435 | 10.1993 | 10.1486 | 10.0527 | 10.2689 | 9.6671 | 10.0766 | 10.1666 | 10.448 |
| 202468_s_at | 8727 | CTNNAL1 | catenin (cadherin-associated protein), alpha-like 1 | 0.07651 | 0.7745 | 0.8838 | 9.9918 | 9.1012 | 8.9674 | 8.7769 | 8.9937 | 8.9824 | 9.5952 | 9.7119 | 8.4293 | 8.3894 | 8.7838 | 9.4319 |
| 203570_at | 4016 | LOXL1 | lysyl oxidase-like 1 | 0.07526 | 0.8423 | 0.9256 | 8.7978 | 10.6074 | 10.2889 | 9.1633 | 10.5765 | 10.2447 | 10.212 | 10.2533 | 9.644 | 9.9424 | 9.6102 | 9.5248 |
| 213384_x_at | 5331 | PLCB3 | phospholipase C, beta 3 (phosphatidylinositol-specific) | 0.07352 | 0.7725 | 0.8836 | 7.829 | 7.8823 | 7.9529 | 8.2444 | 7.9732 | 8.2348 | 7.9529 | 7.9514 | 7.829 | 7.7942 | 8.1545 | 8.2736 |
| 218826_at | 54733 | SLC35F2 | solute carrier family 35, member F2 | 0.07134 | 0.8623 | 0.9349 | 8.2553 | 8.1223 | 7.5316 | 8.5681 | 8.351 | 7.1057 | 8.009 | 7.9848 | 7.9069 | 7.0631 | 8.3045 | 8.2369 |
| 212453_at | 26128 | KIAA1279 | KIAA1279 | 0.0709 | 0.8463 | 0.9279 | 8.8986 | 9.2559 | 7.6906 | 7.654 | 9.1834 | 7.8751 | 7.2619 | 7.7572 | 8.5346 | 8.5757 | 9.0083 | 8.8085 |
| 202659_at | 5699 | PSMB10 | proteasome (prosome, macropain) subunit, beta type, 10 | 0.07034 | 0.9701 | 0.9841 | 7.9707 | 7.863 | 7.7998 | 7.829 | 7.7844 | 7.8588 | 7.6486 | 7.0192 | 8.0383 | 8.2102 | 8.2903 | 7.6719 |
| 218104_at | 54881 | TEX10 | testis expressed 10 | 0.06804 | 0.8723 | 0.9405 | 7.8729 | 7.5348 | 7.5538 | 7.7832 | 7.6144 | 7.6517 | 7.6684 | 7.716 | 7.2813 | 7.6412 | 7.8329 | 7.7386 |
| 203304_at | 25805 | BAMBI | BMP and activin membrane-bound inhibitor homolog (Xenopus laevis) | 0.0665 | 0.8583 | 0.9327 | 6.1674 | 5.9854 | 5.9556 | 6.2812 | 6.4322 | 6.8475 | 7.3884 | 5.694 | 6.0589 | 5.7377 | 6.4951 | 5.9059 |
| 212140_at | 23244 | PDS5A | PDS5, regulator of cohesion maintenance, homolog A (S. cerevisiae) | 0.06626 | 0.7764 | 0.884 | 9.331 | 9.1881 | 9.148 | 9.6721 | 9.0829 | 9.0888 | 9.1737 | 9.3848 | 9.2894 | 8.8501 | 9.1881 | 9.451 |
| 218597_s_at | 55847 | CISD1 | CDGSH iron sulfur domain 1 | 0.06572 | 0.7884 | 0.8883 | 12.3655 | 11.6793 | 11.451 | 11.6213 | 11.0563 | 11.5278 | 11.6464 | 11.3401 | 11.7005 | 11.3882 | 11.7221 | 11.669 |
| 201339_s_at | 6342 | SCP2 | sterol carrier protein 2 | 0.06457 | 0.7844 | 0.8879 | 7.6219 | 8.0069 | 7.5876 | 7.3828 | 8.2587 | 7.6563 | 7.4693 | 8.0884 | 6.9763 | 7.2245 | 8.3257 | 8.0939 |
| 205909_at | 5427 | POLE2 | polymerase (DNA directed), epsilon 2 (p59 subunit) | 0.06327 | 0.8084 | 0.9017 | 8.7006 | 9.1834 | 8.7752 | 8.8705 | 8.5091 | 9.3465 | 8.5303 | 8.402 | 8.9674 | 8.82 | 9.1936 | 9.2245 |
| 202188_at | 9688 | NUP93 | nucleoporin 93kDa | 0.06293 | 0.8224 | 0.9129 | 9.1834 | 9.1936 | 9.0741 | 9.3686 | 9.1181 | 9.5469 | 8.6715 | 9.6721 | 9.18 | 9.2368 | 9.1707 | 9.3635 |
| 203373_at | 8835 | SOCS2 | suppressor of cytokine signaling 2 | 0.059 | 0.7365 | 0.8575 | 6.4857 | 5.7124 | 9.0651 | 5.9854 | 5.7626 | 6.1518 | 6.8203 | 6.1518 | 6.8095 | 6.6738 | 6.3178 | 5.7924 |
| 202703_at | 8446 | DUSP11 | dual specificity phosphatase 11 (RNA/RNP complex 1-interacting) | 0.0585 | 0.9261 | 0.9605 | 9.9633 | 9.7268 | 9.7626 | 9.7626 | 9.6384 | 9.7894 | 9.6523 | 9.3977 | 10.1202 | 9.9746 | 9.6634 | 9.7072 |
| 207877_s_at | 4931 | NVL | nuclear VCP-like | 0.05804 | 0.8244 | 0.9141 | 10.212 | 9.7894 | 11.4259 | 10.5704 | 10.4396 | 11.8824 | 8.7027 | 11.0671 | 12.5394 | 11.1258 | 8.5903 | 11.4675 |
| 209263_x_at | 7106 | TSPAN4 | tetraspanin 4 | 0.05791 | 0.8523 | 0.9315 | 11.7779 | 11.7779 | 11.4922 | 11.5382 | 11.9552 | 11.669 | 11.5736 | 11.7993 | 11.3634 | 11.7333 | 11.7779 | 11.8403 |
| 207143_at | 1021 | CDK6 | cyclin-dependent kinase 6 | 0.05749 | 0.8263 | 0.9142 | 4.309 | 5.1287 | 4.9409 | 4.4456 | 4.6365 | 4.3979 | 4.4114 | 4.7967 | 4.7967 | 4.2043 | 4.5291 | 4.9134 |
| 200083_at | 23326 | USP22 | ubiquitin specific peptidase 22 | 0.05361 | 0.9301 | 0.9626 | 12.3115 | 12.4521 | 12.1784 | 12.1186 | 12.1267 | 10.9477 | 12.0869 | 12.246 | 11.8403 | 11.2124 | 12.1519 | 12.2931 |
| 201976_s_at | 4651 | MYO10 | myosin X | 0.052 | 0.8942 | 0.9529 | 9.7119 | 9.5469 | 9.3913 | 9.1834 | 9.3614 | 9.3502 | 9.3913 | 9.5193 | 9.0132 | 9.4486 | 9.5933 | 9.459 |
| 202282_at | 3028 | HSD17B10 | hydroxysteroid (17-beta) dehydrogenase 10 | 0.05178 | 0.8403 | 0.9244 | 9.6206 | 9.5385 | 9.3502 | 9.6102 | 9.4293 | 9.5548 | 9.7514 | 9.8854 | 8.4496 | 9.6968 | 9.5451 | 9.5801 |
| 218051_s_at | 64943 | NT5DC2 | 5'-nucleotidase domain containing 2 | 0.05101 | 0.9122 | 0.9551 | 8.8705 | 7.7416 | 9.2225 | 9.4319 | 8.941 | 9.1448 | 9.1545 | 7.9514 | 9.0717 | 8.7482 | 8.9263 | 9.1754 |
| 201788_at | 11325 | DDX42 | DEAD (Asp-Glu-Ala-Asp) box polypeptide 42 | 0.04687 | 0.8563 | 0.9315 | 7.7998 | 8.9522 | 8.6279 | 8.6032 | 7.5507 | 7.6358 | 7.4609 | 7.7942 | 9.2134 | 7.708 | 9.0518 | 7.5507 |
| 219821_s_at | 54438 | GFOD1 | glucose-fructose oxidoreductase domain containing 1 | 0.04624 | 0.9641 | 0.9821 | 6.0438 | 5.7815 | 5.7914 | 5.8199 | 6.0338 | 6.0115 | 6.0728 | 5.505 | 6.1988 | 6.3704 | 5.8494 | 5.338 |
| 201058_s_at | 10398 | MYL9 | myosin, light chain 9, regulatory | 0.04498 | 0.9182 | 0.9573 | 10.8176 | 11.0671 | 10.8639 | 10.7988 | 11.0778 | 10.9582 | 10.7641 | 11.1114 | 10.4209 | 10.9943 | 11.1997 | 10.9837 |
| 218206_x_at | 51282 | SCAND1 | SCAN domain containing 1 | 0.04345 | 0.9281 | 0.9616 | 9.5703 | 9.4319 | 9.6129 | 9.5062 | 8.3782 | 8.4293 | 8.941 | 9.3545 | 7.6505 | 9.8338 | 9.5676 | 9.2281 |
| 209304_x_at | 4616 | GADD45B | growth arrest and DNA-damage-inducible, beta | 0.04164 | 0.9182 | 0.9573 | 7.708 | 5.7482 | 8.2999 | 8.1169 | 7.9604 | 7.9529 | 8.6205 | 5.3457 | 7.863 | 7.9848 | 7.3609 | 8.0884 |
| 203054_s_at | 6988 | TCTA | T-cell leukemia translocation altered gene | 0.04145 | 0.9401 | 0.9668 | 10.9182 | 10.8808 | 11.04 | 9.8572 | 9.7514 | 10.7254 | 10.7003 | 10.0766 | 11.2558 | 10.6659 | 9.7678 | 10.435 |
| 201817_at | 9690 | UBE3C | ubiquitin protein ligase E3C | 0.03827 | 0.8842 | 0.9482 | 9.804 | 9.7541 | 9.7268 | 10.5815 | 9.5062 | 10.5209 | 10.3671 | 10.0323 | 9.0741 | 10.5073 | 9.6671 | 10.0226 |
| 200882_s_at | 5710 | PSMD4 | proteasome (prosome, macropain) 26S subunit, non-ATPase, 4 | 0.03604 | 0.9242 | 0.9605 | 11.2734 | 11.3634 | 11.6464 | 11.7715 | 11.5185 | 11.6793 | 11.3791 | 11.7221 | 11.1114 | 11.6793 | 11.6464 | 11.6213 |
| 204256_at | 79071 | ELOVL6 | ELOVL fatty acid elongase 6 | 0.03417 | 0.9361 | 0.9658 | 8.9175 | 8.9219 | 8.8705 | 8.9367 | 8.855 | 9.0552 | 9.0276 | 9.0769 | 8.5681 | 8.8432 | 8.8266 | 9.1559 |
| 204605_at | 10668 | CGRRF1 | cell growth regulator with ring finger domain 1 | 0.03367 | 0.9022 | 0.9529 | 8.8131 | 8.0196 | 8.335 | 8.5389 | 9.2281 | 8.7838 | 8.4443 | 8.5834 | 8.3743 | 8.2196 | 8.5986 | 9.3347 |
| 207238_s_at | 5788 | PTPRC | protein tyrosine phosphatase, receptor type, C | 0.02984 | 0.8703 | 0.9405 | 5.5235 | 6.2018 | 6.6869 | 5.9959 | 6.0054 | 6.3516 | 5.8377 | 5.6765 | 7.4218 | 5.8494 | 5.7707 | 6.0209 |
| 204937_s_at | 10782 | ZNF274 | zinc finger protein 274 | 0.02765 | 0.9421 | 0.9678 | 10.8392 | 10.0183 | 10.212 | 10.7641 | 10.5324 | 10.3354 | 10.653 | 9.936 | 11.0534 | 10.5491 | 9.7626 | 10.6137 |
| 208992_s_at | 6774 | STAT3 | signal transducer and activator of transcription 3 (acute-phase response factor) | 0.02641 | 0.9002 | 0.9529 | 4.4285 | 3.5043 | 4.1186 | 4.2483 | 4.0151 | 3.924 | 4.0394 | 4.4949 | 4.6041 | 4.1342 | 3.4175 | 3.4175 |
| 202743_at | 8503 | PIK3R3 | phosphoinositide-3-kinase, regulatory subunit 3 (gamma) | 0.02501 | 0.8643 | 0.9361 | 5.2783 | 5.4454 | 5.5606 | 5.2783 | 5.2552 | 5.8835 | 5.1836 | 5.3633 | 5.6797 | 5.2594 | 5.0492 | 6.0728 |
| 218898_at | 79850 | FAM57A | family with sequence similarity 57, member A | 0.02388 | 0.9381 | 0.9668 | 6.9656 | 7.1221 | 7.7961 | 6.8095 | 6.741 | 7.8184 | 6.8515 | 7.8823 | 6.2812 | 6.8247 | 7.7844 | 7.4693 |
| 203013_at | 11319 | ECD | ecdysoneless homolog (Drosophila) | 0.02364 | 0.8942 | 0.9529 | 8.8233 | 8.402 | 8.9102 | 8.5037 | 8.4583 | 8.443 | 8.587 | 8.6336 | 8.2999 | 8.4924 | 8.4698 | 8.9937 |
| 213343_s_at | 81544 | GDPD5 | glycerophosphodiester phosphodiesterase domain containing 5 | 0.02329 | 0.9261 | 0.9605 | 6.2018 | 6.4262 | 9.0518 | 6.4485 | 6.6336 | 6.3476 | 6.4611 | 6.2938 | 9.5056 | 6.1199 | 6.1227 | 6.2681 |
| 212231_at | 23014 | FBXO21 | F-box protein 21 | 0.02329 | 0.9441 | 0.9689 | 9.8141 | 9.631 | 9.4533 | 9.6129 | 9.2225 | 9.6882 | 9.6721 | 9.5059 | 9.9558 | 9.7929 | 9.6384 | 8.7702 |
| 201052_s_at | 9491 | PSMF1 | proteasome (prosome, macropain) inhibitor subunit 1 (PI31) | 0.02152 | 0.9481 | 0.9699 | 7.2595 | 9.3223 | 7.6251 | 7.3707 | 7.708 | 7.5316 | 7.9809 | 7.296 | 8.9175 | 8.181 | 7.0661 | 7.1843 |
| 201829_at | 10276 | NET1 | neuroepithelial cell transforming 1 | 0.01752 | 0.976 | 0.9861 | 7.6517 | 7.6616 | 7.6517 | 7.8184 | 7.8976 | 7.8588 | 7.973 | 7.868 | 7.4609 | 7.8091 | 7.8136 | 7.5831 |
| 203217_s_at | 8869 | ST3GAL5 | ST3 beta-galactoside alpha-2,3-sialyltransferase 5 | 0.01352 | 0.99 | 0.9961 | 5.1407 | 5.231 | 5.0447 | 5.3457 | 5.3955 | 4.997 | 5.3955 | 5.0596 | 5.0492 | 5.2205 | 5.251 | 5.1552 |
| 219208_at | 80204 | FBXO11 | F-box protein 11 | 0.01242 | 0.9341 | 0.9647 | 5.6421 | 5.7377 | 5.6292 | 5.9438 | 5.614 | 5.7626 | 5.8782 | 5.6828 | 5.424 | 6.1988 | 5.7815 | 5.3313 |
| 203302_at | 1633 | DCK | deoxycytidine kinase | 0.01176 | 0.8563 | 0.9315 | 10.7988 | 10.4803 | 10.365 | 10.7254 | 10.5568 | 8.609 | 9.6671 | 10.0849 | 10.3126 | 10.468 | 10.365 | 10.5568 |
| 203494_s_at | 9702 | CEP57 | centrosomal protein 57kDa | 0.01151 | 0.9102 | 0.9541 | 7.0111 | 7.0691 | 7.0631 | 7.1638 | 7.1596 | 6.0363 | 6.102 | 7.1029 | 8.1495 | 7.4112 | 5.8123 | 6.8357 |
| 203306_s_at | 10559 | SLC35A1 | solute carrier family 35 (CMP-sialic acid transporter), member A1 | 0.009888 | 0.998 | 0.998 | 8.6448 | 8.6367 | 8.6398 | 8.7482 | 8.5144 | 8.7702 | 8.7598 | 8.6173 | 8.7931 | 8.5037 | 8.6032 | 8.6652 |
| 201774_s_at | 9918 | NCAPD2 | non-SMC condensin I complex, subunit D2 | 0.009387 | 0.9541 | 0.975 | 8.2081 | 8.9323 | 9.4881 | 9.0984 | 8.8233 | 9.1423 | 8.5629 | 9.0651 | 9.2368 | 8.6986 | 8.511 | 9.5703 |
| 221741_s_at | 54915 | YTHDF1 | YTH domain family, member 1 | 0.002283 | 0.9621 | 0.9811 | 8.6032 | 8.5629 | 7.3569 | 8.109 | 7.0936 | 7.3752 | 8.181 | 8.7049 | 6.9655 | 8.0774 | 6.8975 | 8.2553 |
| 212894_at | 6832 | SUPV3L1 | suppressor of var1, 3-like 1 (S. cerevisiae) | -0.00007841 | 0.9182 | 0.9573 | 9.4687 | 9.7072 | 10.5669 | 9.4486 | 9.0741 | 9.2225 | 10.1028 | 7.5158 | 10.972 | 10.1384 | 9.4133 | 9.3465 |
| 211980_at | 1282 | COL4A1 | collagen, type IV, alpha 1 | -0.002074 | 0.996 | 0.998 | 12.2931 | 12.3315 | 12.1105 | 11.4977 | 12.2931 | 12.3655 | 12.1784 | 12.6305 | 11.2718 | 12.0613 | 12.3797 | 12.3797 |
| 205417_s_at | 1605 | DAG1 | dystroglycan 1 (dystrophin-associated glycoprotein 1) | -0.007494 | 0.998 | 0.998 | 4.9409 | 4.5075 | 4.3685 | 4.9025 | 5.0492 | 4.7515 | 4.7103 | 4.6432 | 4.6365 | 4.7827 | 4.7763 | 4.9889 |
| 203279_at | 9695 | EDEM1 | ER degradation enhancer, mannosidase alpha-like 1 | -0.009571 | 0.8962 | 0.9529 | 9.4438 | 9.0888 | 9.0518 | 9.2709 | 12.8415 | 8.9293 | 14.4357 | 8.855 | 8.7838 | 8.82 | 9.0166 | 8.9323 |
| 205581_s_at | 4846 | NOS3 | nitric oxide synthase 3 (endothelial cell) | -0.01145 | 0.992 | 0.9971 | 5.1836 | 5.4729 | 5.2462 | 4.769 | 5.0402 | 5.1552 | 4.8724 | 5.0402 | 5.1767 | 5.0596 | 5.2594 | 5.4894 |
| 216913_s_at | 23223 | RRP12 | ribosomal RNA processing 12 homolog (S. cerevisiae) | -0.01183 | 0.7984 | 0.8944 | 6.8975 | 7.2268 | 9.4116 | 6.8421 | 7.2099 | 7.3226 | 7.7458 | 7.4112 | 7.6906 | 7.3429 | 7.2156 | 7.5876 |
| 201379_s_at | 7165 | TPD52L2 | tumor protein D52-like 2 | -0.0139 | 0.994 | 0.998 | 7.5876 | 7.4967 | 7.5755 | 7.9335 | 6.4935 | 6.1518 | 6.63 | 7.7499 | 6.4096 | 8.009 | 6.661 | 7.8993 |
| 204725_s_at | 4690 | NCK1 | NCK adaptor protein 1 | -0.01649 | 0.9082 | 0.9541 | 9.7678 | 9.6968 | 9.9167 | 9.8461 | 10.3006 | 10.1666 | 9.3202 | 10.1384 | 10.0849 | 9.9273 | 10.1126 | 10.1666 |
| 36936_at | 7264 | TSTA3 | tissue specific transplantation antigen P35B | -0.01704 | 0.9461 | 0.9689 | 4.7256 | 5.0832 | 4.8917 | 4.9736 | 5.1287 | 5.1089 | 4.7515 | 5.101 | 4.6715 | 4.7827 | 5.0733 | 5.5822 |
| 202635_s_at | 5440 | POLR2K | polymerase (RNA) II (DNA directed) polypeptide K, 7.0kDa | -0.01713 | 0.8882 | 0.9515 | 10.9335 | 11.1997 | 10.9335 | 10.908 | 10.9837 | 11.0282 | 11.1114 | 11.04 | 10.9582 | 11.04 | 11.0993 | 10.7621 |
| 202722_s_at | 2673 | GFPT1 | glutamine--fructose-6-phosphate transaminase 1 | -0.02052 | 0.986 | 0.9931 | 7.2722 | 6.8837 | 7.1944 | 7.3226 | 7.0813 | 7.0064 | 6.9458 | 7.1885 | 7.1221 | 6.5948 | 7.2245 | 7.7522 |
| 204587_at | 9016 | SLC25A14 | solute carrier family 25 (mitochondrial carrier, brain), member 14 | -0.02202 | 0.9242 | 0.9605 | 6.3152 | 6.3108 | 6.4485 | 6.2156 | 6.6561 | 6.0639 | 6.4074 | 6.2805 | 6.1358 | 6.5745 | 6.3178 | 6.34 |
| 201778_s_at | 9813 | KIAA0494 | KIAA0494 | -0.02266 | 0.9102 | 0.9541 | 8.5389 | 8.7665 | 8.2903 | 8.7665 | 8.4647 | 8.7752 | 8.8131 | 8.3111 | 9.5385 | 8.9323 | 8.3545 | 7.7634 |
| 201152_s_at | 4154 | MBNL1 | muscleblind-like (Drosophila) | -0.02715 | 0.9601 | 0.9801 | 10.7641 | 10.5324 | 10.3293 | 9.8403 | 10.2447 | 9.8141 | 9.5783 | 10.7601 | 10.3293 | 9.7268 | 10.5295 | 10.7456 |
| 205467_at | 843 | CASP10 | caspase 10, apoptosis-related cysteine peptidase | -0.02895 | 0.7984 | 0.8944 | 5.8629 | 5.8589 | 6.9763 | 12.0358 | 5.8629 | 6.3739 | 7.1323 | 6.7698 | 8.27 | 7.0192 | 6.9125 | 7.3828 |
| 220643_s_at | 55179 | FAIM | Fas apoptotic inhibitory molecule | -0.02916 | 0.982 | 0.9912 | 7.6563 | 7.9299 | 7.7961 | 7.7416 | 9.3589 | 8.1062 | 7.9993 | 7.829 | 8.5534 | 8.1327 | 7.8045 | 8.4362 |
| 201466_s_at | 3725 | JUN | jun proto-oncogene | -0.03089 | 0.984 | 0.9921 | 9.644 | 9.8248 | 10.0183 | 9.7859 | 9.9273 | 9.8499 | 10.1099 | 9.618 | 10.6417 | 9.5056 | 9.6384 | 9.6412 |
| 211318_s_at | 8480 | RAE1 | RAE1 RNA export 1 homolog (S. pombe) | -0.03323 | 0.8383 | 0.9243 | 10.2447 | 10.0183 | 10.0766 | 10.1251 | 10.0938 | 10.3293 | 10.1154 | 10.3719 | 9.5548 | 10.2533 | 10.2864 | 10.3917 |
| 212614_at | 84159 | ARID5B | AT rich interactive domain 5B (MRF1-like) | -0.03428 | 0.9042 | 0.9539 | 8.3475 | 7.5108 | 7.3854 | 8.3294 | 7.437 | 7.4922 | 8.3111 | 7.197 | 8.0306 | 7.7599 | 7.5056 | 7.8729 |
| 208716_s_at | 54499 | TMCO1 | transmembrane and coiled-coil domains 1 | -0.03773 | 0.9461 | 0.9689 | 11.5736 | 11.6213 | 11.3791 | 11.3122 | 11.5656 | 11.7277 | 11.5278 | 11.7111 | 11.1857 | 11.4922 | 11.5278 | 11.8192 |
| 218193_s_at | 51026 | GOLT1B | golgi transport 1B | -0.03911 | 0.9401 | 0.9668 | 11.5032 | 11.5185 | 11.3254 | 3.4609 | 11.2734 | 11.3254 | 4.3213 | 11.4675 | 11.1592 | 11.7058 | 11.8403 | 11.3634 |
| 203120_at | 7159 | TP53BP2 | tumor protein p53 binding protein, 2 | -0.03925 | 0.9082 | 0.9541 | 10.3354 | 10.042 | 10.4209 | 10.2689 | 9.8804 | 10.1514 | 10.365 | 10.1486 | 10.5669 | 10.0849 | 10.0145 | 10.0183 |
| 213150_at | 3206 | HOXA10 | homeobox A10 | -0.03977 | 0.9741 | 0.9861 | 8.4914 | 8.3465 | 8.2613 | 8.4749 | 8.351 | 9.5193 | 8.5432 | 9.2487 | 8.3872 | 8.5903 | 8.2845 | 8.5834 |
| 208872_s_at | 7905 | REEP5 | receptor accessory protein 5 | -0.04026 | 0.9661 | 0.9832 | 7.8993 | 8.2903 | 8.335 | 8.5986 | 7.8823 | 8.5629 | 8.0939 | 8.7093 | 8.0684 | 8.2368 | 8.1782 | 8.4157 |
| 201014_s_at | 10606 | PAICS | phosphoribosylaminoimidazole carboxylase, phosphoribosylaminoimidazole succinocarboxamide synthetase | -0.04355 | 0.976 | 0.9861 | 7.3439 | 7.4693 | 7.2619 | 7.708 | 7.2619 | 7.4236 | 7.3306 | 7.7004 | 7.2378 | 7.3114 | 7.4609 | 7.5154 |
| 219061_s_at | 8270 | LAGE3 | L antigen family, member 3 | -0.04377 | 0.9022 | 0.9529 | 6.0209 | 6.0728 | 6.1916 | 8.8343 | 5.9556 | 6.0314 | 6.224 | 9.4948 | 5.7482 | 6.2589 | 6.2618 | 5.7914 |
| 217784_at | 10652 | YKT6 | YKT6 v-SNARE homolog (S. cerevisiae) | -0.04475 | 0.9202 | 0.9584 | 9.4486 | 9.0717 | 9.5783 | 8.9674 | 9.1386 | 9.148 | 9.2928 | 9.5248 | 8.7931 | 9.1423 | 9.4631 | 9.2702 |
| 203301_s_at | 9988 | DMTF1 | cyclin D binding myb-like transcription factor 1 | -0.04857 | 0.8623 | 0.9349 | 7.4474 | 7.9031 | 7.6754 | 6.7023 | 6.8615 | 6.5685 | 7.7599 | 7.4402 | 6.743 | 7.5151 | 7.6122 | 6.4074 |
| 201664_at | 10051 | SMC4 | structural maintenance of chromosomes 4 | -0.04954 | 0.8822 | 0.9471 | 10.1993 | 10.3293 | 10.2533 | 10.3719 | 10.365 | 9.9273 | 10.0226 | 10.448 | 9.9038 | 10.4396 | 10.2689 | 10.4861 |
| 217140_s_at | 7416 | VDAC1 | voltage-dependent anion channel 1 | -0.05162 | 0.8044 | 0.8991 | 10.3126 | 12.8415 | 12.4521 | 13.1644 | 12.3655 | 13.0802 | 11.5542 | 12.6867 | 12.9116 | 12.5073 | 12.7014 | 12.3315 |
| 201528_at | 6117 | RPA1 | replication protein A1, 70kDa | -0.05423 | 0.9082 | 0.9541 | 10.6708 | 10.1993 | 10.1993 | 10.1666 | 10.0938 | 10.3671 | 9.9424 | 10.5765 | 9.9633 | 10.5013 | 10.3671 | 10.5073 |
| 218407_x_at | 29937 | NENF | neudesin neurotrophic factor | -0.05537 | 0.8962 | 0.9529 | 11.3401 | 11.3254 | 11.2514 | 11.5857 | 11.3882 | 11.3401 | 11.5382 | 11.3791 | 11.2124 | 11.5857 | 11.3122 | 11.2899 |
| 217763_s_at | 11031 | RAB31 | RAB31, member RAS oncogene family | -0.05821 | 0.8982 | 0.9529 | 7.1323 | 7.2747 | 7.5831 | 8.9937 | 8.7786 | 7.4112 | 7.437 | 8.3092 | 8.5629 | 7.5316 | 7.2619 | 8.5629 |
| 204542_at | 10610 | ST6GALNAC2 | ST6 (alpha-N-acetyl-neuraminyl-2,3-beta-galactosyl-1,3)-N-acetylgalactosaminide alpha-2,6-sialyltransferase 2 | -0.05857 | 0.9002 | 0.9529 | 5.1407 | 4.6365 | 4.8366 | 4.841 | 4.769 | 10.3006 | 5.2031 | 10.3671 | 5.3719 | 4.997 | 4.9298 | 5.1925 |
| 206501_x_at | 2115 | ETV1 | ets variant 1 | -0.0588 | 0.8762 | 0.9428 | 8.0684 | 7.6517 | 8.009 | 8.7071 | 8.0669 | 7.7499 | 7.5056 | 8.3958 | 7.1596 | 8.6279 | 8.6965 | 8.2196 |
| 217080_s_at | 9455 | HOMER2 | homer homolog 2 (Drosophila) | -0.06226 | 0.9701 | 0.9841 | 9.1101 | 5.7707 | 6.2743 | 5.9149 | 5.6189 | 6.4888 | 6.1958 | 5.928 | 6.8804 | 6.736 | 6.8106 | 7.2999 |
| 200830_at | 5708 | PSMD2 | proteasome (prosome, macropain) 26S subunit, non-ATPase, 2 | -0.06297 | 0.8663 | 0.9372 | 11.7333 | 11.7544 | 11.669 | 11.7005 | 11.9623 | 11.7277 | 11.8527 | 12.0358 | 11.6213 | 11.5032 | 11.7715 | 11.8741 |
| 219952_s_at | 57192 | MCOLN1 | mucolipin 1 | -0.06381 | 0.7206 | 0.8511 | 6.0798 | 6.5529 | 5.9813 | 6.3383 | 6.4231 | 6.0937 | 5.7554 | 5.9718 | 5.7707 | 5.7223 | 5.8494 | 8.9824 |
| 202870_s_at | 991 | CDC20 | cell division cycle 20 homolog (S. cerevisiae) | -0.06499 | 0.9022 | 0.9529 | 6.1843 | 12.5912 | 12.6305 | 12.9116 | 6.6115 | 12.9116 | 6.5208 | 13.5329 | 6.5065 | 13.3248 | 13.2128 | 13.4075 |
| 202494_at | 10450 | PPIE | peptidylprolyl isomerase E (cyclophilin E) | -0.07003 | 0.8723 | 0.9405 | 9.7963 | 10.2737 | 9.7825 | 9.9558 | 10.0323 | 10.4209 | 9.7929 | 10.4396 | 10.1486 | 10.1771 | 10.0183 | 9.8902 |
| 208626_s_at | 10493 | VAT1 | vesicle amine transport protein 1 homolog (T. californica) | -0.0749 | 0.6487 | 0.8184 | 10.9837 | 10.9278 | 10.6708 | 5.2594 | 10.8361 | 10.8392 | 10.1486 | 10.3917 | 9.6861 | 10.0226 | 10.2533 | 10.15 |
| 202240_at | 5347 | PLK1 | polo-like kinase 1 | -0.07567 | 0.8563 | 0.9315 | 9.8644 | 9.1048 | 8.9817 | 10.1154 | 9.5056 | 9.153 | 9.148 | 9.0276 | 8.6965 | 9.3223 | 10.5209 | 10.5815 |
| 209015_s_at | 10049 | DNAJB6 | DnaJ (Hsp40) homolog, subfamily B, member 6 | -0.07668 | 0.7844 | 0.8879 | 10.232 | 9.9918 | 9.2051 | 9.804 | 8.6398 | 9.7514 | 10.7777 | 8.8085 | 10.5353 | 10.1993 | 9.4948 | 8.5064 |
| 212943_at | 9847 | KIAA0528 | KIAA0528 | -0.07769 | 0.6866 | 0.8397 | 12.5073 | 12.246 | 8.9476 | 12.1784 | 12.1519 | 12.2657 | 11.8741 | 12.0242 | 11.7111 | 11.8922 | 12.0869 | 11.451 |
| 210416_s_at | 11200 | CHEK2 | CHK2 checkpoint homolog (S. pombe) | -0.07946 | 0.8463 | 0.9279 | 6.7698 | 6.7449 | 6.6585 | 6.9531 | 10.3818 | 6.5887 | 6.7698 | 7.0095 | 6.7706 | 7.1944 | 10.7456 | 7.0585 |
| 89476_r_at | 79716 | NPEPL1 | aminopeptidase-like 1 | -0.08463 | 0.8104 | 0.9017 | 7.0936 | 6.794 | 6.9655 | 7.8823 | 7.088 | 7.8224 | 7.937 | 7.9732 | 6.6561 | 6.9125 | 6.9657 | 7.7314 |
| 204679_at | 3775 | KCNK1 | potassium channel, subfamily K, member 1 | -0.08464 | 0.7345 | 0.8562 | 5.424 | 8.7598 | 5.0949 | 5.1697 | 4.6841 | 4.9592 | 5.2031 | 4.6966 | 4.8567 | 7.9529 | 4.6041 | 8.4443 |
| 212846_at | 23076 | RRP1B | ribosomal RNA processing 1 homolog B (S. cerevisiae) | -0.08823 | 0.7884 | 0.8883 | 7.7018 | 8.1393 | 8.3782 | 7.7844 | 8.2126 | 8.4362 | 8.4647 | 8.4647 | 7.5108 | 7.6754 | 8.3263 | 8.6173 |
| 201671_x_at | 9097 | USP14 | ubiquitin specific peptidase 14 (tRNA-guanine transglycosylase) | -0.08829 | 0.7764 | 0.884 | 7.1423 | 7.2722 | 6.4951 | 6.8975 | 7.4055 | 7.278 | 6.5104 | 7.5322 | 7.4527 | 7.0974 | 6.7449 | 7.5658 |
| 202868_s_at | 10775 | POP4 | processing of precursor 4, ribonuclease P/MRP subunit (S. cerevisiae) | -0.0903 | 0.8802 | 0.946 | 9.9746 | 9.9902 | 10.1486 | 10.042 | 9.8902 | 10.1154 | 9.936 | 10.0808 | 9.8804 | 10.0183 | 10.1028 | 10.2689 |
| 212648_at | 54505 | DHX29 | DEAH (Asp-Glu-Ala-His) box polypeptide 29 | -0.0909 | 0.8104 | 0.9017 | 7.9171 | 7.5073 | 7.6251 | 7.8993 | 6.8101 | 6.9943 | 7.7004 | 7.7104 | 7.6517 | 7.4434 | 7.1323 | 7.4877 |
| 208510_s_at | 5468 | PPARG | peroxisome proliferator-activated receptor gamma | -0.09174 | 0.7804 | 0.8875 | 8.5681 | 8.2149 | 8.2613 | 8.5629 | 8.587 | 8.511 | 7.9848 | 8.1545 | 10.9335 | 8.1908 | 8.2126 | 7.9604 |
| 204313_s_at | 1385 | CREB1 | cAMP responsive element binding protein 1 | -0.0937 | 0.7625 | 0.8773 | 5.005 | 6.63 | 6.5559 | 6.7482 | 6.2546 | 5.2594 | 5.3313 | 4.9813 | 7.5729 | 6.5887 | 5.3633 | 7.716 |
| 204656_at | 6461 | SHB | Src homology 2 domain containing adaptor protein B | -0.09976 | 0.8004 | 0.8956 | 8.6611 | 9.0552 | 8.0281 | 7.4693 | 8.6578 | 7.7756 | 8.1709 | 7.9728 | 10.0226 | 8.0719 | 8.1062 | 8.1415 |
| 202854_at | 3251 | HPRT1 | hypoxanthine phosphoribosyltransferase 1 | -0.1023 | 0.7485 | 0.8653 | 10.2864 | 9.4724 | 9.5248 | 10.0145 | 9.4438 | 9.5248 | 10.8331 | 9.3037 | 9.148 | 9.6482 | 10.3354 | 9.6105 |
| 202647_s_at | 4893 | NRAS | neuroblastoma RAS viral (v-ras) oncogene homolog | -0.104 | 0.7206 | 0.8511 | 8.9954 | 8.9219 | 9.0166 | 9.2051 | 9.1737 | 8.6652 | 9.2787 | 8.4914 | 9.1181 | 9.1936 | 8.9854 | 9.2134 |
| 201560_at | 25932 | CLIC4 | chloride intracellular channel 4 | -0.1043 | 0.7226 | 0.8524 | 14.4357 | 13.7451 | 14.1383 | 13.7451 | 14.2088 | 14.2088 | 13.6591 | 14.3206 | 13.962 | 14.1383 | 14.4357 | 14.3206 |
| 205130_at | 5891 | MOK | MOK protein kinase | -0.1056 | 0.7285 | 0.8546 | 6.661 | 6.868 | 6.4262 | 6.3933 | 7.0538 | 6.7227 | 7.6906 | 6.3339 | 6.5104 | 6.7706 | 7.1996 | 6.1479 |
| 203458_at | 6697 | SPR | sepiapterin reductase (7,8-dihydrobiopterin:NADP+ oxidoreductase) | -0.1084 | 0.7265 | 0.854 | 8.9824 | 8.901 | 8.7434 | 8.9175 | 6.7023 | 8.4647 | 8.6279 | 8.7931 | 8.3198 | 8.6545 | 8.7702 | 8.2638 |
| 201841_s_at | 3315 | HSPB1 | heat shock 27kDa protein 1 | -0.1094 | 0.7525 | 0.8679 | 12.417 | 12.6867 | 12.5394 | 12.4521 | 13.0242 | 13.0242 | 12.6867 | 12.8415 | 12.3797 | 12.9116 | 12.8415 | 12.7892 |
| 218039_at | 51203 | NUSAP1 | nucleolar and spindle associated protein 1 | -0.1104 | 0.7026 | 0.8461 | 9.3361 | 9.2695 | 9.1448 | 6.5385 | 9.1101 | 9.3202 | 9.7096 | 9.3255 | 9.5056 | 9.5801 | 9.5336 | 6.5948 |
| 202184_s_at | 55746 | NUP133 | nucleoporin 133kDa | -0.1115 | 0.7405 | 0.8601 | 8.8039 | 8.8039 | 8.7049 | 9.0741 | 8.7482 | 9.0083 | 8.82 | 8.6578 | 8.8501 | 8.8416 | 9.045 | 9.1448 |
| 203565_s_at | 4331 | MNAT1 | menage a trois homolog 1, cyclin H assembly factor (Xenopus laevis) | -0.1142 | 0.6347 | 0.8147 | 8.1908 | 9.684 | 8.1256 | 9.4881 | 9.4724 | 9.7825 | 9.1834 | 8.5986 | 9.4319 | 9.5013 | 9.1348 | 9.6721 |
| 219485_s_at | 5716 | PSMD10 | proteasome (prosome, macropain) 26S subunit, non-ATPase, 10 | -0.1163 | 0.8762 | 0.9428 | 7.2156 | 8.0719 | 8.1338 | 8.1545 | 8.0444 | 8.3102 | 7.3609 | 7.989 | 7.5056 | 9.4687 | 8.2797 | 8.1223 |
| 209586_s_at | 58497 | PRUNE | prune homolog (Drosophila) | -0.1174 | 0.7944 | 0.893 | 3.4175 | 3.8529 | 3.8792 | 3.924 | 3.8267 | 3.7012 | 3.4175 | 3.9934 | 3.5043 | 3.8013 | 3.8792 | 4.3843 |
| 209050_s_at | 5900 | RALGDS | ral guanine nucleotide dissociation stimulator | -0.1176 | 0.8543 | 0.9315 | 7.3956 | 6.1358 | 6.3152 | 6.1958 | 6.5208 | 6.0115 | 6.3593 | 6.1518 | 7.973 | 6.34 | 6.4004 | 6.1958 |
| 202908_at | 7466 | WFS1 | Wolfram syndrome 1 (wolframin) | -0.1211 | 0.8283 | 0.9154 | 8.4496 | 8.5629 | 8.6431 | 8.0403 | 8.206 | 8.3407 | 7.7545 | 9.2325 | 8.0774 | 8.0363 | 9.7541 | 8.1327 |
| 215980_s_at | 3508 | IGHMBP2 | immunoglobulin mu binding protein 2 | -0.1234 | 0.7864 | 0.8883 | 5.9503 | 5.8589 | 5.7924 | 5.7223 | 6.0338 | 5.4729 | 5.4539 | 5.4347 | 5.3719 | 5.4729 | 5.6828 | 8.4647 |
| 202446_s_at | 5359 | PLSCR1 | phospholipid scramblase 1 | -0.1246 | 0.8403 | 0.9244 | 8.1327 | 8.3111 | 7.6616 | 8.1223 | 8.4924 | 8.1665 | 7.9217 | 7.9069 | 7.6467 | 7.8184 | 10.7843 | 7.9171 |
| 212047_s_at | 26001 | RNF167 | ring finger protein 167 | -0.1288 | 0.6467 | 0.8184 | 8.1908 | 8.82 | 9.4724 | 8.3629 | 8.7049 | 9.3288 | 9.3589 | 9.2051 | 9.3288 | 8.1709 | 8.206 | 9.4687 |
| 202633_at | 11073 | TOPBP1 | topoisomerase (DNA) II binding protein 1 | -0.1342 | 0.6427 | 0.8163 | 9.0602 | 8.8898 | 9.0083 | 9.0091 | 8.7598 | 8.9954 | 9.2281 | 8.8807 | 9.1448 | 8.6986 | 9.1834 | 8.8501 |
| 201519_at | 9868 | TOMM70A | translocase of outer mitochondrial membrane 70 homolog A (S. cerevisiae) | -0.1347 | 0.996 | 0.998 | 9.4724 | 9.3288 | 9.3037 | 9.4116 | 9.4116 | 9.2979 | 9.0829 | 9.5548 | 9.0242 | 9.1181 | 10.5013 | 9.451 |
| 202447_at | 1666 | DECR1 | 2,4-dienoyl CoA reductase 1, mitochondrial | -0.1364 | 0.7824 | 0.8879 | 9.5933 | 9.618 | 9.4948 | 9.5548 | 9.5434 | 9.644 | 9.4724 | 9.8338 | 9.388 | 9.5221 | 9.804 | 9.6206 |
| 218093_s_at | 55608 | ANKRD10 | ankyrin repeat domain 10 | -0.1379 | 0.6707 | 0.8292 | 7.2813 | 6.7607 | 6.5065 | 6.618 | 7.2813 | 7.3028 | 7.3439 | 7.5316 | 7.1524 | 7.5056 | 6.4888 | 6.443 |
| 202797_at | 22908 | SACM1L | SAC1 suppressor of actin mutations 1-like (yeast) | -0.1381 | 0.6946 | 0.8439 | 10.5815 | 10.2864 | 10.5295 | 10.459 | 10.448 | 10.5704 | 10.6137 | 10.633 | 10.2689 | 10.28 | 10.5568 | 10.7843 |
| 201069_at | 4313 | MMP2 | matrix metallopeptidase 2 (gelatinase A, 72kDa gelatinase, 72kDa type IV collagenase) | -0.1383 | 0.6567 | 0.8202 | 9.3768 | 9.9273 | 9.5971 | 9.7401 | 9.7584 | 9.6153 | 9.6788 | 9.7072 | 9.936 | 9.6814 | 9.388 | 9.9491 |
| 202776_at | 30836 | DNTTIP2 | deoxynucleotidyltransferase, terminal, interacting protein 2 | -0.1412 | 0.6387 | 0.8155 | 11.1258 | 10.9335 | 11.1746 | 11.2734 | 10.9182 | 11.1857 | 11.669 | 11.4259 | 11.4259 | 10.1666 | 11.3882 | 11.1114 |
| 204949_at | 3385 | ICAM3 | intercellular adhesion molecule 3 | -0.1421 | 0.8942 | 0.9529 | 6.0527 | 6.6394 | 6.4052 | 6.3476 | 6.2649 | 6.4672 | 5.9438 | 6.2451 | 5.9854 | 6.3361 | 8.3629 | 6.2531 |
| 201174_s_at | 54386 | TERF2IP | telomeric repeat binding factor 2, interacting protein | -0.1469 | 0.5928 | 0.7969 | 11.8741 | 11.6252 | 11.7715 | 11.6464 | 11.8824 | 11.5032 | 11.7779 | 11.8192 | 11.8527 | 11.8403 | 11.6793 | 11.5656 |
| 205026_at | 6777 | STAT5B | signal transducer and activator of transcription 5B | -0.1478 | 0.7006 | 0.8461 | 4.0602 | 4.1186 | 4.231 | 4.3213 | 4.5201 | 4.1498 | 4.0151 | 4.309 | 3.9934 | 4.4949 | 4.3685 | 4.5854 |
| 211725_s_at | 637 | BID | BH3 interacting domain death agonist | -0.149 | 0.6068 | 0.8072 | 6.6115 | 5.8306 | 5.8589 | 5.9556 | 6.7227 | 6.5793 | 6.7227 | 5.7025 | 7.707 | 6.6336 | 6.7023 | 5.2415 |
| 212811_x_at | 6509 | SLC1A4 | solute carrier family 1 (glutamate/neutral amino acid transporter), member 4 | -0.1494 | 0.7944 | 0.893 | 5.338 | 5.1925 | 5.5139 | 5.3097 | 5.2415 | 5.0596 | 5.0557 | 4.9813 | 9.1348 | 4.7967 | 4.9409 | 4.4625 |
| 203885_at | 23011 | RAB21 | RAB21, member RAS oncogene family | -0.152 | 0.6168 | 0.8095 | 7.8588 | 9.1181 | 7.9869 | 9.153 | 7.913 | 8.7786 | 8.5432 | 7.5876 | 9.2225 | 9.5703 | 9.3768 | 7.8329 |
| 202545_at | 5580 | PRKCD | protein kinase C, delta | -0.1536 | 0.6287 | 0.8112 | 4.6715 | 5.2031 | 5.3719 | 5.5373 | 5.5688 | 5.251 | 5.4347 | 5.231 | 4.9736 | 5.4894 | 5.3313 | 5.663 |
| 200071_at | 10285 | SMNDC1 | survival motor neuron domain containing 1 | -0.1541 | 0.6647 | 0.8249 | 9.331 | 9.2787 | 9.3953 | 9.4533 | 9.4319 | 9.4948 | 9.3361 | 9.3686 | 9.459 | 9.4187 | 9.5451 | 9.4001 |
| 218435_at | 29103 | DNAJC15 | DnaJ (Hsp40) homolog, subfamily C, member 15 | -0.1564 | 0.6108 | 0.8072 | 9.3589 | 9.3635 | 9.2368 | 9.3589 | 9.2264 | 9.2281 | 9.7541 | 8.7931 | 10.1993 | 8.82 | 9.4133 | 9.3686 |
| 213844_at | 3202 | HOXA5 | homeobox A5 | -0.1588 | 0.6208 | 0.8095 | 8.3294 | 8.6715 | 8.8131 | 7.6412 | 7.6283 | 8.4749 | 8.4749 | 8.1923 | 7.989 | 8.4583 | 8.6398 | 8.5211 |
| 202528_at | 2582 | GALE | UDP-galactose-4-epimerase | -0.1598 | 0.6347 | 0.8147 | 8.8501 | 8.7702 | 8.6715 | 8.8761 | 8.5389 | 7.513 | 8.6545 | 8.8416 | 8.3958 | 8.4093 | 8.6652 | 8.9674 |
| 209112_at | 1027 | CDKN1B | cyclin-dependent kinase inhibitor 1B (p27, Kip1) | -0.1673 | 0.6507 | 0.8184 | 10.8639 | 10.972 | 11.0534 | 10.9335 | 10.8479 | 10.972 | 11.0563 | 11.4025 | 10.5491 | 10.8479 | 10.9943 | 11.1592 |
| 202680_at | 2961 | GTF2E2 | general transcription factor IIE, polypeptide 2, beta 34kDa | -0.1679 | 0.6427 | 0.8163 | 8.7387 | 8.714 | 8.6545 | 8.6896 | 8.4293 | 8.8131 | 8.5037 | 8.8761 | 8.8432 | 8.6631 | 8.498 | 8.9954 |
| 212536_at | 23200 | ATP11B | ATPase, class VI, type 11B | -0.1681 | 0.5848 | 0.7905 | 8.7583 | 8.5237 | 8.4241 | 7.6867 | 7.6827 | 8.6032 | 9.0729 | 7.6219 | 9.8403 | 7.4186 | 8.3894 | 8.7304 |
| 205963_s_at | 9093 | DNAJA3 | DnaJ (Hsp40) homolog, subfamily A, member 3 | -0.1682 | 0.5868 | 0.7905 | 13.4075 | 12.6305 | 13.5329 | 11.9623 | 12.1105 | 12.0358 | 13.2745 | 11.669 | 14.2088 | 14.8479 | 12.5073 | 11.3002 |
| 205034_at | 9134 | CCNE2 | cyclin E2 | -0.1694 | 0.6587 | 0.821 | 6.4458 | 6.5948 | 6.4611 | 6.4611 | 5.0635 | 6.4888 | 6.5842 | 6.1743 | 6.5147 | 6.661 | 6.5147 | 5.9438 |
| 207540_s_at | 6850 | SYK | spleen tyrosine kinase | -0.1696 | 0.6108 | 0.8072 | 5.4828 | 5.614 | 6.0589 | 5.8629 | 6.0527 | 5.9718 | 6.0558 | 5.9059 | 6.4485 | 5.9356 | 5.5788 | 5.6765 |
| 200810_s_at | 1153 | CIRBP | cold inducible RNA binding protein | -0.1713 | 0.7745 | 0.8838 | 7.209 | 7.6101 | 7.4112 | 7.7145 | 7.5876 | 7.3439 | 7.1423 | 7.5507 | 7.2869 | 8.5283 | 7.7386 | 7.3402 |
| 205811_at | 11232 | POLG2 | polymerase (DNA directed), gamma 2, accessory subunit | -0.1714 | 0.9162 | 0.9573 | 6.4857 | 6.3555 | 6.1777 | 6.4201 | 6.3516 | 6.2546 | 7.3028 | 6.3593 | 6.3908 | 6.0903 | 6.1874 | 6.2805 |
| 208891_at | 1848 | DUSP6 | dual specificity phosphatase 6 | -0.1743 | 0.6407 | 0.8163 | 11.0563 | 11.247 | 11.3122 | 12.3797 | 11.161 | 10.65 | 11.6232 | 11.5032 | 12.015 | 10.9306 | 11.7111 | 11.0563 |
| 201102_s_at | 5211 | PFKL | phosphofructokinase, liver | -0.1768 | 0.7405 | 0.8601 | 6.3272 | 6.3826 | 6.4201 | 6.4262 | 6.4611 | 6.6115 | 5.5976 | 6.0115 | 5.9772 | 5.8083 | 12.015 | 5.9854 |
| 203530_s_at | 6810 | STX4 | syntaxin 4 | -0.1779 | 0.5768 | 0.7868 | 9.3073 | 8.6545 | 9.2841 | 9.4224 | 9.1224 | 8.8163 | 9.684 | 8.941 | 10.0145 | 9.6814 | 8.5807 | 8.6754 |
| 204418_x_at | 2946 | GSTM2 | glutathione S-transferase mu 2 (muscle) | -0.1782 | 0.5349 | 0.7577 | 4.9592 | 5.005 | 5.1167 | 4.1186 | 5.1287 | 4.2483 | 5.005 | 5.1697 | 5.0402 | 4.1342 | 5.0402 | 5.0861 |
| 218571_s_at | 29082 | CHMP4A | charged multivesicular body protein 4A | -0.1791 | 0.5509 | 0.7686 | 8.3263 | 8.5757 | 8.5389 | 8.6279 | 8.6652 | 8.3102 | 8.4914 | 8.5037 | 8.3545 | 8.5177 | 8.6827 | 8.8424 |
| 214062_x_at | 4793 | NFKBIB | nuclear factor of kappa light polypeptide gene enhancer in B-cells inhibitor, beta | -0.1809 | 0.6248 | 0.8104 | 5.0492 | 5.2783 | 5.4729 | 5.6525 | 5.2953 | 5.694 | 5.3633 | 5.338 | 5.6525 | 5.2783 | 5.4828 | 5.8234 |
| 212910_at | 57215 | THAP11 | THAP domain containing 11 | -0.1848 | 0.6367 | 0.8151 | 6.9495 | 7.4743 | 7.1166 | 7.2619 | 7.0538 | 6.8421 | 7.1221 | 7.0631 | 7.1996 | 7.1002 | 7.1524 | 7.479 |
| 201185_at | 5654 | HTRA1 | HtrA serine peptidase 1 | -0.1849 | 0.5369 | 0.7577 | 6.5084 | 6.6032 | 6.224 | 6.0979 | 6.5385 | 6.3957 | 6.5208 | 6.4485 | 6.3826 | 6.7086 | 6.6478 | 6.1139 |
| 210038_at | 5588 | PRKCQ | protein kinase C, theta | -0.1855 | 0.5569 | 0.7731 | 6.4611 | 6.5178 | 6.5685 | 6.5208 | 6.6943 | 6.5104 | 6.6943 | 6.5887 | 6.6264 | 6.7706 | 6.6796 | 6.2197 |
| 203442_x_at | 256364 | EML3 | echinoderm microtubule associated protein like 3 | -0.1858 | 0.5629 | 0.7753 | 7.4434 | 7.5658 | 7.7545 | 7.5876 | 7.863 | 7.708 | 7.8858 | 7.7004 | 7.5322 | 7.4609 | 7.9217 | 7.7989 |
| 205120_s_at | 6443 | SGCB | sarcoglycan, beta (43kDa dystrophin-associated glycoprotein) | -0.1872 | 0.5749 | 0.7863 | 8.4014 | 9.6105 | 8.1062 | 8.714 | 8.5944 | 8.1602 | 8.2797 | 9.8403 | 7.9644 | 9.1737 | 8.3294 | 9.4631 |
| 204451_at | 8321 | FZD1 | frizzled family receptor 1 | -0.1875 | 0.5948 | 0.7969 | 6.7388 | 6.8837 | 7.95 | 7.2921 | 6.3826 | 7.6616 | 7.0846 | 6.9896 | 7.632 | 7.7545 | 6.7764 | 7.8447 |
| 209215_at | 10227 | MFSD10 | major facilitator superfamily domain containing 10 | -0.1878 | 0.5749 | 0.7863 | 10.7393 | 10.737 | 9.4319 | 9.5469 | 9.6755 | 10.1251 | 9.8878 | 10.2889 | 10.232 | 9.9918 | 10.3818 | 10.3671 |
| 210105_s_at | 2534 | FYN | FYN oncogene related to SRC, FGR, YES | -0.1901 | 0.6008 | 0.8016 | 6.3064 | 6.3669 | 6.1558 | 6.2589 | 6.0709 | 6.5462 | 6.2114 | 6.6943 | 6.0438 | 5.8494 | 6.63 | 6.9531 |
| 218794_s_at | 54957 | TXNL4B | thioredoxin-like 4B | -0.1914 | 0.6826 | 0.8377 | 7.356 | 7.4155 | 7.4833 | 6.7482 | 7.4434 | 7.4112 | 8.5091 | 7.0691 | 6.8203 | 7.1751 | 7.0491 | 8.4157 |
| 202738_s_at | 5257 | PHKB | phosphorylase kinase, beta | -0.197 | 0.5689 | 0.7814 | 6.9531 | 6.992 | 7.0192 | 6.9943 | 6.7713 | 7.1221 | 7.6219 | 6.224 | 7.6101 | 6.8753 | 6.9458 | 7.3439 |
| 213417_at | 6909 | TBX2 | T-box 2 | -0.2023 | 0.517 | 0.7453 | 5.9356 | 5.8163 | 5.5688 | 5.8083 | 5.424 | 5.9772 | 5.9059 | 5.8629 | 5.9149 | 6.04 | 5.663 | 5.6058 |
| 203343_at | 7358 | UGDH | UDP-glucose 6-dehydrogenase | -0.204 | 0.6926 | 0.8436 | 13.0802 | 9.2744 | 12.8415 | 12.8415 | 12.983 | 12.5394 | 12.5912 | 12.8646 | 12.2657 | 12.6305 | 12.5651 | 12.6867 |
| 208611_s_at | 6709 | SPTAN1 | spectrin, alpha, non-erythrocytic 1 (alpha-fodrin) | -0.2041 | 0.6088 | 0.8072 | 6.6478 | 6.492 | 6.3555 | 6.3476 | 6.3957 | 6.1199 | 6.3392 | 6.8155 | 6.1874 | 6.1777 | 6.5208 | 6.9209 |
| 201202_at | 5111 | PCNA | proliferating cell nuclear antigen | -0.2101 | 0.5589 | 0.7731 | 14.0469 | 13.962 | 13.8177 | 13.9002 | 13.962 | 14.0469 | 13.7451 | 14.2088 | 13.6591 | 14.2088 | 14.2088 | 14.1383 |
| 210719_s_at | 10362 | HMG20B | high mobility group 20B | -0.2106 | 0.513 | 0.7443 | 8.8807 | 9.4881 | 9.3614 | 9.4825 | 9.5703 | 9.5434 | 9.498 | 9.6925 | 8.9817 | 9.6206 | 9.8141 | 9.415 |
| 209875_s_at | 6696 | SPP1 | secreted phosphoprotein 1 | -0.2117 | 0.8263 | 0.9142 | 6.1169 | 6.1199 | 5.6698 | 6.3064 | 5.8408 | 5.7025 | 5.5822 | 11.1258 | 5.5606 | 5.5235 | 5.338 | 5.844 |
| 212500_at | 84890 | ADO | 2-aminoethanethiol (cysteamine) dioxygenase | -0.2135 | 0.8543 | 0.9315 | 7.4568 | 7.5569 | 7.3429 | 7.5507 | 7.3385 | 7.2607 | 7.1057 | 7.4527 | 6.5842 | 12.2657 | 7.0033 | 7.0126 |
| 221492_s_at | 64422 | ATG3 | ATG3 autophagy related 3 homolog (S. cerevisiae) | -0.2142 | 0.523 | 0.7466 | 10.1771 | 10.212 | 10.1099 | 10.1028 | 10.0808 | 9.7541 | 10.2447 | 10.4209 | 9.6968 | 10.1486 | 10.5353 | 9.9902 |
| 202230_s_at | 10523 | CHERP | calcium homeostasis endoplasmic reticulum protein | -0.2279 | 0.481 | 0.7205 | 7.3174 | 7.4112 | 7.4501 | 7.9479 | 7.716 | 7.4661 | 7.156 | 7.868 | 7.7004 | 7.9809 | 7.829 | 7.5073 |
| 206846_s_at | 10013 | HDAC6 | histone deacetylase 6 | -0.2337 | 0.509 | 0.7397 | 7.6906 | 7.7998 | 7.6467 | 7.9869 | 8.0669 | 7.8091 | 7.7356 | 8.2026 | 7.6827 | 8.0238 | 7.8823 | 7.9732 |
| 203857_s_at | 10954 | PDIA5 | protein disulfide isomerase family A, member 5 | -0.2352 | 0.485 | 0.7231 | 4.309 | 4.4456 | 4.0809 | 4.5382 | 4.3213 | 5.2953 | 4.9134 | 4.3843 | 5.2462 | 4.0151 | 4.2483 | 5.6828 |
| 212485_at | 23131 | GPATCH8 | G patch domain containing 8 | -0.2367 | 0.9002 | 0.9529 | 6.2805 | 6.4004 | 6.5426 | 6.5685 | 6.4672 | 6.6264 | 9.9902 | 6.661 | 5.8888 | 5.9149 | 6.0937 | 6.741 |
| 217766_s_at | 23585 | TMEM50A | transmembrane protein 50A | -0.2371 | 0.5269 | 0.7502 | 11.2718 | 11.1393 | 11.0282 | 11.3401 | 11.4145 | 11.1258 | 11.2734 | 11.5278 | 10.8392 | 11.2169 | 11.3906 | 11.7005 |
| 212287_at | 23512 | SUZ12 | suppressor of zeste 12 homolog (Drosophila) | -0.242 | 0.7126 | 0.8488 | 10.5073 | 10.4209 | 10.4861 | 10.0527 | 6.9694 | 10.2864 | 10.1666 | 10.1154 | 10.2948 | 9.9558 | 10.1514 | 10.2247 |
| 209603_at | 2625 | GATA3 | GATA binding protein 3 | -0.2443 | 0.5369 | 0.7577 | 4.3527 | 4.0394 | 4.4114 | 8.7838 | 3.9717 | 4.1186 | 9.4438 | 8.9937 | 4.5201 | 4.7616 | 4.1776 | 4.2043 |
| 202241_at | 10221 | TRIB1 | tribbles homolog 1 (Drosophila) | -0.247 | 0.495 | 0.7302 | 7.2268 | 7.5703 | 7.706 | 7.2869 | 6.9655 | 7.1524 | 8.1923 | 7.0585 | 8.0939 | 7.6563 | 6.9896 | 7.1221 |
| 200783_s_at | 3925 | STMN1 | stathmin 1 | -0.2481 | 0.5329 | 0.7565 | 9.4293 | 9.3614 | 9.6523 | 9.5867 | 9.5013 | 9.8644 | 9.5056 | 9.4438 | 9.6129 | 9.6968 | 9.7072 | 9.9764 |
| 202040_s_at | 5927 | KDM5A | lysine (K)-specific demethylase 5A | -0.2495 | 0.9681 | 0.9841 | 8.1415 | 7.9529 | 8.0363 | 7.8091 | 7.9194 | 7.852 | 7.7458 | 8.0047 | 7.5461 | 10.6137 | 7.8045 | 7.913 |
| 202950_at | 1429 | CRYZ | crystallin, zeta (quinone reductase) | -0.2511 | 0.4611 | 0.7068 | 10.4861 | 10.7254 | 10.1251 | 10.2737 | 10.232 | 10.1384 | 10.2247 | 10.4155 | 10.365 | 10.7456 | 10.6859 | 10.232 |
| 217809_at | 28969 | BZW2 | basic leucine zipper and W2 domains 2 | -0.2527 | 0.7305 | 0.8546 | 9.415 | 9.4116 | 9.4631 | 9.2787 | 9.5783 | 9.4724 | 10.908 | 9.3545 | 9.153 | 9.7401 | 9.2487 | 9.3614 |
| 201146_at | 4780 | NFE2L2 | nuclear factor (erythroid-derived 2)-like 2 | -0.2539 | 0.4471 | 0.693 | 8.6896 | 8.9532 | 8.5037 | 8.6431 | 8.8501 | 8.6545 | 9.0166 | 8.5091 | 9.2979 | 8.5807 | 8.8852 | 8.7006 |
| 200814_at | 5720 | PSME1 | proteasome (prosome, macropain) activator subunit 1 (PA28 alpha) | -0.2555 | 0.489 | 0.7257 | 10.3719 | 10.3917 | 10.3917 | 10.7003 | 10.2737 | 10.5073 | 10.2864 | 10.7777 | 10.1514 | 10.653 | 10.6659 | 10.7254 |
| 212875_s_at | 25966 | C2CD2 | C2 calcium-dependent domain containing 2 | -0.2589 | 0.4451 | 0.691 | 5.0635 | 4.9889 | 4.7967 | 4.9592 | 4.6841 | 4.7616 | 5.1697 | 5.0596 | 4.9216 | 4.882 | 4.9134 | 4.7616 |
| 213419_at | 323 | APBB2 | amyloid beta (A4) precursor protein-binding, family B, member 2 | -0.2595 | 0.519 | 0.7453 | 7.7129 | 7.852 | 7.7657 | 7.4568 | 7.2722 | 7.2291 | 7.8544 | 7.6144 | 9.5469 | 7.7499 | 7.5507 | 6.8018 |
| 203705_s_at | 8324 | FZD7 | frizzled family receptor 7 | -0.2604 | 0.6747 | 0.831 | 7.9809 | 8.2368 | 7.8588 | 7.8729 | 7.7499 | 8.0047 | 7.913 | 9.2744 | 7.5154 | 8.2368 | 8.1169 | 7.852 |
| 201620_at | 8720 | MBTPS1 | membrane-bound transcription factor peptidase, site 1 | -0.2642 | 0.3972 | 0.6432 | 6.3631 | 6.4201 | 6.0482 | 6.1874 | 6.3152 | 6.4376 | 6.492 | 6.102 | 6.6394 | 6.2197 | 6.3669 | 6.5065 |
| 215903_s_at | 23139 | MAST2 | microtubule associated serine/threonine kinase 2 | -0.2644 | 0.4451 | 0.691 | 7.4625 | 7.7314 | 7.5538 | 7.7942 | 8.1893 | 7.7386 | 7.7681 | 7.7268 | 7.9457 | 7.9604 | 7.8993 | 7.7386 |
| 202813_at | 6894 | TARBP1 | TAR (HIV-1) RNA binding protein 1 | -0.265 | 0.483 | 0.7212 | 8.9527 | 7.7942 | 9.0242 | 9.2979 | 8.7883 | 9.1737 | 9.1754 | 9.3347 | 8.7151 | 8.6205 | 9.3589 | 9.1936 |
| 212795_at | 23325 | KIAA1033 | KIAA1033 | -0.2695 | 0.6946 | 0.8439 | 9.1545 | 3.7012 | 8.8266 | 8.907 | 9.0166 | 9.0242 | 8.5237 | 8.82 | 8.7304 | 8.7117 | 8.7883 | 8.7161 |
| 205500_at | 727 | C5 | complement component 5 | -0.271 | 0.523 | 0.7466 | 5.7025 | 5.7626 | 5.922 | 5.6828 | 5.7223 | 5.8083 | 5.614 | 5.7482 | 6.2681 | 5.5856 | 6.0558 | 5.9059 |
| 214074_s_at | 2017 | CTTN | cortactin | -0.2727 | 0.4192 | 0.6633 | 6.6336 | 7.1524 | 7.1801 | 7.5658 | 6.6943 | 6.6738 | 7.168 | 7.042 | 6.7706 | 7.1751 | 7.4501 | 7.2869 |
| 221081_s_at | 79961 | DENND2D | DENN/MADD domain containing 2D | -0.2733 | 0.4012 | 0.6453 | 6.1169 | 6.2531 | 5.8377 | 6.2531 | 6.0589 | 5.7914 | 6.3205 | 5.8835 | 6.5208 | 6.0728 | 6.1777 | 6.0338 |
| 218807_at | 10451 | VAV3 | vav 3 guanine nucleotide exchange factor | -0.2741 | 0.3932 | 0.6388 | 5.6765 | 5.7914 | 5.2205 | 5.5139 | 5.6421 | 5.5916 | 5.7025 | 5.5542 | 5.8548 | 5.6058 | 5.5373 | 5.6982 |
| 201212_at | 5641 | LGMN | legumain | -0.2745 | 0.3932 | 0.6388 | 6.34 | 6.443 | 6.4672 | 6.5745 | 6.3669 | 6.2451 | 6.5529 | 6.5685 | 6.492 | 6.2531 | 6.5745 | 6.3933 |
| 221641_s_at | 23597 | ACOT9 | acyl-CoA thioesterase 9 | -0.2753 | 0.6228 | 0.8099 | 9.2744 | 9.1754 | 8.893 | 9.3347 | 9.0518 | 5.4454 | 8.7161 | 9.3073 | 8.6398 | 8.9527 | 9.4224 | 9.1722 |
| 203154_s_at | 10298 | PAK4 | p21 protein (Cdc42/Rac)-activated kinase 4 | -0.2785 | 0.4072 | 0.6496 | 6.599 | 6.6869 | 6.6647 | 6.9308 | 7.3914 | 6.9593 | 7.3089 | 6.5745 | 7.1944 | 6.6943 | 7.479 | 7.0631 |
| 208190_s_at | 51599 | LSR | lipolysis stimulated lipoprotein receptor | -0.2795 | 0.4152 | 0.6581 | 5.9854 | 6.4096 | 6.4857 | 6.4857 | 6.0438 | 6.7086 | 6.7388 | 6.4074 | 6.3383 | 6.736 | 6.5065 | 6.2197 |
| 207039_at | 1029 | CDKN2A | cyclin-dependent kinase inhibitor 2A (melanoma, p16, inhibits CDK4) | -0.2862 | 0.6607 | 0.821 | 8.5807 | 14.0469 | 13.962 | 14.3206 | 14.1383 | 13.8177 | 14.0469 | 13.8177 | 13.7451 | 13.7451 | 13.8177 | 13.7451 |
| 212051_at | 147179 | WIPF2 | WAS/WASL interacting protein family, member 2 | -0.2965 | 0.3832 | 0.6342 | 8.3894 | 7.8136 | 7.9171 | 8.2196 | 8.0884 | 8.347 | 8.402 | 7.8993 | 8.8963 | 8.4327 | 7.9848 | 8.2149 |
| 206687_s_at | 5777 | PTPN6 | protein tyrosine phosphatase, non-receptor type 6 | -0.2985 | 0.3912 | 0.6388 | 4.7616 | 5.5426 | 5.2783 | 4.5201 | 4.9134 | 4.6498 | 5.3097 | 4.8917 | 4.7256 | 5.8163 | 5.101 | 5.2031 |
| 205897_at | 4776 | NFATC4 | nuclear factor of activated T-cells, cytoplasmic, calcineurin-dependent 4 | -0.2991 | 0.3633 | 0.6136 | 6.9347 | 7.2156 | 7.3385 | 6.7852 | 6.9763 | 6.9593 | 7.4218 | 6.8155 | 7.479 | 7.6616 | 6.9747 | 6.868 |
| 218421_at | 64781 | CERK | ceramide kinase | -0.2994 | 0.3573 | 0.6066 | 7.913 | 7.356 | 7.7458 | 7.2999 | 7.3854 | 7.0608 | 7.8773 | 7.5658 | 7.6684 | 8.0294 | 7.437 | 7.2549 |
| 200621_at | 1465 | CSRP1 | cysteine and glycine-rich protein 1 | -0.3042 | 0.6228 | 0.8099 | 13.5329 | 10.0849 | 13.2745 | 13.6591 | 13.6034 | 13.4469 | 13.2128 | 13.9002 | 13.0242 | 13.4469 | 13.6034 | 13.5329 |
| 202261_at | 6944 | VPS72 | vacuolar protein sorting 72 homolog (S. cerevisiae) | -0.3063 | 0.4012 | 0.6453 | 6.4322 | 6.2812 | 6.4376 | 5.7924 | 5.7914 | 6.1358 | 6.1743 | 5.9772 | 7.2468 | 6.5426 | 6.2066 | 6.1199 |
| 202811_at | 10617 | STAMBP | STAM binding protein | -0.3138 | 0.3573 | 0.6066 | 9.5548 | 9.5867 | 9.3465 | 9.8854 | 9.5933 | 9.6384 | 9.4881 | 9.7401 | 9.5385 | 9.7825 | 9.7514 | 9.9491 |
| 205085_at | 4998 | ORC1 | origin recognition complex, subunit 1 | -0.316 | 0.3214 | 0.5714 | 9.8804 | 9.2928 | 9.7963 | 8.51 | 8.6465 | 11.1208 | 12.2931 | 8.8432 | 11.5278 | 10.6859 | 9.6206 | 8.8266 |
| 203346_s_at | 22823 | MTF2 | metal response element binding transcription factor 2 | -0.3179 | 0.523 | 0.7466 | 8.2771 | 8.2553 | 8.4056 | 8.0306 | 8.5211 | 8.3294 | 8.3587 | 11.8741 | 8.5211 | 8.27 | 8.2126 | 7.7756 |
| 203208_s_at | 9650 | MTFR1 | mitochondrial fission regulator 1 | -0.3209 | 0.3872 | 0.6354 | 9.4319 | 9.4631 | 9.8499 | 9.4705 | 9.7825 | 9.2744 | 10.6837 | 9.2627 | 10.4832 | 10.0604 | 9.3137 | 9.18 |
| 209109_s_at | 7105 | TSPAN6 | tetraspanin 6 | -0.3232 | 0.6886 | 0.8397 | 8.1665 | 8.2519 | 8.0403 | 8.4293 | 8.2771 | 8.1495 | 8.0884 | 8.1106 | 7.8773 | 12.3655 | 8.2527 | 8.2519 |
| 206173_x_at | 2553 | GABPB1 | GA binding protein transcription factor, beta subunit 1 | -0.3235 | 0.4551 | 0.7031 | 9.0374 | 9.1423 | 9.0984 | 9.0485 | 9.2134 | 8.904 | 8.7838 | 9.8804 | 8.7387 | 9.6153 | 9.8804 | 8.8371 |
| 218595_s_at | 55127 | HEATR1 | HEAT repeat containing 1 | -0.3242 | 0.3653 | 0.6138 | 10.6417 | 8.3686 | 9.5933 | 10.5013 | 10.459 | 10.7988 | 9.8338 | 11.2899 | 10.6137 | 9.8902 | 10.6708 | 11.0165 |
| 202433_at | 10237 | SLC35B1 | solute carrier family 35, member B1 | -0.3246 | 0.3453 | 0.5935 | 9.7338 | 10.2533 | 10.1154 | 10.1926 | 10.0145 | 10.1926 | 10.0808 | 10.5295 | 9.7275 | 10.212 | 10.5073 | 10.4155 |
| 203546_at | 9670 | IPO13 | importin 13 | -0.3252 | 0.3613 | 0.6113 | 8.1122 | 7.3226 | 8.5091 | 7.9685 | 7.3569 | 8.2587 | 9.631 | 7.1373 | 10.4396 | 9.5971 | 7.3439 | 7.2245 |
| 218547_at | 79947 | DHDDS | dehydrodolichyl diphosphate synthase | -0.3255 | 0.3413 | 0.5898 | 7.042 | 7.1996 | 7.2722 | 7.4186 | 7.1057 | 7.209 | 7.0936 | 7.3419 | 7.5831 | 7.042 | 7.4661 | 7.3914 |
| 203228_at | 5050 | PAFAH1B3 | platelet-activating factor acetylhydrolase 1b, catalytic subunit 3 (29kDa) | -0.3265 | 0.2894 | 0.5443 | 6.7449 | 7.7681 | 6.7086 | 7.7599 | 6.8428 | 7.075 | 7.7998 | 7.8184 | 7.1221 | 6.8515 | 7.5569 | 7.4661 |
| 203395_s_at | 3280 | HES1 | hairy and enhancer of split 1, (Drosophila) | -0.3285 | 0.5309 | 0.7547 | 5.6165 | 5.8083 | 5.6189 | 5.6765 | 5.694 | 9.2841 | 6.1139 | 10.1993 | 8.6794 | 6.224 | 6.1479 | 6.6115 |
| 204126_s_at | 8318 | CDC45 | cell division cycle 45 homolog (S. cerevisiae) | -0.3323 | 0.3293 | 0.5783 | 8.5903 | 8.4014 | 8.9175 | 8.4919 | 10.1384 | 8.6414 | 9.2264 | 9.4116 | 9.6523 | 9.3913 | 8.3872 | 9.2744 |
| 205172_x_at | 1212 | CLTB | clathrin, light chain B | -0.3342 | 0.2774 | 0.5317 | 7.9457 | 8.135 | 6.7713 | 7.7314 | 8.1338 | 6.7738 | 8.7978 | 8.0684 | 7.7616 | 8.4443 | 6.718 | 8.4824 |
| 203897_at | 57149 | LYRM1 | LYR motif containing 1 | -0.3354 | 0.3293 | 0.5783 | 5.6828 | 5.424 | 5.2462 | 5.424 | 5.1836 | 5.1881 | 5.2868 | 5.5235 | 5.5478 | 5.1978 | 5.614 | 5.8234 |
| 219076_s_at | 5827 | PXMP2 | peroxisomal membrane protein 2, 22kDa | -0.3357 | 0.4591 | 0.7059 | 8.0884 | 7.9732 | 7.9217 | 7.9069 | 8.5534 | 8.4093 | 8.2263 | 8.2519 | 7.5461 | 7.9457 | 8.5237 | 12.983 |
| 202623_at | 55837 | EAPP | E2F-associated phosphoprotein | -0.336 | 0.7206 | 0.8511 | 6.3272 | 6.2218 | 6.0868 | 6.6074 | 6.2546 | 6.1255 | 12.246 | 6.0527 | 6.0903 | 6.1988 | 6.1227 | 6.3064 |
| 210754_s_at | 4067 | LYN | v-yes-1 Yamaguchi sarcoma viral related oncogene homolog | -0.3374 | 0.3673 | 0.615 | 6.5887 | 6.618 | 6.7353 | 6.8615 | 6.8753 | 6.887 | 6.8357 | 6.2938 | 7.5154 | 7.4625 | 6.5622 | 7.168 |
| 205899_at | 8900 | CCNA1 | cyclin A1 | -0.3377 | 0.2974 | 0.553 | 5.3955 | 5.5688 | 5.5856 | 5.7626 | 5.0861 | 5.2118 | 5.6292 | 5.505 | 5.4828 | 5.7865 | 5.8928 | 5.2783 |
| 205205_at | 5971 | RELB | v-rel reticuloendotheliosis viral oncogene homolog B | -0.3381 | 0.5509 | 0.7686 | 6.2589 | 6.0338 | 5.8589 | 6.1582 | 5.7377 | 5.9149 | 11.9142 | 5.928 | 5.8123 | 6.0527 | 6.0209 | 5.6292 |
| 203557_s_at | 5092 | PCBD1 | pterin-4 alpha-carbinolamine dehydratase/dimerization cofactor of hepatocyte nuclear factor 1 alpha | -0.3403 | 0.3932 | 0.6388 | 10.1099 | 9.8994 | 10.159 | 10.0849 | 9.9167 | 9.9392 | 9.8461 | 10.2737 | 10.1202 | 10.0527 | 10.0685 | 10.3323 |
| 205607_s_at | 57147 | SCYL3 | SCY1-like 3 (S. cerevisiae) | -0.3405 | 0.3553 | 0.6054 | 4.6966 | 4.4114 | 4.5201 | 5.2031 | 4.0809 | 5.0596 | 5.1287 | 4.7103 | 4.5618 | 5.3457 | 4.4456 | 5.5542 |
| 201391_at | 10131 | TRAP1 | TNF receptor-associated protein 1 | -0.3407 | 0.3134 | 0.5655 | 8.0403 | 8.6794 | 8.9532 | 9.0769 | 8.5834 | 9.2487 | 8.8501 | 9.1448 | 8.9896 | 8.9323 | 9.2264 | 8.7093 |
| 205452_at | 9488 | PIGB | phosphatidylinositol glycan anchor biosynthesis, class B | -0.3423 | 0.3174 | 0.5674 | 9.2264 | 8.9821 | 8.5807 | 7.8976 | 7.6754 | 8.6827 | 8.5986 | 9.039 | 8.6431 | 9.8141 | 8.0335 | 9.498 |
| 217906_at | 23588 | KLHDC2 | kelch domain containing 2 | -0.3437 | 0.3533 | 0.603 | 10.1666 | 10.0527 | 10.5209 | 10.6708 | 10.0604 | 11.1997 | 10.5704 | 10.6659 | 11.161 | 10.5295 | 10.647 | 10.5209 |
| 201502_s_at | 4792 | NFKBIA | nuclear factor of kappa light polypeptide gene enhancer in B-cells inhibitor, alpha | -0.3442 | 0.3174 | 0.5674 | 7.5322 | 7.4218 | 7.2386 | 7.4112 | 7.7998 | 7.7844 | 7.7832 | 7.9457 | 7.5569 | 7.2895 | 7.5876 | 8.0774 |
| 203209_at | 5985 | RFC5 | replication factor C (activator 1) 5, 36.5kDa | -0.3456 | 0.2675 | 0.5263 | 9.0407 | 9.0083 | 9.1559 | 8.8343 | 9.0717 | 8.9674 | 9.1545 | 8.9674 | 9.3545 | 9.1834 | 9.1754 | 8.8424 |
| 206770_s_at | 23443 | SLC35A3 | solute carrier family 35 (UDP-N-acetylglucosamine (UDP-GlcNAc) transporter), member A3 | -0.346 | 0.2715 | 0.5303 | 6.9495 | 6.2805 | 7.172 | 6.2156 | 6.4795 | 7.296 | 7.3609 | 6.492 | 7.042 | 7.0585 | 6.741 | 7.3997 |
| 215945_s_at | 23321 | TRIM2 | tripartite motif containing 2 | -0.3465 | 0.2974 | 0.553 | 9.0242 | 9.0957 | 9.1348 | 7.9335 | 7.852 | 8.1122 | 9.7096 | 8.8085 | 10.2533 | 9.4293 | 7.7681 | 8.3743 |
| 209367_at | 6813 | STXBP2 | syntaxin binding protein 2 | -0.3471 | 0.2754 | 0.5303 | 4.5201 | 4.2397 | 4.3213 | 3.7012 | 3.924 | 4.0602 | 4.7763 | 4.1186 | 4.7763 | 4.4456 | 4.2967 | 3.7758 |
| 200045_at | 23 | ABCF1 | ATP-binding cassette, sub-family F (GCN20), member 1 | -0.3474 | 0.3134 | 0.5655 | 9.9424 | 10.2447 | 10.2864 | 10.8331 | 10.5491 | 9.9392 | 10.1926 | 10.737 | 10.647 | 10.0808 | 10.7417 | 10.7621 |
| 212660_at | 23338 | PHF15 | PHD finger protein 15 | -0.3488 | 0.2754 | 0.5303 | 7.4877 | 6.7367 | 5.6058 | 6.0558 | 6.2114 | 5.7377 | 7.4767 | 6.9308 | 7.2921 | 5.8969 | 7.708 | 5.743 |
| 212557_at | 26036 | ZNF451 | zinc finger protein 451 | -0.3532 | 0.2735 | 0.5303 | 8.1754 | 8.1122 | 7.868 | 7.7018 | 8.0719 | 8.0155 | 8.0669 | 8.2771 | 7.7756 | 8.152 | 8.1327 | 8.326 |
| 218961_s_at | 11284 | PNKP | polynucleotide kinase 3'-phosphatase | -0.356 | 0.3792 | 0.6286 | 5.8629 | 6.1358 | 6.2531 | 6.1777 | 5.9503 | 5.8548 | 5.934 | 6.1169 | 5.6165 | 6.0209 | 11.3634 | 6.2589 |
| 203381_s_at | 348 | APOE | apolipoprotein E | -0.3563 | 0.2615 | 0.5198 | 6.5426 | 4.9298 | 4.9298 | 5.0733 | 4.5382 | 6.4096 | 6.6561 | 7.2841 | 5.4454 | 4.9353 | 6.7148 | 5.251 |
| 207042_at | 1870 | E2F2 | E2F transcription factor 2 | -0.3572 | 0.3752 | 0.6252 | 5.3457 | 4.3213 | 4.9592 | 5.0402 | 4.7515 | 4.5075 | 4.9298 | 4.4625 | 4.8917 | 12.7437 | 5.4036 | 4.2967 |
| 204711_at | 9851 | KIAA0753 | KIAA0753 | -0.3573 | 0.3194 | 0.57 | 6.7227 | 6.9009 | 7.042 | 6.9042 | 6.4458 | 6.7388 | 6.9888 | 6.7738 | 8.7027 | 6.9896 | 6.9387 | 6.4888 |
| 202491_s_at | 8518 | IKBKAP | inhibitor of kappa light polypeptide gene enhancer in B-cells, kinase complex-associated protein | -0.3586 | 0.3094 | 0.5635 | 8.6336 | 9.2134 | 8.3903 | 8.181 | 8.3227 | 8.5475 | 9.0518 | 9.0485 | 8.1574 | 9.0829 | 8.4093 | 9.2787 |
| 201985_at | 9897 | KIAA0196 | KIAA0196 | -0.3594 | 0.2675 | 0.5263 | 9.1737 | 9.1707 | 9.1048 | 8.7161 | 9.0099 | 8.829 | 9.1181 | 9.0729 | 9.5703 | 9.1448 | 9.1423 | 8.8628 |
| 205393_s_at | 1111 | CHEK1 | CHK1 checkpoint homolog (S. pombe) | -0.3628 | 0.3872 | 0.6354 | 10.3818 | 10.232 | 10.3126 | 10.2247 | 10.1771 | 10.2737 | 9.8499 | 10.4861 | 10.2737 | 10.448 | 14.3206 | 10.0604 |
| 204579_at | 2264 | FGFR4 | fibroblast growth factor receptor 4 | -0.3646 | 0.2854 | 0.5399 | 4.6041 | 4.6365 | 5.005 | 5.0949 | 4.9298 | 4.7103 | 5.2031 | 5.0492 | 5.0889 | 4.8322 | 4.8724 | 4.7616 |
| 208908_s_at | 831 | CAST | calpastatin | -0.365 | 0.4052 | 0.6486 | 10.3293 | 10.4155 | 10.5734 | 10.633 | 10.4209 | 4.997 | 11.2124 | 9.9902 | 11.5185 | 10.9131 | 10.4155 | 9.7514 |
| 201158_at | 4836 | NMT1 | N-myristoyltransferase 1 | -0.3655 | 0.3353 | 0.5815 | 7.8136 | 7.4501 | 7.1524 | 7.5073 | 7.4254 | 7.3402 | 8.0719 | 7.3065 | 8.1665 | 8.5389 | 7.2869 | 7.0908 |
| 209899_s_at | 22827 | PUF60 | poly-U binding splicing factor 60KDa | -0.3655 | 0.6188 | 0.8095 | 12.015 | 11.9552 | 11.985 | 11.8824 | 7.2291 | 12.0242 | 12.1105 | 12.1267 | 11.4798 | 11.9303 | 12.1784 | 12.0869 |
| 203709_at | 5261 | PHKG2 | phosphorylase kinase, gamma 2 (testis) | -0.3689 | 0.2675 | 0.5263 | 6.7164 | 6.8203 | 6.4376 | 6.6264 | 7.1002 | 6.718 | 7.4661 | 6.4951 | 7.3914 | 7.4254 | 6.9042 | 6.3339 |
| 209939_x_at | 8837 | CFLAR | CASP8 and FADD-like apoptosis regulator | -0.3708 | 0.4112 | 0.6539 | 5.844 | 6.0903 | 5.7223 | 5.928 | 5.4117 | 6.6264 | 6.0209 | 5.7223 | 7.1057 | 6.102 | 7.1057 | 5.8589 |
| 209531_at | 2954 | GSTZ1 | glutathione transferase zeta 1 | -0.3709 | 0.3114 | 0.5655 | 6.7852 | 7.0355 | 6.9209 | 6.8475 | 7.2099 | 6.9763 | 7.1002 | 9.7514 | 7.0033 | 7.3777 | 6.9531 | 6.5104 |
| 204995_at | 8851 | CDK5R1 | cyclin-dependent kinase 5, regulatory subunit 1 (p35) | -0.371 | 0.3593 | 0.609 | 4.7256 | 4.7827 | 4.7763 | 4.8322 | 4.2483 | 4.7763 | 4.6365 | 5.0832 | 4.9409 | 4.7515 | 4.741 | 4.8322 |
| 212166_at | 23039 | XPO7 | exportin 7 | -0.3723 | 0.2695 | 0.5281 | 8.0238 | 8.855 | 7.913 | 7.6669 | 7.5319 | 7.7145 | 8.8628 | 7.4693 | 9.0485 | 9.039 | 8.0939 | 7.7942 |
| 200825_s_at | 10525 | HYOU1 | hypoxia up-regulated 1 | -0.3732 | 0.3014 | 0.5572 | 8.6652 | 8.3294 | 8.4566 | 8.2845 | 8.5303 | 8.5346 | 9.0729 | 8.124 | 9.2281 | 8.6061 | 8.3743 | 8.6431 |
| 204809_at | 10845 | CLPX | ClpX caseinolytic peptidase X homolog (E. coli) | -0.3767 | 0.2615 | 0.5198 | 8.4293 | 8.351 | 8.5757 | 7.9771 | 8.3294 | 7.8993 | 8.1495 | 8.6652 | 8.3111 | 8.4241 | 8.5719 | 8.4548 |
| 209341_s_at | 3551 | IKBKB | inhibitor of kappa light polypeptide gene enhancer in B-cells, kinase beta | -0.3772 | 0.2655 | 0.5256 | 5.694 | 5.5426 | 5.6828 | 5.4117 | 5.5788 | 5.5478 | 5.7924 | 5.8083 | 6.0728 | 5.7626 | 5.3804 | 5.4539 |
| 217873_at | 51719 | CAB39 | calcium binding protein 39 | -0.3792 | 0.2335 | 0.4965 | 8.1034 | 7.8447 | 6.7515 | 6.6478 | 7.4474 | 6.7388 | 7.9299 | 7.8935 | 8.2553 | 7.042 | 7.868 | 7.1029 |
| 209380_s_at | 10057 | ABCC5 | ATP-binding cassette, sub-family C (CFTR/MRP), member 5 | -0.3801 | 0.2136 | 0.4662 | 4.5382 | 4.7515 | 4.7103 | 4.6365 | 4.4949 | 4.4456 | 4.8366 | 4.5947 | 11.0282 | 4.5618 | 4.4114 | 4.4456 |
| 218695_at | 54512 | EXOSC4 | exosome component 4 | -0.3809 | 0.2555 | 0.5131 | 5.7707 | 5.6421 | 5.8782 | 5.6189 | 6.0209 | 5.6765 | 6.0115 | 5.8377 | 5.8888 | 6.1558 | 5.7124 | 5.743 |
| 211758_x_at | 10190 | TXNDC9 | thioredoxin domain containing 9 | -0.3851 | 0.6248 | 0.8104 | 8.2845 | 4.7035 | 8.1958 | 8.2771 | 8.2329 | 8.1393 | 8.2613 | 8.1495 | 8.2999 | 8.4914 | 8.1545 | 8.1223 |
| 203218_at | 5601 | MAPK9 | mitogen-activated protein kinase 9 | -0.3886 | 0.2375 | 0.4995 | 10.2889 | 9.4825 | 10.448 | 9.6968 | 9.2627 | 9.5703 | 10.232 | 9.5434 | 10.0527 | 10.4304 | 9.8902 | 10.5765 |
| 200622_x_at | 808 | CALM3 | calmodulin 3 (phosphorylase kinase, delta) | -0.3924 | 0.2754 | 0.5303 | 5.7914 | 6.0527 | 6.9387 | 6.9896 | 6.3361 | 7.2156 | 6.6796 | 6.868 | 7.7756 | 7.0095 | 6.4292 | 6.9896 |
| 202599_s_at | 8204 | NRIP1 | nuclear receptor interacting protein 1 | -0.3968 | 0.1976 | 0.4484 | 9.3037 | 6.9731 | 9.18 | 6.7227 | 8.9532 | 8.8501 | 8.8628 | 9.0957 | 8.7702 | 8.6173 | 8.8371 | 8.9532 |
| 208708_x_at | 1983 | EIF5 | eukaryotic translation initiation factor 5 | -0.3977 | 0.489 | 0.7257 | 10.3671 | 10.3354 | 10.2737 | 10.5295 | 6.718 | 10.3917 | 10.1771 | 10.7157 | 10.2247 | 10.3006 | 10.7254 | 10.6659 |
| 203047_at | 6793 | STK10 | serine/threonine kinase 10 | -0.3981 | 0.2176 | 0.4718 | 6.5147 | 6.5405 | 5.9373 | 5.9438 | 6.2451 | 6.2546 | 6.443 | 6.3669 | 6.988 | 6.3516 | 6.04 | 6.6336 |
| 217722_s_at | 51335 | NGRN | neugrin, neurite outgrowth associated | -0.3994 | 0.2794 | 0.5317 | 7.1944 | 5.7124 | 6.8421 | 7.5348 | 7.028 | 7.0813 | 7.3752 | 7.1473 | 7.3569 | 7.7018 | 6.9531 | 7.0192 |
| 201432_at | 847 | CAT | catalase | -0.4003 | 0.2275 | 0.4859 | 8.5534 | 9.0428 | 9.1737 | 9.0091 | 8.9954 | 8.8628 | 9.1224 | 9.0552 | 9.4775 | 9.0729 | 8.8314 | 9.0922 |
| 212070_at | 9289 | GPR56 | G protein-coupled receptor 56 | -0.4085 | 0.3094 | 0.5635 | 8.7838 | 8.8371 | 9.1224 | 8.8131 | 8.9817 | 8.7482 | 9.3848 | 8.8898 | 9.631 | 9.3502 | 8.7161 | 8.6545 |
| 204617_s_at | 65057 | ACD | adrenocortical dysplasia homolog (mouse) | -0.4103 | 0.1796 | 0.4254 | 6.9888 | 8.4767 | 8.0306 | 7.5831 | 7.4877 | 6.4052 | 8.3475 | 7.4402 | 8.4093 | 8.3294 | 7.8993 | 7.4617 |
| 218014_at | 79902 | NUP85 | nucleoporin 85kDa | -0.4111 | 0.6148 | 0.8092 | 11.0165 | 11.04 | 3.924 | 11.04 | 10.9582 | 10.9837 | 11.2169 | 11.2124 | 11.0993 | 10.9306 | 11.0282 | 10.9278 |
| 204977_at | 1662 | DDX10 | DEAD (Asp-Glu-Ala-Asp) box polypeptide 10 | -0.4128 | 0.2794 | 0.5317 | 9.0769 | 9.3465 | 7.4186 | 9.0888 | 8.9175 | 8.7978 | 9.2695 | 9.2245 | 8.8784 | 8.9134 | 9.3465 | 9.2051 |
| 218102_at | 51071 | DERA | deoxyribose-phosphate aldolase (putative) | -0.4141 | 0.4631 | 0.7076 | 11.1393 | 7.8578 | 10.8479 | 11.0282 | 10.7843 | 10.6708 | 10.9406 | 10.9335 | 10.9943 | 11.0165 | 10.8392 | 10.879 |
| 221732_at | 124583 | CANT1 | calcium activated nucleotidase 1 | -0.418 | 0.2395 | 0.4995 | 11.0671 | 10.9837 | 10.8331 | 10.8639 | 10.8082 | 10.448 | 10.7988 | 10.9943 | 10.7456 | 11.3122 | 11.0671 | 11.161 |
| 212849_at | 8312 | AXIN1 | axin 1 | -0.4222 | 0.1697 | 0.4145 | 6.4096 | 6.0639 | 5.5478 | 5.7761 | 6.0937 | 6.0115 | 6.6943 | 6.1874 | 6.3593 | 6.3178 | 6.3476 | 5.6292 |
| 207700_s_at | 8202 | NCOA3 | nuclear receptor coactivator 3 | -0.4233 | 0.3673 | 0.615 | 9.2487 | 9.24 | 9.3073 | 9.1633 | 9.0242 | 9.1203 | 9.2225 | 9.6037 | 9.2264 | 9.0729 | 9.1448 | 9.9746 |
| 217950_at | 51070 | NOSIP | nitric oxide synthase interacting protein | -0.4258 | 0.2236 | 0.4805 | 6.0589 | 6.2218 | 6.0979 | 5.7025 | 6.1558 | 6.1139 | 6.1874 | 7.0491 | 5.8123 | 6.3957 | 6.0868 | 6.3516 |
| 203464_s_at | 22905 | EPN2 | epsin 2 | -0.4281 | 0.2076 | 0.4604 | 8.4647 | 8.5177 | 9.2695 | 9.4293 | 8.2824 | 9.1881 | 9.0428 | 8.8705 | 9.7678 | 8.9674 | 9.2225 | 9.3913 |
| 218856_at | 27242 | TNFRSF21 | tumor necrosis factor receptor superfamily, member 21 | -0.4347 | 0.2655 | 0.5256 | 8.7583 | 8.511 | 8.7093 | 9.5676 | 8.4443 | 9.3635 | 8.7568 | 9.5059 | 10.6708 | 9.331 | 8.8039 | 9.3055 |
| 209710_at | 2624 | GATA2 | GATA binding protein 2 | -0.4364 | 0.2016 | 0.4512 | 7.4218 | 7.17 | 7.1002 | 7.3551 | 7.3174 | 6.9125 | 7.2245 | 7.6635 | 7.075 | 7.479 | 7.6122 | 7.32 |
| 217496_s_at | 3416 | IDE | insulin-degrading enzyme | -0.4368 | 0.1637 | 0.4073 | 4.7827 | 4.7035 | 4.4949 | 4.7929 | 4.4456 | 4.3527 | 4.7929 | 5.1287 | 4.5618 | 8.27 | 4.4787 | 4.6715 |
| 205088_at | 10046 | MAMLD1 | mastermind-like domain containing 1 | -0.439 | 0.02994 | 0.1457 | 4.841 | 4.3843 | 4.4456 | 4.7462 | 4.769 | 4.5075 | 12.0358 | 4.5947 | 4.9889 | 4.9736 | 4.8917 | 4.5201 |
| 215884_s_at | 29978 | UBQLN2 | ubiquilin 2 | -0.44 | 0.1936 | 0.4424 | 7.7832 | 7.4661 | 7.7458 | 7.798 | 7.7756 | 7.8309 | 7.8588 | 7.8136 | 8.4785 | 8.0294 | 7.716 | 7.6511 |
| 218145_at | 57761 | TRIB3 | tribbles homolog 3 (Drosophila) | -0.4443 | 0.1876 | 0.4338 | 10.4396 | 10.448 | 9.5336 | 10.2876 | 10.0766 | 10.4155 | 11.0993 | 10.5491 | 12.0869 | 10.633 | 9.2787 | 10.9335 |
| 205046_at | 1062 | CENPE | centromere protein E, 312kDa | -0.4465 | 0.2794 | 0.5317 | 7.9604 | 7.6283 | 7.3777 | 8.5852 | 7.7942 | 7.6653 | 8.8163 | 9.0083 | 8.907 | 8.0487 | 7.4661 | 7.6827 |
| 203186_s_at | 6275 | S100A4 | S100 calcium binding protein A4 | -0.4496 | 0.2196 | 0.4751 | 8.8233 | 8.4919 | 8.5903 | 8.5903 | 8.4362 | 8.941 | 8.7665 | 8.6465 | 8.582 | 8.7752 | 9.0741 | 9.2979 |
| 219363_s_at | 51001 | MTERFD1 | MTERF domain containing 1 | -0.4498 | 0.1717 | 0.4145 | 10.4304 | 10.5209 | 10.6074 | 10.1099 | 10.3671 | 10.0849 | 10.6417 | 10.365 | 10.2864 | 10.5815 | 10.5704 | 10.6837 |
| 206170_at | 154 | ADRB2 | adrenergic, beta-2-, receptor, surface | -0.4505 | 0.1238 | 0.3508 | 6.6796 | 6.5104 | 6.3064 | 6.4376 | 6.1255 | 6.0438 | 6.3908 | 6.5948 | 9.0407 | 6.4826 | 6.4148 | 6.6394 |
| 205055_at | 3682 | ITGAE | integrin, alpha E (antigen CD103, human mucosal lymphocyte antigen 1; alpha polypeptide) | -0.458 | 0.1996 | 0.4512 | 9.5676 | 9.2051 | 9.3848 | 9.5783 | 9.5509 | 9.3953 | 9.3037 | 9.6634 | 9.5783 | 9.5469 | 10.0226 | 9.6102 |
| 204092_s_at | 6790 | AURKA | aurora kinase A | -0.4595 | 0.2216 | 0.4773 | 7.2747 | 7.2595 | 7.0064 | 7.2747 | 7.0974 | 6.868 | 7.1241 | 7.5099 | 7.0661 | 7.267 | 7.2595 | 7.6122 |
| 203973_s_at | 1052 | CEBPD | CCAAT/enhancer binding protein (C/EBP), delta | -0.4673 | 0.2495 | 0.5084 | 6.4262 | 7.0955 | 6.4795 | 6.6943 | 6.5842 | 6.3669 | 7.1843 | 6.5529 | 7.5729 | 8.9954 | 6.5405 | 6.3383 |
| 216598_s_at | 6347 | CCL2 | chemokine (C-C motif) ligand 2 | -0.4703 | 0.1657 | 0.4081 | 8.855 | 8.9674 | 8.6652 | 8.4583 | 8.6578 | 8.9134 | 8.6431 | 8.7665 | 9.045 | 8.8898 | 9.0651 | 9.7401 |
| 218529_at | 51293 | CD320 | CD320 molecule | -0.4728 | 0.2216 | 0.4773 | 10.5765 | 10.7621 | 10.7456 | 10.5353 | 10.6859 | 10.5013 | 10.5815 | 11.3188 | 10.3867 | 11.0671 | 10.9477 | 10.7777 |
| 201292_at | 7153 | TOP2A | topoisomerase (DNA) II alpha 170kDa | -0.4745 | 0.3154 | 0.567 | 12.5912 | 12.7014 | 12.9116 | 12.7437 | 12.7437 | 12.7892 | 12.7437 | 13.4075 | 12.6305 | 12.6867 | 13.0242 | 13.1644 |
| 219968_at | 51385 | ZNF589 | zinc finger protein 589 | -0.4761 | 0.1677 | 0.412 | 5.8629 | 6.3555 | 6.0209 | 5.7914 | 5.7924 | 5.3633 | 6.2451 | 5.9503 | 6.6796 | 6.4458 | 5.8928 | 5.8589 |
| 204781_s_at | 355 | FAS | Fas (TNF receptor superfamily, member 6) | -0.4764 | 0.1717 | 0.4145 | 4.7827 | 4.3527 | 4.9134 | 4.6041 | 4.2967 | 5.0447 | 4.7929 | 4.9409 | 4.6966 | 5.1287 | 5.2205 | 4.7103 |
| 211015_s_at | 3308 | HSPA4 | heat shock 70kDa protein 4 | -0.4765 | 0.2615 | 0.5198 | 11.7221 | 11.4922 | 11.5857 | 11.0534 | 11.2718 | 11.2365 | 13.1644 | 10.9837 | 13.4469 | 12.5651 | 11.4675 | 10.7417 |
| 204061_at | 5613 | PRKX | protein kinase, X-linked | -0.4769 | 0.1776 | 0.4227 | 6.1358 | 6.256 | 6.1139 | 5.8548 | 5.8928 | 6.3152 | 6.2618 | 5.9924 | 6.8533 | 6.2812 | 6.6032 | 6.0558 |
| 209100_at | 7866 | IFRD2 | interferon-related developmental regulator 2 | -0.4794 | 0.2076 | 0.4604 | 9.936 | 10.2689 | 6.868 | 10.1486 | 6.9387 | 9.9167 | 9.9633 | 10.2447 | 9.2559 | 10.0145 | 10.2447 | 10.1384 |
| 214404_x_at | 25803 | SPDEF | SAM pointed domain containing ets transcription factor | -0.4823 | 0.1956 | 0.4459 | 7.8224 | 7.6412 | 8.0444 | 7.9479 | 8.1415 | 7.479 | 8.2999 | 7.798 | 8.6827 | 7.95 | 8.2369 | 7.8224 |
| 221142_s_at | 55825 | PECR | peroxisomal trans-2-enoyl-CoA reductase | -0.486 | 0.1816 | 0.4291 | 6.9084 | 7.0585 | 7.3956 | 7.2386 | 7.075 | 7.3569 | 7.4474 | 7.168 | 7.7314 | 7.3429 | 7.1944 | 7.3047 |
| 218290_at | 55111 | PLEKHJ1 | pleckstrin homology domain containing, family J member 1 | -0.4895 | 0.1537 | 0.3945 | 8.2669 | 8.5064 | 8.5303 | 7.8568 | 8.27 | 8.4924 | 8.9134 | 8.2366 | 8.855 | 8.5211 | 8.4241 | 8.4914 |
| 205750_at | 670 | BPHL | biphenyl hydrolase-like (serine hydrolase) | -0.4929 | 0.1737 | 0.4163 | 8.4362 | 7.2268 | 7.4402 | 7.5569 | 7.1241 | 8.7598 | 7.8985 | 7.5793 | 10.4304 | 9.0083 | 7.868 | 8.9134 |
| 221610_s_at | 55620 | STAP2 | signal transducing adaptor family member 2 | -0.4958 | 0.00998 | 0.08955 | 4.6498 | 4.9736 | 4.8366 | 4.8724 | 4.7891 | 4.7929 | 5.0492 | 4.9216 | 5.5788 | 5.0861 | 4.8366 | 11.1258 |
| 213292_s_at | 23161 | SNX13 | sorting nexin 13 | -0.4966 | 0.2116 | 0.4629 | 8.6173 | 8.2638 | 8.901 | 8.9954 | 8.714 | 9.3333 | 10.0183 | 8.5681 | 10.1718 | 9.8499 | 8.9817 | 8.5389 |
| 202910_s_at | 976 | CD97 | CD97 molecule | -0.4971 | 0.1397 | 0.3775 | 7.798 | 7.6791 | 7.6827 | 7.913 | 7.5319 | 7.5658 | 7.9993 | 7.8224 | 7.6653 | 7.7634 | 7.868 | 7.8091 |
| 202689_at | 29890 | RBM15B | RNA binding motif protein 15B | -0.4998 | 0.1497 | 0.3894 | 7.8894 | 7.8091 | 8.0047 | 7.6219 | 8.0363 | 7.7998 | 8.135 | 7.7572 | 8.9954 | 7.798 | 8.1665 | 8.1034 |
| 217838_s_at | 51466 | EVL | Enah/Vasp-like | -0.5055 | 0.05788 | 0.2089 | 5.2415 | 5.1836 | 5.1767 | 5.1287 | 5.2031 | 5.2118 | 5.0889 | 5.3313 | 6.5948 | 5.424 | 5.2783 | 5.2205 |
| 201853_s_at | 994 | CDC25B | cell division cycle 25 homolog B (S. pombe) | -0.5057 | 0.1876 | 0.4338 | 11.5185 | 8.1495 | 11.8824 | 11.8403 | 12.0485 | 7.5793 | 11.3634 | 12.2657 | 11.247 | 11.7993 | 11.7993 | 11.9303 |
| 202185_at | 8985 | PLOD3 | procollagen-lysine, 2-oxoglutarate 5-dioxygenase 3 | -0.5062 | 0.1856 | 0.4322 | 11.1592 | 10.7777 | 11.0671 | 11.1592 | 11.2365 | 11.0563 | 11.04 | 11.2734 | 11.3791 | 10.972 | 11.5185 | 11.4259 |
| 206050_s_at | 6050 | RNH1 | ribonuclease/angiogenin inhibitor 1 | -0.5064 | 0.1537 | 0.3945 | 9.9558 | 5.6765 | 10.0226 | 9.9918 | 10.3209 | 10.3818 | 10.459 | 10.1028 | 10.737 | 10.4803 | 10.232 | 10.6074 |
| 203341_at | 10153 | CEBPZ | CCAAT/enhancer binding protein (C/EBP), zeta | -0.5101 | 0.1737 | 0.4163 | 8.3872 | 8.4824 | 9.6634 | 9.459 | 8.1256 | 8.6611 | 9.9764 | 9.922 | 9.3361 | 9.0467 | 8.7006 | 9.2245 |
| 206066_s_at | 5889 | RAD51C | RAD51 homolog C (S. cerevisiae) | -0.5123 | 0.1537 | 0.3945 | 6.9888 | 7.1423 | 6.9763 | 7.0491 | 7.168 | 6.8779 | 7.072 | 6.988 | 7.0491 | 7.3385 | 7.5755 | 7.1944 |
| 203639_s_at | 2263 | FGFR2 | fibroblast growth factor receptor 2 | -0.5194 | 0.2754 | 0.5303 | 6.2681 | 6.1811 | 6.8247 | 6.224 | 5.9356 | 5.7924 | 7.6754 | 5.7815 | 8.3629 | 7.2999 | 6.2451 | 6.1358 |
| 209572_s_at | 8726 | EED | embryonic ectoderm development | -0.5222 | 0.1557 | 0.3965 | 7.8773 | 8.0653 | 8.7665 | 8.6205 | 7.5614 | 8.7883 | 9.2134 | 8.6965 | 8.2587 | 8.5389 | 8.8163 | 8.7665 |
| 202708_s_at | 8349 | HIST2H2BE | histone cluster 2, H2be | -0.5222 | 0.2156 | 0.4695 | 5.8234 | 5.6828 | 5.7815 | 5.0492 | 5.5976 | 5.5373 | 6.8435 | 5.231 | 7.8544 | 6.7449 | 5.5478 | 5.3955 |
| 203911_at | 5909 | RAP1GAP | RAP1 GTPase activating protein | -0.5232 | 0.1637 | 0.4073 | 5.9149 | 6.2451 | 6.0482 | 6.0338 | 5.5478 | 5.5788 | 6.1558 | 6.0438 | 6.8421 | 5.8888 | 5.9373 | 6.5793 |
| 218358_at | 79174 | CRELD2 | cysteine-rich with EGF-like domains 2 | -0.5265 | 0.1717 | 0.4145 | 6.9084 | 7.3997 | 6.9125 | 7.2434 | 7.8204 | 8.1908 | 8.7702 | 7.5461 | 9.8338 | 8.6061 | 7.5158 | 7.075 |
| 204549_at | 9641 | IKBKE | inhibitor of kappa light polypeptide gene enhancer in B-cells, kinase epsilon | -0.5285 | 0.1277 | 0.357 | 4.7515 | 4.7616 | 5.0949 | 5.1287 | 4.8917 | 4.9216 | 5.6189 | 5.1881 | 5.101 | 5.1836 | 4.8366 | 4.9736 |
| 202986_at | 9915 | ARNT2 | aryl-hydrocarbon receptor nuclear translocator 2 | -0.5307 | 0.1238 | 0.3508 | 11.0282 | 10.3671 | 11.7993 | 10.0808 | 6.9943 | 10.5295 | 12.5651 | 10.0145 | 13.5329 | 12.4521 | 11.2558 | 10.5295 |
| 202093_s_at | 54623 | PAF1 | Paf1, RNA polymerase II associated factor, homolog (S. cerevisiae) | -0.5309 | 0.1577 | 0.4006 | 7.3752 | 7.4877 | 7.868 | 7.4474 | 7.7314 | 7.6505 | 7.8858 | 7.5703 | 8.4919 | 8.2523 | 7.7499 | 7.4743 |
| 201351_s_at | 10730 | YME1L1 | YME1-like 1 (S. cerevisiae) | -0.5356 | 0.1138 | 0.3292 | 8.0069 | 7.6684 | 7.7018 | 7.8224 | 7.509 | 8.0383 | 7.8544 | 8.0363 | 8.0155 | 7.7838 | 7.989 | 8.1782 |
| 212255_s_at | 27032 | ATP2C1 | ATPase, Ca++ transporting, type 2C, member 1 | -0.5361 | 0.1417 | 0.3797 | 8.1169 | 7.9848 | 7.8224 | 8.0444 | 7.7599 | 7.6989 | 7.973 | 8.0884 | 8.0281 | 8.152 | 7.8894 | 8.2999 |
| 202677_at | 5921 | RASA1 | RAS p21 protein activator (GTPase activating protein) 1 | -0.537 | 0.1916 | 0.4389 | 7.8483 | 8.1291 | 8.1223 | 8.2587 | 7.9685 | 7.9809 | 8.7883 | 8.5303 | 7.8894 | 7.973 | 8.5629 | 8.1923 |
| 202454_s_at | 2065 | ERBB3 | v-erb-b2 erythroblastic leukemia viral oncogene homolog 3 (avian) | -0.5374 | 0.1297 | 0.3595 | 4.0151 | 4.1637 | 4.6498 | 4.0151 | 4.1498 | 4.3979 | 4.2967 | 4.309 | 4.4114 | 4.3843 | 4.6432 | 4.741 |
| 205173_x_at | 965 | CD58 | CD58 molecule | -0.5378 | 0.1277 | 0.357 | 10.8911 | 10.6417 | 10.3354 | 10.4803 | 10.7456 | 10.6074 | 10.7456 | 11.0534 | 10.7157 | 10.8331 | 10.5765 | 10.972 |
| 202627_s_at | 5054 | SERPINE1 | serpin peptidase inhibitor, clade E (nexin, plasminogen activator inhibitor type 1), member 1 | -0.5443 | 0.1238 | 0.3508 | 12.5651 | 12.7892 | 12.8646 | 12.6867 | 12.8646 | 12.7437 | 12.8646 | 12.983 | 12.5912 | 12.8646 | 13.1644 | 13.0802 |
| 200895_s_at | 2288 | FKBP4 | FK506 binding protein 4, 59kDa | -0.5496 | 0.2076 | 0.4604 | 13.9002 | 13.6591 | 14.0469 | 13.4075 | 13.2128 | 13.5329 | 14.8479 | 13.6591 | 15 | 14.6935 | 13.6591 | 13.3248 |
| 219170_at | 79187 | FSD1 | fibronectin type III and SPRY domain containing 1 | -0.5613 | 0.1397 | 0.3775 | 6.8247 | 6.8533 | 7.2099 | 6.8804 | 6.5462 | 6.9896 | 8.8807 | 6.6738 | 7.9993 | 7.1801 | 7.4236 | 6.718 |
| 218742_at | 64428 | NARFL | nuclear prelamin A recognition factor-like | -0.5628 | 0.02794 | 0.1457 | 6.8804 | 6.8753 | 7.2869 | 7.0198 | 7.1751 | 7.5658 | 14.6935 | 7.0095 | 8.8784 | 8.2845 | 7.2502 | 7.4236 |
| 200816_s_at | 5048 | PAFAH1B1 | platelet-activating factor acetylhydrolase 1b, regulatory subunit 1 (45kDa) | -0.5638 | 0.1018 | 0.3054 | 8.8314 | 8.5681 | 8.7812 | 8.8163 | 8.4824 | 8.5629 | 8.8314 | 8.8343 | 9.3686 | 8.9854 | 8.7598 | 8.6398 |
| 209515_s_at | 5873 | RAB27A | RAB27A, member RAS oncogene family | -0.5724 | 0.1337 | 0.3643 | 8.0939 | 8.0403 | 8.2369 | 8.2102 | 7.8136 | 8.2369 | 8.0281 | 8.2613 | 8.8416 | 8.4548 | 8.0487 | 9.4775 |
| 202427_s_at | 25874 | BRP44 | brain protein 44 | -0.5751 | 0.1178 | 0.3387 | 10.4803 | 10.7003 | 10.2447 | 10.7456 | 10.4803 | 10.365 | 10.5765 | 10.7641 | 10.468 | 10.8176 | 10.7003 | 10.9182 |
| 214741_at | 7690 | ZNF131 | zinc finger protein 131 | -0.5778 | 0.1836 | 0.4322 | 7.7599 | 7.7018 | 7.5375 | 7.088 | 7.7129 | 7.5348 | 7.4967 | 8.609 | 7.4625 | 8.3257 | 8.4566 | 7.6467 |
| 1007_s_at | 780 | DDR1 | discoidin domain receptor tyrosine kinase 1 | -0.5787 | 0.1856 | 0.4322 | 5.7626 | 5.844 | 6.1605 | 6.0338 | 5.8548 | 6.6647 | 6.0903 | 7.7104 | 6.7164 | 5.7924 | 6.3782 | 7.3707 |
| 212206_s_at | 94239 | H2AFV | H2A histone family, member V | -0.5819 | 0.1437 | 0.384 | 7.5507 | 7.6047 | 7.8993 | 7.0033 | 7.4767 | 8.1196 | 7.5658 | 8.124 | 9.2744 | 8.5986 | 7.2382 | 8.9854 |
| 217872_at | 55011 | PIH1D1 | PIH1 domain containing 1 | -0.5831 | 0.1657 | 0.4081 | 6.4458 | 6.63 | 6.5498 | 6.4795 | 7.3089 | 6.661 | 7.3802 | 6.4857 | 7.2735 | 7.6412 | 6.5918 | 7.5507 |
| 207968_s_at | 4208 | MEF2C | myocyte enhancer factor 2C | -0.5865 | 0.08184 | 0.2599 | 5.9356 | 5.8306 | 5.8888 | 6.0162 | 5.4729 | 6.1743 | 6.0438 | 5.9854 | 6.0162 | 6.04 | 6.1777 | 6.2589 |
| 211297_s_at | 1022 | CDK7 | cyclin-dependent kinase 7 | -0.589 | 0.1038 | 0.3095 | 11.7993 | 11.9303 | 11.7544 | 11.7333 | 11.5542 | 11.6252 | 11.7333 | 11.9303 | 11.7993 | 11.8527 | 11.8824 | 12.015 |
| 205349_at | 2769 | GNA15 | guanine nucleotide binding protein (G protein), alpha 15 (Gq class) | -0.59 | 0.1657 | 0.4081 | 5.0889 | 5.0557 | 5.1767 | 4.9353 | 4.841 | 4.7103 | 5.5822 | 4.841 | 6.108 | 6.0314 | 5.005 | 4.8917 |
| 213669_at | 23149 | FCHO1 | FCH domain only 1 | -0.5909 | 0.1078 | 0.3175 | 5.3719 | 4.9134 | 5.4961 | 5.1925 | 4.8145 | 5.5606 | 5.9718 | 5.7815 | 6.5715 | 5.2415 | 5.1881 | 5.5235 |
| 201565_s_at | 3398 | ID2 | inhibitor of DNA binding 2, dominant negative helix-loop-helix protein | -0.5914 | 0.09581 | 0.2901 | 8.6715 | 6.3739 | 6.1227 | 8.6465 | 5.8408 | 6.3339 | 7.437 | 8.1754 | 7.7893 | 7.863 | 8.4785 | 8.1495 |
| 209806_at | 85236 | HIST1H2BK | histone cluster 1, H2bk | -0.5915 | 0.1118 | 0.3244 | 11.8527 | 11.985 | 11.9552 | 11.9552 | 11.3401 | 11.9623 | 12.1519 | 12.1105 | 11.7779 | 11.9623 | 12.3655 | 12.5394 |
| 201763_s_at | 1616 | DAXX | death-domain associated protein | -0.6029 | 0.1896 | 0.4364 | 8.8986 | 8.6965 | 8.4566 | 8.2444 | 8.8898 | 8.5037 | 11.4145 | 8.1034 | 10.9477 | 11.5736 | 8.6611 | 8.0487 |
| 202891_at | 4817 | NIT1 | nitrilase 1 | -0.609 | 0.1477 | 0.3894 | 9.5062 | 9.1101 | 9.7072 | 9.2384 | 9.1012 | 9.1754 | 10.0145 | 9.2627 | 10.3719 | 9.8461 | 9.4724 | 9.3502 |
| 218346_s_at | 27244 | SESN1 | sestrin 1 | -0.6163 | 0.08982 | 0.2771 | 5.4497 | 4.7035 | 5.0832 | 4.7929 | 4.3527 | 4.7929 | 5.0635 | 5.0889 | 5.3205 | 5.3804 | 5.1552 | 5.0557 |
| 206254_at | 1950 | EGF | epidermal growth factor | -0.6174 | 0.07984 | 0.2577 | 5.2783 | 5.4036 | 5.3205 | 5.0733 | 5.3457 | 5.1089 | 5.7707 | 5.2031 | 5.5187 | 5.3955 | 5.6292 | 5.2953 |
| 222103_at | 466 | ATF1 | activating transcription factor 1 | -0.6201 | 0.1317 | 0.3629 | 8.0747 | 7.9413 | 7.994 | 8.3475 | 8.0069 | 7.8894 | 8.0306 | 8.2613 | 8.135 | 8.2999 | 8.2329 | 8.2874 |
| 208905_at | 54205 | CYCS | cytochrome c, somatic | -0.6204 | 0.08583 | 0.2699 | 10.1154 | 10.1771 | 10.4155 | 10.468 | 10.5013 | 10.1993 | 10.3354 | 10.5209 | 10.3867 | 10.5765 | 10.4861 | 10.5491 |
| 209526_s_at | 50810 | HDGFRP3 | hepatoma-derived growth factor, related protein 3 | -0.6351 | 0.07186 | 0.2423 | 7.568 | 7.7545 | 7.2468 | 7.6412 | 7.4155 | 7.3828 | 7.706 | 7.654 | 7.6754 | 7.6827 | 7.7942 | 7.5319 |
| 203073_at | 22796 | COG2 | component of oligomeric golgi complex 2 | -0.6426 | 0.1257 | 0.3554 | 7.2619 | 7.1057 | 6.9763 | 7.3174 | 7.4767 | 7.5348 | 7.0064 | 7.197 | 8.2102 | 7.994 | 7.9732 | 8.0653 |
| 201767_s_at | 60528 | ELAC2 | elaC homolog 2 (E. coli) | -0.6574 | 0.07385 | 0.2474 | 11.0993 | 11.0563 | 10.459 | 10.249 | 10.6659 | 10.5669 | 10.9943 | 10.9582 | 10.8331 | 10.8826 | 11.1208 | 11.0534 |
| 207535_s_at | 4791 | NFKB2 | nuclear factor of kappa light polypeptide gene enhancer in B-cells 2 (p49/p100) | -0.6628 | 0.07984 | 0.2577 | 4.231 | 4.882 | 4.7515 | 4.7103 | 4.9664 | 5.1881 | 4.9409 | 5.2689 | 5.1407 | 4.9353 | 5.0733 | 5.1836 |
| 211919_s_at | 7852 | CXCR4 | chemokine (C-X-C motif) receptor 4 | -0.6643 | 0.06587 | 0.2268 | 5.5688 | 5.338 | 6.6679 | 6.7388 | 5.694 | 6.5948 | 6.6679 | 6.6032 | 7.5971 | 6.5385 | 6.4485 | 6.9731 |
| 212624_s_at | 1123 | CHN1 | chimerin (chimaerin) 1 | -0.6658 | 0.1717 | 0.4145 | 6.3908 | 5.5788 | 5.694 | 5.251 | 5.0861 | 5.424 | 7.8224 | 5.4347 | 8.4221 | 7.8544 | 5.8234 | 5.4117 |
| 221856_s_at | 55793 | FAM63A | family with sequence similarity 63, member A | -0.6666 | 0.09381 | 0.2858 | 6.2346 | 5.9556 | 5.9718 | 6.3205 | 5.9813 | 5.8234 | 6.2546 | 6.2649 | 6.3152 | 6.5745 | 5.9772 | 6.4951 |
| 203675_at | 4925 | NUCB2 | nucleobindin 2 | -0.6669 | 0.07186 | 0.2423 | 11.295 | 11.1592 | 11.2718 | 11.1208 | 11.04 | 11.2124 | 11.247 | 11.5599 | 11.2365 | 11.161 | 11.4145 | 11.6464 |
| 212717_at | 9842 | PLEKHM1 | pleckstrin homology domain containing, family M (with RUN domain) member 1 | -0.6716 | 0.08782 | 0.2744 | 10.6659 | 9.6102 | 10.0145 | 9.6129 | 9.6523 | 9.9558 | 10.0604 | 9.8499 | 11.7544 | 10.7003 | 10.8479 | 10.6417 |
| 203213_at | 983 | CDK1 | cyclin-dependent kinase 1 | -0.6758 | 0.1218 | 0.3482 | 10.365 | 10.2247 | 10.3006 | 10.647 | 10.2533 | 10.3126 | 10.8392 | 10.6074 | 10.4155 | 10.9582 | 10.6074 | 10.3006 |
| 203566_s_at | 178 | AGL | amylo-alpha-1, 6-glucosidase, 4-alpha-glucanotransferase | -0.6788 | 0.0519 | 0.1967 | 6.3826 | 6.4004 | 6.6264 | 6.6074 | 6.224 | 6.794 | 6.6628 | 6.729 | 7.4743 | 6.7086 | 6.5622 | 7.2549 |
| 205153_s_at | 958 | CD40 | CD40 molecule, TNF receptor superfamily member 5 | -0.6824 | 0.08383 | 0.2645 | 8.4496 | 8.181 | 8.7006 | 7.5108 | 7.8447 | 7.7634 | 9.8994 | 7.7634 | 11.5857 | 11.5382 | 8.5719 | 7.9829 |
| 218168_s_at | 56997 | ADCK3 | aarF domain containing kinase 3 | -0.683 | 0.1078 | 0.3175 | 3.568 | 3.2386 | 3.2386 | 3.4609 | 3.0296 | 3.0296 | 3.9016 | 3.8792 | 3.6316 | 3.8792 | 3.2386 | 3.2386 |
| 203201_at | 5373 | PMM2 | phosphomannomutase 2 | -0.6879 | 0.08982 | 0.2771 | 10.7157 | 10.5669 | 11.9623 | 10.3354 | 9.7678 | 10.8331 | 10.8639 | 10.908 | 12.983 | 12.0358 | 11.7005 | 11.9552 |
| 203521_s_at | 24149 | ZNF318 | zinc finger protein 318 | -0.6951 | 0.07585 | 0.2506 | 8.6205 | 7.3914 | 10.6137 | 11.4145 | 8.5629 | 10.9335 | 11.8192 | 11.7715 | 12.6867 | 9.2726 | 11.2365 | 13.8177 |
| 202466_at | 11044 | PAPD7 | PAP associated domain containing 7 | -0.6952 | 0.0499 | 0.1937 | 8.5534 | 8.3227 | 9.0984 | 8.5177 | 8.3883 | 7.91 | 9.9273 | 8.5807 | 10.7988 | 8.8784 | 8.8852 | 8.901 |
| 212765_at | 23271 | CAMSAP2 | calmodulin regulated spectrin-associated protein family, member 2 | -0.7023 | 0.06188 | 0.2169 | 9.8461 | 10.1514 | 9.9746 | 9.1436 | 9.9038 | 10.0183 | 11.4675 | 9.9424 | 11.5542 | 11.0534 | 9.7119 | 10.1993 |
| 221449_s_at | 81533 | ITFG1 | integrin alpha FG-GAP repeat containing 1 | -0.7087 | 0.02196 | 0.1294 | 7.1782 | 7.5876 | 7.6358 | 7.6669 | 5.101 | 7.296 | 7.989 | 7.7314 | 8.0559 | 7.8858 | 8.0884 | 7.6616 |
| 204191_at | 3454 | IFNAR1 | interferon (alpha, beta and omega) receptor 1 | -0.7115 | 0.07784 | 0.2546 | 6.9988 | 7.1002 | 7.3028 | 6.9731 | 6.8428 | 6.9458 | 8.1992 | 7.1166 | 9.1101 | 8.0559 | 6.8421 | 7.1843 |
| 213721_at | 6657 | SOX2 | SRY (sex determining region Y)-box 2 | -0.7183 | 0.04591 | 0.1871 | 7.2378 | 7.2999 | 7.0691 | 6.6738 | 6.5559 | 6.34 | 7.4055 | 7.2156 | 8.1404 | 7.2895 | 7.1323 | 7.2813 |
| 203358_s_at | 2146 | EZH2 | enhancer of zeste homolog 2 (Drosophila) | -0.7208 | 0.07385 | 0.2474 | 8.3257 | 8.0069 | 7.7129 | 7.8447 | 7.7356 | 8.0238 | 7.9069 | 8.4548 | 8.206 | 8.1393 | 8.4221 | 8.5534 |
| 204985_s_at | 79090 | TRAPPC6A | trafficking protein particle complex 6A | -0.7254 | 0.07585 | 0.2506 | 6.8435 | 6.6115 | 7.1057 | 6.9731 | 6.8292 | 6.8095 | 7.6616 | 6.6365 | 7.8993 | 7.2378 | 7.2869 | 7.0974 |
| 212899_at | 23097 | CDK19 | cyclin-dependent kinase 19 | -0.7314 | 0.02395 | 0.1331 | 7.5894 | 7.5755 | 7.3777 | 7.3569 | 7.3707 | 7.5484 | 7.6358 | 7.6906 | 7.3678 | 9.5336 | 8.0069 | 8.5986 |
| 201887_at | 3597 | IL13RA1 | interleukin 13 receptor, alpha 1 | -0.7316 | 0.07984 | 0.2577 | 8.7049 | 8.0939 | 8.2102 | 8.1495 | 8.0238 | 8.2196 | 9.6412 | 8.4093 | 8.6307 | 9.2787 | 9.2702 | 7.989 |
| 203338_at | 5529 | PPP2R5E | protein phosphatase 2, regulatory subunit B', epsilon isoform | -0.7361 | 0.08782 | 0.2744 | 9.045 | 8.941 | 8.9937 | 9.1936 | 8.9134 | 9.0407 | 9.1012 | 9.2051 | 9.2487 | 8.9937 | 9.3614 | 9.6153 |
| 205067_at | 3553 | IL1B | interleukin 1, beta | -0.7391 | 0.08184 | 0.2599 | 5.8234 | 5.6241 | 6.2114 | 6.0438 | 5.7707 | 5.7707 | 6.2743 | 5.9556 | 6.7367 | 6.3908 | 5.9556 | 6.1874 |
| 218350_s_at | 51053 | GMNN | geminin, DNA replication inhibitor | -0.7416 | 0.0479 | 0.1889 | 12.4521 | 12.3797 | 12.2657 | 12.1907 | 12.2657 | 12.7014 | 13.4075 | 12.5394 | 13.9002 | 13.1644 | 12.4521 | 12.3655 |
| 213689_x_at | 388650 | FAM69A | family with sequence similarity 69, member A | -0.744 | 0.0519 | 0.1967 | 10.2737 | 10.4304 | 10.8176 | 6.1582 | 7.2378 | 10.6815 | 11.0282 | 10.6137 | 11.8741 | 11.1114 | 10.8176 | 11.1208 |
| 203465_at | 9801 | MRPL19 | mitochondrial ribosomal protein L19 | -0.7445 | 0.05988 | 0.213 | 10.633 | 10.3818 | 10.3695 | 10.3917 | 10.7003 | 10.232 | 10.4155 | 10.6708 | 10.7417 | 10.6815 | 10.8911 | 10.8826 |
| 202847_at | 5106 | PCK2 | phosphoenolpyruvate carboxykinase 2 (mitochondrial) | -0.7501 | 0.0519 | 0.1967 | 7.17 | 7.2869 | 7.4718 | 7.2291 | 6.8101 | 7.1596 | 7.95 | 7.088 | 7.8136 | 8.135 | 7.509 | 7.3707 |
| 219384_s_at | 23536 | ADAT1 | adenosine deaminase, tRNA-specific 1 | -0.7533 | 0.04391 | 0.1812 | 6.1558 | 6.0639 | 6.3704 | 6.2743 | 6.3205 | 6.3908 | 6.6943 | 6.2156 | 7.4661 | 6.868 | 6.618 | 6.3178 |
| 203418_at | 890 | CCNA2 | cyclin A2 | -0.7541 | 0.07984 | 0.2577 | 9.4881 | 9.5867 | 9.5548 | 9.644 | 9.6153 | 9.7626 | 9.7752 | 9.9558 | 9.7268 | 9.5221 | 9.9902 | 9.8854 |
| 202052_s_at | 26064 | RAI14 | retinoic acid induced 14 | -0.7592 | 0.0479 | 0.1889 | 11.3791 | 10.9477 | 11.0778 | 11.1997 | 10.9943 | 11.2899 | 11.7111 | 11.1393 | 12.1519 | 11.5185 | 11.247 | 11.5382 |
| 202889_x_at | 9053 | MAP7 | microtubule-associated protein 7 | -0.7594 | 0.007984 | 0.0805 | 4.1498 | 4.0809 | 4.0273 | 3.9717 | 3.5043 | 3.7758 | 4.8917 | 4.2483 | 4.8567 | 4.6041 | 14.1383 | 13.6591 |
| 203277_at | 1676 | DFFA | DNA fragmentation factor, 45kDa, alpha polypeptide | -0.7595 | 0.03792 | 0.1656 | 7.5099 | 7.5151 | 7.8329 | 7.2722 | 7.2747 | 7.7018 | 7.7634 | 7.6047 | 8.5629 | 8.1256 | 7.9809 | 7.7018 |
| 206688_s_at | 10898 | CPSF4 | cleavage and polyadenylation specific factor 4, 30kDa | -0.7597 | 0.02994 | 0.1457 | 8.7883 | 8.8424 | 8.9954 | 9.0242 | 8.4749 | 9.1834 | 9.2979 | 8.9476 | 10.1099 | 9.3137 | 9.1012 | 9.5056 |
| 218744_s_at | 29763 | PACSIN3 | protein kinase C and casein kinase substrate in neurons 3 | -0.7605 | 0.05389 | 0.2012 | 5.9994 | 6.0903 | 6.2743 | 6.1777 | 5.9813 | 5.8629 | 6.4672 | 6.2018 | 6.6796 | 6.5065 | 6.4888 | 5.934 |
| 208478_s_at | 581 | BAX | BCL2-associated X protein | -0.7607 | 0.01597 | 0.11 | 8.181 | 8.0444 | 8.27 | 8.0653 | 7.7756 | 7.7756 | 8.7387 | 8.254 | 10.8639 | 8.6398 | 8.3475 | 8.6465 |
| 201967_at | 10180 | RBM6 | RNA binding motif protein 6 | -0.7616 | 0.05988 | 0.213 | 7.8976 | 7.5894 | 8.1545 | 8.3743 | 7.6283 | 7.994 | 8.27 | 8.2999 | 8.5903 | 7.937 | 8.5389 | 8.9175 |
| 203258_at | 10589 | DRAP1 | DR1-associated protein 1 (negative cofactor 2 alpha) | -0.7639 | 0.01796 | 0.1156 | 11.1208 | 11.2365 | 11.2169 | 11.2169 | 11.3254 | 11.2169 | 11.3882 | 11.8922 | 11.2899 | 11.3002 | 11.3791 | 11.4145 |
| 200789_at | 1891 | ECH1 | enoyl CoA hydratase 1, peroxisomal | -0.764 | 0.02994 | 0.1457 | 8.5346 | 7.9728 | 7.5154 | 8.1122 | 8.3903 | 8.5264 | 9.4319 | 8.6032 | 10.0766 | 9.2928 | 8.0403 | 8.714 |
| 218457_s_at | 1788 | DNMT3A | DNA (cytosine-5-)-methyltransferase 3 alpha | -0.7655 | 0.05589 | 0.207 | 7.6684 | 8.0306 | 8.3894 | 8.0047 | 7.9809 | 7.91 | 8.9532 | 7.8578 | 9.6102 | 9.3602 | 8.2519 | 8.2366 |
| 200636_s_at | 5792 | PTPRF | protein tyrosine phosphatase, receptor type, F | -0.7659 | 0.03593 | 0.1619 | 8.0747 | 7.0204 | 7.5461 | 7.7599 | 6.8804 | 7.4743 | 9.3073 | 7.6827 | 10.1718 | 8.6827 | 7.7314 | 7.8773 |
| 218149_s_at | 55893 | ZNF395 | zinc finger protein 395 | -0.7685 | 0.0519 | 0.1967 | 9.0166 | 8.2587 | 9.1936 | 7.9194 | 7.6989 | 7.8136 | 11.7993 | 8.4824 | 13.1644 | 12.015 | 8.7978 | 8.3827 |
| 209467_s_at | 8569 | MKNK1 | MAP kinase interacting serine/threonine kinase 1 | -0.7733 | 0.02994 | 0.1457 | 8.2669 | 8.587 | 8.0939 | 8.5389 | 8.1574 | 8.2736 | 10.0849 | 8.5504 | 8.6307 | 9.2051 | 8.7093 | 8.5064 |
| 210761_s_at | 2886 | GRB7 | growth factor receptor-bound protein 7 | -0.7745 | 0.06188 | 0.2169 | 9.5434 | 10.5815 | 10.5734 | 9.3465 | 9.3848 | 9.4687 | 14.1383 | 9.3913 | 14.1383 | 13.6034 | 10.7641 | 9.8994 |
| 212851_at | 23142 | DCUN1D4 | DCN1, defective in cullin neddylation 1, domain containing 4 (S. cerevisiae) | -0.7757 | 0.03593 | 0.1619 | 7.9514 | 8.0196 | 8.3198 | 8.2999 | 8.1338 | 8.2736 | 8.3894 | 8.5211 | 8.3263 | 8.3872 | 8.6465 | 8.1893 |
| 209257_s_at | 9126 | SMC3 | structural maintenance of chromosomes 3 | -0.7764 | 0.0479 | 0.1889 | 8.4749 | 8.2845 | 7.3226 | 8.2527 | 7.8329 | 7.4609 | 8.4093 | 8.2824 | 8.901 | 8.2587 | 8.4824 | 8.6279 |
| 202720_at | 26136 | TES | testis derived transcript (3 LIM domains) | -0.7779 | 0.007984 | 0.0805 | 6.1255 | 11.0282 | 11.1997 | 11.0778 | 10.6417 | 11.247 | 12.1267 | 12.0613 | 13.0802 | 12.2931 | 11.5032 | 12.0613 |
| 336_at | 6915 | TBXA2R | thromboxane A2 receptor | -0.7852 | 0.06387 | 0.2207 | 7.5056 | 7.3385 | 7.6653 | 7.4254 | 7.5703 | 7.2378 | 8.2519 | 7.2468 | 8.5091 | 8.0884 | 7.6467 | 7.8588 |
| 218755_at | 10112 | KIF20A | kinesin family member 20A | -0.7881 | 0.007984 | 0.0805 | 8.9854 | 6.1139 | 8.8371 | 8.8986 | 8.9476 | 8.941 | 9.5952 | 9.922 | 9.6861 | 9.0829 | 9.4687 | 10.1771 |
| 203659_s_at | 10206 | TRIM13 | tripartite motif containing 13 | -0.7901 | 0.02994 | 0.1457 | 7.0631 | 6.661 | 7.6684 | 6.8292 | 6.9458 | 7.4474 | 8.0669 | 7.296 | 9.3037 | 7.706 | 7.7599 | 7.632 |
| 200059_s_at | 387 | RHOA | ras homolog gene family, member A | -0.7995 | 0.06387 | 0.2207 | 8.8163 | 8.5944 | 8.6465 | 8.855 | 8.7838 | 8.9522 | 9.2051 | 9.1754 | 8.609 | 9.0276 | 9.2134 | 9.1881 |
| 203119_at | 79080 | CCDC86 | coiled-coil domain containing 86 | -0.8012 | 0.03992 | 0.1705 | 10.908 | 10.8176 | 10.5815 | 10.8826 | 10.8868 | 10.6659 | 11.161 | 10.9182 | 11.1997 | 10.8639 | 10.8639 | 11.1746 |
| 203952_at | 22926 | ATF6 | activating transcription factor 6 | -0.8085 | 0.05589 | 0.207 | 8.4824 | 8.4075 | 8.5629 | 8.7482 | 8.5807 | 8.2999 | 8.8371 | 8.9954 | 9.2709 | 8.8705 | 8.3407 | 9.0166 |
| 202801_at | 5566 | PRKACA | protein kinase, cAMP-dependent, catalytic, alpha | -0.8134 | 0.02196 | 0.1294 | 9.1707 | 9.2709 | 8.8163 | 8.6336 | 9.2787 | 9.4438 | 9.2787 | 9.3977 | 11.0671 | 9.8854 | 9.7275 | 9.8804 |
| 219127_at | 79170 | PRR15L | proline rich 15-like | -0.8155 | 0.03792 | 0.1656 | 5.5976 | 5.0492 | 5.6698 | 5.694 | 5.005 | 5.4961 | 6.4485 | 5.5976 | 6.9387 | 6.4458 | 5.6421 | 5.6982 |
| 201710_at | 4605 | MYBL2 | v-myb myeloblastosis viral oncogene homolog (avian)-like 2 | -0.819 | 0.02994 | 0.1457 | 5.8234 | 6.3205 | 6.256 | 5.9745 | 6.1958 | 6.2197 | 6.3739 | 6.3516 | 6.794 | 6.3826 | 6.2743 | 6.4548 |
| 207515_s_at | 9533 | POLR1C | polymerase (RNA) I polypeptide C, 30kDa | -0.8228 | 0.02994 | 0.1457 | 7.8184 | 8.3903 | 8.0829 | 7.6219 | 8.2369 | 8.2149 | 8.8761 | 8.2149 | 9.3465 | 8.7978 | 8.2669 | 8.4293 |
| 205051_s_at | 3815 | KIT | v-kit Hardy-Zuckerman 4 feline sarcoma viral oncogene homolog | -0.8256 | 0.0479 | 0.1889 | 5.1925 | 4.5075 | 5.6421 | 6.1255 | 4.4114 | 6.1605 | 6.8106 | 7.4625 | 7.8544 | 5.0596 | 6.34 | 8.9522 |
| 207034_s_at | 2736 | GLI2 | GLI family zinc finger 2 | -0.826 | 0.05389 | 0.2012 | 4.9298 | 5.101 | 5.0635 | 4.6498 | 4.9889 | 5.338 | 5.0402 | 5.4539 | 5.73 | 5.4539 | 5.1881 | 5.5822 |
| 202396_at | 10915 | TCERG1 | transcription elongation regulator 1 | -0.8287 | 0.04391 | 0.1812 | 9.148 | 9.8902 | 8.5211 | 10.0604 | 8.9263 | 8.6173 | 9.7752 | 9.9746 | 9.6384 | 9.8403 | 9.9764 | 9.8141 |
| 202900_s_at | 4927 | NUP88 | nucleoporin 88kDa | -0.8309 | 0.007984 | 0.0805 | 9.7338 | 9.5248 | 9.644 | 9.684 | 9.4533 | 9.7072 | 9.9167 | 9.7825 | 9.8248 | 9.6968 | 10.1993 | 10.633 |
| 214061_at | 93594 | WDR67 | WD repeat domain 67 | -0.8312 | 0.02595 | 0.141 | 8.2348 | 7.8329 | 8.2845 | 8.8233 | 8.8963 | 8.5782 | 9.0099 | 8.7568 | 10.0183 | 9.039 | 8.8963 | 9.1722 |
| 204788_s_at | 5498 | PPOX | protoporphyrinogen oxidase | -0.8346 | 0.02196 | 0.1294 | 8.7702 | 8.7161 | 8.6205 | 8.0939 | 8.1223 | 8.1908 | 10.6837 | 8.4785 | 12.0358 | 11.7544 | 8.6715 | 8.6896 |
| 218584_at | 79600 | TCTN1 | tectonic family member 1 | -0.8355 | 0.01796 | 0.1156 | 6.855 | 6.7227 | 6.6336 | 6.8106 | 6.3064 | 6.8779 | 6.9731 | 6.6738 | 7.863 | 7.5755 | 7.042 | 7.32 |
| 208820_at | 5747 | PTK2 | PTK2 protein tyrosine kinase 2 | -0.8428 | 0.01397 | 0.1059 | 10.7456 | 10.8392 | 10.8911 | 10.9477 | 10.2889 | 10.7601 | 11.7715 | 10.8911 | 11.6464 | 11.5656 | 10.9837 | 10.8911 |
| 204466_s_at | 6622 | SNCA | synuclein, alpha (non A4 component of amyloid precursor) | -0.8489 | 0.01796 | 0.1156 | 8.941 | 9.4533 | 9.3768 | 9.3223 | 9.2368 | 9.3073 | 9.5676 | 9.2979 | 9.8994 | 9.7929 | 9.5801 | 9.498 |
| 212880_at | 23335 | WDR7 | WD repeat domain 7 | -0.8513 | 0.01198 | 0.09843 | 7.1166 | 7.0095 | 7.2468 | 7.0936 | 6.7764 | 7.2722 | 7.4767 | 7.3997 | 8.2368 | 7.5885 | 7.3439 | 7.2291 |
| 202678_at | 2958 | GTF2A2 | general transcription factor IIA, 2, 12kDa | -0.8551 | 0.02395 | 0.1331 | 11.2124 | 11.2899 | 11.2365 | 11.0993 | 11.1208 | 11.1592 | 11.3002 | 11.451 | 11.2169 | 11.2558 | 11.5857 | 11.3882 |
| 201136_at | 5355 | PLP2 | proteolipid protein 2 (colonic epithelium-enriched) | -0.8553 | 0.01198 | 0.09843 | 8.7093 | 8.0559 | 8.5264 | 8.4241 | 8.2527 | 8.5944 | 9.9038 | 8.7304 | 9.4533 | 9.1012 | 8.7482 | 8.5264 |
| 213135_at | 7074 | TIAM1 | T-cell lymphoma invasion and metastasis 1 | -0.8558 | 0.01198 | 0.09843 | 5.3097 | 5.231 | 4.8724 | 5.0949 | 4.7256 | 4.7103 | 5.1287 | 4.9216 | 9.7119 | 9.7678 | 9.6153 | 5.1552 |
| 202095_s_at | 332 | BIRC5 | baculoviral IAP repeat containing 5 | -0.8655 | 0.03393 | 0.1558 | 9.8293 | 10.1666 | 10.042 | 10.3293 | 10.1993 | 10.2889 | 10.4396 | 10.5353 | 10.2948 | 10.2384 | 10.633 | 10.8639 |
| 212180_at | 1399 | CRKL | v-crk sarcoma virus CT10 oncogene homolog (avian)-like | -0.8745 | 0.02794 | 0.1457 | 7.716 | 8.7568 | 9.2559 | 9.3137 | 7.6635 | 7.9299 | 10.923 | 8.8963 | 11.3122 | 10.7641 | 9.1737 | 9.1834 |
| 202916_s_at | 9917 | FAM20B | family with sequence similarity 20, member B | -0.8767 | 0.02196 | 0.1294 | 7.5569 | 7.6219 | 7.8858 | 7.8136 | 7.2549 | 7.9732 | 8.0403 | 8.4583 | 8.5144 | 7.9258 | 7.7832 | 8.3827 |
| 204654_s_at | 7020 | TFAP2A | transcription factor AP-2 alpha (activating enhancer binding protein 2 alpha) | -0.887 | 0.007984 | 0.0805 | 8.7786 | 8.7387 | 8.9824 | 7.7998 | 8.5629 | 8.7568 | 9.0829 | 8.9817 | 9.8461 | 9.2487 | 9.0407 | 9.3202 |
| 203575_at | 1459 | CSNK2A2 | casein kinase 2, alpha prime polypeptide | -0.8905 | 0.04591 | 0.1871 | 6.8095 | 7.1423 | 7.168 | 7.6616 | 6.7515 | 7.4661 | 8.4785 | 7.1241 | 8.0884 | 7.8993 | 8.0606 | 7.6906 |
| 201087_at | 5829 | PXN | paxillin | -0.8935 | 0.03194 | 0.1524 | 11.985 | 11.8403 | 12.0242 | 12.1519 | 11.9142 | 12.3315 | 12.7014 | 12.3655 | 12.7892 | 12.1784 | 12.1267 | 12.4808 |
| 208407_s_at | 1500 | CTNND1 | catenin (cadherin-associated protein), delta 1 | -0.8937 | 0.02595 | 0.141 | 8.4924 | 8.1992 | 9.1754 | 8.3092 | 7.6989 | 8.3898 | 9.4486 | 8.9134 | 10.3671 | 9.1224 | 8.7568 | 9.2911 |
| 204106_at | 7016 | TESK1 | testis-specific kinase 1 | -0.9121 | 0.03393 | 0.1558 | 9.0602 | 9.1348 | 10.0849 | 9.2695 | 8.6794 | 9.4306 | 10.8826 | 9.6523 | 11.451 | 10.5686 | 9.5248 | 10.1926 |
| 34408_at | 6253 | RTN2 | reticulon 2 | -0.9184 | 0.00998 | 0.08955 | 9.3465 | 7.7832 | 9.6206 | 8.9854 | 8.8432 | 9.2702 | 10.3818 | 9.4687 | 11.4798 | 10.1514 | 9.9038 | 9.8499 |
| 210145_at | 5321 | PLA2G4A | phospholipase A2, group IVA (cytosolic, calcium-dependent) | -0.9461 | 0.01198 | 0.09843 | 9.2368 | 9.4001 | 9.8432 | 8.6896 | 9.18 | 8.8807 | 9.9918 | 9.7268 | 11.8192 | 11.1592 | 10.1251 | 9.6671 |
| 209747_at | 7043 | TGFB3 | transforming growth factor, beta 3 | -0.9493 | 0.02794 | 0.1457 | 5.6292 | 5.6241 | 6.2812 | 5.4961 | 5.3804 | 5.8835 | 5.8888 | 6.3205 | 7.3174 | 6.618 | 6.1139 | 6.8435 |
| 201995_at | 2131 | EXT1 | exostosin 1 | -0.965 | 0.02395 | 0.1331 | 10.9943 | 10.6659 | 11.161 | 10.4155 | 10.3209 | 10.3719 | 11.8824 | 11.0563 | 12.417 | 11.6464 | 11.1114 | 11.0671 |
| 222217_s_at | 11000 | SLC27A3 | solute carrier family 27 (fatty acid transporter), member 3 | -0.9744 | 0.00998 | 0.08955 | 7.9514 | 8.1034 | 8.5629 | 7.8773 | 7.8729 | 8.0829 | 10.3719 | 8.3629 | 10.653 | 11.2417 | 8.2196 | 8.7568 |
| 218215_s_at | 7376 | NR1H2 | nuclear receptor subfamily 1, group H, member 2 | -0.9753 | 0.007984 | 0.0805 | 6.5385 | 6.1958 | 6.5745 | 6.492 | 6.4052 | 6.4951 | 7.0204 | 9.1101 | 7.2735 | 7.2813 | 6.9943 | 6.8018 |
| 206109_at | 2523 | FUT1 | fucosyltransferase 1 (galactoside 2-alpha-L-fucosyltransferase, H blood group) | -0.9957 | 0.01597 | 0.11 | 6.2156 | 5.8835 | 6.5385 | 6.4052 | 5.7815 | 6.1874 | 7.2895 | 6.4672 | 7.9299 | 6.9731 | 6.6647 | 6.661 |
| 201697_s_at | 1786 | DNMT1 | DNA (cytosine-5-)-methyltransferase 1 | -1.0008 | 0.02794 | 0.1457 | 11.8298 | 11.9142 | 12.0869 | 11.7993 | 11.8527 | 12.1105 | 12.3797 | 12.417 | 13.2128 | 13.0242 | 11.8741 | 12.417 |
| 205036_at | 11157 | LSM6 | LSM6 homolog, U6 small nuclear RNA associated (S. cerevisiae) | -1.0022 | 0.02395 | 0.1331 | 7.9299 | 7.9413 | 8.1415 | 7.9869 | 8.1495 | 8.181 | 8.4221 | 8.5629 | 8.4038 | 8.1992 | 8.4698 | 8.0684 |
| 201649_at | 9246 | UBE2L6 | ubiquitin-conjugating enzyme E2L 6 | -1.0104 | 0.007984 | 0.0805 | 10.972 | 10.8479 | 10.7988 | 10.8176 | 10.4861 | 11.0778 | 11.7005 | 11.1857 | 11.7715 | 11.1997 | 10.9582 | 11.3254 |
| 206752_s_at | 1677 | DFFB | DNA fragmentation factor, 40kDa, beta polypeptide (caspase-activated DNase) | -1.0181 | 0.01996 | 0.1243 | 6.3631 | 5.9994 | 6.2618 | 5.9959 | 5.9356 | 5.9503 | 6.4262 | 6.4262 | 7.0095 | 6.4262 | 6.3782 | 6.4292 |
| 206723_s_at | 9170 | LPAR2 | lysophosphatidic acid receptor 2 | -1.026 | 0.01198 | 0.09843 | 6.0363 | 6.4322 | 6.9308 | 6.718 | 5.8928 | 6.988 | 7.4055 | 6.7388 | 8.4496 | 7.2245 | 7.7386 | 7.9529 |
| 205573_s_at | 51375 | SNX7 | sorting nexin 7 | -1.0274 | 0.03593 | 0.1619 | 8.5037 | 8.2196 | 7.6563 | 8.3155 | 7.9031 | 8.2824 | 9.5469 | 10.468 | 7.4155 | 9.9902 | 9.9633 | 10.435 |
| 201896_s_at | 84722 | PSRC1 | proline/serine-rich coiled-coil 1 | -1.0426 | 0.01597 | 0.11 | 8.5091 | 8.3227 | 8.9522 | 8.2196 | 8.5037 | 7.8447 | 9.2787 | 9.684 | 9.8141 | 8.8784 | 8.5629 | 10.0829 |
| 209083_at | 11151 | CORO1A | coronin, actin binding protein, 1A | -1.0508 | 0.03593 | 0.1619 | 6.2589 | 7.4254 | 6.3704 | 6.7713 | 7.6101 | 6.7515 | 8.4924 | 8.6827 | 9.9902 | 8.4221 | 6.729 | 9.0407 |
| 211518_s_at | 652 | BMP4 | bone morphogenetic protein 4 | -1.0598 | 0.00998 | 0.08955 | 6.0115 | 5.9356 | 6.3957 | 5.8782 | 5.6421 | 6.0728 | 7.0064 | 6.2156 | 7.8224 | 6.718 | 6.3361 | 7.1996 |
| 202763_at | 836 | CASP3 | caspase 3, apoptosis-related cysteine peptidase | -1.0613 | 0.01397 | 0.1059 | 8.2527 | 8.5834 | 8.7812 | 8.3958 | 8.3465 | 8.6965 | 8.9476 | 8.8266 | 9.3333 | 8.904 | 9.9558 | 10.2447 |
| 219581_at | 80746 | TSEN2 | tRNA splicing endonuclease 2 homolog (S. cerevisiae) | -1.0637 | 0.01796 | 0.1156 | 7.9915 | 8.1754 | 7.9685 | 8.1256 | 8.0774 | 8.1062 | 8.2613 | 8.439 | 8.3894 | 8.1034 | 8.3903 | 8.7568 |
| 203233_at | 3566 | IL4R | interleukin 4 receptor | -1.0704 | 0.00998 | 0.08955 | 5.8888 | 5.7924 | 6.0709 | 5.5688 | 5.4961 | 5.6421 | 7.1111 | 5.9059 | 7.4254 | 6.5126 | 6.2066 | 6.3631 |
| 212997_s_at | 11011 | TLK2 | tousled-like kinase 2 | -1.0733 | 0.01198 | 0.09843 | 8.3111 | 7.8578 | 7.9869 | 8.3883 | 7.95 | 8.135 | 8.7786 | 8.1602 | 9.24 | 8.4785 | 8.855 | 9.3055 |
| 203246_s_at | 10641 | NPRL2 | nitrogen permease regulator-like 2 (S. cerevisiae) | -1.091 | 0.007984 | 0.0805 | 6.9656 | 6.9009 | 7.0491 | 7.0355 | 6.988 | 7.0974 | 7.8184 | 7.3569 | 8.1574 | 8.3743 | 7.1638 | 7.1111 |
| 212968_at | 5986 | RFNG | RFNG O-fucosylpeptide 3-beta-N-acetylglucosaminyltransferase | -1.1276 | 0.007984 | 0.0805 | 9.2093 | 9.3768 | 9.7119 | 9.3635 | 9.2744 | 9.4224 | 10.1993 | 9.8994 | 11.6793 | 10.1099 | 9.7268 | 10.9582 |
| 205633_s_at | 211 | ALAS1 | aminolevulinate, delta-, synthase 1 | -1.1367 | 0.007984 | 0.0805 | 11.669 | 11.4259 | 11.8527 | 11.8741 | 11.247 | 11.8192 | 12.0613 | 12.1907 | 12.7437 | 12.1519 | 12.0613 | 12.1907 |
| 200024_at | 6193 | RPS5 | ribosomal protein S5 | -1.1457 | 0.03393 | 0.1558 | 6.4052 | 5.4539 | 5.6058 | 5.4454 | 5.0557 | 5.3457 | 7.437 | 6.5529 | 5.6292 | 7.1241 | 7.0781 | 7.075 |
| 203256_at | 1001 | CDH3 | cadherin 3, type 1, P-cadherin (placental) | -1.1608 | 0.01796 | 0.1156 | 4.5854 | 4.5854 | 4.5854 | 4.9353 | 4.8724 | 5.0447 | 5.3955 | 5.1881 | 5.3418 | 5.5478 | 5.1552 | 4.9298 |
| 219270_at | 79094 | CHAC1 | ChaC, cation transport regulator homolog 1 (E. coli) | -1.1845 | 0.01597 | 0.11 | 8.907 | 8.9854 | 9.5193 | 9.4001 | 9.2709 | 9.2384 | 9.6102 | 10.1771 | 10.5815 | 9.8644 | 9.4825 | 10.3818 |
| 217825_s_at | 51465 | UBE2J1 | ubiquitin-conjugating enzyme E2, J1, U | -1.2023 | 0.01397 | 0.1059 | 7.3854 | 7.7499 | 8.2196 | 7.2156 | 7.4527 | 8.0829 | 8.582 | 8.254 | 8.8898 | 9.4533 | 8.4566 | 8.4785 |
| 203919_at | 6919 | TCEA2 | transcription elongation factor A (SII), 2 | -1.2206 | 0.01597 | 0.11 | 7.1057 | 6.9531 | 7.5154 | 6.5498 | 7.1885 | 7.6283 | 8.1545 | 8.0238 | 9.4001 | 8.2149 | 7.798 | 8.3111 |
| 221308_at | 10818 | FRS2 | fibroblast growth factor receptor substrate 2 | -1.2231 | 0.007984 | 0.0805 | 5.9718 | 6.34 | 6.6394 | 6.3476 | 6.5147 | 6.9308 | 6.9763 | 8.0559 | 8.181 | 6.9943 | 7.4434 | 8.7978 |
| 203755_at | 701 | BUB1B | budding uninhibited by benzimidazoles 1 homolog beta (yeast) | -1.2295 | 0.007984 | 0.0805 | 9.6153 | 9.6882 | 10.0565 | 10.1771 | 9.2487 | 10.042 | 11.0534 | 10.647 | 11.7221 | 10.3126 | 10.4209 | 11.247 |
| 203927_at | 4794 | NFKBIE | nuclear factor of kappa light polypeptide gene enhancer in B-cells inhibitor, epsilon | -1.239 | 0.007984 | 0.0805 | 5.251 | 5.5606 | 5.7025 | 5.4539 | 5.5373 | 5.614 | 5.9556 | 6.3383 | 7.0355 | 6.1874 | 5.694 | 6.661 |
| 203685_at | 596 | BCL2 | B-cell CLL/lymphoma 2 | -1.2684 | 0.007984 | 0.0805 | 3.2386 | 3.8529 | 4.1776 | 4.1776 | 3.4175 | 4.2967 | 4.6715 | 5.4729 | 6.0314 | 4.9592 | 4.7515 | 6.661 |
| 203110_at | 2185 | PTK2B | PTK2B protein tyrosine kinase 2 beta | -1.282 | 0.007984 | 0.0805 | 6.7713 | 6.741 | 6.6115 | 6.4672 | 6.5715 | 6.5385 | 7.4877 | 7.2386 | 8.3782 | 7.3226 | 7.1002 | 6.9655 |
| 203285_s_at | 9653 | HS2ST1 | heparan sulfate 2-O-sulfotransferase 1 | -1.2826 | 0.00998 | 0.08955 | 6.0314 | 6.0438 | 6.0338 | 5.8969 | 6.2805 | 6.0527 | 6.1255 | 6.5405 | 6.887 | 6.6032 | 6.4733 | 6.5498 |
| 201614_s_at | 8607 | RUVBL1 | RuvB-like 1 (E. coli) | -1.3089 | 0.01597 | 0.11 | 8.8898 | 8.8314 | 9.1423 | 9.0717 | 9.0407 | 9.3223 | 9.3223 | 9.4187 | 9.4462 | 9.4116 | 9.3333 | 9.631 |
| 201980_s_at | 6251 | RSU1 | Ras suppressor protein 1 | -1.3207 | 0.01198 | 0.09843 | 7.2268 | 7.2619 | 7.2043 | 7.1221 | 6.9308 | 6.9009 | 7.2095 | 7.7356 | 8.1923 | 7.6947 | 7.6947 | 8.0047 |
| 201531_at | 7538 | ZFP36 | zinc finger protein 36, C3H type, homolog (mouse) | -1.3241 | 0.007984 | 0.0805 | 8.4418 | 8.7304 | 8.6794 | 8.8039 | 8.3743 | 8.6279 | 8.907 | 9.7541 | 10.6074 | 9.2894 | 9.2928 | 9.9103 |
| 200053_at | 9552 | SPAG7 | sperm associated antigen 7 | -1.3502 | 0.02395 | 0.1331 | 8.2081 | 8.1291 | 8.5389 | 8.3958 | 8.109 | 8.1415 | 8.4583 | 8.7598 | 8.9896 | 8.7049 | 8.6545 | 8.7752 |
| 209464_at | 9212 | AURKB | aurora kinase B | -1.3669 | 0.00998 | 0.08955 | 7.9069 | 8.0281 | 8.5177 | 7.3047 | 7.6563 | 7.989 | 9.4533 | 9.8902 | 10.8479 | 8.2553 | 9.3223 | 10.0527 |
| 218170_at | 51015 | ISOC1 | isochorismatase domain containing 1 | -1.3767 | 0.01996 | 0.1243 | 8.0306 | 8.7752 | 8.4362 | 7.9728 | 7.8568 | 7.7961 | 8.6827 | 8.8501 | 8.9532 | 8.7838 | 9.1101 | 9.1348 |
| 205450_at | 5255 | PHKA1 | phosphorylase kinase, alpha 1 (muscle) | -1.4173 | 0.007984 | 0.0805 | 5.6165 | 5.3804 | 5.4497 | 5.3955 | 5.4454 | 5.5187 | 6.1479 | 6.0903 | 7.0585 | 5.9637 | 5.7914 | 6.8357 |
| 204369_at | 5290 | PIK3CA | phosphoinositide-3-kinase, catalytic, alpha polypeptide | -1.4361 | 0.007984 | 0.0805 | 9.0083 | 8.9134 | 9.0717 | 9.2487 | 8.9522 | 9.103 | 10.633 | 9.3686 | 11.3882 | 10.28 | 9.9167 | 9.8403 |
| 203360_s_at | 26292 | MYCBP | c-myc binding protein | -1.4513 | 0.007984 | 0.0805 | 9.5783 | 9.644 | 9.6968 | 9.5385 | 9.6634 | 9.804 | 10.042 | 10.1993 | 9.7072 | 10.3917 | 10.2247 | 10.3323 |
| 208894_at | 3122 | HLA-DRA | major histocompatibility complex, class II, DR alpha | -1.5512 | 0.007984 | 0.0805 | 5.8969 | 6.0115 | 5.7667 | 6.5104 | 5.8782 | 6.2018 | 7.5658 | 7.3226 | 7.2595 | 7.4693 | 6.2531 | 7.5319 |
| 201932_at | 10489 | LRRC41 | leucine rich repeat containing 41 | -1.6763 | 0.007984 | 0.0805 | 11.1746 | 11.1208 | 11.1114 | 11.1114 | 11.2169 | 11.1393 | 11.4922 | 11.6213 | 11.4145 | 11.3401 | 11.2718 | 11.6252 |
| 202602_s_at | 27336 | HTATSF1 | HIV-1 Tat specific factor 1 | -1.698 | 0.007984 | 0.0805 | 10.653 | 10.5073 | 10.9837 | 11.0563 | 10.633 | 10.9182 | 11.451 | 11.7544 | 11.9623 | 11.451 | 11.3401 | 12.0242 |
| 216347_s_at | 23368 | PPP1R13B | protein phosphatase 1, regulatory subunit 13B | -1.7562 | 0.007984 | 0.0805 | 6.2812 | 6.2114 | 6.741 | 6.1169 | 6.3908 | 6.4262 | 7.8329 | 7.1473 | 8.2845 | 7.3649 | 7.2382 | 7.2747 |
| 202016_at | 4232 | MEST | mesoderm specific transcript homolog (mouse) | -1.7642 | 0.007984 | 0.0805 | 13.6034 | 13.4469 | 13.6034 | 13.6034 | 13.6591 | 13.6034 | 13.962 | 14.4357 | 14.6375 | 14.0469 | 13.962 | 14.5815 |
| 209262_s_at | 2063 | NR2F6 | nuclear receptor subfamily 2, group F, member 6 | -1.9393 | 0.007984 | 0.0805 | 8.8371 | 8.7883 | 8.7304 | 8.6794 | 8.3111 | 8.5346 | 10.4304 | 10.2864 | 11.1208 | 9.2979 | 9.8572 | 10.212 |

| 621-102 |  |  |  |  |  |  | 5uM Chloroquine -24hrs |  |  |  |  |  | H2O treatment |  |  |  |  |  |
| --- | --- | --- | --- | --- | --- | --- | --- | --- | --- | --- | --- | --- | --- | --- | --- | --- | --- | --- |
| id | pr_gene_id | pr_gene_symbol | pr_gene_title | Signal to noise | p_value | FDR(BH) | HEN004_621-102_XH_X1.L2_B21:E02 | HEN004_621-102_XH_X1.L2_B21:E04 | HEN004_621-102_XH_X1.L2_B21:E06 | HEN004_621-102_XH_X1.L2_B21:E08 | HEN004_621-102_XH_X1.L2_B21:E10 | HEN004_621-102_XH_X1.L2_B21:E12 | HEN004_621-102_XH_X1.L2_B21:A14 | HEN004_621-102_XH_X1.L2_B21:A16 | HEN004_621-102_XH_X1.L2_B21:A18 | HEN004_621-102_XH_X1.L2_B21:A20 | HEN004_621-102_XH_X1.L2_B21:A22 | HEN004_621-102_XH_X1.L2_B21:A24 |
| 201626_at | 3638 | INSIG1 | insulin induced gene 1 | 1.9445 | 0.001996 | 0.3549 | 9.8234 | 9.5715 | 9.7177 | 9.5414 | 9.6359 | 9.5657 | 8.1402 | 8.47 | 8.6247 | 8.9058 | 8.8358 | 8.1881 |
| 208647_at | 2222 | FDFT1 | farnesyl-diphosphate farnesyltransferase 1 | 1.3129 | 0.001996 | 0.3549 | 10.7277 | 10.6716 | 10.9321 | 10.9719 | 10.6622 | 10.7811 | 9.9192 | 10.518 | 10.4012 | 10.3781 | 10.5113 | 10.3927 |
| 206562_s_at | 1452 | CSNK1A1 | casein kinase 1, alpha 1 | 1.1175 | 0.003992 | 0.3549 | 9.647 | 9.7917 | 9.6908 | 9.6092 | 9.6129 | 9.6213 | 9.0922 | 9.5814 | 9.1706 | 9.3768 | 9.4008 | 9.1143 |
| 204695_at | 993 | CDC25A | cell division cycle 25 homolog A (S. pombe) | 1.0703 | 0.001996 | 0.3549 | 11.0744 | 11.2801 | 11.2563 | 11.3003 | 11.4966 | 11.4472 | 10.8448 | 10.6579 | 11.0907 | 11.0139 | 11.0907 | 10.8535 |
| 204285_s_at | 5366 | PMAIP1 | phorbol-12-myristate-13-acetate-induced protein 1 | 0.9911 | 0.01198 | 0.3796 | 11.3003 | 11.0543 | 11.1454 | 11.2078 | 11.2801 | 11.2259 | 10.7811 | 10.8448 | 10.5015 | 10.6469 | 10.9184 | 11.1378 |
| 201393_s_at | 3482 | IGF2R | insulin-like growth factor 2 receptor | 0.9793 | 0.02196 | 0.411 | 8.4166 | 8.6688 | 8.7781 | 8.5715 | 8.6663 | 8.6338 | 8.4339 | 7.7509 | 7.9783 | 8.5966 | 7.8826 | 7.4558 |
| 201700_at | 896 | CCND3 | cyclin D3 | 0.9645 | 0.00998 | 0.3615 | 7.2396 | 7.2654 | 7.0954 | 7.2601 | 7.3415 | 7.2265 | 6.7033 | 7.0176 | 6.4991 | 6.8938 | 7.0176 | 6.7228 |
| 205504_at | 695 | BTK | Bruton agammaglobulinemia tyrosine kinase | 0.9629 | 0.007984 | 0.3615 | 4.0871 | 4.0252 | 4.1889 | 3.8487 | 4.052 | 3.641 | 3.1221 | 3.7081 | 3.7648 | 3.5495 | 3.4398 | 3.641 |
| 204514_at | 1802 | DPH2 | DPH2 homolog (S. cerevisiae) | 0.9574 | 0.005988 | 0.3615 | 8.5919 | 8.5852 | 8.5096 | 8.801 | 8.6071 | 8.7346 | 8.1089 | 8.5919 | 8.4002 | 8.581 | 8.2633 | 8.1439 |
| 212399_s_at | 9686 | VGLL4 | vestigial like 4 (Drosophila) | 0.9217 | 0.02395 | 0.411 | 7.7382 | 7.6781 | 7.7413 | 7.7509 | 7.6742 | 7.7733 | 7.5546 | 7.375 | 7.245 | 7.4715 | 7.7509 | 7.4435 |
| 212281_s_at | 27346 | TMEM97 | transmembrane protein 97 | 0.9212 | 0.00998 | 0.3615 | 10.1954 | 10.4318 | 10.6579 | 10.491 | 10.4499 | 10.3258 | 10.0817 | 10.3258 | 9.6247 | 9.9331 | 9.8234 | 9.753 |
| 200972_at | 10099 | TSPAN3 | tetraspanin 3 | 0.9199 | 0.01397 | 0.3796 | 11.0543 | 10.9929 | 11.237 | 11.1454 | 10.8754 | 10.9664 | 10.2308 | 11.0907 | 10.3997 | 10.518 | 10.5327 | 10.6225 |
| 208711_s_at | 595 | CCND1 | cyclin D1 | 0.8763 | 0.00998 | 0.3615 | 4.7409 | 4.8161 | 5.1321 | 4.7924 | 4.5716 | 4.6373 | 4.5899 | 4.65 | 4.2682 | 4.601 | 4.2178 | 4.2034 |
| 202540_s_at | 3156 | HMGCR | 3-hydroxy-3-methylglutaryl-CoA reductase | 0.8655 | 0.01198 | 0.3796 | 8.801 | 8.7356 | 8.9432 | 8.78 | 8.7913 | 8.6729 | 7.8575 | 8.7293 | 8.871 | 8.6346 | 8.4399 | 8.3972 |
| 201323_at | 10969 | EBNA1BP2 | EBNA1 binding protein 2 | 0.8538 | 0.01996 | 0.411 | 12.2004 | 12.2342 | 12.1763 | 12.1763 | 12.1763 | 12.2244 | 11.7843 | 12.3004 | 11.8331 | 11.9462 | 11.9984 | 11.7089 |
| 201555_at | 4172 | MCM3 | minichromosome maintenance complex component 3 | 0.8501 | 0.03792 | 0.4313 | 12.1301 | 12.1565 | 12.1565 | 11.8756 | 12.2771 | 12.1565 | 11.7318 | 12.0363 | 11.9863 | 12.0363 | 12.1067 | 11.8756 |
| 202651_at | 9926 | LPGAT1 | lysophosphatidylglycerol acyltransferase 1 | 0.8422 | 0.00998 | 0.3615 | 10.0188 | 10.0593 | 10.0094 | 9.9331 | 9.8973 | 9.9531 | 9.7997 | 9.8376 | 8.9454 | 9.6648 | 9.4264 | 9.4649 |
| 208424_s_at | 57019 | CIAPIN1 | cytokine induced apoptosis inhibitor 1 | 0.8323 | 0.005988 | 0.3615 | 10.0321 | 10.0005 | 10.1697 | 10.0321 | 9.9977 | 10.2496 | 9.455 | 9.5177 | 9.6908 | 10.1954 | 9.6961 | 9.6716 |
| 218847_at | 10644 | IGF2BP2 | insulin-like growth factor 2 mRNA binding protein 2 | 0.8312 | 0.01996 | 0.411 | 8.7683 | 8.6071 | 8.5472 | 8.7574 | 8.6517 | 8.2547 | 7.583 | 8.3723 | 8.1521 | 7.6211 | 8.231 | 8.0384 |
| 207620_s_at | 8573 | CASK | calcium/calmodulin-dependent serine protein kinase (MAGUK family) | 0.8257 | 0.01597 | 0.411 | 8.9058 | 8.6808 | 8.9681 | 8.8128 | 8.801 | 8.359 | 8.0446 | 8.0709 | 8.4166 | 8.4101 | 8.3088 | 7.848 |
| 202683_s_at | 8731 | RNMT | RNA (guanine-7-) methyltransferase | 0.8223 | 0.01796 | 0.411 | 8.3822 | 8.4025 | 8.6906 | 8.4049 | 8.2354 | 8.2084 | 8.1986 | 8.1918 | 7.8544 | 8.0104 | 8.0375 | 8.0781 |
| 202284_s_at | 1026 | CDKN1A | cyclin-dependent kinase inhibitor 1A (p21, Cip1) | 0.8219 | 0.02196 | 0.411 | 8.9671 | 8.9841 | 9.1601 | 8.8935 | 8.0321 | 7.9445 | 9.0088 | 7.7914 | 7.4385 | 7.9327 | 7.6723 | 7.7968 |
| 201727_s_at | 1994 | ELAVL1 | ELAV (embryonic lethal, abnormal vision, Drosophila)-like 1 (Hu antigen R) | 0.8119 | 0.02595 | 0.423 | 7.6257 | 7.5446 | 7.4044 | 7.5612 | 7.2 | 7.1088 | 6.9073 | 7.4435 | 6.3975 | 6.731 | 6.5355 | 6.6825 |
| 204995_at | 8851 | CDK5R1 | cyclin-dependent kinase 5, regulatory subunit 1 (p35) | 0.8079 | 0.01397 | 0.3796 | 5.9626 | 6.2473 | 6.1187 | 6.299 | 5.9915 | 5.8749 | 5.5531 | 5.8474 | 5.8059 | 5.9277 | 5.5863 | 5.9114 |
| 203046_s_at | 8914 | TIMELESS | timeless homolog (Drosophila) | 0.8043 | 0.03593 | 0.4285 | 10.0593 | 10.3469 | 10.2024 | 10.262 | 10.1954 | 10.2552 | 10.0188 | 10.2024 | 9.6875 | 10.2223 | 10.0094 | 10.1489 |
| 217168_s_at | 9709 | HERPUD1 | homocysteine-inducible, endoplasmic reticulum stress-inducible, ubiquitin-like domain member 1 | 0.8036 | 0.03992 | 0.4338 | 8.4501 | 8.3689 | 8.332 | 8.3516 | 8.1652 | 8.4775 | 8.4622 | 7.1241 | 7.1421 | 7.9661 | 6.9298 | 8.4002 |
| 212054_x_at | 23061 | TBC1D9B | TBC1 domain family, member 9B (with GRAM domain) | 0.7969 | 0.01397 | 0.3796 | 7.5528 | 7.8512 | 7.0824 | 7.6005 | 7.9623 | 7.0649 | 7.3549 | 7.1202 | 6.5746 | 6.7472 | 6.8157 | 6.8338 |
| 201000_at | 16 | AARS | alanyl-tRNA synthetase | 0.7961 | 0.04591 | 0.4402 | 8.446 | 8.4339 | 8.5324 | 8.4602 | 8.4815 | 8.524 | 8.2633 | 8.5113 | 8.3273 | 8.2886 | 8.3606 | 8.1874 |
| 202911_at | 2956 | MSH6 | mutS homolog 6 (E. coli) | 0.793 | 0.007984 | 0.3615 | 11.4472 | 11.2259 | 11.4763 | 11.3665 | 11.4334 | 11.1454 | 11.1454 | 11.1141 | 11.0139 | 11.2801 | 11.1454 | 10.8218 |
| 202735_at | 10682 | EBP | emopamil binding protein (sterol isomerase) | 0.782 | 0.00998 | 0.3615 | 8.8561 | 8.9351 | 9.455 | 8.9841 | 9.0859 | 8.9167 | 8.557 | 8.8377 | 8.2259 | 8.557 | 8.6639 | 8.4612 |
| 202794_at | 3628 | INPP1 | inositol polyphosphate-1-phosphatase | 0.7712 | 0.02196 | 0.411 | 7.848 | 7.6595 | 7.8326 | 7.7617 | 7.8144 | 7.499 | 7.3277 | 7.6365 | 7.1228 | 7.4973 | 7.6257 | 7.657 |
| 202675_at | 6390 | SDHB | succinate dehydrogenase complex, subunit B, iron sulfur (Ip) | 0.7508 | 0.007984 | 0.3615 | 12.0917 | 12.1301 | 12.2771 | 12.1565 | 12.3455 | 12.3004 | 11.7089 | 12.1067 | 11.5374 | 12.1067 | 12.1565 | 12.0594 |
| 210640_s_at | 2852 | GPER | G protein-coupled estrogen receptor 1 | 0.7486 | 0.02994 | 0.4285 | 9.1906 | 9.5537 | 9.5038 | 8.8894 | 8.6135 | 9.5496 | 8.5665 | 8.4815 | 7.9916 | 8.3368 | 8.4101 | 8.5715 |
| 208644_at | 142 | PARP1 | poly (ADP-ribose) polymerase 1 | 0.7463 | 0.01996 | 0.411 | 11.2563 | 11.1141 | 11.1661 | 11.1291 | 11.1378 | 11.4966 | 10.8754 | 11.237 | 11.1962 | 10.9533 | 10.8535 | 10.891 |
| 201074_at | 6599 | SMARCC1 | SWI/SNF related, matrix associated, actin dependent regulator of chromatin, subfamily c, member 1 | 0.7427 | 0.03992 | 0.4338 | 11.2078 | 11.353 | 11.5189 | 11.2563 | 11.3911 | 11.2801 | 10.8682 | 11.3665 | 10.8682 | 11.1141 | 10.7927 | 10.9664 |
| 219016_at | 60493 | FASTKD5 | FAST kinase domains 5 | 0.7426 | 0.01198 | 0.3796 | 9.1706 | 9.0733 | 9.3099 | 9.0411 | 9.0577 | 9.3433 | 8.7844 | 9.2127 | 8.094 | 8.8671 | 8.6071 | 8.9175 |
| 201170_s_at | 8553 | BHLHE40 | basic helix-loop-helix family, member e40 | 0.7407 | 0.01397 | 0.3796 | 4.2682 | 4.4407 | 4.356 | 4.601 | 4.1889 | 4.151 | 4.0871 | 4.0871 | 4.151 | 3.7648 | 3.9141 | 3.955 |
| 202100_at | 5899 | RALB | v-ral simian leukemia viral oncogene homolog B (ras related; GTP binding protein) | 0.7395 | 0.02595 | 0.423 | 7.1768 | 7.461 | 7.7811 | 7.7011 | 7.6781 | 7.2967 | 6.6373 | 6.8597 | 6.7251 | 7.0859 | 7.2265 | 7.5385 |
| 202733_at | 8974 | P4HA2 | prolyl 4-hydroxylase, alpha polypeptide II | 0.737 | 0.03593 | 0.4285 | 5.609 | 5.831 | 5.9524 | 5.7925 | 5.4641 | 5.6937 | 5.452 | 5.4947 | 5.803 | 5.3609 | 5.5037 | 5.1062 |
| 209260_at | 2810 | SFN | stratifin | 0.7317 | 0.01996 | 0.411 | 3.955 | 4.3164 | 4.052 | 3.7081 | 3.8487 | 4.3308 | 3.7365 | 3.8487 | 3.1221 | 3.4398 | 3.5495 | 3.7365 |
| 217784_at | 10652 | YKT6 | YKT6 v-SNARE homolog (S. cerevisiae) | 0.7304 | 0.02994 | 0.4285 | 9.4345 | 9.8234 | 9.6648 | 9.7015 | 9.3333 | 9.6785 | 9.1143 | 9.4517 | 8.779 | 9.0765 | 9.032 | 9.2815 |
| 203123_s_at | 4891 | SLC11A2 | solute carrier family 11 (proton-coupled divalent metal ion transporters), member 2 | 0.7252 | 0.03194 | 0.4285 | 6.9298 | 7.2524 | 7.6044 | 7.0229 | 6.9803 | 6.9073 | 6.93 | 6.7289 | 6.4268 | 6.0329 | 6.731 | 6.2411 |
| 212115_at | 90861 | HN1L | hematological and neurological expressed 1-like | 0.7248 | 0.04591 | 0.4402 | 9.41 | 9.5376 | 9.7373 | 9.5814 | 9.4483 | 9.6908 | 9.1036 | 9.5715 | 9.1386 | 9.4219 | 9.238 | 9.0765 |
| 200059_s_at | 387 | RHOA | ras homolog gene family, member A | 0.7245 | 0.05988 | 0.4761 | 8.4049 | 8.7153 | 8.6858 | 8.5831 | 8.5919 | 8.6639 | 8.6071 | 8.6247 | 8.231 | 8.359 | 8.249 | 8.7012 |
| 202396_at | 10915 | TCERG1 | transcription elongation regulator 1 | 0.7233 | 0.01996 | 0.411 | 10.891 | 10.9321 | 11.0744 | 11.1378 | 11.0744 | 10.6225 | 10.7025 | 10.771 | 10.6469 | 10.5646 | 10.6326 | 10.6579 |
| 207143_at | 1021 | CDK6 | cyclin-dependent kinase 6 | 0.7146 | 0.02994 | 0.4285 | 5.4862 | 5.432 | 5.5863 | 5.6175 | 5.3609 | 5.2393 | 5.0322 | 5.3047 | 5.3817 | 4.9166 | 4.8469 | 4.7131 |
| 212795_at | 23325 | KIAA1033 | KIAA1033 | 0.7135 | 0.03194 | 0.4285 | 7.4188 | 7.3307 | 7.6938 | 7.5612 | 7.3914 | 7.4973 | 7.4511 | 7.2667 | 7.1254 | 7.2601 | 7.0111 | 6.8978 |
| 200807_s_at | 3329 | HSPD1 | heat shock 60kDa protein 1 (chaperonin) | 0.7107 | 0.02794 | 0.427 | 11.5626 | 11.617 | 11.8924 | 12.2004 | 11.7662 | 11.8563 | 11.4204 | 11.7318 | 11.3234 | 11.2563 | 11.4334 | 11.3665 |
| 201899_s_at | 7319 | UBE2A | ubiquitin-conjugating enzyme E2A | 0.6999 | 0.02395 | 0.411 | 10.7112 | 10.7789 | 11.0139 | 10.7415 | 10.6326 | 10.7631 | 10.3997 | 10.6909 | 10.2832 | 10.5015 | 10.518 | 10.4499 |
| 204977_at | 1662 | DDX10 | DEAD (Asp-Glu-Ala-Asp) box polypeptide 10 | 0.6911 | 0.03194 | 0.4285 | 9.1196 | 8.9027 | 9.2633 | 8.9671 | 8.633 | 8.7457 | 8.5194 | 8.8748 | 8.3516 | 8.5919 | 8.6391 | 8.4166 |
| 204092_s_at | 6790 | AURKA | aurora kinase A | 0.67 | 0.04591 | 0.4402 | 6.7107 | 6.1155 | 6.836 | 6.765 | 6.5425 | 6.2473 | 6.2758 | 5.4536 | 6.1336 | 6.5867 | 5.6125 | 6.289 |
| 202788_at | 7867 | MAPKAPK3 | mitogen-activated protein kinase-activated protein kinase 3 | 0.6627 | 0.02794 | 0.427 | 7.2118 | 6.4346 | 7.1856 | 6.4014 | 7.2304 | 6.2473 | 6.0275 | 6.1406 | 6.1957 | 6.282 | 5.9693 | 7.2304 |
| 203291_at | 4850 | CNOT4 | CCR4-NOT transcription complex, subunit 4 | 0.6564 | 0.0479 | 0.442 | 7.4732 | 7.6257 | 7.6126 | 10.7927 | 11.0139 | 7.0649 | 6.7423 | 7.1587 | 6.7472 | 7.4336 | 7.4435 | 6.9618 |
| 201719_s_at | 2037 | EPB41L2 | erythrocyte membrane protein band 4.1-like 2 | 0.6464 | 0.03593 | 0.4285 | 9.8376 | 10.0094 | 10.4072 | 10.4012 | 10.0688 | 9.955 | 9.8546 | 9.9689 | 9.8129 | 9.8973 | 9.955 | 9.6908 |
| 203132_at | 5925 | RB1 | retinoblastoma 1 | 0.6375 | 0.05389 | 0.4718 | 8.5665 | 8.748 | 8.6368 | 8.6071 | 8.332 | 8.3739 | 8.2886 | 8.5324 | 8.261 | 8.4664 | 8.1652 | 8.1305 |
| 209409_at | 2887 | GRB10 | growth factor receptor-bound protein 10 | 0.6263 | 0.04192 | 0.4361 | 7.9882 | 8.3273 | 8.0874 | 8.2735 | 7.9386 | 7.9916 | 7.7231 | 8.1808 | 7.7968 | 7.6257 | 7.8575 | 7.7856 |
| 206015_s_at | 22887 | FOXJ3 | forkhead box J3 | 0.6261 | 0.005988 | 0.3615 | 10.1697 | 9.955 | 10.1385 | 10.3359 | 10.4133 | 9.8849 | 9.6247 | 10.0688 | 9.7101 | 9.7917 | 9.7561 | 9.9229 |
| 221478_at | 665 | BNIP3L | BCL2/adenovirus E1B 19kDa interacting protein 3-like | 0.6144 | 0.06986 | 0.488 | 7.8826 | 7.848 | 8.0709 | 7.9752 | 7.9783 | 7.6416 | 7.4911 | 7.6595 | 7.2654 | 7.3277 | 7.6723 | 7.1835 |
| 208624_s_at | 1981 | EIF4G1 | eukaryotic translation initiation factor 4 gamma, 1 | 0.6119 | 0.07585 | 0.5012 | 9.1962 | 8.9295 | 9.1143 | 9.2815 | 9.0922 | 8.7878 | 8.5324 | 8.3178 | 9.1601 | 8.9235 | 8.9147 | 8.1565 |
| 205562_at | 10557 | RPP38 | ribonuclease P/MRP 38kDa subunit | 0.605 | 0.04591 | 0.4402 | 8.3273 | 8.4622 | 7.7878 | 8.321 | 8.3158 | 7.4511 | 7.4662 | 7.4144 | 8.0781 | 7.2064 | 7.3549 | 7.4336 |
| 203135_at | 6908 | TBP | TATA box binding protein | 0.6037 | 0.07984 | 0.5104 | 9.2876 | 9.3308 | 9.5537 | 9.4264 | 9.3685 | 9.6056 | 8.5996 | 9.5094 | 8.6391 | 9.3333 | 9.2876 | 8.7978 |
| 203013_at | 11319 | ECD | ecdysoneless homolog (Drosophila) | 0.6033 | 0.07585 | 0.5012 | 8.5356 | 8.6338 | 8.5388 | 8.0938 | 8.5127 | 8.3107 | 7.8245 | 8.2519 | 7.9661 | 8.3754 | 8.4172 | 7.9945 |
| 202174_s_at | 5108 | PCM1 | pericentriolar material 1 | 0.6018 | 0.05988 | 0.4761 | 7.2601 | 7.3806 | 7.6781 | 7.3377 | 7.6485 | 6.8316 | 6.8597 | 7.3377 | 6.7251 | 7.2641 | 7.0859 | 6.8551 |
| 201664_at | 10051 | SMC4 | structural maintenance of chromosomes 4 | 0.5993 | 0.06587 | 0.4807 | 10.7789 | 10.7415 | 10.8682 | 10.7112 | 10.7811 | 10.5248 | 10.2086 | 10.584 | 10.3576 | 10.6154 | 10.4689 | 10.4012 |
| 218595_s_at | 55127 | HEATR1 | HEAT repeat containing 1 | 0.5979 | 0.1058 | 0.5474 | 10.0688 | 9.9192 | 10.0554 | 9.7249 | 10.174 | 10.3074 | 9.6648 | 10.3074 | 9.8325 | 10.0005 | 10.2086 | 9.2516 |
| 213702_x_at | 427 | ASAH1 | N-acylsphingosine amidohydrolase (acid ceramidase) 1 | 0.597 | 0.03792 | 0.4313 | 8.8935 | 9.2815 | 9.2936 | 9.238 | 9.1036 | 9.0656 | 7.9445 | 8.9681 | 8.8182 | 8.9398 | 9.0765 | 8.2886 |
| 201528_at | 6117 | RPA1 | replication protein A1, 70kDa | 0.5966 | 0.09381 | 0.5294 | 11.353 | 11.4048 | 11.4334 | 11.4334 | 11.3348 | 11.4204 | 11.353 | 11.4966 | 11.1661 | 11.3665 | 11.1661 | 11.2801 |
| 208025_s_at | 8091 | HMGA2 | high mobility group AT-hook 2 | 0.5926 | 0.1118 | 0.5521 | 9.2274 | 9.1996 | 9.3022 | 9.2754 | 9.2274 | 8.9514 | 8.5466 | 9.2876 | 8.7683 | 8.9841 | 8.7079 | 8.6099 |
| 208763_s_at | 1831 | TSC22D3 | TSC22 domain family, member 3 | 0.5906 | 0.06986 | 0.488 | 10.3258 | 10.0612 | 10.1602 | 10.1453 | 10.1489 | 9.9211 | 9.6038 | 10.0188 | 9.3568 | 9.776 | 9.4345 | 9.6827 |
| 201098_at | 9276 | COPB2 | coatomer protein complex, subunit beta 2 (beta prime) | 0.5866 | 0.07984 | 0.5104 | 10.5962 | 10.4689 | 10.5646 | 10.6225 | 10.5765 | 10.6579 | 10.5765 | 10.8535 | 10.0946 | 10.2024 | 10.4318 | 10.4396 |
| 212740_at | 30849 | PIK3R4 | phosphoinositide-3-kinase, regulatory subunit 4 | 0.5865 | 0.06387 | 0.4805 | 7.3188 | 7.5564 | 7.6595 | 7.3841 | 7.4511 | 7.266 | 7.2343 | 7.5244 | 6.7251 | 7.3089 | 6.8703 | 6.1877 |
| 203304_at | 25805 | BAMBI | BMP and activin membrane-bound inhibitor homolog (Xenopus laevis) | 0.5865 | 0.06986 | 0.488 | 8.557 | 8.272 | 8.5869 | 8.5506 | 8.7265 | 8.4664 | 8.1103 | 8.3368 | 8.1439 | 8.5388 | 8.1263 | 8.3088 |
| 218193_s_at | 51026 | GOLT1B | golgi transport 1B | 0.5862 | 0.2116 | 0.6675 | 9.9373 | 9.6171 | 9.9229 | 9.8546 | 10.0612 | 10.1697 | 9.955 | 9.8445 | 4.8161 | 9.6609 | 5.4536 | 9.9531 |
| 217761_at | 55256 | ADI1 | acireductone dioxygenase 1 | 0.5855 | 0.2455 | 0.696 | 8.5869 | 8.3623 | 8.581 | 8.5167 | 8.4835 | 8.2886 | 6.9296 | 8.557 | 8.4835 | 8.78 | 5.7166 | 6.3138 |
| 204087_s_at | 8884 | SLC5A6 | solute carrier family 5 (sodium-dependent vitamin transporter), member 6 | 0.5806 | 0.09182 | 0.5282 | 9.2754 | 9.4599 | 7.9752 | 9.1512 | 9.2516 | 9.3308 | 8.9175 | 7.5446 | 9.0023 | 9.0411 | 8.6171 | 8.6247 |
| 202954_at | 11065 | UBE2C | ubiquitin-conjugating enzyme E2C | 0.577 | 0.1038 | 0.5428 | 11.4048 | 11.6611 | 11.6409 | 11.617 | 11.4472 | 11.7662 | 11.0744 | 10.3576 | 10.9533 | 11.2259 | 11.1291 | 11.1454 |
| 202963_at | 5993 | RFX5 | regulatory factor X, 5 (influences HLA class II expression) | 0.577 | 0.00998 | 0.3615 | 8.2457 | 8.4825 | 8.3857 | 8.1881 | 8.3098 | 8.8195 | 7.9285 | 7.9512 | 7.8928 | 8.4339 | 7.8928 | 7.8165 |
| 218086_at | 56654 | NPDC1 | neural proliferation, differentiation and control, 1 | 0.5763 | 0.04391 | 0.4402 | 8.581 | 8.4825 | 8.6808 | 8.1808 | 8.7116 | 8.6391 | 8.272 | 8.261 | 8.3088 | 5.3457 | 8.2886 | 8.6517 |
| 218346_s_at | 27244 | SESN1 | sestrin 1 | 0.5728 | 0.05589 | 0.4718 | 7.4296 | 6.7967 | 6.7033 | 6.6825 | 7.3914 | 7.5692 | 7.2265 | 6.7967 | 6.3433 | 6.2911 | 6.5942 | 6.4408 |
| 202784_s_at | 23530 | NNT | nicotinamide nucleotide transhydrogenase | 0.5636 | 0.0519 | 0.4614 | 8.1263 | 7.8401 | 8.2423 | 8.4892 | 8.6663 | 7.9974 | 7.9974 | 7.8877 | 7.8544 | 7.6126 | 7.6211 | 7.5131 |
| 202813_at | 6894 | TARBP1 | TAR (HIV-1) RNA binding protein 1 | 0.5624 | 0.09581 | 0.5294 | 8.78 | 9.3908 | 9.1906 | 9.1169 | 9.3603 | 9.3209 | 8.7293 | 9.0983 | 8.9027 | 8.5282 | 8.9058 | 9.0859 |
| 200882_s_at | 5710 | PSMD4 | proteasome (prosome, macropain) 26S subunit, non-ATPase, 4 | 0.5593 | 0.07585 | 0.5012 | 11.9863 | 11.9863 | 11.9462 | 11.809 | 12.019 | 12.2771 | 11.5374 | 11.809 | 11.6864 | 11.7843 | 11.9863 | 11.809 |
| 201080_at | 8396 | PIP4K2B | phosphatidylinositol-5-phosphate 4-kinase, type II, beta | 0.5548 | 0.06587 | 0.4807 | 6.2268 | 5.5732 | 6.2131 | 6.2473 | 6.3706 | 6.095 | 5.5138 | 6.0004 | 5.5037 | 5.8552 | 5.5606 | 5.6175 |
| 202491_s_at | 8518 | IKBKAP | inhibitor of kappa light polypeptide gene enhancer in B-cells, kinase complex-associated protein | 0.5542 | 0.01796 | 0.411 | 10.4689 | 10.8682 | 10.5327 | 10.584 | 10.8218 | 10.8754 | 10.6469 | 10.3997 | 10.3258 | 10.5113 | 10.4499 | 10.7277 |
| 208634_s_at | 23499 | MACF1 | microtubule-actin crosslinking factor 1 | 0.553 | 0.03393 | 0.4285 | 9.0351 | 9.1315 | 9.3515 | 9.4483 | 9.032 | 8.8561 | 9.0351 | 9.1706 | 8.7527 | 8.6729 | 8.9897 | 8.6858 |
| 201498_at | 7874 | USP7 | ubiquitin specific peptidase 7 (herpes virus-associated) | 0.5529 | 0.0479 | 0.442 | 6.9645 | 6.6276 | 7.0649 | 7.0843 | 6.7968 | 10.0094 | 6.4441 | 6.8907 | 6.2473 | 6.5676 | 6.2624 | 6.2034 |
| 202148_s_at | 5831 | PYCR1 | pyrroline-5-carboxylate reductase 1 | 0.5464 | 0.08583 | 0.5118 | 8.4602 | 8.0446 | 8.1348 | 7.7351 | 8.4049 | 8.4166 | 7.5652 | 8.2519 | 7.4255 | 7.3841 | 7.3188 | 7.6938 |
| 200981_x_at | 2778 | GNAS | GNAS complex locus | 0.5459 | 0.03393 | 0.4285 | 12.2293 | 11.9462 | 12.1067 | 12.3234 | 12.0917 | 12.1763 | 11.9462 | 12.1565 | 11.8563 | 11.8756 | 11.8331 | 11.9863 |
| 210788_s_at | 51635 | DHRS7 | dehydrogenase/reductase (SDR family) member 7 | 0.5424 | 0.1756 | 0.6322 | 10.1602 | 10.4237 | 10.3576 | 10.1184 | 10.2832 | 10.3359 | 10.1602 | 9.9331 | 9.4008 | 8.4169 | 7.4435 | 10.2055 |
| 203209_at | 5985 | RFC5 | replication factor C (activator 1) 5, 36.5kDa | 0.5422 | 0.09581 | 0.5294 | 9.5117 | 9.4254 | 9.3568 | 9.455 | 9.2079 | 9.2437 | 9.1265 | 9.0922 | 9.2995 | 9.2995 | 9.2274 | 9.0574 |
| 201416_at | 6659 | SOX4 | SRY (sex determining region Y)-box 4 | 0.5407 | 0.08583 | 0.5118 | 9.9689 | 9.8129 | 9.9759 | 10.045 | 9.9468 | 9.7373 | 9.5376 | 9.7285 | 9.3209 | 9.6785 | 9.6806 | 9.4888 |
| 218780_at | 29911 | HOOK2 | hook homolog 2 (Drosophila) | 0.5394 | 0.06986 | 0.488 | 5.0107 | 4.7409 | 4.7131 | 5.0263 | 4.7754 | 4.9937 | 4.8161 | 4.7487 | 4.9783 | 4.7052 | 4.9103 | 4.685 |
| 202705_at | 9133 | CCNB2 | cyclin B2 | 0.5392 | 0.3553 | 0.7756 | 11.0292 | 11.0744 | 11.1291 | 10.7631 | 10.9184 | 11.2078 | 11.0543 | 6.9123 | 10.7789 | 11.0543 | 7.9661 | 11.1962 |
| 40562_at | 2767 | GNA11 | guanine nucleotide binding protein (G protein), alpha 11 (Gq class) | 0.5377 | 0.06188 | 0.4765 | 9.4008 | 9.3603 | 8.7913 | 8.502 | 9.4599 | 8.6944 | 8.1565 | 8.446 | 8.249 | 8.4835 | 8.3723 | 9.1706 |
| 208626_s_at | 10493 | VAT1 | vesicle amine transport protein 1 homolog (T. californica) | 0.5334 | 0.09581 | 0.5294 | 8.231 | 8.3425 | 8.6284 | 8.3754 | 8.261 | 8.3822 | 7.872 | 8.446 | 7.7165 | 8.0384 | 7.8745 | 8.1986 |
| 206070_s_at | 2042 | EPHA3 | EPH receptor A3 | 0.5314 | 0.08184 | 0.5118 | 6.6599 | 6.5474 | 7.2383 | 6.93 | 5.7319 | 7.0649 | 5.7928 | 5.706 | 5.7319 | 5.7925 | 5.5378 | 5.7166 |
| 202220_at | 22889 | KIAA0907 | KIAA0907 | 0.5311 | 0.1018 | 0.5428 | 9.7213 | 9.8849 | 9.7177 | 9.8325 | 9.8376 | 9.5067 | 9.2437 | 9.6785 | 9.4219 | 9.6047 | 9.4768 | 9.455 |
| 201985_at | 9897 | KIAA0196 | KIAA0196 | 0.5216 | 0.07984 | 0.5104 | 9.7917 | 9.7285 | 9.5894 | 9.7142 | 9.6213 | 9.3187 | 9.2754 | 9.6359 | 9.6038 | 9.6047 | 9.3433 | 9.4483 |
| 204761_at | 9712 | USP6NL | USP6 N-terminal like | 0.5205 | 0.05788 | 0.4718 | 7.6365 | 7.4715 | 10.2986 | 7.872 | 7.8967 | 7.7351 | 7.5546 | 7.8165 | 7.4732 | 7.6485 | 7.7072 | 7.4144 |
| 212166_at | 23039 | XPO7 | exportin 7 | 0.5196 | 0.02395 | 0.411 | 10.4237 | 10.5646 | 10.6154 | 10.4318 | 10.4772 | 10.2024 | 10.2223 | 10.3927 | 9.9331 | 10.4012 | 9.776 | 10.0946 |
| 201361_at | 79073 | TMEM109 | transmembrane protein 109 | 0.5182 | 0.05589 | 0.4718 | 8.7574 | 8.6247 | 8.6476 | 8.8358 | 8.7191 | 8.7153 | 8.5388 | 8.6688 | 7.5761 | 8.6071 | 8.0517 | 8.5324 |
| 206501_x_at | 2115 | ETV1 | ets variant 1 | 0.5174 | 0.08184 | 0.5118 | 6.1957 | 6.1155 | 6.1871 | 6.2758 | 6.0718 | 5.8537 | 5.9329 | 5.7514 | 5.8804 | 5.9488 | 5.641 | 5.9659 |
| 200915_x_at | 3895 | KTN1 | kinectin 1 (kinesin receptor) | 0.5166 | 0.07385 | 0.5012 | 10.2552 | 10.0554 | 9.9531 | 10.1453 | 9.9759 | 9.537 | 9.4888 | 9.9531 | 9.995 | 9.5657 | 9.7498 | 9.7917 |
| 218474_s_at | 54442 | KCTD5 | potassium channel tetramerisation domain containing 5 | 0.5141 | 0.1317 | 0.5804 | 6.3809 | 6.3809 | 6.4346 | 6.5127 | 6.4346 | 6.3706 | 6.3105 | 6.3575 | 6.1406 | 6.3105 | 6.1214 | 6.1092 |
| 201334_s_at | 23365 | ARHGEF12 | Rho guanine nucleotide exchange factor (GEF) 12 | 0.5071 | 0.08583 | 0.5118 | 6.3308 | 6.0253 | 6.2473 | 6.3388 | 6.0544 | 5.831 | 6.1155 | 6.5245 | 5.8715 | 5.9009 | 5.641 | 6.0427 |
| 200078_s_at | 533 | ATP6V0B | ATPase, H+ transporting, lysosomal 21kDa, V0 subunit b | 0.5058 | 0.1158 | 0.5605 | 7.9822 | 7.6044 | 7.9327 | 7.7986 | 7.8692 | 7.7986 | 7.0859 | 7.6442 | 7.1202 | 7.7733 | 7.4255 | 7.4222 |
| 208872_s_at | 7905 | REEP5 | receptor accessory protein 5 | 0.504 | 0.0978 | 0.5314 | 8.1986 | 8.9574 | 8.8182 | 8.3639 | 8.2547 | 8.4835 | 7.9019 | 8.3248 | 7.5357 | 7.7198 | 8.0672 | 7.9882 |
| 201519_at | 9868 | TOMM70A | translocase of outer mitochondrial membrane 70 homolog A (S. cerevisiae) | 0.5019 | 0.1118 | 0.5521 | 9.5238 | 9.4483 | 9.647 | 9.647 | 9.5715 | 9.2876 | 9.1555 | 9.5496 | 9.3768 | 9.4008 | 9.3908 | 9.1601 |
| 217496_s_at | 3416 | IDE | insulin-degrading enzyme | 0.4992 | 0.0519 | 0.4614 | 5.3933 | 5.2092 | 5.473 | 5.4195 | 8.2423 | 5.1384 | 5.3008 | 5.0884 | 5.1258 | 5.0107 | 5.0739 | 5.2488 |
| 207871_s_at | 7982 | ST7 | suppression of tumorigenicity 7 | 0.4954 | 0.1038 | 0.5428 | 6.0818 | 6.2855 | 6.4669 | 6.1917 | 6.0359 | 6.0078 | 5.6458 | 6.4515 | 5.7514 | 5.8338 | 5.8715 | 5.8411 |
| 201589_at | 8243 | SMC1A | structural maintenance of chromosomes 1A | 0.4946 | 0.001996 | 0.3549 | 11.5374 | 11.6409 | 11.6864 | 11.7089 | 11.5842 | 11.3665 | 11.2078 | 5.001 | 11.4204 | 11.5189 | 11.3665 | 11.237 |
| 200670_at | 7494 | XBP1 | X-box binding protein 1 | 0.4913 | 0.08583 | 0.5118 | 8.446 | 8.7157 | 8.633 | 8.7265 | 8.1881 | 8.1475 | 7.9595 | 8.3516 | 8.0384 | 8.0555 | 8.0104 | 8.249 |
| 204089_x_at | 4216 | MAP3K4 | mitogen-activated protein kinase kinase kinase 4 | 0.4895 | 0.1317 | 0.5804 | 6.794 | 6.93 | 7.3021 | 7.1254 | 7.0745 | 6.8126 | 6.9073 | 7.0932 | 6.5676 | 6.836 | 6.5867 | 6.3388 |
| 203775_at | 10165 | SLC25A13 | solute carrier family 25, member 13 (citrin) | 0.4852 | 0.03792 | 0.4313 | 10.8365 | 10.4133 | 10.4772 | 10.4499 | 10.3997 | 10.3106 | 9.9229 | 10.2959 | 10.2959 | 9.9192 | 10.303 | 9.4259 |
| 210105_s_at | 2534 | FYN | FYN oncogene related to SRC, FGR, YES | 0.4843 | 0.1776 | 0.6322 | 7.6974 | 7.7413 | 7.9078 | 7.7617 | 7.6211 | 8.0043 | 7.4732 | 7.9822 | 7.2733 | 7.6416 | 7.5612 | 7.2 |
| 208309_s_at | 10892 | MALT1 | mucosa associated lymphoid tissue lymphoma translocation gene 1 | 0.4839 | 0.1397 | 0.5865 | 6.0275 | 6.1877 | 6.1727 | 6.1092 | 6.3575 | 5.938 | 5.5531 | 5.8783 | 6.3187 | 5.641 | 6.0359 | 5.341 |
| 217901_at | 1829 | DSG2 | desmoglein 2 | 0.4838 | 0.1277 | 0.5784 | 9.7142 | 9.9658 | 10.2086 | 10.0188 | 9.8234 | 9.8546 | 9.647 | 7.5973 | 9.4259 | 9.706 | 9.706 | 9.3768 |
| 201379_s_at | 7165 | TPD52L2 | tumor protein D52-like 2 | 0.4837 | 0.1238 | 0.5763 | 6.413 | 6.1984 | 6.1533 | 6.3163 | 6.2011 | 6.1957 | 6.157 | 6.2473 | 6.2473 | 6.2011 | 6.0847 | 6.0193 |
| 204252_at | 1017 | CDK2 | cyclin-dependent kinase 2 | 0.4801 | 0.1078 | 0.549 | 9.5009 | 9.5414 | 9.9133 | 9.9192 | 9.7498 | 9.4345 | 9.2192 | 9.706 | 9.7498 | 9.5094 | 9.6806 | 9.405 |
| 203408_s_at | 6304 | SATB1 | SATB homeobox 1 | 0.4787 | 0.08982 | 0.5198 | 5.0786 | 4.2682 | 4.4407 | 5.294 | 5.2092 | 5.2092 | 3.7365 | 4.1889 | 4.1172 | 4.972 | 4.4038 | 4.2444 |
| 213233_s_at | 55958 | KLHL9 | kelch-like 9 (Drosophila) | 0.4783 | 0.1816 | 0.6322 | 7.5897 | 7.2667 | 7.4662 | 7.044 | 7.2304 | 6.7228 | 7.1254 | 7.0623 | 7.3055 | 7.2396 | 7.2396 | 6.4589 |
| 218093_s_at | 55608 | ANKRD10 | ankyrin repeat domain 10 | 0.4781 | 0.1876 | 0.6394 | 8.0375 | 7.9285 | 8.0321 | 7.6442 | 7.9974 | 6.3348 | 7.4435 | 7.836 | 7.4435 | 7.4715 | 6.9462 | 6.3575 |
| 209567_at | 23212 | RRS1 | RRS1 ribosome biogenesis regulator homolog (S. cerevisiae) | 0.477 | 0.1038 | 0.5428 | 7.5385 | 7.044 | 9.0656 | 7.3188 | 7.375 | 7.3467 | 7.0229 | 7.4873 | 6.9591 | 7.1331 | 7.0745 | 7.0202 |
| 201853_s_at | 994 | CDC25B | cell division cycle 25 homolog B (S. pombe) | 0.4752 | 0.3094 | 0.7309 | 9.4483 | 9.3187 | 9.4237 | 9.5376 | 9.5376 | 9.1623 | 9.41 | 9.3685 | 9.1512 | 5.6619 | 9.3768 | 9.1623 |
| 202761_s_at | 23224 | SYNE2 | spectrin repeat containing, nuclear envelope 2 | 0.4752 | 0.1038 | 0.5428 | 6.9573 | 6.8126 | 8.1565 | 7.3307 | 6.9712 | 7.0284 | 6.6193 | 6.6781 | 6.7808 | 6.8082 | 7.1445 | 6.5894 |
| 203640_at | 10150 | MBNL2 | muscleblind-like 2 (Drosophila) | 0.4745 | 0.2834 | 0.7295 | 9.955 | 9.7213 | 10.1489 | 9.8234 | 9.7997 | 9.3099 | 9.7839 | 8.2735 | 9.3308 | 9.6875 | 9.8973 | 9.7719 |
| 202726_at | 3978 | LIG1 | ligase I, DNA, ATP-dependent | 0.4732 | 0.2016 | 0.6529 | 8.0481 | 8.0321 | 7.8826 | 8.231 | 8.2084 | 8.0219 | 7.848 | 8.2259 | 7.0474 | 7.3974 | 7.9019 | 7.3848 |
| 203315_at | 8440 | NCK2 | NCK adaptor protein 2 | 0.4724 | 0.1277 | 0.5784 | 6.873 | 7.4435 | 7.441 | 7.3089 | 7.3009 | 6.6825 | 6.9073 | 7.3519 | 7.028 | 7.2784 | 6.8126 | 6.8597 |
| 201011_at | 6184 | RPN1 | ribophorin I | 0.4699 | 0.1397 | 0.5865 | 13.0626 | 12.9163 | 13.0626 | 13.2279 | 13.2954 | 13.2279 | 12.662 | 12.8313 | 12.7071 | 13.0626 | 13.0053 | 12.9163 |
| 201186_at | 4043 | LRPAP1 | low density lipoprotein receptor-related protein associated protein 1 | 0.4669 | 0.2016 | 0.6529 | 9.0023 | 9.1831 | 9.2876 | 9.3022 | 9.3099 | 9.2323 | 8.6808 | 9.1386 | 8.779 | 9.2192 | 8.801 | 8.8561 |
| 205046_at | 1062 | CENPE | centromere protein E, 312kDa | 0.4652 | 0.1597 | 0.6077 | 7.5357 | 9.0922 | 8.9748 | 8.9432 | 8.446 | 8.2172 | 8.3723 | 8.6858 | 8.6071 | 8.5506 | 8.205 | 6.9865 |
| 218695_at | 54512 | EXOSC4 | exosome component 4 | 0.4648 | 0.1617 | 0.6129 | 5.6507 | 6.1336 | 6.282 | 6.1336 | 5.1612 | 6.282 | 5.7669 | 5.8983 | 4.8576 | 4.972 | 5.1384 | 4.9901 |
| 218852_at | 55012 | PPP2R3C | protein phosphatase 2, regulatory subunit B'', gamma | 0.4644 | 0.3074 | 0.7309 | 9.6609 | 9.8376 | 10.045 | 9.6785 | 9.6056 | 9.5414 | 9.2274 | 9.7498 | 5.9559 | 9.5117 | 9.8521 | 9.7285 |
| 212846_at | 23076 | RRP1B | ribosomal RNA processing 1 homolog B (S. cerevisiae) | 0.4642 | 0.1018 | 0.5428 | 9.9331 | 9.8445 | 9.8849 | 10.1782 | 9.9229 | 9.7285 | 9.4483 | 9.5067 | 9.5363 | 9.5715 | 9.6247 | 9.5894 |
| 202797_at | 22908 | SACM1L | SAC1 suppressor of actin mutations 1-like (yeast) | 0.4641 | 0.1657 | 0.6184 | 9.8973 | 9.9331 | 9.9468 | 9.955 | 9.8849 | 9.8445 | 10.0593 | 9.9192 | 9.5814 | 9.7213 | 9.9901 | 9.776 |
| 201875_s_at | 9019 | MPZL1 | myelin protein zero-like 1 | 0.4616 | 0.1238 | 0.5763 | 9.3603 | 9.2204 | 9.6827 | 9.5094 | 9.1962 | 9.4008 | 9.0733 | 9.0292 | 9.0922 | 9.232 | 9.2198 | 8.8209 |
| 213844_at | 3202 | HOXA5 | homeobox A5 | 0.4609 | 0.003992 | 0.3549 | 12.465 | 12.7591 | 12.8313 | 12.5889 | 12.5889 | 12.4511 | 12.1301 | 12.3234 | 12.0917 | 12.3455 | 12.2771 | 12.1565 |
| 205963_s_at | 9093 | DNAJA3 | DnaJ (Hsp40) homolog, subfamily A, member 3 | 0.4604 | 0.2176 | 0.6689 | 12.4511 | 12.5889 | 11.5626 | 11.6409 | 11.3003 | 11.3911 | 11.237 | 11.3234 | 13.6371 | 6.0818 | 12.3753 | 12.7071 |
| 200617_at | 9761 | MLEC | malectin | 0.4599 | 0.2615 | 0.7084 | 10.6579 | 10.7927 | 10.8754 | 11.0292 | 10.8448 | 10.8535 | 10.8535 | 10.9719 | 9.8849 | 10.7631 | 10.3576 | 10.7789 |
| 204662_at | 9738 | CCP110 | centriolar coiled coil protein 110kDa | 0.4598 | 0.1138 | 0.5563 | 8.3197 | 8.249 | 8.3178 | 8.4101 | 8.4134 | 8.0517 | 8.0781 | 8.5749 | 7.7011 | 7.8928 | 8.4612 | 8.1521 |
| 217956_s_at | 58478 | ENOPH1 | enolase-phosphatase 1 | 0.4517 | 0.08383 | 0.5118 | 10.8535 | 10.584 | 10.8218 | 10.8365 | 10.6469 | 10.4185 | 10.3927 | 10.5646 | 10.2308 | 10.7927 | 10.5393 | 6.1727 |
| 217873_at | 51719 | CAB39 | calcium binding protein 39 | 0.4495 | 0.2156 | 0.6689 | 7.8745 | 7.7856 | 7.848 | 7.8826 | 7.6126 | 7.2396 | 6.9462 | 7.9327 | 7.5198 | 6.3975 | 6.7423 | 6.7539 |
| 201710_at | 4605 | MYBL2 | v-myb myeloblastosis viral oncogene homolog (avian)-like 2 | 0.4452 | 0.1158 | 0.5605 | 7.375 | 7.2836 | 7.4144 | 7.4973 | 7.5244 | 7.6527 | 7.2032 | 7.6723 | 7.3277 | 7.5546 | 7.1703 | 7.3089 |
| 221449_s_at | 81533 | ITFG1 | integrin alpha FG-GAP repeat containing 1 | 0.4446 | 0.2934 | 0.7309 | 7.2836 | 7.2167 | 7.4889 | 7.1924 | 7.0597 | 6.9296 | 6.9214 | 6.9556 | 7.245 | 5.4028 | 7.2733 | 7.1528 |
| 212140_at | 23244 | PDS5A | PDS5, regulator of cohesion maintenance, homolog A (S. cerevisiae) | 0.4426 | 0.1756 | 0.6322 | 9.1578 | 9.2686 | 9.4483 | 9.2936 | 9.1512 | 9.2936 | 9.5496 | 9.3187 | 8.5665 | 9.1555 | 9.2686 | 9.2198 |
| 203117_s_at | 9924 | PAN2 | PAN2 poly(A) specific ribonuclease subunit homolog (S. cerevisiae) | 0.441 | 0.0978 | 0.5314 | 5.8537 | 5.8148 | 6.413 | 6.1796 | 6.1406 | 6.4589 | 5.8625 | 5.9169 | 5.6977 | 5.8825 | 3.9983 | 5.8552 |
| 202433_at | 10237 | SLC35B1 | solute carrier family 35, member B1 | 0.4376 | 0.1856 | 0.637 | 10.5765 | 10.491 | 10.8365 | 10.6038 | 10.6154 | 10.7277 | 9.9925 | 10.5327 | 9.9901 | 10.1697 | 10.4012 | 10.3781 |
| 219390_at | 55033 | FKBP14 | FK506 binding protein 14, 22 kDa | 0.435 | 0.1577 | 0.6071 | 6.1877 | 5.8625 | 6.3308 | 6.4199 | 6.1533 | 6.7968 | 5.8059 | 5.7604 | 5.7319 | 5.6977 | 5.7748 | 5.7835 |
| 219076_s_at | 5827 | PXMP2 | peroxisomal membrane protein 2, 22kDa | 0.4343 | 0.1317 | 0.5804 | 10.2086 | 9.983 | 10.3258 | 9.0983 | 10.0094 | 9.4483 | 8.7683 | 8.7265 | 8.5966 | 9.0859 | 10.0593 | 8.9748 |
| 201591_s_at | 11188 | NISCH | nischarin | 0.4339 | 0.1637 | 0.6157 | 7.6704 | 8.0157 | 8.1402 | 8.1543 | 8.1021 | 8.9136 | 7.8692 | 7.9783 | 7.836 | 7.9445 | 7.7986 | 7.9661 |
| 204131_s_at | 2309 | FOXO3 | forkhead box O3 | 0.4334 | 0.1497 | 0.5982 | 7.7231 | 7.7351 | 7.7011 | 8.0104 | 8.0709 | 7.7856 | 7.4055 | 7.9882 | 7.5064 | 7.5266 | 7.3639 | 7.1397 |
| 205026_at | 6777 | STAT5B | signal transducer and activator of transcription 5B | 0.4317 | 0.1257 | 0.5784 | 4.5716 | 4.6951 | 4.6702 | 4.766 | 4.7924 | 5.0412 | 4.5098 | 4.9937 | 4.6373 | 4.601 | 4.5716 | 4.4407 |
| 203557_s_at | 5092 | PCBD1 | pterin-4 alpha-carbinolamine dehydratase/dimerization cofactor of hepatocyte nuclear factor 1 alpha | 0.4316 | 0.1517 | 0.5982 | 11.1141 | 11.3911 | 11.353 | 11.3234 | 11.4763 | 11.6864 | 11.4472 | 11.1962 | 10.5886 | 11.4763 | 11.237 | 11.4763 |
| 205895_s_at | 9221 | NOLC1 | nucleolar and coiled-body phosphoprotein 1 | 0.4303 | 0.1457 | 0.5982 | 10.7811 | 10.8218 | 10.9664 | 10.0554 | 10.9719 | 10.2455 | 10.1184 | 10.1954 | 10.0612 | 10.3997 | 10.4237 | 10.5393 |
| 214741_at | 7690 | ZNF131 | zinc finger protein 131 | 0.4262 | 0.1297 | 0.5804 | 8.0157 | 8.2578 | 8.0938 | 8.4501 | 8.0446 | 7.9623 | 7.5371 | 7.9445 | 7.9445 | 7.9595 | 7.5528 | 8.231 |
| 206066_s_at | 5889 | RAD51C | RAD51 homolog C (S. cerevisiae) | 0.4239 | 0.2275 | 0.6759 | 7.9131 | 8.0728 | 8.332 | 7.8512 | 7.8205 | 7.9704 | 7.6126 | 7.9916 | 7.685 | 7.9916 | 7.657 | 7.3841 |
| 218755_at | 10112 | KIF20A | kinesin family member 20A | 0.4213 | 0.1816 | 0.6322 | 8.5217 | 8.4555 | 8.502 | 8.4002 | 8.3857 | 8.4481 | 8.6346 | 8.546 | 8.2886 | 8.2423 | 8.272 | 8.2547 |
| 212973_at | 22934 | RPIA | ribose 5-phosphate isomerase A | 0.4213 | 0.1178 | 0.5646 | 11.6864 | 11.5374 | 11.8331 | 12.0917 | 11.7089 | 11.6611 | 11.5842 | 11.6409 | 11.6611 | 11.6611 | 6.2473 | 11.8563 |
| 212330_at | 7027 | TFDP1 | transcription factor Dp-1 | 0.4205 | 0.1477 | 0.5982 | 9.2516 | 8.9691 | 9.2192 | 8.7226 | 8.7457 | 9.0411 | 8.495 | 8.8263 | 8.2084 | 9.0263 | 8.8671 | 8.6391 |
| 201819_at | 949 | SCARB1 | scavenger receptor class B, member 1 | 0.4199 | 0.1277 | 0.5784 | 7.3021 | 6.9488 | 7.872 | 6.7758 | 7.4511 | 6.9757 | 6.5769 | 6.0253 | 6.9335 | 7.3415 | 7.2601 | 6.2209 |
| 217911_s_at | 9531 | BAG3 | BCL2-associated athanogene 3 | 0.4193 | 0.1996 | 0.6529 | 9.4888 | 9.5067 | 9.5038 | 9.7917 | 9.5067 | 8.8195 | 9.2323 | 9.7142 | 9.3187 | 9.1768 | 8.5194 | 8.5919 |
| 201174_s_at | 54386 | TERF2IP | telomeric repeat binding factor 2, interacting protein | 0.4152 | 0.1537 | 0.6012 | 11.4763 | 11.4472 | 11.6611 | 11.6611 | 11.3665 | 11.5626 | 11.1962 | 11.4204 | 11.4966 | 11.617 | 11.4204 | 11.4472 |
| 201078_at | 9375 | TM9SF2 | transmembrane 9 superfamily member 2 | 0.4116 | 0.1597 | 0.6077 | 8.3822 | 8.4025 | 7.9478 | 8.3723 | 8.2704 | 8.0672 | 7.9078 | 8.2633 | 7.7675 | 8.0517 | 8.2128 | 7.7133 |
| 203007_x_at | 10434 | LYPLA1 | lysophospholipase I | 0.4098 | 0.509 | 0.8748 | 9.6875 | 9.5496 | 9.9331 | 9.6038 | 9.6785 | 9.5715 | 9.2876 | 9.8325 | 5.9831 | 6.5054 | 9.7213 | 9.8325 |
| 202633_at | 11073 | TOPBP1 | topoisomerase (DNA) II binding protein 1 | 0.4086 | 0.2036 | 0.655 | 9.5686 | 9.4483 | 9.5363 | 9.4649 | 9.3187 | 9.5814 | 9.3568 | 9.3568 | 9.5009 | 9.4649 | 9.2815 | 9.1036 |
| 209050_s_at | 5900 | RALGDS | ral guanine nucleotide dissociation stimulator | 0.4075 | 0.1517 | 0.5982 | 7.9768 | 8.1808 | 8.1521 | 8.3972 | 8.2886 | 8.2621 | 8.0118 | 8.1089 | 7.7878 | 8.1748 | 8.1089 | 8.0446 |
| 205190_at | 5357 | PLS1 | plastin 1 | 0.407 | 0.1497 | 0.5982 | 9.6038 | 9.3022 | 9.0922 | 9.5067 | 8.7366 | 9.032 | 8.9432 | 9.2936 | 8.7346 | 8.7457 | 8.9622 | 8.9574 |
| 207039_at | 1029 | CDKN2A | cyclin-dependent kinase inhibitor 2A (melanoma, p16, inhibits CDK4) | 0.4046 | 0.003992 | 0.3549 | 11.5842 | 11.5189 | 11.7662 | 11.4763 | 11.5374 | 11.8756 | 11.2259 | 11.353 | 11.1454 | 11.3234 | 11.353 | 10.9929 |
| 200083_at | 23326 | USP22 | ubiquitin specific peptidase 22 | 0.4044 | 0.0479 | 0.442 | 12.1565 | 12.0917 | 12.2004 | 12.019 | 12.3234 | 12.1301 | 11.9863 | 11.9984 | 11.8924 | 12.0917 | 11.809 | 12.1067 |
| 203346_s_at | 22823 | MTF2 | metal response element binding transcription factor 2 | 0.4009 | 0.04192 | 0.4361 | 10.3106 | 10.4772 | 10.6909 | 10.5886 | 10.5886 | 10.4185 | 10.1782 | 10.5248 | 10.1782 | 10.2986 | 10.5015 | 10.3106 |
| 200757_s_at | 813 | CALU | calumenin | 0.3991 | 0.3034 | 0.7309 | 9.776 | 9.6785 | 9.706 | 9.8063 | 9.6609 | 9.7213 | 9.1831 | 9.7957 | 9.2936 | 9.3433 | 9.9192 | 9.2936 |
| 214074_s_at | 2017 | CTTN | cortactin | 0.3991 | 0.2735 | 0.717 | 5.3457 | 5.6326 | 8.495 | 5.7669 | 5.6633 | 5.5863 | 5.9102 | 5.7242 | 5.2837 | 5.4862 | 5.4123 | 5.3682 |
| 201158_at | 4836 | NMT1 | N-myristoyltransferase 1 | 0.3965 | 0.1796 | 0.6322 | 7.9704 | 7.5805 | 7.8575 | 7.9822 | 8.359 | 7.6311 | 7.6257 | 8.0446 | 7.5244 | 7.5761 | 7.4336 | 8.1305 |
| 202696_at | 9943 | OXSR1 | oxidative-stress responsive 1 | 0.3949 | 0.2275 | 0.6759 | 7.9327 | 7.8928 | 8.0415 | 8.0321 | 7.8826 | 7.8245 | 7.583 | 7.3974 | 7.5013 | 7.6005 | 7.7867 | 7.5288 |
| 217871_s_at | 4282 | MIF | macrophage migration inhibitory factor (glycosylation-inhibiting factor) | 0.3947 | 0.4092 | 0.8101 | 13.9353 | 14.3602 | 14.3602 | 14.0921 | 14.3602 | 14.3602 | 14.0921 | 14.3602 | 12.9163 | 13.363 | 14.0921 | 14.3602 |
| 217763_s_at | 11031 | RAB31 | RAB31, member RAS oncogene family | 0.3942 | 0.1517 | 0.5982 | 7.8706 | 7.7535 | 7.8245 | 7.9019 | 9.0577 | 8.9295 | 7.9019 | 8.7411 | 7.5612 | 7.5506 | 7.795 | 7.4033 |
| 219888_at | 6676 | SPAG4 | sperm associated antigen 4 | 0.3938 | 0.1078 | 0.549 | 6.1406 | 6.5746 | 6.687 | 6.4515 | 8.4134 | 7.146 | 6.4014 | 6.051 | 5.6125 | 6.7968 | 5.8783 | 5.8103 |
| 202371_at | 79921 | TCEAL4 | transcription elongation factor A (SII)-like 4 | 0.3874 | 0.2974 | 0.7309 | 10.2496 | 10.2308 | 10.2308 | 10.2496 | 10.2308 | 10.0321 | 9.9925 | 10.2496 | 9.7957 | 9.6129 | 10.1261 | 10.1954 |
| 217777_s_at | 51495 | PTPLAD1 | protein tyrosine phosphatase-like A domain containing 1 | 0.3873 | 0.03593 | 0.4285 | 11.3234 | 11.237 | 11.3911 | 11.1962 | 11.4204 | 11.0907 | 10.8036 | 11.0139 | 10.8535 | 10.9719 | 11.2563 | 11.1291 |
| 202870_s_at | 991 | CDC20 | cell division cycle 20 homolog (S. cerevisiae) | 0.3852 | 0.5329 | 0.8789 | 12.7591 | 12.662 | 12.9163 | 12.9163 | 13.0626 | 13.0053 | 12.9163 | 13.0053 | 5.9126 | 13.0053 | 12.7952 | 12.8313 |
| 203512_at | 27095 | TRAPPC3 | trafficking protein particle complex 3 | 0.3849 | 0.1936 | 0.6441 | 12.3004 | 12.1067 | 12.0594 | 12.0594 | 12.0363 | 11.8924 | 11.9239 | 11.8924 | 11.5189 | 11.9239 | 11.9239 | 11.9984 |
| 202224_at | 1398 | CRK | v-crk sarcoma virus CT10 oncogene homolog (avian) | 0.3817 | 0.1836 | 0.6346 | 8.3107 | 8.3024 | 8.5113 | 8.4664 | 8.4622 | 8.1986 | 7.9285 | 7.3268 | 7.8401 | 8.4169 | 8.0982 | 7.9728 |
| 209774_x_at | 2920 | CXCL2 | chemokine (C-X-C motif) ligand 2 | 0.3799 | 0.1796 | 0.6322 | 4.308 | 4.0871 | 8.7191 | 8.8182 | 4.5225 | 4.3669 | 4.2444 | 4.2178 | 4.2311 | 3.955 | 4.2444 | 4.1172 |
| 208708_x_at | 1983 | EIF5 | eukaryotic translation initiation factor 5 | 0.3789 | 0.1078 | 0.549 | 10.9719 | 11.2563 | 11.3234 | 11.3348 | 11.2078 | 10.8218 | 10.7927 | 11.2078 | 10.5646 | 10.4579 | 11.0744 | 6.731 |
| 220643_s_at | 55179 | FAIM | Fas apoptotic inhibitory molecule | 0.378 | 0.3273 | 0.748 | 8.8096 | 8.9883 | 8.7822 | 8.9136 | 9.0472 | 8.2259 | 7.3415 | 7.4942 | 8.5096 | 8.028 | 8.7574 | 8.6639 |
| 212189_s_at | 25839 | COG4 | component of oligomeric golgi complex 4 | 0.3752 | 0.1816 | 0.6322 | 7.4688 | 7.5612 | 7.4873 | 7.4055 | 7.1171 | 7.5288 | 6.7922 | 7.2601 | 7.1703 | 6.9214 | 7.2667 | 7.0649 |
| 208478_s_at | 581 | BAX | BCL2-associated X protein | 0.375 | 0.1417 | 0.5898 | 9.2686 | 9.5657 | 8.4049 | 9.3099 | 8.7781 | 8.5167 | 9.3908 | 8.7411 | 8.581 | 9.2876 | 8.4825 | 8.3388 |
| 202240_at | 5347 | PLK1 | polo-like kinase 1 | 0.3725 | 0.2196 | 0.6689 | 9.6785 | 7.3089 | 9.8445 | 9.623 | 9.7249 | 9.8129 | 7.8745 | 8.5715 | 9.0088 | 8.633 | 9.3568 | 9.3099 |
| 201339_s_at | 6342 | SCP2 | sterol carrier protein 2 | 0.3717 | 0.1756 | 0.6322 | 7.3914 | 8.4338 | 8.0415 | 7.5385 | 7.1171 | 7.2142 | 7.2247 | 7.6126 | 7.114 | 7.2667 | 7.3415 | 6.9462 |
| 204531_s_at | 672 | BRCA1 | breast cancer 1, early onset | 0.3703 | 0.2196 | 0.6689 | 8.3656 | 8.4101 | 8.5852 | 8.6284 | 8.3443 | 8.1899 | 8.249 | 8.6639 | 8.1986 | 8.446 | 8.3368 | 7.9327 |
| 221549_at | 83743 | GRWD1 | glutamate-rich WD repeat containing 1 | 0.363 | 0.2156 | 0.6689 | 7.2167 | 7.3841 | 8.205 | 7.5001 | 7.5288 | 8.3088 | 6.8458 | 7.1364 | 6.8432 | 8.0145 | 7.9512 | 6.8406 |
| 217989_at | 51170 | HSD17B11 | hydroxysteroid (17-beta) dehydrogenase 11 | 0.3626 | 0.2036 | 0.655 | 9.6213 | 9.3768 | 9.4237 | 9.4008 | 9.5117 | 9.3568 | 8.9622 | 9.2686 | 8.8492 | 9.2936 | 9.9229 | 9.0263 |
| 202153_s_at | 23636 | NUP62 | nucleoporin 62kDa | 0.3594 | 0.3333 | 0.7505 | 9.3333 | 9.2936 | 9.4264 | 9.238 | 8.9158 | 9.123 | 9.2204 | 9.2516 | 8.47 | 8.6983 | 9.4092 | 9.3603 |
| 203105_s_at | 10059 | DNM1L | dynamin 1-like | 0.3585 | 0.6028 | 0.9003 | 9.9229 | 9.8325 | 10.0005 | 9.8973 | 9.9331 | 10.1261 | 10.2986 | 10.2223 | 9.7285 | 9.8063 | 4.3164 | 10.4133 |
| 221514_at | 10813 | UTP14A | UTP14, U3 small nucleolar ribonucleoprotein, homolog A (yeast) | 0.3572 | 0.2236 | 0.6759 | 9.101 | 9.2633 | 8.3822 | 8.3516 | 9.2323 | 9.0733 | 8.2259 | 9.0733 | 9.5715 | 8.6688 | 8.5966 | 7.9539 |
| 215093_at | 50814 | NSDHL | NAD(P) dependent steroid dehydrogenase-like | 0.3566 | 0.2056 | 0.6592 | 9.0656 | 8.2172 | 8.7366 | 8.4622 | 9.2204 | 9.41 | 8.2547 | 9.1555 | 8.0157 | 8.231 | 8.1688 | 8.3443 |
| 207181_s_at | 840 | CASP7 | caspase 7, apoptosis-related cysteine peptidase | 0.3563 | 0.1896 | 0.6395 | 8.5472 | 8.205 | 8.5966 | 8.4555 | 8.3972 | 8.4481 | 8.0043 | 8.1874 | 5.7423 | 8.1475 | 8.3248 | 8.0728 |
| 209215_at | 10227 | MFSD10 | major facilitator superfamily domain containing 10 | 0.3536 | 0.3114 | 0.7338 | 7.9768 | 11.7089 | 8.1475 | 8.4399 | 8.4555 | 8.1127 | 8.231 | 8.3248 | 8.1089 | 8.115 | 8.0157 | 8.2354 |
| 203696_s_at | 5982 | RFC2 | replication factor C (activator 1) 2, 40kDa | 0.3499 | 0.3353 | 0.7505 | 8.1688 | 8.359 | 8.3178 | 8.2886 | 7.9566 | 8.6944 | 8.0375 | 8.4101 | 7.8144 | 8.2519 | 8.3839 | 8.115 |
| 201608_s_at | 11137 | PWP1 | PWP1 homolog (S. cerevisiae) | 0.3489 | 0.3493 | 0.7711 | 9.8546 | 9.983 | 10.0602 | 9.9689 | 10.2223 | 9.983 | 9.9689 | 10.1697 | 9.8376 | 10.0094 | 9.6056 | 9.5117 |
| 217766_s_at | 23585 | TMEM50A | transmembrane protein 50A | 0.3485 | 0.2575 | 0.7049 | 10.5962 | 10.6326 | 10.7789 | 10.6909 | 10.4579 | 10.4396 | 10.5248 | 10.491 | 10.1489 | 10.5248 | 10.4579 | 10.5802 |
| 202900_s_at | 4927 | NUP88 | nucleoporin 88kDa | 0.3459 | 0.3633 | 0.7817 | 11.9351 | 12.0363 | 12.0917 | 12.0363 | 11.9984 | 12.0594 | 12.0917 | 12.2244 | 11.9239 | 11.8563 | 12.0363 | 11.6864 |
| 202230_s_at | 10523 | CHERP | calcium homeostasis endoplasmic reticulum protein | 0.3447 | 0.2236 | 0.6759 | 9.3685 | 9.0859 | 9.4345 | 9.7719 | 9.2467 | 9.2754 | 9.0581 | 9.2467 | 8.9295 | 9.2437 | 9.1315 | 8.9158 |
| 209507_at | 6119 | RPA3 | replication protein A3, 14kDa | 0.3366 | 0.3573 | 0.7765 | 8.9841 | 8.8128 | 8.9841 | 8.7366 | 8.5715 | 8.4775 | 8.3248 | 8.4338 | 8.0709 | 8.5282 | 8.6247 | 8.3754 |
| 202260_s_at | 6812 | STXBP1 | syntaxin binding protein 1 | 0.3365 | 0.2675 | 0.7128 | 5.938 | 5.5037 | 5.8783 | 5.9277 | 5.2689 | 6.0427 | 5.8825 | 5.5664 | 5.0692 | 5.244 | 5.2092 | 5.3176 |
| 201976_s_at | 4651 | MYO10 | myosin X | 0.3356 | 0.2655 | 0.7094 | 7.8144 | 7.0954 | 7.1746 | 8.0375 | 7.4732 | 7.6311 | 6.9893 | 6.9803 | 7.6938 | 7.1397 | 7.8706 | 6.7808 |
| 203050_at | 7158 | TP53BP1 | tumor protein p53 binding protein 1 | 0.3353 | 0.2555 | 0.7049 | 8.6171 | 8.7732 | 8.6171 | 8.8492 | 8.8527 | 8.6247 | 8.9671 | 8.9869 | 8.3368 | 8.5113 | 8.5749 | 8.557 |
| 213135_at | 7074 | TIAM1 | T-cell lymphoma invasion and metastasis 1 | 0.3324 | 0.1936 | 0.6441 | 7.2032 | 7.3881 | 7.5371 | 7.4558 | 7.3188 | 8.1543 | 7.1202 | 6.8468 | 7.1421 | 8.546 | 6.9214 | 7.0007 |
| 201146_at | 4780 | NFE2L2 | nuclear factor (erythroid-derived 2)-like 2 | 0.3321 | 0.2934 | 0.7309 | 8.5113 | 8.5472 | 8.4501 | 8.4815 | 8.6997 | 8.3368 | 8.633 | 7.3806 | 8.9432 | 8.7116 | 9.0088 | 8.167 |
| 202825_at | 291 | SLC25A4 | solute carrier family 25 (mitochondrial carrier; adenine nucleotide translocator), member 4 | 0.3308 | 0.3034 | 0.7309 | 10.2959 | 10.2086 | 10.3682 | 10.0946 | 10.1602 | 10.0188 | 10.1261 | 9.8234 | 9.6628 | 9.9229 | 9.9759 | 9.9901 |
| 203367_at | 11072 | DUSP14 | dual specificity phosphatase 14 | 0.3301 | 0.2735 | 0.717 | 6.5993 | 7.0042 | 6.8597 | 6.673 | 6.4515 | 6.7289 | 6.4307 | 6.9922 | 6.7726 | 6.5993 | 6.2691 | 6.2911 |
| 217867_x_at | 25825 | BACE2 | beta-site APP-cleaving enzyme 2 | 0.328 | 0.2176 | 0.6689 | 8.2172 | 6.9073 | 7.1856 | 7.2559 | 7.2247 | 7.2621 | 6.873 | 7.2343 | 6.289 | 7.0275 | 6.6276 | 6.873 |
| 201121_s_at | 10857 | PGRMC1 | progesterone receptor membrane component 1 | 0.3272 | 0.3154 | 0.7373 | 12.5889 | 12.7071 | 12.7591 | 12.7071 | 12.4958 | 12.662 | 12.4097 | 12.9163 | 12.465 | 12.4958 | 12.5889 | 12.2771 |
| 212591_at | 23029 | RBM34 | RNA binding motif protein 34 | 0.3267 | 0.1876 | 0.6394 | 10.7927 | 10.7631 | 10.7415 | 10.5015 | 10.5113 | 10.5765 | 10.0612 | 10.4396 | 10.2552 | 10.7277 | 10.6469 | 10.3997 |
| 200929_at | 10972 | TMED10 | transmembrane emp24-like trafficking protein 10 (yeast) | 0.3255 | 0.3353 | 0.7505 | 10.0946 | 10.2832 | 10.4579 | 10.0688 | 10.2496 | 10.5886 | 9.9759 | 10.2552 | 9.5117 | 9.7719 | 10.3682 | 10.2496 |
| 218249_at | 64429 | ZDHHC6 | zinc finger, DHHC-type containing 6 | 0.3247 | 0.2435 | 0.696 | 8.6025 | 8.8445 | 8.9869 | 9.3333 | 9.0088 | 8.5869 | 8.6983 | 8.8128 | 8.495 | 8.5472 | 8.6688 | 8.4835 |
| 212723_at | 23210 | JMJD6 | jumonji domain containing 6 | 0.3245 | 0.1377 | 0.5865 | 10.8754 | 10.7277 | 10.9533 | 11.3911 | 11.2259 | 11.1291 | 10.891 | 10.7277 | 10.7927 | 10.8448 | 10.8036 | 10.8754 |
| 203119_at | 79080 | CCDC86 | coiled-coil domain containing 86 | 0.3234 | 0.1357 | 0.5865 | 10.4133 | 10.1954 | 10.1782 | 10.518 | 10.2959 | 10.3576 | 10.518 | 10.0946 | 10.1416 | 10.1057 | 10.303 | 10.4689 |
| 201729_s_at | 9703 | KIAA0100 | KIAA0100 | 0.3213 | 0.2874 | 0.7309 | 6.2057 | 6.2855 | 6.731 | 6.7904 | 6.7726 | 6.6825 | 6.2131 | 6.5355 | 6.6348 | 6.5301 | 6.4376 | 6.0914 |
| 212871_at | 8550 | MAPKAPK5 | mitogen-activated protein kinase-activated protein kinase 5 | 0.3169 | 0.3234 | 0.7441 | 8.1475 | 6.9591 | 8.1021 | 8.205 | 7.6365 | 8.3639 | 7.0474 | 6.3758 | 7.0597 | 8.0043 | 8.2259 | 8.0375 |
| 210337_s_at | 47 | ACLY | ATP citrate lyase | 0.3166 | 0.3174 | 0.7373 | 10.6622 | 10.4396 | 10.5765 | 10.6326 | 10.7068 | 10.6909 | 10.0554 | 10.2986 | 9.4345 | 9.9901 | 9.7917 | 10.2586 |
| 218584_at | 79600 | TCTN1 | tectonic family member 1 | 0.3164 | 0.2974 | 0.7309 | 7.6761 | 7.5064 | 7.9244 | 7.7231 | 7.7878 | 7.5761 | 7.4144 | 7.7617 | 7.1768 | 7.4889 | 7.7072 | 7.2967 |
| 205452_at | 9488 | PIGB | phosphatidylinositol glycan anchor biosynthesis, class B | 0.315 | 0.2315 | 0.6759 | 7.9131 | 8.7265 | 7.9945 | 8.0672 | 7.3549 | 7.2247 | 7.4336 | 7.4873 | 7.8144 | 7.4255 | 7.9244 | 7.4732 |
| 201579_at | 2195 | FAT1 | FAT tumor suppressor homolog 1 (Drosophila) | 0.3138 | 0.2515 | 0.7028 | 9.7498 | 9.6056 | 9.6038 | 9.6827 | 9.6359 | 9.2127 | 9.1512 | 9.6171 | 9.4649 | 9.3187 | 9.5238 | 5.432 |
| 217722_s_at | 51335 | NGRN | neugrin, neurite outgrowth associated | 0.3137 | 0.4012 | 0.809 | 6.9488 | 6.6684 | 7.241 | 6.923 | 6.8842 | 6.8574 | 6.8479 | 6.4346 | 6.794 | 6.9522 | 7.1331 | 6.7539 |
| 202188_at | 9688 | NUP93 | nucleoporin 93kDa | 0.3135 | 0.2435 | 0.696 | 9.5894 | 9.7997 | 10.0602 | 9.955 | 9.7561 | 9.4219 | 9.5009 | 9.5894 | 9.2274 | 9.4888 | 9.41 | 9.706 |
| 200614_at | 1213 | CLTC | clathrin, heavy chain (Hc) | 0.3125 | 0.3174 | 0.7373 | 10.2986 | 10.2024 | 10.3469 | 10.2959 | 10.3106 | 9.9133 | 9.9468 | 10.1489 | 9.5414 | 10.0612 | 9.5894 | 10.1261 |
| 200810_s_at | 1153 | CIRBP | cold inducible RNA binding protein | 0.3111 | 0.2794 | 0.7249 | 8.6071 | 8.9454 | 9.2815 | 8.8587 | 8.6391 | 8.8263 | 8.7527 | 8.3178 | 8.6882 | 8.7226 | 8.7346 | 8.9027 |
| 36936_at | 7264 | TSTA3 | tissue specific transplantation antigen P35B | 0.3103 | 0.3094 | 0.7309 | 5.1982 | 5.1828 | 5.2393 | 5.4123 | 5.3457 | 5.1612 | 5.1258 | 5.1982 | 4.5716 | 5.294 | 4.7131 | 5.0263 |
| 200024_at | 6193 | RPS5 | ribosomal protein S5 | 0.3086 | 0.2595 | 0.7049 | 7.795 | 7.6442 | 6.6524 | 7.6938 | 7.5013 | 6.3809 | 6.8236 | 6.1877 | 6.5993 | 6.7205 | 6.3048 | 6.5769 |
| 220091_at | 11182 | SLC2A6 | solute carrier family 2 (facilitated glucose transporter), member 6 | 0.3076 | 0.3154 | 0.7373 | 9.9531 | 10.1057 | 10.112 | 9.8376 | 9.9901 | 9.776 | 9.7561 | 9.9901 | 9.7719 | 10.0946 | 10.0005 | 9.7373 |
| 217933_s_at | 51056 | LAP3 | leucine aminopeptidase 3 | 0.3072 | 0.3054 | 0.7309 | 11.3348 | 6.6032 | 11.4048 | 11.0034 | 11.1962 | 11.4048 | 10.9533 | 11.3911 | 5.7637 | 5.7319 | 6.2131 | 11.617 |
| 201503_at | 10146 | G3BP1 | GTPase activating protein (SH3 domain) binding protein 1 | 0.3063 | 0.495 | 0.8692 | 10.3359 | 10.262 | 10.262 | 10.1954 | 10.3576 | 9.983 | 10.1489 | 10.2382 | 9.1555 | 10.2086 | 10.0254 | 9.8546 |
| 218001_at | 51116 | MRPS2 | mitochondrial ribosomal protein S2 | 0.3043 | 0.3054 | 0.7309 | 10.4012 | 10.6038 | 10.9381 | 10.7789 | 10.8036 | 10.7518 | 10.6038 | 10.5765 | 10.4237 | 10.6909 | 10.7415 | 10.2586 |
| 210058_at | 5603 | MAPK13 | mitogen-activated protein kinase 13 | 0.3043 | 0.2894 | 0.7309 | 5.8904 | 5.5138 | 4.5899 | 4.5613 | 4.8362 | 4.9864 | 5.4641 | 5.5514 | 4.8822 | 4.5509 | 4.9657 | 4.7983 |
| 217934_x_at | 10273 | STUB1 | STIP1 homology and U-box containing protein 1, E3 ubiquitin protein ligase | 0.3014 | 0.3074 | 0.7309 | 11.9984 | 11.8563 | 11.7318 | 11.8563 | 11.8563 | 12.3753 | 11.8924 | 12.0917 | 11.4763 | 11.6409 | 11.7843 | 11.7662 |
| 217900_at | 55699 | IARS2 | isoleucyl-tRNA synthetase 2, mitochondrial | 0.3008 | 0.3333 | 0.7505 | 10.584 | 10.5765 | 10.8036 | 10.6579 | 10.8365 | 10.6469 | 10.6579 | 10.7415 | 10.5765 | 10.6326 | 10.3781 | 10.7415 |
| 218050_at | 51569 | UFM1 | ubiquitin-fold modifier 1 | 0.3004 | 0.3513 | 0.7738 | 8.9351 | 9.0397 | 9.123 | 9.0656 | 9.0263 | 8.9027 | 8.8748 | 8.9375 | 8.502 | 8.7781 | 9.0023 | 8.7265 |
| 202413_s_at | 7398 | USP1 | ubiquitin specific peptidase 1 | 0.2992 | 0.3174 | 0.7373 | 9.2204 | 9.0088 | 9.2127 | 9.1386 | 9.0411 | 8.5472 | 8.6247 | 8.9167 | 8.5749 | 8.9136 | 8.9415 | 8.502 |
| 201572_x_at | 1635 | DCTD | dCMP deaminase | 0.2976 | 0.2874 | 0.7309 | 8.495 | 8.2886 | 8.7265 | 8.7157 | 8.6997 | 8.6048 | 8.1748 | 8.4985 | 8.1748 | 8.3158 | 8.115 | 8.3197 |
| 202318_s_at | 26054 | SENP6 | SUMO1/sentrin specific peptidase 6 | 0.2974 | 0.3014 | 0.7309 | 8.9691 | 9.1601 | 9.1768 | 9.3768 | 8.8096 | 8.7878 | 9.2127 | 8.9432 | 8.7457 | 8.6247 | 9.1934 | 8.9295 |
| 209166_s_at | 4125 | MAN2B1 | mannosidase, alpha, class 2B, member 1 | 0.2972 | 0.3074 | 0.7309 | 7.0597 | 6.6373 | 7.0284 | 7.0333 | 6.9645 | 6.8703 | 6.5159 | 7.0111 | 6.4541 | 6.4053 | 6.4515 | 6.3601 |
| 201260_s_at | 6856 | SYPL1 | synaptophysin-like 1 | 0.2969 | 0.4391 | 0.8275 | 10.6469 | 10.5886 | 10.6038 | 10.8036 | 10.5646 | 10.9533 | 10.6716 | 10.6038 | 10.262 | 9.8325 | 10.7277 | 10.6909 |
| 203338_at | 5529 | PPP2R5E | protein phosphatase 2, regulatory subunit B', epsilon isoform | 0.2967 | 0.521 | 0.8768 | 9.0581 | 8.9748 | 9.3433 | 9.0922 | 9.2079 | 10.0554 | 9.6609 | 9.537 | 9.2686 | 9.109 | 6.486 | 9.5067 |
| 202184_s_at | 55746 | NUP133 | nucleoporin 133kDa | 0.2967 | 0.2934 | 0.7309 | 10.1261 | 9.7373 | 10.0321 | 10.1261 | 10.2024 | 10.1782 | 9.8849 | 9.8546 | 9.954 | 9.9759 | 9.5455 | 9.9162 |
| 201560_at | 25932 | CLIC4 | chloride intracellular channel 4 | 0.2967 | 0.3034 | 0.7309 | 11.8756 | 12.1763 | 12.3004 | 12.2342 | 12.4511 | 12.0363 | 12.2342 | 12.1763 | 11.9984 | 12.019 | 12.019 | 12.0363 |
| 206173_x_at | 2553 | GABPB1 | GA binding protein transcription factor, beta subunit 1 | 0.2958 | 0.2794 | 0.7249 | 8.9869 | 9.3685 | 9.3768 | 9.1555 | 9.1623 | 9.0581 | 8.8981 | 9.1315 | 8.7844 | 8.8209 | 9.1601 | 8.8492 |
| 201620_at | 8720 | MBTPS1 | membrane-bound transcription factor peptidase, site 1 | 0.2942 | 0.3134 | 0.7367 | 6.5023 | 6.3524 | 6.4053 | 6.2624 | 6.4929 | 6.3975 | 6.2011 | 6.217 | 6.3105 | 6.6638 | 6.2758 | 6.2624 |
| 201959_s_at | 23077 | MYCBP2 | MYC binding protein 2 | 0.2937 | 0.2455 | 0.696 | 9.3099 | 9.2192 | 8.9454 | 9.0193 | 9.1265 | 8.4579 | 8.8561 | 8.5996 | 8.47 | 8.9869 | 8.5096 | 8.748 |
| 207993_s_at | 11261 | CHP | calcium binding protein P22 | 0.2913 | 0.3313 | 0.7505 | 9.0922 | 9.1512 | 9.4649 | 9.2274 | 9.1831 | 9.0765 | 8.8671 | 9.2754 | 8.7366 | 8.8748 | 9.1143 | 9.1315 |
| 209045_at | 7511 | XPNPEP1 | X-prolyl aminopeptidase (aminopeptidase P) 1, soluble | 0.2895 | 0.4251 | 0.8201 | 8.4339 | 6.9645 | 8.4338 | 8.321 | 8.4338 | 7.7856 | 6.0275 | 6.1608 | 7.6527 | 8.1055 | 8.0874 | 6.299 |
| 217398_x_at | 2597 | GAPDH | glyceraldehyde-3-phosphate dehydrogenase | 0.2887 | 0.1776 | 0.6322 | 15 | 15 | 15 | 15 | 15 | 15 | 15 | 15 | 14.6801 | 15 | 15 | 15 |
| 219170_at | 79187 | FSD1 | fibronectin type III and SPRY domain containing 1 | 0.2878 | 0.5689 | 0.8876 | 8.359 | 8.2886 | 8.1881 | 7.8401 | 8.446 | 8.4338 | 7.6126 | 6.3247 | 8.0874 | 6.8551 | 8.1808 | 8.446 |
| 203167_at | 7077 | TIMP2 | TIMP metallopeptidase inhibitor 2 | 0.2875 | 0.3533 | 0.7756 | 11.7843 | 11.8331 | 12.019 | 11.8331 | 11.8924 | 11.9462 | 11.809 | 11.9863 | 11.617 | 11.809 | 11.7089 | 11.5842 |
| 212648_at | 54505 | DHX29 | DEAH (Asp-Glu-Ala-His) box polypeptide 29 | 0.2866 | 0.2994 | 0.7309 | 7.3277 | 7.9661 | 7.4911 | 8.0517 | 7.9185 | 7.1962 | 8.1021 | 7.4385 | 7.1331 | 7.0176 | 6.9123 | 7.2142 |
| 204489_s_at | 960 | CD44 | CD44 molecule (Indian blood group) | 0.2863 | 0.4511 | 0.8324 | 10.3074 | 9.1196 | 8.5749 | 9.3515 | 10.7631 | 10.8365 | 11.5626 | 10.4689 | 11.1378 | 10.4499 | 4.5098 | 5.641 |
| 210115_at | 116832 | RPL39L | ribosomal protein L39-like | 0.2835 | 0.2655 | 0.7094 | 7.0007 | 7.0535 | 7.0411 | 5.7669 | 7.1877 | 5.9488 | 5.3609 | 7.0623 | 5.4123 | 6.8157 | 5.8229 | 7.2064 |
| 209662_at | 1070 | CETN3 | centrin, EF-hand protein, 3 | 0.283 | 0.3633 | 0.7817 | 9.8325 | 10.1385 | 9.9689 | 9.955 | 9.9531 | 9.537 | 9.2965 | 10.045 | 9.4888 | 9.5537 | 9.6129 | 9.2467 |
| 201270_x_at | 23386 | NUDCD3 | NudC domain containing 3 | 0.2827 | 0.3653 | 0.7817 | 9.0472 | 7.9623 | 8.3623 | 9.1665 | 8.1348 | 8.5919 | 8.4002 | 8.748 | 7.7856 | 7.6545 | 8.03 | 7.9822 |
| 204781_s_at | 355 | FAS | Fas (TNF receptor superfamily, member 6) | 0.2825 | 0.3593 | 0.7791 | 4.685 | 4.2682 | 5.0107 | 4.5613 | 4.4955 | 4.3308 | 4.2839 | 4.5808 | 4.4486 | 4.5161 | 4.65 | 4.4812 |
| 209095_at | 1738 | DLD | dihydrolipoamide dehydrogenase | 0.2797 | 0.4232 | 0.8179 | 11.1962 | 11.0139 | 11.0292 | 11.0907 | 10.9533 | 10.7112 | 10.6326 | 10.8218 | 10.5146 | 10.9184 | 10.8448 | 11.0543 |
| 214710_s_at | 891 | CCNB1 | cyclin B1 | 0.2795 | 0.8902 | 0.9793 | 12.0363 | 11.9239 | 12.1301 | 12.1067 | 11.9462 | 12.019 | 12.3234 | 12.1301 | 12.3753 | 12.4097 | 8.633 | 9.41 |
| 204849_at | 10732 | TCFL5 | transcription factor-like 5 (basic helix-loop-helix) | 0.2794 | 0.3074 | 0.7309 | 6.0359 | 5.294 | 6.0544 | 5.3176 | 5.1321 | 4.9477 | 5.001 | 5.0692 | 5.2393 | 5.1062 | 5.0412 | 5.7669 |
| 202776_at | 30836 | DNTTIP2 | deoxynucleotidyltransferase, terminal, interacting protein 2 | 0.2782 | 0.2595 | 0.7049 | 12.0594 | 12.2244 | 11.8563 | 11.9239 | 11.809 | 11.7089 | 11.7662 | 11.9239 | 11.809 | 11.7318 | 11.7662 | 11.9462 |
| 201014_s_at | 10606 | PAICS | phosphoribosylaminoimidazole carboxylase, phosphoribosylaminoimidazole succinocarboxamide synthetase | 0.278 | 0.3054 | 0.7309 | 8.3972 | 8.2354 | 8.8263 | 8.6338 | 8.332 | 8.3408 | 8.0709 | 8.5194 | 7.9512 | 7.9078 | 8.03 | 8.0672 |
| 202022_at | 230 | ALDOC | aldolase C, fructose-bisphosphate | 0.2747 | 0.3792 | 0.7942 | 5.6606 | 5.7423 | 5.3755 | 5.6458 | 5.4454 | 5.3875 | 5.6633 | 5.341 | 5.3293 | 5.244 | 5.641 | 5.5732 |
| 202246_s_at | 1019 | CDK4 | cyclin-dependent kinase 4 | 0.2744 | 0.3553 | 0.7756 | 11.5189 | 11.5842 | 11.7089 | 11.5189 | 11.7318 | 11.617 | 11.3911 | 11.6864 | 11.3665 | 11.4334 | 11.2259 | 11.4048 |
| 212300_at | 200081 | TXLNA | taxilin alpha | 0.2742 | 0.4291 | 0.8229 | 11.7662 | 11.6864 | 11.7843 | 11.7318 | 11.8331 | 11.8331 | 11.5189 | 11.8331 | 11.4472 | 11.8924 | 11.8756 | 11.8331 |
| 204061_at | 5613 | PRKX | protein kinase, X-linked | 0.2729 | 0.3832 | 0.7956 | 6.6513 | 6.3388 | 6.2326 | 8.5472 | 6.1242 | 6.1406 | 6.0544 | 5.8825 | 6.6502 | 6.5524 | 6.4199 | 6.3872 |
| 209899_s_at | 22827 | PUF60 | poly-U binding splicing factor 60KDa | 0.2728 | 0.3413 | 0.7552 | 12.4097 | 12.4097 | 12.4958 | 12.4511 | 12.5517 | 12.4958 | 12.2771 | 12.3455 | 12.019 | 12.2771 | 12.2004 | 12.4097 |
| 212051_at | 147179 | WIPF2 | WAS/WASL interacting protein family, member 2 | 0.2726 | 0.3553 | 0.7756 | 8.0672 | 8.495 | 8.9681 | 8.9454 | 8.7346 | 8.5096 | 8.801 | 8.2018 | 8.2704 | 8.0348 | 7.9916 | 8.6048 |
| 221081_s_at | 79961 | DENND2D | DENN/MADD domain containing 2D | 0.2721 | 0.3852 | 0.7956 | 5.8324 | 5.7559 | 5.607 | 5.7669 | 5.4553 | 5.7166 | 5.4862 | 5.1707 | 6.0155 | 5.1384 | 5.6633 | 5.1707 |
| 201460_at | 9261 | MAPKAPK2 | mitogen-activated protein kinase-activated protein kinase 2 | 0.2709 | 0.2874 | 0.7309 | 8.4835 | 8.0043 | 9.9658 | 8.3857 | 8.6025 | 9.7997 | 7.9802 | 8.3839 | 8.5356 | 8.4338 | 7.9822 | 8.1808 |
| 214404_x_at | 25803 | SPDEF | SAM pointed domain containing ets transcription factor | 0.2686 | 0.5848 | 0.8951 | 7.9974 | 8.1305 | 8.272 | 8.0555 | 7.9244 | 8.2457 | 7.6536 | 5.8474 | 8.8311 | 8.1375 | 8.2886 | 7.6629 |
| 203246_s_at | 10641 | NPRL2 | nitrogen permease regulator-like 2 (S. cerevisiae) | 0.2671 | 0.3034 | 0.7309 | 7.1681 | 7.9285 | 7.9783 | 7.2256 | 8.0943 | 6.9462 | 7.7576 | 7.685 | 7.0381 | 7.7856 | 7.0745 | 7.5357 |
| 212047_s_at | 26001 | RNF167 | ring finger protein 167 | 0.2655 | 0.4012 | 0.809 | 9.6908 | 9.7498 | 9.6213 | 9.4219 | 9.6827 | 9.7498 | 9.5715 | 9.6908 | 9.2323 | 9.5363 | 9.455 | 9.4483 |
| 205451_at | 4303 | FOXO4 | forkhead box O4 | 0.2616 | 0.3293 | 0.7505 | 8.9432 | 9.0983 | 9.1386 | 9.4345 | 8.8894 | 8.495 | 8.5852 | 9.0055 | 8.5919 | 8.7116 | 8.6497 | 8.6048 |
| 202689_at | 29890 | RBM15B | RNA binding motif protein 15B | 0.2603 | 0.3353 | 0.7505 | 8.1089 | 7.5288 | 8.0104 | 7.6612 | 8.028 | 7.685 | 7.6416 | 7.6044 | 7.5506 | 7.9244 | 7.8826 | 7.9208 |
| 200698_at | 11014 | KDELR2 | KDEL (Lys-Asp-Glu-Leu) endoplasmic reticulum protein retention receptor 2 | 0.26 | 0.3393 | 0.7542 | 6.5894 | 6.3601 | 6.923 | 6.8126 | 6.6781 | 6.4441 | 6.2011 | 6.4805 | 6.1533 | 6.5127 | 6.1957 | 6.4053 |
| 201152_s_at | 4154 | MBNL1 | muscleblind-like (Drosophila) | 0.2598 | 0.3972 | 0.8076 | 8.7878 | 8.78 | 8.5217 | 8.1543 | 8.748 | 9.123 | 8.0536 | 8.296 | 8.5472 | 8.2259 | 8.8981 | 7.7351 |
| 208898_at | 51382 | ATP6V1D | ATPase, H+ transporting, lysosomal 34kDa, V1 subunit D | 0.2528 | 0.5709 | 0.8876 | 10.3469 | 10.1489 | 10.1954 | 10.3781 | 10.2455 | 10.2308 | 9.9531 | 10.0094 | 9.9468 | 10.0321 | 5.8338 | 10.1602 |
| 208687_x_at | 3312 | HSPA8 | heat shock 70kDa protein 8 | 0.2515 | 0.5409 | 0.8817 | 10.7025 | 11.1962 | 11.4966 | 10.8682 | 11.0907 | 11.0139 | 11.0139 | 10.8365 | 10.8036 | 11.0292 | 11.617 | 11.2078 |
| 218206_x_at | 51282 | SCAND1 | SCAN domain containing 1 | 0.2514 | 0.3014 | 0.7309 | 9.1512 | 8.3024 | 8.4555 | 8.3639 | 9.2316 | 9.1036 | 8.028 | 9.3099 | 8.5356 | 8.261 | 8.3443 | 8.0132 |
| 221750_at | 3157 | HMGCS1 | 3-hydroxy-3-methylglutaryl-CoA synthase 1 (soluble) | 0.2501 | 0.6906 | 0.9421 | 6.0527 | 11.0292 | 10.7631 | 10.8754 | 10.8535 | 10.891 | 9.8129 | 6.923 | 9.5894 | 9.5067 | 9.9468 | 9.6247 |
| 202179_at | 642 | BLMH | bleomycin hydrolase | 0.2467 | 0.4212 | 0.8172 | 9.6056 | 9.41 | 9.9192 | 9.5238 | 9.7917 | 9.9211 | 8.9398 | 9.6628 | 9.2192 | 9.2204 | 9.4768 | 10.4318 |
| 212811_x_at | 6509 | SLC1A4 | solute carrier family 1 (glutamate/neutral amino acid transporter), member 4 | 0.244 | 0.3393 | 0.7542 | 4.8822 | 4.9937 | 4.7983 | 4.8651 | 7.499 | 4.7754 | 4.7131 | 4.5808 | 4.356 | 4.4486 | 5.116 | 4.8576 |
| 212899_at | 23097 | CDK19 | cyclin-dependent kinase 19 | 0.244 | 0.3673 | 0.7842 | 7.0229 | 7.0745 | 7.1421 | 7.1474 | 10.262 | 10.7789 | 7.0649 | 6.923 | 6.6781 | 7.1445 | 7.0275 | 9.8495 |
| 221732_at | 124583 | CANT1 | calcium activated nucleotidase 1 | 0.2433 | 0.6926 | 0.9421 | 9.995 | 9.6038 | 9.863 | 9.8445 | 9.9133 | 9.863 | 9.7719 | 9.955 | 9.5537 | 9.7373 | 9.7285 | 9.8973 |
| 205202_at | 5110 | PCMT1 | protein-L-isoaspartate (D-aspartate) O-methyltransferase | 0.2432 | 0.4631 | 0.8418 | 11.6409 | 11.3003 | 11.617 | 11.4472 | 11.5189 | 11.4763 | 11.3118 | 11.6611 | 10.9184 | 11.4204 | 11.3003 | 11.4204 |
| 203256_at | 1001 | CDH3 | cadherin 3, type 1, P-cadherin (placental) | 0.2418 | 0.4271 | 0.8207 | 5.0412 | 5.0985 | 5.1707 | 5.4354 | 7.27 | 5.609 | 5.5182 | 5.5278 | 5.5182 | 5.2837 | 5.3047 | 5.5138 |
| 205463_s_at | 5154 | PDGFA | platelet-derived growth factor alpha polypeptide | 0.2414 | 0.3872 | 0.7956 | 7.1331 | 7.0824 | 7.2304 | 7.2343 | 8.2018 | 7.0824 | 8.4664 | 7.0042 | 6.4376 | 6.794 | 6.8597 | 6.8479 |
| 201709_s_at | 8508 | NIPSNAP1 | nipsnap homolog 1 (C. elegans) | 0.2392 | 0.6727 | 0.9345 | 7.2265 | 7.5528 | 7.7617 | 7.6527 | 7.5357 | 7.4435 | 7.3914 | 7.5652 | 6.6562 | 7.0977 | 7.6085 | 7.3377 |
| 204725_s_at | 4690 | NCK1 | NCK adaptor protein 1 | 0.2379 | 0.8403 | 0.9716 | 10.4499 | 10.3576 | 10.4689 | 10.5393 | 10.3074 | 10.4012 | 10.1697 | 10.5393 | 10.1697 | 10.4318 | 10.4133 | 10.3469 |
| 200071_at | 10285 | SMNDC1 | survival motor neuron domain containing 1 | 0.2379 | 0.4032 | 0.8097 | 9.6827 | 9.5814 | 9.5657 | 9.5657 | 9.7142 | 9.3603 | 9.4483 | 9.4649 | 9.2754 | 9.3308 | 9.3515 | 9.5657 |
| 201314_at | 10494 | STK25 | serine/threonine kinase 25 | 0.2373 | 0.4391 | 0.8275 | 10.7415 | 10.6622 | 10.6326 | 10.7518 | 10.584 | 10.1954 | 9.9133 | 10.6622 | 10.2986 | 10.5765 | 10.1697 | 10.491 |
| 201412_at | 26020 | LRP10 | low density lipoprotein receptor-related protein 10 | 0.2344 | 0.3733 | 0.7884 | 6.6348 | 6.8597 | 6.8551 | 6.7289 | 6.8938 | 6.4541 | 6.4541 | 6.3433 | 6.4589 | 6.707 | 7.1228 | 6.2411 |
| 219208_at | 80204 | FBXO11 | F-box protein 11 | 0.2335 | 0.4192 | 0.8166 | 7.4296 | 7.3881 | 6.3601 | 7.1587 | 7.0977 | 7.2064 | 7.6005 | 7.3881 | 6.5942 | 6.0004 | 6.157 | 7.3307 |
| 201968_s_at | 5236 | PGM1 | phosphoglucomutase 1 | 0.2333 | 0.4451 | 0.8324 | 8.0709 | 8.0781 | 8.3723 | 8.296 | 8.321 | 7.9852 | 7.7856 | 8.1021 | 7.5385 | 8.0709 | 8.2423 | 8.1021 |
| 203138_at | 8520 | HAT1 | histone acetyltransferase 1 | 0.2324 | 0.5848 | 0.8951 | 9.0574 | 9.2323 | 9.3644 | 8.9691 | 8.9691 | 8.6808 | 8.6099 | 9.1036 | 9.2633 | 9.1386 | 9.2467 | 9.0922 |
| 202990_at | 5836 | PYGL | phosphorylase, glycogen, liver | 0.2315 | 0.4052 | 0.8101 | 10.0094 | 10.045 | 10.7811 | 10.5646 | 10.0321 | 10.2959 | 10.4689 | 10.1782 | 10.2086 | 9.7561 | 10.4841 | 10.0554 |
| 208611_s_at | 6709 | SPTAN1 | spectrin, alpha, non-erythrocytic 1 (alpha-fodrin) | 0.2312 | 0.3653 | 0.7817 | 5.3085 | 5.2837 | 5.5446 | 5.2689 | 5.2974 | 5.3008 | 5.2182 | 5.2137 | 5.2488 | 5.1948 | 5.1494 | 5.0515 |
| 202468_s_at | 8727 | CTNNAL1 | catenin (cadherin-associated protein), alpha-like 1 | 0.2309 | 0.3733 | 0.7884 | 8.7346 | 8.3857 | 8.2216 | 9.3568 | 9.3768 | 8.2621 | 8.0321 | 8.2128 | 8.7293 | 8.3248 | 8.2354 | 8.0555 |
| 203405_at | 8624 | PSMG1 | proteasome (prosome, macropain) assembly chaperone 1 | 0.2289 | 0.4711 | 0.85 | 11.1661 | 10.9719 | 11.1962 | 11.353 | 11.2563 | 11.0543 | 10.6622 | 11.1378 | 10.4579 | 11.1454 | 10.9533 | 10.9533 |
| 201693_s_at | 1958 | EGR1 | early growth response 1 | 0.2255 | 0.4032 | 0.8097 | 6.3588 | 6.1444 | 6.3872 | 6.5365 | 6.289 | 6.0231 | 5.803 | 5.609 | 7.6044 | 6.1155 | 5.5182 | 5.5732 |
| 218051_s_at | 64943 | NT5DC2 | 5'-nucleotidase domain containing 2 | 0.2248 | 0.3892 | 0.7964 | 9.0351 | 9.2754 | 9.1555 | 9.4092 | 9.4254 | 9.4254 | 9.4092 | 9.2192 | 9.1036 | 9.1906 | 9.0694 | 9.0383 |
| 202431_s_at | 4609 | MYC | v-myc myelocytomatosis viral oncogene homolog (avian) | 0.2243 | 0.4531 | 0.8345 | 9.1386 | 8.6983 | 9.1623 | 9.1315 | 9.3022 | 9.1996 | 8.6688 | 9.3009 | 8.871 | 8.9235 | 8.6983 | 8.7226 |
| 203973_s_at | 1052 | CEBPD | CCAAT/enhancer binding protein (C/EBP), delta | 0.224 | 0.4631 | 0.8418 | 9.4254 | 5.9488 | 5.9626 | 5.8825 | 6.217 | 6.1092 | 6.5993 | 6.0359 | 6.836 | 6.54 | 6.6619 | 6.3809 |
| 203857_s_at | 10954 | PDIA5 | protein disulfide isomerase family A, member 5 | 0.2221 | 0.3633 | 0.7817 | 5.2393 | 5.5732 | 5.0884 | 5.5138 | 5.5548 | 4.7091 | 5.1948 | 5.3933 | 5.166 | 5.473 | 4.8469 | 5.1948 |
| 202125_s_at | 66008 | TRAK2 | trafficking protein, kinesin binding 2 | 0.2219 | 0.4052 | 0.8101 | 6.6513 | 6.4376 | 6.9198 | 6.6524 | 6.3247 | 6.0877 | 5.8229 | 6.5054 | 5.9035 | 6.0329 | 6.1727 | 6.1533 |
| 201202_at | 5111 | PCNA | proliferating cell nuclear antigen | 0.2211 | 0.9721 | 0.9921 | 13.363 | 13.9353 | 13.9353 | 13.363 | 13.9353 | 13.9353 | 13.9353 | 13.9353 | 13.363 | 13.6371 | 13.6371 | 13.9353 |
| 212815_at | 10973 | ASCC3 | activating signal cointegrator 1 complex subunit 3 | 0.2188 | 0.509 | 0.8748 | 7.4144 | 7.6168 | 7.7214 | 7.7878 | 7.3806 | 7.4732 | 7.5244 | 7.7986 | 7.3848 | 7.685 | 7.4732 | 7.6527 |
| 204297_at | 5289 | PIK3C3 | phosphoinositide-3-kinase, class 3 | 0.2186 | 0.493 | 0.8692 | 7.836 | 7.7733 | 7.7986 | 7.9238 | 7.7461 | 7.9238 | 7.6595 | 7.9078 | 7.499 | 7.7509 | 7.8094 | 7.7617 |
| 205034_at | 9134 | CCNE2 | cyclin E2 | 0.2174 | 0.6786 | 0.9361 | 7.146 | 7.1331 | 7.1228 | 7.1331 | 7.3549 | 6.8938 | 6.9214 | 6.9475 | 7.2601 | 7.4188 | 7.2343 | 7.0042 |
| 207842_s_at | 22794 | CASC3 | cancer susceptibility candidate 3 | 0.2161 | 0.5269 | 0.8768 | 8.4643 | 8.6882 | 8.7116 | 8.8209 | 8.9398 | 8.5715 | 8.7457 | 8.6433 | 7.9974 | 8.0906 | 8.3606 | 8.3822 |
| 201767_s_at | 60528 | ELAC2 | elaC homolog 2 (E. coli) | 0.2143 | 0.479 | 0.8596 | 11.7089 | 11.4334 | 11.5842 | 11.5374 | 11.4048 | 11.5189 | 11.4048 | 11.5374 | 11.5626 | 11.5842 | 11.5842 | 11.4966 |
| 207808_s_at | 5627 | PROS1 | protein S (alpha) | 0.2135 | 0.4192 | 0.8166 | 5.5328 | 5.5732 | 5.341 | 5.0739 | 5.5514 | 4.8576 | 4.5225 | 5.736 | 4.9166 | 5.1494 | 5.2182 | 5.4992 |
| 200678_x_at | 2896 | GRN | granulin | 0.2131 | 0.4092 | 0.8101 | 9.0411 | 9.0656 | 9.2995 | 9.41 | 9.3209 | 9.5894 | 8.6171 | 8.9375 | 8.7945 | 9.1601 | 8.9175 | 8.8112 |
| 219952_s_at | 57192 | MCOLN1 | mucolipin 1 | 0.2128 | 0.3733 | 0.7884 | 5.4388 | 9.4219 | 5.5278 | 5.3176 | 5.4159 | 6.1957 | 5.341 | 5.3176 | 5.2278 | 5.6125 | 5.3755 | 5.4123 |
| 212458_at | 200734 | SPRED2 | sprouty-related, EVH1 domain containing 2 | 0.2127 | 0.4391 | 0.8275 | 10.262 | 9.6247 | 9.7719 | 9.2686 | 10.0593 | 10.1489 | 9.5238 | 10.2382 | 9.0383 | 9.3586 | 10.2959 | 9.9689 |
| 209304_x_at | 4616 | GADD45B | growth arrest and DNA-damage-inducible, beta | 0.2123 | 0.3952 | 0.8052 | 7.6761 | 6.4991 | 6.6638 | 6.4199 | 6.095 | 7.4881 | 6.3187 | 6.2326 | 6.54 | 6.4268 | 6.3601 | 6.5894 |
| 212453_at | 26128 | KIAA1279 | KIAA1279 | 0.2102 | 0.3872 | 0.7956 | 8.5096 | 8.4501 | 7.5244 | 8.5749 | 8.0938 | 7.1254 | 7.0745 | 8.4002 | 6.9335 | 8.495 | 7.249 | 8.2704 |
| 202680_at | 2961 | GTF2E2 | general transcription factor IIE, polypeptide 2, beta 34kDa | 0.2098 | 0.515 | 0.8768 | 8.6601 | 8.6476 | 8.8263 | 8.6639 | 8.7191 | 8.3857 | 8.654 | 8.7346 | 8.3107 | 8.4735 | 8.4985 | 8.7157 |
| 212997_s_at | 11011 | TLK2 | tousled-like kinase 2 | 0.2093 | 0.4511 | 0.8324 | 7.1877 | 8.6171 | 8.6639 | 8.748 | 8.6247 | 8.8821 | 6.8082 | 9.2316 | 8.8894 | 8.8096 | 8.8894 | 8.8671 |
| 203188_at | 11041 | B3GNT1 | UDP-GlcNAc:betaGal beta-1,3-N-acetylglucosaminyltransferase 1 | 0.2089 | 0.4591 | 0.8408 | 8.8263 | 8.8358 | 9.0574 | 8.9574 | 8.9295 | 8.6858 | 8.5919 | 8.8613 | 8.446 | 8.748 | 8.78 | 8.6391 |
| 206687_s_at | 5777 | PTPN6 | protein tyrosine phosphatase, non-receptor type 6 | 0.2079 | 0.509 | 0.8748 | 5.0884 | 5.3008 | 5.0692 | 5.1828 | 4.9657 | 4.7983 | 5.0786 | 4.3164 | 5.4641 | 5.0985 | 4.9783 | 4.8161 |
| 202109_at | 23647 | ARFIP2 | ADP-ribosylation factor interacting protein 2 | 0.2076 | 0.4631 | 0.8418 | 8.9454 | 8.7293 | 8.996 | 9.2316 | 8.7913 | 8.801 | 8.8358 | 8.6983 | 8.7012 | 8.5715 | 8.581 | 8.524 |
| 209257_s_at | 9126 | SMC3 | structural maintenance of chromosomes 3 | 0.2074 | 0.4611 | 0.8418 | 9.2192 | 9.3433 | 9.203 | 9.0765 | 9.266 | 8.9167 | 9.135 | 9.2323 | 9.1831 | 9.1196 | 8.9748 | 9.3308 |
| 202555_s_at | 4638 | MYLK | myosin light chain kinase | 0.2057 | 0.4511 | 0.8324 | 4.308 | 4.1889 | 7.7856 | 4.4407 | 4.2996 | 6.9865 | 4.6601 | 6.56 | 4.2996 | 4.65 | 4.4038 | 4.5098 |
| 210151_s_at | 8444 | DYRK3 | dual-specificity tyrosine-(Y)-phosphorylation regulated kinase 3 | 0.2039 | 0.4651 | 0.8439 | 6.5127 | 6.3105 | 6.2658 | 8.1652 | 5.9524 | 6.0818 | 6.4014 | 6.0877 | 6.54 | 6.4361 | 6.6825 | 6.413 |
| 201887_at | 3597 | IL13RA1 | interleukin 13 receptor, alpha 1 | 0.2038 | 0.4112 | 0.8124 | 8.1688 | 8.8209 | 7.7351 | 7.2733 | 7.4889 | 7.3139 | 7.1924 | 7.5312 | 7.9752 | 7.5064 | 7.8144 | 7.1725 |
| 214447_at | 2113 | ETS1 | v-ets erythroblastosis virus E26 oncogene homolog 1 (avian) | 0.2029 | 0.509 | 0.8748 | 7.1397 | 6.6562 | 7.4732 | 7.2654 | 7.1802 | 6.7922 | 7.9704 | 7.241 | 6.3187 | 6.5746 | 6.5746 | 6.5127 |
| 205398_s_at | 4088 | SMAD3 | SMAD family member 3 | 0.2028 | 0.4331 | 0.8257 | 5.3457 | 5.2689 | 5.7166 | 8.9351 | 5.4905 | 5.3682 | 5.1384 | 5.0204 | 5.452 | 5.0412 | 5.1948 | 5.2393 |
| 208716_s_at | 54499 | TMCO1 | transmembrane and coiled-coil domains 1 | 0.2019 | 0.5669 | 0.8876 | 11.809 | 11.7843 | 11.9239 | 11.6864 | 11.7843 | 11.7843 | 11.4763 | 11.7843 | 11.2801 | 11.6864 | 11.7318 | 11.7318 |
| 202809_s_at | 65123 | INTS3 | integrator complex subunit 3 | 0.201 | 0.4391 | 0.8275 | 7.8326 | 7.4732 | 7.7856 | 7.5506 | 7.4715 | 7.8826 | 7.3377 | 7.7198 | 7.3055 | 7.4911 | 7.375 | 7.3258 |
| 221482_s_at | 10776 | ARPP19 | cAMP-regulated phosphoprotein, 19kDa | 0.1993 | 0.7745 | 0.971 | 7.6044 | 7.1421 | 7.5528 | 7.5244 | 7.3377 | 7.4511 | 7.1703 | 7.114 | 5.5138 | 6.1871 | 7.5446 | 7.2733 |
| 203217_s_at | 8869 | ST3GAL5 | ST3 beta-galactoside alpha-2,3-sialyltransferase 5 | 0.1976 | 0.493 | 0.8692 | 6.8104 | 6.7156 | 6.9123 | 6.7968 | 6.673 | 6.5769 | 6.4014 | 6.8842 | 6.475 | 6.6204 | 6.8082 | 6.5425 |
| 202927_at | 5300 | PIN1 | peptidylprolyl cis/trans isomerase, NIMA-interacting 1 | 0.1968 | 0.9321 | 0.9855 | 11.617 | 11.5626 | 11.4204 | 11.4204 | 11.617 | 11.9863 | 11.6611 | 11.5842 | 11.3348 | 11.5374 | 11.4472 | 11.6611 |
| 205173_x_at | 965 | CD58 | CD58 molecule | 0.1948 | 0.487 | 0.8692 | 9.2323 | 9.4888 | 9.1962 | 9.2995 | 8.6346 | 8.5388 | 8.1305 | 7.9752 | 9.3685 | 8.5141 | 8.3107 | 9.5363 |
| 216321_s_at | 2908 | NR3C1 | nuclear receptor subfamily 3, group C, member 1 (glucocorticoid receptor) | 0.191 | 0.4551 | 0.8366 | 4.7565 | 10.6909 | 4.8949 | 5.1553 | 4.8102 | 10.9929 | 5.2689 | 5.2336 | 10.5393 | 10.9664 | 4.7565 | 5.3047 |
| 204729_s_at | 6804 | STX1A | syntaxin 1A (brain) | 0.19 | 0.499 | 0.873 | 6.2549 | 6.5091 | 6.624 | 6.8082 | 6.8383 | 6.645 | 6.5023 | 6.486 | 6.0427 | 6.4589 | 6.3975 | 6.4307 |
| 210416_s_at | 11200 | CHEK2 | CHK2 checkpoint homolog (S. pombe) | 0.19 | 0.4571 | 0.8387 | 7.8401 | 8.7046 | 7.8745 | 7.9945 | 7.8733 | 8.7079 | 7.3009 | 8.1207 | 7.4511 | 7.8967 | 7.6365 | 7.6781 |
| 213190_at | 91949 | COG7 | component of oligomeric golgi complex 7 | 0.1879 | 0.7864 | 0.9711 | 11.4334 | 11.4763 | 11.3665 | 11.5842 | 11.5626 | 11.6409 | 11.4966 | 11.7662 | 11.7843 | 11.237 | 11.4763 | 11.5626 |
| 211071_s_at | 10962 | MLLT11 | myeloid/lymphoid or mixed-lineage leukemia (trithorax homolog, Drosophila); translocated to, 11 | 0.1866 | 0.8643 | 0.9716 | 7.9512 | 8.2633 | 8.0672 | 8.1475 | 8.1103 | 8.0555 | 8.0384 | 8.3623 | 7.9704 | 7.9783 | 8.1348 | 8.0709 |
| 209015_s_at | 10049 | DNAJB6 | DnaJ (Hsp40) homolog, subfamily B, member 6 | 0.1853 | 0.5629 | 0.8876 | 9.3838 | 8.5113 | 9.7997 | 9.7498 | 9.4599 | 9.4264 | 9.0472 | 7.5546 | 10.8832 | 9.3209 | 9.5117 | 10.1057 |
| 200799_at | 3303 | HSPA1A | heat shock 70kDa protein 1A | 0.1842 | 0.5329 | 0.8789 | 7.3974 | 7.1877 | 6.0877 | 6.0544 | 7.2118 | 6.7968 | 7.0597 | 5.9035 | 7.8326 | 6.1242 | 6.5921 | 7.2836 |
| 201762_s_at | 5721 | PSME2 | proteasome (prosome, macropain) activator subunit 2 (PA28 beta) | 0.1831 | 0.5649 | 0.8876 | 10.7631 | 11.1291 | 10.7025 | 10.8448 | 10.7415 | 11.1141 | 10.7789 | 10.6154 | 10.9719 | 10.8218 | 10.7112 | 10.8036 |
| 203885_at | 23011 | RAB21 | RAB21, member RAS oncogene family | 0.1824 | 0.507 | 0.8748 | 8.5966 | 9.0263 | 7.8967 | 8.4169 | 7.836 | 8.9351 | 8.3248 | 8.1207 | 8.1652 | 7.8155 | 8.9147 | 8.3388 |
| 203154_s_at | 10298 | PAK4 | p21 protein (Cdc42/Rac)-activated kinase 4 | 0.1815 | 0.5289 | 0.8768 | 8.028 | 8.1608 | 8.1089 | 8.115 | 7.9916 | 7.8928 | 7.9882 | 8.0375 | 7.8745 | 7.7351 | 7.9768 | 7.836 |
| 217752_s_at | 55748 | CNDP2 | CNDP dipeptidase 2 (metallopeptidase M20 family) | 0.1809 | 0.982 | 0.9921 | 10.0554 | 9.9689 | 10.1261 | 9.9133 | 9.8546 | 9.8325 | 9.8325 | 7.3055 | 10.1057 | 10.2308 | 10.0688 | 9.5376 |
| 40829_at | 23038 | WDTC1 | WD and tetratricopeptide repeats 1 | 0.1781 | 0.5509 | 0.8876 | 7.5528 | 7.7072 | 7.8826 | 10.6622 | 7.8205 | 7.6211 | 7.8094 | 7.8496 | 7.5528 | 7.594 | 7.3806 | 7.7878 |
| 207334_s_at | 7048 | TGFBR2 | transforming growth factor, beta receptor II (70/80kDa) | 0.1778 | 0.4271 | 0.8207 | 4.5899 | 9.1143 | 4.4407 | 8.9175 | 4.3452 | 4.4649 | 4.9103 | 4.5098 | 4.2311 | 4.2311 | 10.0254 | 4.5367 |
| 221046_s_at | 29083 | GTPBP8 | GTP-binding protein 8 (putative) | 0.1769 | 0.9521 | 0.9921 | 10.0612 | 9.9531 | 10.0188 | 10.1602 | 9.9689 | 9.995 | 9.9373 | 9.5657 | 9.9229 | 10.1261 | 10.1868 | 9.863 |
| 201695_s_at | 4860 | PNP | purine nucleoside phosphorylase | 0.1767 | 0.5988 | 0.8996 | 11.1454 | 11.3234 | 11.3003 | 11.0543 | 10.891 | 11.3234 | 10.9184 | 11.2563 | 10.584 | 11.3348 | 10.9719 | 11.2563 |
| 201380_at | 10491 | CRTAP | cartilage associated protein | 0.1766 | 0.6168 | 0.9084 | 7.6974 | 7.4336 | 7.6365 | 7.4732 | 7.1587 | 7.2967 | 7.4715 | 7.5854 | 6.8978 | 7.0805 | 7.4144 | 7.1215 |
| 204458_at | 23659 | PLA2G15 | phospholipase A2, group XV | 0.1757 | 0.5349 | 0.8807 | 5.9169 | 6.0877 | 6.0389 | 6.1917 | 6.3601 | 5.9915 | 6.0427 | 6.1336 | 5.8537 | 5.9659 | 5.5606 | 6.0275 |
| 218108_at | 55148 | UBR7 | ubiquitin protein ligase E3 component n-recognin 7 (putative) | 0.1693 | 0.475 | 0.8556 | 10.6154 | 10.9533 | 9.9901 | 9.8495 | 10.7277 | 9.6609 | 9.1996 | 10.9381 | 9.3515 | 10.5886 | 9.8129 | 10.6622 |
| 210153_s_at | 4200 | ME2 | malic enzyme 2, NAD(+)-dependent, mitochondrial | 0.1687 | 0.525 | 0.8768 | 8.0728 | 7.7986 | 8.6025 | 8.261 | 8.5506 | 7.7617 | 8.2423 | 7.5312 | 7.5805 | 7.6666 | 8.4338 | 7.7198 |
| 208050_s_at | 835 | CASP2 | caspase 2, apoptosis-related cysteine peptidase | 0.1669 | 0.5709 | 0.8876 | 6.9416 | 11.8756 | 7.1088 | 7.2032 | 6.8675 | 6.4929 | 6.7107 | 6.3947 | 6.3919 | 6.5942 | 6.9591 | 6.3433 |
| 209341_s_at | 3551 | IKBKB | inhibitor of kappa light polypeptide gene enhancer in B-cells, kinase beta | 0.1648 | 0.5289 | 0.8768 | 6.7447 | 6.6781 | 6.355 | 6.4929 | 6.4268 | 5.9592 | 6.051 | 6.0701 | 6.607 | 6.3601 | 6.3433 | 6.0544 |
| 211318_s_at | 8480 | RAE1 | RAE1 RNA export 1 homolog (S. pombe) | 0.16 | 0.6128 | 0.9066 | 9.5067 | 9.4345 | 9.753 | 9.7561 | 9.7015 | 9.6648 | 9.135 | 9.4345 | 9.0983 | 9.2686 | 9.5657 | 9.5414 |
| 203727_at | 6499 | SKIV2L | superkiller viralicidic activity 2-like (S. cerevisiae) | 0.1595 | 0.5629 | 0.8876 | 7.0275 | 7.1703 | 7.375 | 7.2118 | 7.1802 | 7.266 | 6.5746 | 7.4732 | 7.1088 | 7.3639 | 6.8703 | 6.9712 |
| 201178_at | 25793 | FBXO7 | F-box protein 7 | 0.1587 | 0.6387 | 0.9161 | 6.9803 | 6.4515 | 7.5288 | 7.375 | 6.7423 | 7.1397 | 7.1877 | 6.7968 | 7.028 | 7.2247 | 6.8551 | 6.6032 |
| 202518_at | 9275 | BCL7B | B-cell CLL/lymphoma 7B | 0.1584 | 0.6926 | 0.9421 | 6.282 | 5.9102 | 6.1984 | 6.0683 | 5.9211 | 5.8749 | 6.1444 | 6.0544 | 5.4947 | 6.0683 | 5.9102 | 5.938 |
| 218421_at | 64781 | CERK | ceramide kinase | 0.1576 | 0.5489 | 0.8873 | 7.5528 | 7.3015 | 7.499 | 7.6044 | 7.4044 | 7.3277 | 7.3848 | 7.2032 | 7.9078 | 7.6044 | 7.3848 | 7.5013 |
| 208905_at | 54205 | CYCS | cytochrome c, somatic | 0.1572 | 0.6986 | 0.9451 | 10.5393 | 10.6154 | 10.7518 | 10.7277 | 10.7068 | 10.9719 | 10.584 | 10.7811 | 10.5146 | 10.8754 | 5.5138 | 11.0292 |
| 205393_s_at | 1111 | CHEK1 | CHK1 checkpoint homolog (S. pombe) | 0.1563 | 0.5589 | 0.8876 | 9.3258 | 9.3099 | 9.41 | 10.1697 | 9.5094 | 9.8973 | 10.1057 | 9.1623 | 9.1196 | 9.3908 | 9.3333 | 10.0005 |
| 201825_s_at | 51097 | SCCPDH | saccharopine dehydrogenase (putative) | 0.1525 | 0.6068 | 0.9005 | 8.7293 | 8.5506 | 9.2467 | 8.5894 | 8.5852 | 8.7683 | 8.4101 | 8.6433 | 8.5113 | 8.5194 | 8.4399 | 8.1439 |
| 214061_at | 93594 | WDR67 | WD repeat domain 67 | 0.1521 | 0.5709 | 0.8876 | 9.5363 | 8.9058 | 8.6688 | 9.6648 | 9.5537 | 9.764 | 9.8495 | 9.8129 | 9.6628 | 8.8613 | 8.7012 | 8.9841 |
| 205581_s_at | 4846 | NOS3 | nitric oxide synthase 3 (endothelial cell) | 0.1516 | 0.5629 | 0.8876 | 5.3085 | 5.0412 | 5.1321 | 4.8576 | 5.1494 | 5.5664 | 4.7754 | 5.0412 | 5.0204 | 5.5278 | 5.2393 | 4.7409 |
| 214274_s_at | 30 | ACAA1 | acetyl-CoA acyltransferase 1 | 0.1512 | 0.5449 | 0.8853 | 5.8324 | 9.2467 | 5.6633 | 5.5863 | 5.8375 | 6.6562 | 5.6276 | 5.5182 | 5.6507 | 6.9296 | 5.4862 | 5.831 |
| 215000_s_at | 9637 | FEZ2 | fasciculation and elongation protein zeta 2 (zygin II) | 0.15 | 0.6327 | 0.9127 | 6.0718 | 6.0389 | 4.7983 | 5.9592 | 4.8576 | 5.7423 | 5.0655 | 5.8589 | 5.8983 | 4.7839 | 5.9114 | 4.5716 |
| 202756_s_at | 2817 | GPC1 | glypican 1 | 0.1493 | 0.5709 | 0.8876 | 5.7979 | 6.0253 | 5.641 | 5.8715 | 5.7928 | 5.9102 | 5.8338 | 5.1494 | 6.3524 | 5.6226 | 6.1406 | 5.4992 |
| 218742_at | 64428 | NARFL | nuclear prelamin A recognition factor-like | 0.1481 | 0.5948 | 0.8991 | 6.9073 | 6.8479 | 13.6371 | 14.3602 | 6.9123 | 7.5244 | 13.6371 | 7.105 | 8.8397 | 8.2172 | 7.7351 | 7.3914 |
| 204466_s_at | 6622 | SNCA | synuclein, alpha (non A4 component of amyloid precursor) | 0.148 | 0.7425 | 0.9683 | 9.3838 | 8.9883 | 9.1768 | 9.0472 | 9.5414 | 9.3515 | 9.5094 | 9.41 | 9.3908 | 9.1315 | 9.2127 | 9.2995 |
| 218679_s_at | 51160 | VPS28 | vacuolar protein sorting 28 homolog (S. cerevisiae) | 0.1474 | 0.6148 | 0.9069 | 6.673 | 5.607 | 6.9712 | 6.8236 | 5.8537 | 6.6562 | 7.2559 | 7.3268 | 5.6368 | 6.607 | 5.4536 | 6.7967 |
| 206170_at | 154 | ADRB2 | adrenergic, beta-2-, receptor, surface | 0.1471 | 0.7186 | 0.9548 | 9.2815 | 4.6702 | 4.7052 | 4.8822 | 5.0412 | 4.6702 | 4.6246 | 4.8362 | 4.685 | 4.6702 | 4.8822 | 4.6702 |
| 203336_s_at | 9270 | ITGB1BP1 | integrin beta 1 binding protein 1 | 0.1454 | 0.9561 | 0.9921 | 8.8894 | 8.7046 | 9.123 | 8.9058 | 8.9897 | 9.1623 | 8.3788 | 8.8561 | 8.9574 | 8.7574 | 8.9869 | 9.0656 |
| 204042_at | 10810 | WASF3 | WAS protein family, member 3 | 0.1449 | 0.7146 | 0.9508 | 7.1877 | 7.0824 | 6.9335 | 5.2837 | 7.2641 | 7.1331 | 5.0985 | 7.3841 | 6.1608 | 7.0474 | 6.7472 | 6.8157 |
| 204093_at | 902 | CCNH | cyclin H | 0.1439 | 0.5988 | 0.8996 | 9.0141 | 8.9398 | 8.9398 | 8.8263 | 8.4339 | 8.5324 | 8.3857 | 8.6808 | 8.6663 | 8.5749 | 8.7781 | 8.6391 |
| 203127_s_at | 9517 | SPTLC2 | serine palmitoyltransferase, long chain base subunit 2 | 0.1438 | 0.6687 | 0.9329 | 4.8161 | 5.1258 | 5.2689 | 5.1258 | 5.0204 | 5.0412 | 4.5716 | 5.1321 | 4.9166 | 5.1258 | 4.8949 | 4.9166 |
| 204605_at | 10668 | CGRRF1 | cell growth regulator with ring finger domain 1 | 0.1426 | 0.505 | 0.8748 | 9.4264 | 8.6284 | 8.9027 | 9.5537 | 9.8325 | 8.748 | 8.5715 | 8.7822 | 8.5356 | 9.5376 | 8.8096 | 8.9671 |
| 217950_at | 51070 | NOSIP | nitric oxide synthase interacting protein | 0.1425 | 0.5409 | 0.8817 | 6.3388 | 7.1088 | 6.0718 | 7.0176 | 6.0231 | 5.9795 | 6.0818 | 6.2034 | 5.6606 | 6.0544 | 6.519 | 5.7748 |
| 216836_s_at | 2064 | ERBB2 | v-erb-b2 erythroblastic leukemia viral oncogene homolog 2, neuro/glioblastoma derived oncogene homolog (avian) | 0.1406 | 0.5988 | 0.8996 | 9.2127 | 8.7945 | 9.1036 | 8.2084 | 8.1263 | 8.205 | 7.8326 | 8.9748 | 8.0321 | 9.3022 | 8.2547 | 8.7913 |
| 202880_s_at | 9267 | CYTH1 | cytohesin 1 | 0.139 | 0.5269 | 0.8768 | 5.1494 | 5.1494 | 5.4123 | 4.9783 | 5.5138 | 5.0412 | 4.8949 | 5.2837 | 5.0618 | 5.1612 | 5.001 | 5.0515 |
| 201829_at | 10276 | NET1 | neuroepithelial cell transforming 1 | 0.1372 | 0.8084 | 0.9716 | 9.3515 | 9.1555 | 9.3908 | 9.0859 | 9.1386 | 8.2735 | 9.0411 | 7.8967 | 8.9869 | 8.9681 | 9.1623 | 9.0193 |
| 201351_s_at | 10730 | YME1L1 | YME1-like 1 (S. cerevisiae) | 0.1371 | 0.9042 | 0.9825 | 7.4558 | 7.3974 | 7.2524 | 6.9712 | 7.7231 | 7.4055 | 7.7733 | 7.4055 | 6.7033 | 7.114 | 7.3055 | 7.3695 |
| 203787_at | 23635 | SSBP2 | single-stranded DNA binding protein 2 | 0.1369 | 0.6068 | 0.9005 | 6.3105 | 6.8406 | 6.3138 | 6.0231 | 6.9922 | 6.8574 | 6.2326 | 5.5037 | 6.8938 | 5.8783 | 6.7968 | 6.6513 |
| 209253_at | 10174 | SORBS3 | sorbin and SH3 domain containing 3 | 0.1367 | 0.6188 | 0.9086 | 5.6977 | 5.5982 | 6.4541 | 5.8537 | 5.8715 | 8.9897 | 5.4641 | 7.8496 | 8.1348 | 5.7514 | 5.5982 | 5.452 |
| 207515_s_at | 9533 | POLR1C | polymerase (RNA) I polypeptide C, 30kDa | 0.1365 | 0.9641 | 0.9921 | 9.0141 | 8.801 | 8.9158 | 8.9158 | 8.8182 | 8.9058 | 8.8195 | 8.8894 | 9.2876 | 9.2516 | 9.2198 | 8.6171 |
| 217906_at | 23588 | KLHDC2 | kelch domain containing 2 | 0.1351 | 0.6607 | 0.9284 | 10.2308 | 10.7025 | 10.518 | 10.4396 | 10.6909 | 11.1962 | 11.3665 | 10.3682 | 10.4772 | 10.3106 | 10.4841 | 10.7025 |
| 209710_at | 2624 | GATA2 | GATA binding protein 2 | 0.1348 | 0.6048 | 0.9003 | 7.2836 | 6.9803 | 7.0859 | 6.7808 | 6.687 | 9.1906 | 6.4014 | 8.0828 | 7.0042 | 6.6348 | 8.1878 | 6.4567 |
| 205055_at | 3682 | ITGAE | integrin, alpha E (antigen CD103, human mucosal lymphocyte antigen 1; alpha polypeptide) | 0.1344 | 0.6826 | 0.939 | 10.518 | 10.3258 | 10.3074 | 10.2986 | 10.3308 | 10.6716 | 10.4133 | 10.3359 | 10.1602 | 10.4689 | 10.7789 | 10.8365 |
| 206662_at | 2745 | GLRX | glutaredoxin (thioltransferase) | 0.1323 | 0.9222 | 0.9845 | 8.5852 | 8.3754 | 8.2633 | 8.1089 | 8.1918 | 8.3248 | 7.9916 | 8.231 | 5.0985 | 8.2354 | 8.524 | 8.4555 |
| 201811_x_at | 9467 | SH3BP5 | SH3-domain binding protein 5 (BTK-associated) | 0.1313 | 0.6148 | 0.9069 | 8.4643 | 8.5869 | 8.8935 | 8.7457 | 8.524 | 8.3248 | 8.5996 | 8.7781 | 8.5852 | 8.5869 | 8.3754 | 8.4339 |
| 202417_at | 9817 | KEAP1 | kelch-like ECH-associated protein 1 | 0.1312 | 0.5908 | 0.8986 | 9.5686 | 9.5117 | 9.8129 | 9.5117 | 9.5657 | 9.6875 | 9.4254 | 9.7957 | 9.2815 | 9.3685 | 9.5363 | 9.3022 |
| 212833_at | 91137 | SLC25A46 | solute carrier family 25, member 46 | 0.1307 | 0.8204 | 0.9716 | 9.101 | 9.2274 | 9.0193 | 9.1036 | 9.0694 | 8.4338 | 8.446 | 8.7683 | 8.633 | 9.0656 | 9.1831 | 8.8112 |
| 201628_s_at | 10670 | RRAGA | Ras-related GTP binding A | 0.1306 | 0.6467 | 0.9193 | 7.8512 | 7.6168 | 7.7509 | 7.6723 | 7.7834 | 8.4579 | 7.5288 | 7.8285 | 7.6761 | 7.4873 | 7.7535 | 7.4055 |
| 209572_s_at | 8726 | EED | embryonic ectoderm development | 0.1285 | 0.6427 | 0.9163 | 8.8377 | 9.0765 | 8.996 | 8.9622 | 8.9841 | 8.9398 | 8.5749 | 9.1868 | 8.4049 | 8.8311 | 8.6906 | 8.4815 |
| 201548_s_at | 10765 | KDM5B | lysine (K)-specific demethylase 5B | 0.1282 | 0.6048 | 0.9003 | 5.8148 | 5.9831 | 5.9693 | 6.3601 | 5.8783 | 5.4195 | 5.6868 | 5.0618 | 5.7104 | 5.641 | 5.3457 | 5.5182 |
| 202187_s_at | 5525 | PPP2R5A | protein phosphatase 2, regulatory subunit B', alpha | 0.128 | 0.6527 | 0.9211 | 9.1578 | 9.3308 | 9.3308 | 9.0733 | 9.1601 | 9.0383 | 9.0574 | 9.3433 | 8.8311 | 8.7683 | 8.9681 | 9.2686 |
| 202006_at | 5782 | PTPN12 | protein tyrosine phosphatase, non-receptor type 12 | 0.1279 | 0.6287 | 0.9127 | 8.5356 | 8.5715 | 8.0043 | 8.5831 | 8.581 | 8.094 | 8.2067 | 8.5506 | 8.2354 | 8.2704 | 8.0446 | 7.8285 |
| 202324_s_at | 64746 | ACBD3 | acyl-CoA binding domain containing 3 | 0.127 | 0.5848 | 0.8951 | 7.7733 | 7.9704 | 7.3639 | 8.1263 | 8.0828 | 7.249 | 7.3021 | 7.9071 | 7.49 | 7.1768 | 7.1877 | 7.6429 |
| 222125_s_at | 54681 | P4HTM | prolyl 4-hydroxylase, transmembrane (endoplasmic reticulum) | 0.1261 | 0.6926 | 0.9421 | 9.2452 | 4.6437 | 4.9824 | 4.8042 | 5.0692 | 5.9277 | 4.8822 | 5.0786 | 4.5509 | 5.5698 | 5.1707 | 4.8949 |
| 218375_at | 53343 | NUDT9 | nudix (nucleoside diphosphate linked moiety X)-type motif 9 | 0.1238 | 0.517 | 0.8768 | 8.3723 | 8.3197 | 8.4101 | 8.2704 | 8.1874 | 8.4172 | 7.9185 | 8.0008 | 7.8826 | 8.0781 | 8.0938 | 7.7231 |
| 219384_s_at | 23536 | ADAT1 | adenosine deaminase, tRNA-specific 1 | 0.1238 | 0.6687 | 0.9329 | 6.3524 | 6.3308 | 5.7559 | 5.7278 | 6.051 | 6.607 | 6.282 | 5.8229 | 6.7033 | 6.2473 | 6.282 | 6.1406 |
| 202011_at | 7082 | TJP1 | tight junction protein 1 (zona occludens 1) | 0.1221 | 0.5848 | 0.8951 | 6.7205 | 7.0649 | 6.5894 | 6.6348 | 7.0176 | 7.4732 | 6.7539 | 6.6951 | 6.9488 | 7.2247 | 6.8479 | 6.6276 |
| 204484_at | 5287 | PIK3C2B | phosphoinositide-3-kinase, class 2, beta polypeptide | 0.1207 | 0.7126 | 0.9495 | 4.9477 | 5.1384 | 6.0466 | 5.1384 | 5.0322 | 5.8148 | 4.7487 | 4.7487 | 4.7565 | 4.8822 | 5.726 | 5.8103 |
| 218826_at | 54733 | SLC35F2 | solute carrier family 35, member F2 | 0.1169 | 0.9242 | 0.9845 | 7.8706 | 7.5854 | 7.9232 | 7.7382 | 7.8733 | 7.6044 | 7.4873 | 7.9566 | 6.5091 | 7.8245 | 7.657 | 7.6781 |
| 201565_s_at | 3398 | ID2 | inhibitor of DNA binding 2, dominant negative helix-loop-helix protein | 0.1155 | 0.8443 | 0.9716 | 9.1623 | 9.2437 | 7.0042 | 9.0023 | 9.2437 | 9.266 | 8.4338 | 8.8209 | 6.9462 | 8.732 | 9.2936 | 8.7079 |
| 203358_s_at | 2146 | EZH2 | enhancer of zeste homolog 2 (Drosophila) | 0.1154 | 0.8263 | 0.9716 | 10.1782 | 10.1782 | 10.2552 | 10.2223 | 10.1261 | 10.0612 | 10.3106 | 10.3781 | 9.6213 | 10.1782 | 10.1184 | 10.1416 |
| 201292_at | 7153 | TOP2A | topoisomerase (DNA) II alpha 170kDa | 0.1151 | 0.6188 | 0.9086 | 12.3455 | 12.2771 | 12.465 | 12.3753 | 12.8313 | 12.3234 | 12.3455 | 12.7071 | 12.3234 | 12.2342 | 12.4511 | 12.4511 |
| 204014_at | 1846 | DUSP4 | dual specificity phosphatase 4 | 0.1143 | 0.6547 | 0.9226 | 6.2911 | 6.6204 | 6.6781 | 6.794 | 6.6562 | 6.7922 | 6.3138 | 6.0701 | 6.2789 | 6.3758 | 6.3575 | 6.1187 |
| 200766_at | 1509 | CTSD | cathepsin D | 0.1131 | 0.6866 | 0.9405 | 8.6368 | 8.4166 | 7.9623 | 7.8285 | 8.0157 | 8.094 | 7.4188 | 8.2388 | 8.1021 | 8.1103 | 7.7291 | 8.2423 |
| 209213_at | 873 | CBR1 | carbonyl reductase 1 | 0.1128 | 0.7026 | 0.9465 | 8.3158 | 7.4873 | 8.2332 | 8.5966 | 7.7165 | 8.1874 | 7.5652 | 7.6527 | 8.1874 | 8.1263 | 7.5692 | 7.5612 |
| 209367_at | 6813 | STXBP2 | syntaxin binding protein 2 | 0.1115 | 0.6786 | 0.9361 | 6.1533 | 5.7669 | 6.1608 | 5.9873 | 7.1292 | 6.051 | 5.5922 | 5.9559 | 6.8126 | 5.7319 | 5.9559 | 7.2601 |
| 205376_at | 8821 | INPP4B | inositol polyphosphate-4-phosphatase, type II, 105kDa | 0.1097 | 0.8224 | 0.9716 | 10.1385 | 4.5225 | 4.4407 | 4.2444 | 4.2444 | 4.2682 | 4.4812 | 4.3929 | 4.7131 | 4.151 | 4.601 | 4.6373 |
| 201466_s_at | 3725 | JUN | jun proto-oncogene | 0.1054 | 0.7485 | 0.9685 | 10.1416 | 10.0321 | 9.8495 | 10.4772 | 10.5327 | 10.4689 | 10.3074 | 10.3469 | 9.41 | 10.0593 | 10.6579 | 9.9331 |
| 204937_s_at | 10782 | ZNF274 | zinc finger protein 274 | 0.1049 | 0.7425 | 0.9683 | 9.6648 | 10.2496 | 10.3927 | 9.995 | 9.6648 | 9.6827 | 10.1385 | 9.5537 | 9.8546 | 9.955 | 9.8376 | 9.5094 |
| 203341_at | 10153 | CEBPZ | CCAAT/enhancer binding protein (C/EBP), zeta | 0.1019 | 0.7006 | 0.9451 | 9.8129 | 10.2223 | 9.8973 | 9.9229 | 9.706 | 8.9432 | 10.6154 | 9.0581 | 9.6092 | 9.8849 | 8.8492 | 10.0612 |
| 200697_at | 3098 | HK1 | hexokinase 1 | 0.1016 | 0.7325 | 0.9668 | 5.2837 | 4.9783 | 5.4536 | 5.3503 | 5.432 | 5.001 | 5.2488 | 5.7708 | 4.6702 | 5.0322 | 4.7754 | 5.294 |
| 203884_s_at | 22841 | RAB11FIP2 | RAB11 family interacting protein 2 (class I) | 0.1002 | 0.7006 | 0.9451 | 6.8978 | 7.1202 | 6.8104 | 8.4399 | 6.73 | 6.9618 | 6.4832 | 6.6204 | 7.7535 | 6.6599 | 6.8316 | 6.6183 |
| 201502_s_at | 4792 | NFKBIA | nuclear factor of kappa light polypeptide gene enhancer in B-cells inhibitor, alpha | 0.09895 | 0.7625 | 0.9685 | 7.044 | 6.5894 | 7.1012 | 7.0333 | 6.6204 | 6.2209 | 6.5676 | 6.7922 | 6.0683 | 6.6502 | 6.496 | 6.6348 |
| 204039_at | 1050 | CEBPA | CCAAT/enhancer binding protein (C/EBP), alpha | 0.09852 | 0.5948 | 0.8991 | 6.2758 | 6.0527 | 6.486 | 6.289 | 5.938 | 5.4947 | 5.7104 | 5.8715 | 5.8338 | 7.2343 | 6.0004 | 5.6175 |
| 201827_at | 6603 | SMARCD2 | SWI/SNF related, matrix associated, actin dependent regulator of chromatin, subfamily d, member 2 | 0.09683 | 0.6667 | 0.9329 | 7.8144 | 7.8155 | 8.1808 | 7.8634 | 8.1565 | 7.5564 | 7.7856 | 7.6723 | 7.5692 | 7.8512 | 7.9327 | 7.5131 |
| 204475_at | 4312 | MMP1 | matrix metallopeptidase 1 (interstitial collagenase) | 0.09646 | 0.7445 | 0.9683 | 6.2691 | 6.3433 | 6.4929 | 6.7107 | 6.5676 | 10.2223 | 6.5375 | 9.0411 | 7.0977 | 7.4044 | 6.7783 | 6.5676 |
| 202812_at | 2548 | GAA | glucosidase, alpha; acid | 0.09581 | 0.6946 | 0.9422 | 4.4486 | 4.3452 | 4.6702 | 4.685 | 4.4407 | 3.9141 | 4.0252 | 4.8613 | 4.4147 | 4.4147 | 4.2996 | 4.4147 |
| 201536_at | 1845 | DUSP3 | dual specificity phosphatase 3 | 0.09514 | 0.7006 | 0.9451 | 8.1688 | 7.9078 | 8.7527 | 9.1996 | 8.3723 | 8.1263 | 8.0672 | 8.5472 | 8.9158 | 8.2886 | 9.3685 | 7.7011 |
| 218597_s_at | 55847 | CISD1 | CDGSH iron sulfur domain 1 | 0.09373 | 0.7705 | 0.9698 | 11.4966 | 10.7811 | 11.0907 | 11.9863 | 11.1291 | 11.1378 | 11.1661 | 10.9184 | 10.7631 | 11.353 | 10.9664 | 11.3911 |
| 218547_at | 79947 | DHDDS | dehydrodolichyl diphosphate synthase | 0.08827 | 0.8463 | 0.9716 | 6.7968 | 6.9803 | 6.5355 | 6.7968 | 12.1565 | 6.8406 | 7.1835 | 6.8157 | 6.7033 | 6.673 | 6.6619 | 6.6513 |
| 203409_at | 1643 | DDB2 | damage-specific DNA binding protein 2, 48kDa | 0.08554 | 0.7844 | 0.9711 | 5.7669 | 5.9559 | 6.4268 | 5.9693 | 6.2442 | 5.6326 | 5.8715 | 5.7708 | 5.8537 | 6.0155 | 6.1871 | 5.9114 |
| 205450_at | 5255 | PHKA1 | phosphorylase kinase, alpha 1 (muscle) | 0.08548 | 0.6806 | 0.9376 | 7.657 | 7.2396 | 6.8458 | 6.8406 | 7.6044 | 6.5676 | 6.7968 | 7.5546 | 6.2691 | 6.54 | 6.5054 | 7.4688 |
| 202623_at | 55837 | EAPP | E2F-associated phosphoprotein | 0.08459 | 0.7665 | 0.9685 | 7.2343 | 7.1474 | 7.2836 | 7.1012 | 7.0252 | 6.9522 | 6.4929 | 6.6312 | 6.4053 | 6.9123 | 7.0333 | 6.9803 |
| 203192_at | 10058 | ABCB6 | ATP-binding cassette, sub-family B (MDR/TAP), member 6 | 0.08074 | 0.6567 | 0.9241 | 6.1124 | 8.1986 | 6.8479 | 6.5301 | 6.4567 | 6.4376 | 6.7289 | 6.937 | 6.2268 | 6.5301 | 6.7033 | 7.3549 |
| 214259_s_at | 8574 | AKR7A2 | aldo-keto reductase family 7, member A2 (aflatoxin aldehyde reductase) | 0.0806 | 0.8743 | 0.9738 | 10.491 | 10.518 | 10.7927 | 10.4133 | 10.6579 | 10.518 | 10.3469 | 10.6326 | 10.2496 | 10.8365 | 10.3927 | 10.5886 |
| 203418_at | 890 | CCNA2 | cyclin A2 | 0.07944 | 0.7465 | 0.9683 | 10.8218 | 10.8754 | 10.9929 | 10.7025 | 10.7927 | 10.7415 | 10.5646 | 10.8682 | 10.4689 | 10.8036 | 10.7631 | 10.6716 |
| 218064_s_at | 26993 | AKAP8L | A kinase (PRKA) anchor protein 8-like | 0.07937 | 0.7565 | 0.9685 | 6.8104 | 6.9803 | 6.8938 | 7.0649 | 7.6629 | 8.0781 | 7.5198 | 6.5931 | 7.2265 | 7.5385 | 6.645 | 6.3947 |
| 202368_s_at | 9697 | TRAM2 | translocation associated membrane protein 2 | 0.07699 | 0.8283 | 0.9716 | 4.8613 | 5.223 | 4.9937 | 4.9103 | 4.9353 | 4.612 | 4.7052 | 4.9864 | 5.3293 | 4.9477 | 5.1612 | 5.1384 |
| 202261_at | 6944 | VPS72 | vacuolar protein sorting 72 homolog (S. cerevisiae) | 0.07641 | 0.7645 | 0.9685 | 7.5198 | 6.8458 | 6.7205 | 6.6399 | 6.7033 | 6.3105 | 6.6638 | 6.6951 | 7.0176 | 6.9462 | 6.7904 | 7.0745 |
| 206846_s_at | 10013 | HDAC6 | histone deacetylase 6 | 0.0763 | 0.7585 | 0.9685 | 7.7856 | 7.9916 | 8.3088 | 8.1986 | 7.7834 | 7.9852 | 7.7986 | 8.1348 | 7.6545 | 8.1375 | 7.6085 | 7.8512 |
| 204985_s_at | 79090 | TRAPPC6A | trafficking protein particle complex 6A | 0.07536 | 0.8523 | 0.9716 | 7.7198 | 7.6416 | 7.9478 | 7.8155 | 7.7165 | 8.0104 | 7.5506 | 7.7914 | 7.848 | 7.848 | 7.8706 | 7.7351 |
| 204143_s_at | 55556 | ENOSF1 | enolase superfamily member 1 | 0.07513 | 0.8683 | 0.9716 | 4.1889 | 3.9141 | 5.9329 | 3.9983 | 3.9983 | 3.8487 | 4.1172 | 4.2444 | 3.8487 | 4.1889 | 4.1889 | 3.8487 |
| 212665_at | 25976 | TIPARP | TCDD-inducible poly(ADP-ribose) polymerase | 0.07299 | 0.7764 | 0.9711 | 6.7447 | 7.375 | 7.2247 | 7.4336 | 6.8383 | 6.7107 | 6.4669 | 7.2881 | 7.1587 | 6.9335 | 7.1202 | 6.8842 |
| 215116_s_at | 1759 | DNM1 | dynamin 1 | 0.07253 | 0.7844 | 0.9711 | 6.2268 | 6.1608 | 6.5676 | 6.5474 | 6.4832 | 6.4053 | 6.1877 | 6.6599 | 6.3187 | 6.2057 | 6.0701 | 6.0683 |
| 121_at | 7849 | PAX8 | paired box 8 | 0.07147 | 0.7625 | 0.9685 | 5.4862 | 5.223 | 4.7754 | 5.2393 | 5.0985 | 5.2278 | 5.2837 | 4.9477 | 5.5378 | 5.3131 | 5.5606 | 4.612 |
| 219742_at | 80758 | PRR7 | proline rich 7 (synaptic) | 0.06914 | 0.7725 | 0.971 | 7.3519 | 7.5198 | 6.5968 | 6.4361 | 7.4144 | 6.5921 | 6.0275 | 8.0008 | 6.2057 | 6.3919 | 6.5769 | 7.2304 |
| 209860_s_at | 310 | ANXA7 | annexin A7 | 0.06881 | 0.7605 | 0.9685 | 9.0859 | 9.1996 | 9.2274 | 9.2516 | 9.0983 | 8.9514 | 8.5869 | 8.9897 | 8.6497 | 8.9158 | 8.9454 | 8.8311 |
| 202630_at | 10513 | APPBP2 | amyloid beta precursor protein (cytoplasmic tail) binding protein 2 | 0.06858 | 0.8024 | 0.9716 | 6.56 | 6.0466 | 6.1984 | 6.3575 | 6.3524 | 5.9693 | 6.2011 | 6.6032 | 6.2624 | 6.1957 | 6.2411 | 6.1608 |
| 203458_at | 6697 | SPR | sepiapterin reductase (7,8-dihydrobiopterin:NADP+ oxidoreductase) | 0.06634 | 0.8623 | 0.9716 | 8.6284 | 8.5665 | 9.4483 | 8.5096 | 8.6833 | 9.1996 | 8.3788 | 9.4092 | 9.0765 | 8.446 | 9.0694 | 9.2633 |
| 201200_at | 8804 | CREG1 | cellular repressor of E1A-stimulated genes 1 | 0.06615 | 0.7884 | 0.9711 | 10.6326 | 9.647 | 10.6225 | 9.4254 | 11.0543 | 9.764 | 9.8445 | 9.6875 | 10.6579 | 9.8495 | 9.8445 | 10.2223 |
| 202778_s_at | 7750 | ZMYM2 | zinc finger, MYM-type 2 | 0.06505 | 0.6347 | 0.9129 | 7.1012 | 7.5357 | 7.2733 | 7.114 | 7.1228 | 6.6638 | 6.6502 | 7.2247 | 6.673 | 6.8551 | 6.836 | 6.6399 |
| 203221_at | 7088 | TLE1 | transducin-like enhancer of split 1 (E(sp1) homolog, Drosophila) | 0.06303 | 0.8383 | 0.9716 | 5.9035 | 10.2959 | 10.5113 | 10.3106 | 9.9373 | 10.1385 | 5.9169 | 10.0554 | 9.7561 | 9.8063 | 9.9133 | 9.8849 |
| 211518_s_at | 652 | BMP4 | bone morphogenetic protein 4 | 0.06261 | 0.7944 | 0.9716 | 6.0073 | 6.0329 | 5.7559 | 6.051 | 9.1315 | 6.2057 | 7.2383 | 6.1214 | 6.8082 | 6.3524 | 6.4589 | 6.5942 |
| 200045_at | 23 | ABCF1 | ATP-binding cassette, sub-family F (GCN20), member 1 | 0.06209 | 0.8643 | 0.9716 | 9.5496 | 10.4012 | 9.8234 | 9.776 | 10.6038 | 10.7025 | 10.262 | 10.1385 | 10.0386 | 9.8445 | 10.6038 | 10.2832 |
| 214062_x_at | 4793 | NFKBIB | nuclear factor of kappa light polypeptide gene enhancer in B-cells inhibitor, beta | 0.0618 | 0.7944 | 0.9716 | 6.0466 | 5.7748 | 5.9329 | 5.8783 | 6.1727 | 6.3575 | 5.9488 | 5.7928 | 5.607 | 5.8715 | 5.8474 | 5.8411 |
| 201432_at | 847 | CAT | catalase | 0.06062 | 0.8323 | 0.9716 | 7.8144 | 6.937 | 7.583 | 7.6257 | 7.436 | 6.519 | 7.8144 | 6.5245 | 7.8967 | 8.0906 | 7.9882 | 6.8938 |
| 201778_s_at | 9813 | KIAA0494 | KIAA0494 | 0.05995 | 0.8084 | 0.9716 | 8.2735 | 8.028 | 8.3107 | 8.1402 | 8.2633 | 8.8935 | 8.0728 | 8.4049 | 9.4092 | 8.7157 | 8.3656 | 7.7509 |
| 202916_s_at | 9917 | FAM20B | family with sequence similarity 20, member B | 0.05619 | 0.8244 | 0.9716 | 7.4889 | 7.5761 | 7.5337 | 7.6126 | 7.5805 | 7.9131 | 8.2067 | 8.1475 | 7.4188 | 7.3914 | 7.5805 | 7.7968 |
| 212644_s_at | 93487 | MAPK1IP1L | mitogen-activated protein kinase 1 interacting protein 1-like | 0.05468 | 0.8623 | 0.9716 | 8.4338 | 9.1265 | 8.9622 | 9.2127 | 8.4602 | 8.6338 | 8.5113 | 8.4602 | 8.0043 | 8.9027 | 8.8397 | 8.5919 |
| 203381_s_at | 348 | APOE | apolipoprotein E | 0.05454 | 0.8583 | 0.9716 | 4.2444 | 4.4486 | 6.9803 | 4.5225 | 6.9298 | 5.473 | 7.7811 | 4.7754 | 5.0884 | 6.6276 | 4.4812 | 4.5899 |
| 203306_s_at | 10559 | SLC35A1 | solute carrier family 35 (CMP-sialic acid transporter), member A1 | 0.05399 | 0.9062 | 0.9825 | 8.9027 | 9.0472 | 8.8587 | 8.8096 | 8.6729 | 8.557 | 8.4501 | 8.801 | 8.6497 | 8.8561 | 8.9351 | 8.7781 |
| 200752_s_at | 823 | CAPN1 | calpain 1, (mu/I) large subunit | 0.05138 | 0.8882 | 0.9782 | 4.6702 | 4.4812 | 4.5225 | 4.685 | 4.4147 | 4.3929 | 4.5509 | 4.5367 | 4.3164 | 4.7565 | 4.3669 | 4.151 |
| 202722_s_at | 2673 | GFPT1 | glutamine--fructose-6-phosphate transaminase 1 | 0.05072 | 0.8064 | 0.9716 | 5.9488 | 5.9102 | 5.9035 | 5.452 | 6.0359 | 6.1289 | 5.6458 | 6.0253 | 5.5378 | 5.9009 | 5.9277 | 5.9277 |
| 202647_s_at | 4893 | NRAS | neuroblastoma RAS viral (v-ras) oncogene homolog | 0.04845 | 0.8483 | 0.9716 | 8.9398 | 8.5217 | 8.8128 | 8.7945 | 8.7574 | 8.6476 | 8.5466 | 8.6346 | 8.8561 | 8.5919 | 8.8561 | 8.5096 |
| 203582_s_at | 5867 | RAB4A | RAB4A, member RAS oncogene family | 0.04742 | 0.8283 | 0.9716 | 8.205 | 8.8263 | 9.0581 | 8.3107 | 8.8209 | 8.0781 | 7.6781 | 7.8401 | 8.4815 | 8.3443 | 8.8128 | 8.6729 |
| 207419_s_at | 5880 | RAC2 | ras-related C3 botulinum toxin substrate 2 (rho family, small GTP binding protein Rac2) | 0.04737 | 0.8004 | 0.9716 | 4.8042 | 4.8042 | 3.955 | 4.3308 | 4.3929 | 5.7242 | 4.4147 | 4.4407 | 4.5098 | 5.0739 | 4.6373 | 4.7754 |
| 201611_s_at | 23463 | ICMT | isoprenylcysteine carboxyl methyltransferase | 0.04568 | 0.8463 | 0.9716 | 9.9901 | 9.5363 | 10.0688 | 10.3074 | 9.4414 | 9.5537 | 11.4334 | 10.262 | 9.1962 | 10.0502 | 9.4219 | 9.1512 |
| 201462_at | 9805 | SCRN1 | secernin 1 | 0.04312 | 0.9321 | 0.9855 | 7.0932 | 7.2 | 7.2 | 7.2167 | 6.8551 | 6.7472 | 7.266 | 7.3377 | 6.8636 | 6.8675 | 7.0042 | 6.8126 |
| 206050_s_at | 6050 | RNH1 | ribonuclease/angiogenin inhibitor 1 | 0.04238 | 0.9461 | 0.9921 | 8.5141 | 8.7457 | 8.8492 | 6.8236 | 8.5472 | 10.5113 | 9.1623 | 9.0383 | 9.0733 | 9.0983 | 9.3308 | 8.9897 |
| 201397_at | 26227 | PHGDH | phosphoglycerate dehydrogenase | 0.04172 | 0.9042 | 0.9825 | 11.4204 | 11.3348 | 11.5374 | 11.5626 | 11.6611 | 11.5842 | 10.9691 | 11.617 | 11.0292 | 11.3003 | 11.3911 | 11.5189 |
| 220094_s_at | 63933 | CCDC90A | coiled-coil domain containing 90A | 0.04162 | 0.8762 | 0.9749 | 6.4805 | 7.0007 | 5.9126 | 7.0954 | 6.8597 | 7.146 | 6.3388 | 5.9915 | 6.4805 | 6.3348 | 7.2396 | 6.3524 |
| 209526_s_at | 50810 | HDGFRP3 | hepatoma-derived growth factor, related protein 3 | 0.04076 | 0.6327 | 0.9127 | 7.7351 | 8.0104 | 8.0132 | 7.9512 | 7.8326 | 7.5528 | 7.2881 | 7.6974 | 7.375 | 7.4715 | 7.5013 | 7.499 |
| 209100_at | 7866 | IFRD2 | interferon-related developmental regulator 2 | 0.03544 | 0.8383 | 0.9716 | 10.4396 | 10.3997 | 10.2223 | 10.4579 | 10.5248 | 10.7927 | 10.2024 | 10.4133 | 10.0141 | 10.7811 | 10.3258 | 10.3258 |
| 212689_s_at | 55818 | KDM3A | lysine (K)-specific demethylase 3A | 0.03453 | 0.7904 | 0.9716 | 8.633 | 8.7844 | 8.8894 | 4.3799 | 8.5869 | 8.5749 | 8.261 | 8.8182 | 8.3516 | 8.732 | 8.557 | 8.446 |
| 209161_at | 9128 | PRPF4 | PRP4 pre-mRNA processing factor 4 homolog (yeast) | 0.03255 | 0.8363 | 0.9716 | 9.706 | 9.5094 | 9.6609 | 4.8949 | 9.5009 | 9.5238 | 9.3022 | 9.0812 | 9.0656 | 9.0574 | 6.1155 | 8.9869 |
| 202087_s_at | 1514 | CTSL1 | cathepsin L1 | 0.03072 | 0.8782 | 0.9749 | 9.9759 | 9.995 | 10.112 | 9.6609 | 10.045 | 9.455 | 9.7498 | 9.995 | 9.7373 | 9.9658 | 9.863 | 9.405 |
| 207847_s_at | 4582 | MUC1 | mucin 1, cell surface associated | 0.03019 | 0.8283 | 0.9716 | 4.65 | 4.3669 | 4.1172 | 4.1889 | 4.1889 | 4.1889 | 4.0252 | 4.4486 | 4.0871 | 4.3452 | 4.151 | 4.2034 |
| 201870_at | 10953 | TOMM34 | translocase of outer mitochondrial membrane 34 | 0.02893 | 0.8882 | 0.9782 | 9.0765 | 8.8671 | 9.1512 | 9.2876 | 9.2815 | 8.8377 | 8.78 | 9.0523 | 8.7153 | 8.8914 | 9.0228 | 8.5966 |
| 218104_at | 54881 | TEX10 | testis expressed 10 | 0.02817 | 0.8583 | 0.9716 | 8.3623 | 8.3425 | 8.2519 | 8.1918 | 8.2018 | 5.1258 | 8.1878 | 8.3107 | 7.9566 | 7.9512 | 8.0555 | 7.5854 |
| 203342_at | 10245 | TIMM17B | translocase of inner mitochondrial membrane 17 homolog B (yeast) | 0.02776 | 0.8842 | 0.9782 | 12.2293 | 13.0626 | 13.0053 | 12.3004 | 12.2244 | 12.3455 | 12.2244 | 13.363 | 12.1301 | 12.3753 | 12.7071 | 12.1763 |
| 204610_s_at | 11007 | CCDC85B | coiled-coil domain containing 85B | 0.02697 | 0.8503 | 0.9716 | 6.7289 | 6.5769 | 6.2473 | 6.2057 | 6.6183 | 7.3609 | 5.9329 | 6.4376 | 6.0038 | 6.289 | 5.7604 | 7.1681 |
| 204126_s_at | 8318 | CDC45 | cell division cycle 45 homolog (S. cerevisiae) | 0.02506 | 0.6327 | 0.9127 | 9.8495 | 9.7561 | 9.776 | 9.9901 | 10.0188 | 9.9658 | 9.7373 | 10.0005 | 10.3469 | 10.2455 | 10.0946 | 10.045 |
| 202945_at | 2356 | FPGS | folylpolyglutamate synthase | 0.02487 | 0.9222 | 0.9845 | 7.146 | 7.0535 | 7.6527 | 9.1831 | 7.241 | 7.5357 | 6.8907 | 7.1835 | 8.8128 | 7.3806 | 7.1835 | 7.0977 |
| 201833_at | 3066 | HDAC2 | histone deacetylase 2 | 0.02363 | 1 | 1 | 10.2223 | 10.3074 | 10.3106 | 10.2024 | 10.1416 | 10.0593 | 10.0817 | 10.1057 | 9.5657 | 9.9133 | 10.0554 | 10.2455 |
| 211783_s_at | 9112 | MTA1 | metastasis associated 1 | 0.02249 | 0.8703 | 0.9716 | 7.6044 | 8.3689 | 7.6545 | 7.6365 | 8.7683 | 7.4662 | 7.0275 | 8.3839 | 7.3415 | 7.3848 | 8.5141 | 8.6562 |
| 204617_s_at | 65057 | ACD | adrenocortical dysplasia homolog (mouse) | 0.02237 | 0.9242 | 0.9845 | 7.8948 | 7.4942 | 6.4805 | 7.5612 | 7.5385 | 7.5446 | 7.4033 | 7.7413 | 8.028 | 7.4558 | 7.4873 | 7.2654 |
| 201795_at | 3930 | LBR | lamin B receptor | 0.02194 | 0.9002 | 0.9825 | 9.5537 | 9.7142 | 9.7015 | 9.706 | 9.41 | 9.4768 | 9.2516 | 9.9468 | 9.2079 | 9.5814 | 9.4483 | 9.3568 |
| 204828_at | 5883 | RAD9A | RAD9 homolog A (S. pombe) | 0.02133 | 0.8204 | 0.9716 | 5.4862 | 5.523 | 5.7242 | 5.6458 | 5.7469 | 5.2488 | 4.9229 | 5.5664 | 5.3085 | 5.9126 | 5.0618 | 5.473 |
| 202679_at | 4864 | NPC1 | Niemann-Pick disease, type C1 | 0.01975 | 0.9022 | 0.9825 | 7.5244 | 7.0176 | 7.3415 | 7.3639 | 7.4662 | 7.0995 | 8.2886 | 7.7856 | 6.7726 | 6.873 | 7.105 | 7.594 |
| 203395_s_at | 3280 | HES1 | hairy and enhancer of split 1, (Drosophila) | 0.01936 | 0.9261 | 0.9845 | 8.6858 | 8.557 | 10.2832 | 8.6882 | 8.5966 | 8.4101 | 8.8613 | 8.6729 | 9.8495 | 8.4622 | 8.4049 | 9.3333 |
| 218039_at | 51203 | NUSAP1 | nucleolar and spindle associated protein 1 | 0.01934 | 0.8403 | 0.9716 | 9.8445 | 9.6171 | 9.6785 | 9.6908 | 5.4905 | 9.6038 | 9.3333 | 9.3958 | 9.776 | 9.4483 | 5.7748 | 9.6213 |
| 201471_s_at | 8878 | SQSTM1 | sequestosome 1 | 0.01823 | 0.976 | 0.9921 | 9.4092 | 9.3308 | 9.753 | 10.0005 | 9.4008 | 10.1184 | 9.5814 | 9.7719 | 9.4483 | 9.9373 | 9.3603 | 9.753 |
| 212894_at | 6832 | SUPV3L1 | suppressor of var1, 3-like 1 (S. cerevisiae) | 0.01804 | 0.8503 | 0.9716 | 9.9192 | 9.4599 | 9.0088 | 9.2467 | 9.4219 | 9.0088 | 9.2316 | 9.203 | 10.2223 | 9.8546 | 10.045 | 10.1184 |
| 202329_at | 1445 | CSK | c-src tyrosine kinase | 0.01623 | 0.9281 | 0.9845 | 8.8748 | 8.9432 | 9.4008 | 9.2204 | 8.9351 | 9.3022 | 9.0656 | 9.2633 | 8.9398 | 9.1512 | 8.8748 | 8.7683 |
| 218794_s_at | 54957 | TXNL4B | thioredoxin-like 4B | 0.01564 | 0.8663 | 0.9716 | 7.5064 | 7.4385 | 7.1924 | 7.49 | 7.2925 | 7.5064 | 7.0843 | 6.8938 | 8.205 | 7.358 | 7.3549 | 7.1012 |
| 201561_s_at | 22883 | CLSTN1 | calsyntenin 1 | 0.01426 | 0.8822 | 0.9782 | 8.6476 | 7.6545 | 7.5727 | 8.0446 | 7.5337 | 7.3848 | 7.3258 | 7.6595 | 7.3914 | 7.3188 | 7.4188 | 7.9078 |
| 200666_s_at | 3337 | DNAJB1 | DnaJ (Hsp40) homolog, subfamily B, member 1 | 0.01355 | 0.9242 | 0.9845 | 9.9468 | 10.1602 | 10.4237 | 10.2086 | 10.491 | 10.1057 | 9.8973 | 10.1602 | 9.6785 | 10.3927 | 10.1057 | 10.0188 |
| 203574_at | 4783 | NFIL3 | nuclear factor, interleukin 3 regulated | 0.01329 | 0.984 | 0.9921 | 6.607 | 6.7808 | 6.624 | 6.765 | 6.519 | 6.486 | 6.3706 | 6.8126 | 6.8316 | 6.413 | 6.7251 | 6.4307 |
| 217838_s_at | 51466 | EVL | Enah/Vasp-like | 0.0128 | 0.9701 | 0.9921 | 5.5037 | 5.4686 | 5.4388 | 6.157 | 5.6226 | 5.8411 | 6.6193 | 5.4862 | 5.3609 | 5.4028 | 5.473 | 5.5548 |
| 218498_s_at | 30001 | ERO1L | ERO1-like (S. cerevisiae) | 0.01243 | 0.6327 | 0.9127 | 6.7033 | 6.8842 | 6.5375 | 7.0042 | 6.5894 | 6.7693 | 6.3601 | 6.7156 | 6.1444 | 6.4478 | 6.0488 | 5.9659 |
| 203952_at | 22926 | ATF6 | activating transcription factor 6 | 0.01233 | 0.986 | 0.9931 | 8.6099 | 8.5749 | 8.8748 | 8.7116 | 8.5749 | 9.0922 | 9.2686 | 8.9622 | 8.6099 | 8.7366 | 8.8182 | 8.8894 |
| 202009_at | 11344 | TWF2 | twinfilin, actin-binding protein, homolog 2 (Drosophila) | 0.01168 | 0.9002 | 0.9825 | 6.9198 | 6.7289 | 7.1474 | 6.9556 | 6.7726 | 7.044 | 6.4376 | 6.7156 | 6.486 | 6.8842 | 6.8406 | 6.6204 |
| 202780_at | 5019 | OXCT1 | 3-oxoacid CoA transferase 1 | 0.01108 | 0.9281 | 0.9845 | 8.3443 | 8.1089 | 7.8155 | 7.9131 | 7.8544 | 7.3258 | 7.5013 | 8.3754 | 8.296 | 7.8094 | 7.7856 | 7.5805 |
| 203931_s_at | 6182 | MRPL12 | mitochondrial ribosomal protein L12 | 0.009423 | 0.98 | 0.9921 | 12.7071 | 12.5517 | 12.7071 | 12.662 | 12.9163 | 12.9163 | 12.7071 | 12.7591 | 12.0594 | 12.7071 | 12.3004 | 12.5889 |
| 205691_at | 9143 | SYNGR3 | synaptogyrin 3 | 0.007937 | 0.99 | 0.9951 | 4.4812 | 4.5509 | 4.356 | 4.601 | 4.7052 | 4.5613 | 4.4486 | 4.6702 | 4.7754 | 3.9141 | 4.2682 | 4.4486 |
| 204808_s_at | 10329 | TMEM5 | transmembrane protein 5 | 0.005693 | 0.996 | 0.998 | 10.1057 | 10.1261 | 10.1416 | 10.2455 | 9.9658 | 10.6154 | 10.3781 | 10.0612 | 9.647 | 10.2496 | 10.6225 | 10.0593 |
| 209806_at | 85236 | HIST1H2BK | histone cluster 1, H2bk | 0.004604 | 0.982 | 0.9921 | 12.019 | 11.809 | 11.2259 | 12.1301 | 11.1454 | 12.0917 | 11.6864 | 11.0543 | 10.5327 | 12.2004 | 10.891 | 12.0917 |
| 203228_at | 5050 | PAFAH1B3 | platelet-activating factor acetylhydrolase 1b, catalytic subunit 3 (29kDa) | 0.003766 | 0.9581 | 0.9921 | 7.1171 | 7.1703 | 7.0597 | 7.4463 | 7.4911 | 7.5337 | 6.9556 | 7.2559 | 6.9893 | 7.249 | 7.2836 | 7.2559 |
| 200989_at | 3091 | HIF1A | hypoxia inducible factor 1, alpha subunit (basic helix-loop-helix transcription factor) | 0.002524 | 0.98 | 0.9921 | 12.9163 | 12.4958 | 12.5517 | 12.7591 | 12.7071 | 12.7591 | 12.8313 | 12.4511 | 12.5517 | 12.7591 | 12.4097 | 12.7591 |
| 202123_s_at | 25 | ABL1 | c-abl oncogene 1, non-receptor tyrosine kinase | 0.00123 | 0.982 | 0.9921 | 7.8575 | 7.8575 | 8.0728 | 8.1103 | 8.0728 | 7.8094 | 8.1475 | 7.6416 | 8.0555 | 7.7351 | 8.0384 | 7.8826 |
| 203897_at | 57149 | LYRM1 | LYR motif containing 1 | -0.001509 | 0.9421 | 0.9918 | 5.0322 | 5.3817 | 5.4862 | 4.685 | 4.9864 | 5.1062 | 5.2278 | 4.3669 | 4.9166 | 5.0739 | 4.9353 | 4.972 |
| 207805_s_at | 5715 | PSMD9 | proteasome (prosome, macropain) 26S subunit, non-ATPase, 9 | -0.001979 | 0.9401 | 0.9908 | 8.9136 | 8.9175 | 8.8587 | 8.6983 | 8.8748 | 8.8096 | 8.7012 | 9.0656 | 8.1565 | 8.8914 | 8.5113 | 8.7574 |
| 212660_at | 23338 | PHF15 | PHD finger protein 15 | -0.002283 | 0.8703 | 0.9716 | 3.641 | 3.641 | 3.7648 | 3.9141 | 6.9025 | 3.9983 | 6.7693 | 3.7648 | 3.7081 | 4.052 | 6.7783 | 4.0695 |
| 212500_at | 84890 | ADO | 2-aminoethanethiol (cysteamine) dioxygenase | -0.002364 | 0.9741 | 0.9921 | 8.1103 | 7.2091 | 7.5612 | 8.0728 | 6.8316 | 6.673 | 8.3098 | 8.4501 | 6.9712 | 7.1856 | 7.0535 | 7.0202 |
| 206571_s_at | 9448 | MAP4K4 | mitogen-activated protein kinase kinase kinase kinase 4 | -0.002438 | 0.9721 | 0.9921 | 5.6175 | 4.7754 | 4.8161 | 5.9035 | 6.0231 | 6.157 | 5.5664 | 5.8589 | 5.294 | 5.5548 | 5.8045 | 5.2092 |
| 201997_s_at | 23013 | SPEN | spen homolog, transcriptional regulator (Drosophila) | -0.002507 | 0.8323 | 0.9716 | 6.9922 | 10.5113 | 10.2496 | 10.5248 | 10.3682 | 10.5393 | 6.8675 | 7.0977 | 10.3927 | 10.2832 | 10.2455 | 10.5015 |
| 214435_x_at | 5898 | RALA | v-ral simian leukemia viral oncogene homolog A (ras related) | -0.003403 | 0.976 | 0.9921 | 8.4172 | 8.4735 | 8.4735 | 8.9295 | 8.5127 | 8.2923 | 8.7527 | 8.5996 | 8.0728 | 8.1543 | 8.3408 | 8.3224 |
| 202332_at | 1454 | CSNK1E | casein kinase 1, epsilon | -0.005568 | 0.9601 | 0.9921 | 11.237 | 11.1454 | 11.809 | 11.2259 | 11.8756 | 11.3348 | 10.8365 | 11.9462 | 11.7318 | 11.2078 | 11.8924 | 11.3348 |
| 212287_at | 23512 | SUZ12 | suppressor of zeste 12 homolog (Drosophila) | -0.008112 | 0.984 | 0.9921 | 10.6716 | 10.4579 | 10.584 | 10.6154 | 10.6225 | 10.3469 | 10.4318 | 10.6225 | 10.4318 | 10.5327 | 10.3469 | 10.5646 |
| 209408_at | 11004 | KIF2C | kinesin family member 2C | -0.00876 | 0.9521 | 0.9921 | 4.1172 | 4.0252 | 4.0871 | 4.1172 | 4.1172 | 4.052 | 3.8814 | 3.955 | 4.052 | 4.3164 | 3.955 | 4.2996 |
| 218046_s_at | 51021 | MRPS16 | mitochondrial ribosomal protein S16 | -0.01112 | 0.9641 | 0.9921 | 10.2455 | 10.0188 | 10.5886 | 6.7367 | 10.3308 | 10.262 | 9.9658 | 10.1261 | 9.8234 | 10.2959 | 7.4055 | 10.2986 |
| 204826_at | 899 | CCNF | cyclin F | -0.01211 | 0.978 | 0.9921 | 7.4973 | 7.7351 | 8.6983 | 7.9445 | 7.9728 | 7.6442 | 7.6629 | 7.5446 | 7.1877 | 7.5805 | 8.6099 | 8.3857 |
| 203665_at | 3162 | HMOX1 | heme oxygenase (decycling) 1 | -0.0122 | 0.9621 | 0.9921 | 5.9915 | 6.5375 | 6.2411 | 6.7033 | 5.7748 | 6.289 | 5.7514 | 6.4991 | 5.6226 | 6.707 | 5.6937 | 5.9873 |
| 202854_at | 3251 | HPRT1 | hypoxanthine phosphoribosyltransferase 1 | -0.01297 | 0.8563 | 0.9716 | 10.9664 | 11.1661 | 10.891 | 11.0034 | 10.9664 | 10.9184 | 10.3359 | 11.1454 | 11.4048 | 10.9321 | 11.0139 | 9.5765 |
| 201494_at | 5547 | PRCP | prolylcarboxypeptidase (angiotensinase C) | -0.01367 | 0.9581 | 0.9921 | 7.375 | 7.5244 | 7.4044 | 7.4732 | 7.2383 | 6.9123 | 7.2167 | 6.93 | 7.5337 | 7.1254 | 7.3914 | 7.1215 |
| 211980_at | 1282 | COL4A1 | collagen, type IV, alpha 1 | -0.01563 | 0.978 | 0.9921 | 8.5732 | 8.4172 | 8.5506 | 8.2886 | 8.249 | 8.3739 | 7.8928 | 8.1688 | 7.9327 | 8.6247 | 8.6025 | 8.6906 |
| 211297_s_at | 1022 | CDK7 | cyclin-dependent kinase 7 | -0.01615 | 0.9441 | 0.9921 | 10.3781 | 10.5393 | 10.8448 | 10.1385 | 10.3469 | 10.3682 | 10.2832 | 10.1416 | 9.9658 | 10.4396 | 10.3359 | 10.6038 |
| 209603_at | 2625 | GATA3 | GATA binding protein 3 | -0.01662 | 0.9261 | 0.9845 | 5.6716 | 5.6619 | 5.6937 | 5.8411 | 5.7604 | 9.9468 | 5.609 | 5.641 | 5.4553 | 5.3457 | 9.9689 | 8.6284 |
| 218145_at | 57761 | TRIB3 | tribbles homolog 3 (Drosophila) | -0.0182 | 0.9721 | 0.9921 | 9.5094 | 9.9229 | 8.5141 | 9.5715 | 9.9977 | 10.2832 | 9.5414 | 9.3958 | 10.9321 | 9.2467 | 10.1489 | 10.2959 |
| 202811_at | 10617 | STAMBP | STAM binding protein | -0.01938 | 0.9461 | 0.9921 | 9.863 | 9.5238 | 9.8376 | 9.8063 | 9.5363 | 9.2492 | 9.1196 | 9.6827 | 9.3603 | 9.5238 | 9.6648 | 9.3433 |
| 220140_s_at | 29916 | SNX11 | sorting nexin 11 | -0.02175 | 0.8862 | 0.9782 | 5.8589 | 5.6937 | 6.0193 | 6.0302 | 8.6833 | 5.9831 | 5.736 | 6.0078 | 5.6109 | 5.5182 | 5.4641 | 9.5765 |
| 218175_at | 80212 | CCDC92 | coiled-coil domain containing 92 | -0.02176 | 0.6327 | 0.9127 | 6.7693 | 6.4832 | 6.6348 | 6.5758 | 6.2911 | 7.4715 | 6.0914 | 6.1444 | 5.9626 | 10.7518 | 6.4346 | 6.2789 |
| 203848_at | 10270 | AKAP8 | A kinase (PRKA) anchor protein 8 | -0.02379 | 0.8443 | 0.9716 | 8.8492 | 9.0023 | 9.0472 | 9.1169 | 8.9574 | 7.3307 | 8.7153 | 9.1196 | 8.9058 | 8.654 | 8.7913 | 8.7366 |
| 203301_s_at | 9988 | DMTF1 | cyclin D binding myb-like transcription factor 1 | -0.02469 | 0.9002 | 0.9825 | 6.6183 | 7.7198 | 7.0111 | 7.8826 | 6.7472 | 7.795 | 7.7072 | 7.3055 | 7.7165 | 6.727 | 8.1521 | 7.1474 |
| 203288_at | 9710 | KIAA0355 | KIAA0355 | -0.02576 | 0.9361 | 0.9876 | 5.7922 | 6.8703 | 6.0329 | 5.9873 | 5.9277 | 5.7017 | 5.4354 | 6.5746 | 5.5797 | 5.7748 | 6.5425 | 5.3047 |
| 200791_s_at | 8826 | IQGAP1 | IQ motif containing GTPase activating protein 1 | -0.02613 | 0.9162 | 0.9845 | 7.9623 | 7.8745 | 7.8512 | 7.5854 | 8.296 | 7.9238 | 7.6536 | 8.2388 | 8.1881 | 7.7878 | 7.6005 | 7.8285 |
| 208945_s_at | 8678 | BECN1 | beclin 1, autophagy related | -0.02622 | 0.9202 | 0.9845 | 8.9175 | 8.5217 | 8.7116 | 8.8587 | 8.9622 | 8.6048 | 8.3656 | 9.0812 | 8.2886 | 8.6858 | 8.8981 | 8.495 |
| 207199_at | 7015 | TERT | telomerase reverse transcriptase | -0.02641 | 0.9261 | 0.9845 | 7.0284 | 7.3519 | 7.0745 | 7.1445 | 6.9462 | 7.3377 | 6.9893 | 6.9073 | 7.7231 | 7.0042 | 7.1254 | 6.9198 |
| 201614_s_at | 8607 | RUVBL1 | RuvB-like 1 (E. coli) | -0.02675 | 0.8643 | 0.9716 | 10.6909 | 10.1416 | 10.6622 | 10.5765 | 10.5393 | 10.8036 | 10.4012 | 10.7981 | 10.4133 | 10.3258 | 10.584 | 10.7112 |
| 200895_s_at | 2288 | FKBP4 | FK506 binding protein 4, 59kDa | -0.02758 | 0.9142 | 0.9845 | 13.6371 | 13.6371 | 12.4097 | 12.4958 | 12.7591 | 13.363 | 13.0626 | 12.465 | 14.6801 | 14.3602 | 14.3602 | 13.6371 |
| 208992_s_at | 6774 | STAT3 | signal transducer and activator of transcription 3 (acute-phase response factor) | -0.03032 | 0.8563 | 0.9716 | 3.1221 | 3.2809 | 3.4398 | 3.1221 | 3.2809 | 3.1221 | 3.4398 | 3.5495 | 3.4398 | 3.1221 | 3.1221 | 3.1221 |
| 202282_at | 3028 | HSD17B10 | hydroxysteroid (17-beta) dehydrogenase 10 | -0.03034 | 0.9301 | 0.9855 | 10.9929 | 11.2078 | 11.1141 | 11.4048 | 11.3234 | 11.237 | 11.0292 | 10.771 | 10.7277 | 10.9381 | 11.0292 | 10.9719 |
| 219573_at | 55604 | LRRC16A | leucine rich repeat containing 16A | -0.03063 | 0.6527 | 0.9211 | 5.7835 | 5.8045 | 5.8474 | 6.1796 | 6.0683 | 6.295 | 5.8474 | 5.9795 | 5.3755 | 5.9102 | 5.9114 | 5.2182 |
| 218358_at | 79174 | CRELD2 | cysteine-rich with EGF-like domains 2 | -0.03193 | 0.9281 | 0.9845 | 8.9574 | 7.4732 | 7.0474 | 7.0859 | 7.1725 | 7.0859 | 7.0932 | 7.1703 | 9.0472 | 7.9595 | 8.4002 | 7.4435 |
| 207877_s_at | 4931 | NVL | nuclear VCP-like | -0.03611 | 0.7984 | 0.9716 | 10.3682 | 10.8535 | 9.8325 | 9.9468 | 10.518 | 11.0292 | 12.0363 | 11.0292 | 11.8756 | 10.7112 | 10.9321 | 11.1141 |
| 218316_at | 26520 | TIMM9 | translocase of inner mitochondrial membrane 9 homolog (yeast) | -0.03809 | 0.9481 | 0.9921 | 12.1067 | 12.019 | 6.8104 | 11.7843 | 12.2004 | 11.9984 | 11.8563 | 11.8756 | 11.9462 | 12.2244 | 7.0535 | 12.019 |
| 37152_at | 5467 | PPARD | peroxisome proliferator-activated receptor delta | -0.03847 | 0.8942 | 0.9815 | 5.5138 | 5.5514 | 5.432 | 5.7514 | 5.7922 | 5.5732 | 5.3176 | 5.4641 | 5.5606 | 5.2278 | 5.8474 | 5.3933 |
| 206770_s_at | 23443 | SLC35A3 | solute carrier family 35 (UDP-N-acetylglucosamine (UDP-GlcNAc) transporter), member A3 | -0.03991 | 0.8882 | 0.9782 | 6.1289 | 5.9009 | 5.7423 | 6.1155 | 5.641 | 5.6175 | 5.7204 | 5.9488 | 5.6716 | 5.4195 | 5.8983 | 5.6868 |
| 201185_at | 5654 | HTRA1 | HtrA serine peptidase 1 | -0.04079 | 0.9361 | 0.9876 | 4.7052 | 5.341 | 5.5606 | 5.3875 | 4.65 | 5.4536 | 5.3755 | 5.7922 | 4.5899 | 6.0718 | 5.8229 | 4.7565 |
| 201788_at | 11325 | DDX42 | DEAD (Asp-Glu-Ala-Asp) box polypeptide 42 | -0.04288 | 0.8703 | 0.9716 | 7.4385 | 7.499 | 7.594 | 7.3467 | 7.6416 | 7.3944 | 7.0977 | 7.5198 | 7.2118 | 7.1681 | 6.8842 | 7.3188 |
| 209092_s_at | 51031 | GLOD4 | glyoxalase domain containing 4 | -0.04339 | 0.8483 | 0.9716 | 10.5327 | 10.0688 | 10.6469 | 10.0593 | 10.4689 | 10.5327 | 9.7015 | 10.5886 | 9.8973 | 10.7789 | 10.5886 | 10.4237 |
| 219485_s_at | 5716 | PSMD10 | proteasome (prosome, macropain) 26S subunit, non-ATPase, 10 | -0.04519 | 0.8263 | 0.9716 | 8.546 | 8.4664 | 9.0263 | 7.4662 | 7.5198 | 8.502 | 8.7079 | 7.2343 | 8.3158 | 8.3248 | 8.1521 | 7.6168 |
| 203725_at | 1647 | GADD45A | growth arrest and DNA-damage-inducible, alpha | -0.04524 | 0.8244 | 0.9716 | 8.4101 | 8.7732 | 8.6562 | 8.6391 | 8.1089 | 8.8613 | 8.7366 | 7.8094 | 8.9622 | 8.8492 | 9.2316 | 8.7978 |
| 202749_at | 7485 | WRB | tryptophan rich basic protein | -0.04561 | 0.8523 | 0.9716 | 7.2654 | 7.1254 | 7.1746 | 7.241 | 6.0076 | 6.836 | 6.673 | 7.0007 | 6.0038 | 6.9073 | 7.105 | 6.0004 |
| 204549_at | 9641 | IKBKE | inhibitor of kappa light polypeptide gene enhancer in B-cells, kinase epsilon | -0.04608 | 0.9082 | 0.9825 | 4.5225 | 4.2682 | 4.2682 | 4.4486 | 4.7131 | 4.8949 | 4.3669 | 4.4147 | 4.7983 | 4.8042 | 4.601 | 4.3452 |
| 219363_s_at | 51001 | MTERFD1 | MTERF domain containing 1 | -0.04691 | 0.6747 | 0.9346 | 9.7285 | 9.9468 | 10.2959 | 9.8849 | 10.0946 | 9.7015 | 9.7142 | 9.9229 | 9.8445 | 10.1602 | 9.9658 | 10.2055 |
| 203302_at | 1633 | DCK | deoxycytidine kinase | -0.04951 | 0.8583 | 0.9716 | 9.5414 | 10.3359 | 9.2323 | 9.5363 | 9.3308 | 9.5009 | 9.5117 | 10.4318 | 9.1143 | 9.1623 | 10.2024 | 9.0574 |
| 209029_at | 50813 | COPS7A | COP9 constitutive photomorphogenic homolog subunit 7A (Arabidopsis) | -0.05158 | 0.8623 | 0.9716 | 10.3997 | 10.2552 | 10.3781 | 10.3682 | 10.2086 | 10.4841 | 10.2496 | 11.4472 | 10.0688 | 10.3074 | 10.1868 | 10.3576 |
| 212070_at | 9289 | GPR56 | G protein-coupled receptor 56 | -0.05376 | 0.9501 | 0.9921 | 7.358 | 7.6527 | 7.0824 | 10.7811 | 7.3292 | 8.0321 | 7.9802 | 7.5013 | 8.5141 | 7.838 | 8.1521 | 8.2172 |
| 218014_at | 79902 | NUP85 | nucleoporin 85kDa | -0.05397 | 0.8623 | 0.9716 | 11.0139 | 10.891 | 11.2078 | 11.1661 | 4.8102 | 11.3003 | 10.7518 | 11.0744 | 10.9929 | 10.9929 | 10.6909 | 10.4772 |
| 218292_s_at | 51422 | PRKAG2 | protein kinase, AMP-activated, gamma 2 non-catalytic subunit | -0.05714 | 0.9082 | 0.9825 | 6.3433 | 6.1727 | 6.1406 | 6.2691 | 6.2758 | 5.8059 | 5.8229 | 6.1727 | 6.4929 | 6.2411 | 6.6348 | 5.706 |
| 203047_at | 6793 | STK10 | serine/threonine kinase 10 | -0.06041 | 0.7625 | 0.9685 | 6.289 | 6.0818 | 6.5425 | 6.486 | 6.2326 | 6.0275 | 6.937 | 6.413 | 6.54 | 6.2131 | 6.3809 | 6.7107 |
| 212849_at | 8312 | AXIN1 | axin 1 | -0.06138 | 0.8283 | 0.9716 | 8.1565 | 8.0672 | 8.1874 | 8.5388 | 8.2886 | 8.3197 | 7.9512 | 7.8826 | 8.0672 | 8.1748 | 7.7772 | 7.8401 |
| 209191_at | 84617 | TUBB6 | tubulin, beta 6 | -0.06177 | 0.7984 | 0.9716 | 5.2689 | 5.4388 | 9.5376 | 5.3875 | 9.5238 | 9.3333 | 4.8469 | 5.3556 | 5.5797 | 5.2182 | 9.5067 | 9.4345 |
| 202394_s_at | 55324 | ABCF3 | ATP-binding cassette, sub-family F (GCN20), member 3 | -0.06194 | 0.8363 | 0.9716 | 7.3307 | 6.7904 | 7.6742 | 7.5783 | 6.7251 | 7.4336 | 7.266 | 7.5652 | 6.9556 | 6.8458 | 7.2247 | 7.4732 |
| 202494_at | 10450 | PPIE | peptidylprolyl isomerase E (cyclophilin E) | -0.06269 | 0.7884 | 0.9711 | 10.5015 | 10.3682 | 10.3997 | 10.6469 | 10.4237 | 10.2086 | 10.2455 | 10.7025 | 9.5094 | 10.262 | 10.262 | 10.3682 |
| 218350_s_at | 51053 | GMNN | geminin, DNA replication inhibitor | -0.06273 | 0.8144 | 0.9716 | 13.0053 | 13.0053 | 12.662 | 12.8313 | 13.0053 | 12.8313 | 12.7591 | 12.662 | 12.662 | 12.9163 | 13.0626 | 13.0053 |
| 209435_s_at | 9181 | ARHGEF2 | Rho/Rac guanine nucleotide exchange factor (GEF) 2 | -0.06323 | 0.7525 | 0.9685 | 8.0132 | 8.2084 | 8.2084 | 8.4338 | 7.2559 | 8.1688 | 7.9661 | 8.094 | 7.9071 | 8.0728 | 7.8326 | 8.0874 |
| 205088_at | 10046 | MAMLD1 | mastermind-like domain containing 1 | -0.06407 | 0.7405 | 0.9683 | 4.8362 | 12.4511 | 5.0107 | 5.0263 | 4.9026 | 4.65 | 4.6246 | 11.4334 | 4.8362 | 11.5626 | 4.8161 | 5.0786 |
| 203073_at | 22796 | COG2 | component of oligomeric golgi complex 2 | -0.06591 | 0.7924 | 0.9716 | 8.1021 | 7.1768 | 8.4825 | 7.249 | 7.1924 | 8.1808 | 7.3639 | 8.4172 | 8.2633 | 8.1952 | 7.7072 | 8.1096 |
| 202040_s_at | 5927 | KDM5A | lysine (K)-specific demethylase 5A | -0.06637 | 0.8523 | 0.9716 | 7.8826 | 7.8826 | 7.8094 | 8.0043 | 8.8128 | 8.1127 | 7.7461 | 8.6562 | 7.6257 | 7.6742 | 8.5472 | 7.6025 |
| 200887_s_at | 6772 | STAT1 | signal transducer and activator of transcription 1, 91kDa | -0.06654 | 0.7086 | 0.948 | 10.5248 | 7.8826 | 7.5371 | 7.5312 | 7.6257 | 7.5612 | 10.0094 | 7.3639 | 7.3824 | 7.7011 | 7.3277 | 10.6469 |
| 205349_at | 2769 | GNA15 | guanine nucleotide binding protein (G protein), alpha 15 (Gq class) | -0.06674 | 0.7964 | 0.9716 | 5.7166 | 5.0692 | 9.2316 | 4.9657 | 4.7565 | 5.1707 | 5.1707 | 8.1521 | 8.8209 | 5.6619 | 5.6606 | 5.4862 |
| 213720_s_at | 6597 | SMARCA4 | SWI/SNF related, matrix associated, actin dependent regulator of chromatin, subfamily a, member 4 | -0.06702 | 0.8543 | 0.9716 | 8.0555 | 8.0132 | 8.115 | 8.0874 | 8.2172 | 8.0446 | 8.0536 | 8.1402 | 7.5288 | 7.9974 | 7.9566 | 7.7856 |
| 208717_at | 5018 | OXA1L | oxidase (cytochrome c) assembly 1-like | -0.06788 | 0.8343 | 0.9716 | 11.6611 | 11.4966 | 8.028 | 11.4966 | 11.6409 | 11.7318 | 11.2801 | 11.5189 | 10.0141 | 11.7662 | 11.5374 | 11.4334 |
| 202095_s_at | 332 | BIRC5 | baculoviral IAP repeat containing 5 | -0.06816 | 0.8283 | 0.9716 | 10.7518 | 10.3106 | 10.5248 | 10.2308 | 10.2986 | 11.0744 | 10.2959 | 10.3106 | 10.1954 | 10.5393 | 10.3997 | 10.5248 |
| 203068_at | 9903 | KLHL21 | kelch-like 21 (Drosophila) | -0.06872 | 0.5908 | 0.8986 | 4.7754 | 4.8651 | 5.1494 | 4.9864 | 5.1062 | 4.9229 | 4.8469 | 4.685 | 4.7983 | 4.5161 | 4.8042 | 4.7983 |
| 206364_at | 9928 | KIF14 | kinesin family member 14 | -0.06968 | 0.8184 | 0.9716 | 6.836 | 5.9915 | 6.9922 | 6.0847 | 6.9865 | 5.6799 | 6.3575 | 5.9169 | 6.4376 | 6.0877 | 6.6825 | 6.7693 |
| 209189_at | 2353 | FOS | FBJ murine osteosarcoma viral oncogene homolog | -0.07011 | 0.9082 | 0.9825 | 6.8515 | 6.4441 | 6.7607 | 6.5867 | 6.3138 | 6.7228 | 6.5524 | 6.4441 | 6.4376 | 6.3575 | 6.3247 | 6.3105 |
| 210986_s_at | 7168 | TPM1 | tropomyosin 1 (alpha) | -0.07088 | 0.8144 | 0.9716 | 8.2704 | 8.3224 | 8.3723 | 7.8155 | 7.7733 | 8.2923 | 7.8512 | 8.4985 | 8.0481 | 8.3088 | 7.848 | 7.7811 |
| 202862_at | 2184 | FAH | fumarylacetoacetate hydrolase (fumarylacetoacetase) | -0.07347 | 0.994 | 0.9971 | 5.7104 | 5.6109 | 5.5138 | 5.6799 | 5.7104 | 5.9169 | 5.4195 | 5.609 | 6.6399 | 6.2209 | 5.4354 | 5.609 |
| 207968_s_at | 4208 | MEF2C | myocyte enhancer factor 2C | -0.07524 | 0.6267 | 0.9127 | 6.2011 | 6.2268 | 6.3105 | 6.6276 | 6.282 | 6.5375 | 6.3308 | 6.4268 | 5.9102 | 5.8474 | 6.3524 | 6.1289 |
| 218399_s_at | 55038 | CDCA4 | cell division cycle associated 4 | -0.07642 | 0.8743 | 0.9738 | 6.0359 | 6.1865 | 6.6524 | 6.5023 | 6.2473 | 6.5301 | 6.1406 | 6.3121 | 5.938 | 6.1021 | 6.1444 | 6.0078 |
| 221492_s_at | 64422 | ATG3 | ATG3 autophagy related 3 homolog (S. cerevisiae) | -0.07734 | 0.8862 | 0.9782 | 9.6129 | 9.6908 | 9.6875 | 6.2011 | 9.6038 | 9.6247 | 9.3433 | 9.5177 | 9.455 | 9.7015 | 9.5814 | 9.4259 |
| 209586_s_at | 58497 | PRUNE | prune homolog (Drosophila) | -0.07835 | 0.8204 | 0.9716 | 5.1258 | 5.0835 | 5.2837 | 5.1828 | 5.1707 | 4.9103 | 5.0412 | 5.4195 | 5.2037 | 4.8949 | 4.6951 | 5.1258 |
| 201212_at | 5641 | LGMN | legumain | -0.07964 | 0.8104 | 0.9716 | 6.5894 | 6.6373 | 6.7726 | 6.8479 | 6.6373 | 6.5355 | 6.2209 | 6.873 | 6.4541 | 6.8938 | 6.496 | 6.4805 |
| 219281_at | 4482 | MSRA | methionine sulfoxide reductase A | -0.08034 | 0.7525 | 0.9685 | 5.9693 | 5.8625 | 5.6326 | 6.0078 | 6.0004 | 6.8978 | 5.8783 | 5.9626 | 6.7607 | 6.1021 | 7.2925 | 5.8749 |
| 200711_s_at | 6500 | SKP1 | S-phase kinase-associated protein 1 | -0.08049 | 0.8004 | 0.9716 | 6.3588 | 6.4589 | 7.2925 | 7.4873 | 6.6276 | 7.0111 | 7.0176 | 6.836 | 6.6638 | 6.8316 | 7.0859 | 6.2691 |
| 200060_s_at | 10921 | RNPS1 | RNA binding protein S1, serine-rich domain | -0.08053 | 0.8523 | 0.9716 | 14.3602 | 13.2279 | 13.363 | 13.9353 | 13.6371 | 13.6371 | 13.0053 | 13.6371 | 14.0921 | 14.0921 | 13.363 | 13.363 |
| 202382_s_at | 10007 | GNPDA1 | glucosamine-6-phosphate deaminase 1 | -0.08068 | 0.8024 | 0.9716 | 9.2316 | 9.2936 | 9.5238 | 9.3198 | 9.2754 | 9.1386 | 9.3099 | 9.5009 | 8.9351 | 9.032 | 9.1706 | 9.1962 |
| 203171_s_at | 23378 | RRP8 | ribosomal RNA processing 8, methyltransferase, homolog (yeast) | -0.0813 | 0.7844 | 0.9711 | 7.0805 | 6.923 | 7.1202 | 7.1877 | 6.8104 | 6.9335 | 6.7758 | 7.0333 | 6.9198 | 7.0229 | 7.0229 | 6.8458 |
| 208190_s_at | 51599 | LSR | lipolysis stimulated lipoprotein receptor | -0.08205 | 0.7645 | 0.9685 | 6.8458 | 6.7758 | 6.8406 | 6.7367 | 6.923 | 7.5198 | 7.1474 | 6.6638 | 6.5091 | 7.2925 | 6.9757 | 7.0411 |
| 203379_at | 6195 | RPS6KA1 | ribosomal protein S6 kinase, 90kDa, polypeptide 1 | -0.0825 | 0.998 | 0.999 | 8.6517 | 8.5096 | 9.0765 | 9.0581 | 8.8311 | 8.9855 | 8.5141 | 8.4555 | 8.6983 | 8.6808 | 8.5919 | 8.5113 |
| 201756_at | 6118 | RPA2 | replication protein A2, 32kDa | -0.08257 | 0.98 | 0.9921 | 11.3911 | 11.4204 | 11.3348 | 11.237 | 11.237 | 10.6326 | 10.5015 | 11.1661 | 13.0626 | 10.891 | 11.1962 | 11.3234 |
| 1007_s_at | 780 | DDR1 | discoidin domain receptor tyrosine kinase 1 | -0.08272 | 0.7545 | 0.9685 | 5.8715 | 6.2057 | 5.7922 | 6.0466 | 6.2057 | 7.1962 | 6.8551 | 6.5867 | 6.299 | 6.1406 | 6.4199 | 6.7904 |
| 203705_s_at | 8324 | FZD7 | frizzled family receptor 7 | -0.08437 | 0.9621 | 0.9921 | 9.1315 | 8.581 | 8.9136 | 8.5167 | 8.78 | 7.8967 | 8.1305 | 8.6071 | 10.2024 | 8.4815 | 8.261 | 8.4049 |
| 212557_at | 26036 | ZNF451 | zinc finger protein 451 | -0.08477 | 0.8144 | 0.9716 | 7.375 | 7.461 | 7.583 | 7.3881 | 7.2064 | 7.1835 | 6.9757 | 7.2881 | 7.2207 | 7.4385 | 7.2 | 7.2142 |
| 209112_at | 1027 | CDKN1B | cyclin-dependent kinase inhibitor 1B (p27, Kip1) | -0.08535 | 0.9641 | 0.9921 | 12.8313 | 12.8313 | 5.6606 | 13.0053 | 12.662 | 12.5889 | 12.3753 | 13.0626 | 12.2342 | 12.662 | 12.4958 | 12.4958 |
| 202763_at | 836 | CASP3 | caspase 3, apoptosis-related cysteine peptidase | -0.08598 | 0.8084 | 0.9716 | 7.9916 | 8.5919 | 8.6368 | 8.4892 | 8.9869 | 8.0709 | 8.7157 | 8.1103 | 8.3754 | 8.502 | 8.4166 | 9.032 |
| 212462_at | 23522 | KAT6B | K(lysine) acetyltransferase 6B | -0.08635 | 0.7625 | 0.9685 | 7.3415 | 6.5355 | 6.2209 | 6.3163 | 7.1292 | 6.3187 | 6.0004 | 6.3758 | 6.0078 | 5.8148 | 6.9214 | 7.0411 |
| 205172_x_at | 1212 | CLTB | clathrin, light chain B | -0.08689 | 0.7405 | 0.9683 | 7.9445 | 7.41 | 6.4541 | 7.5064 | 7.7351 | 8.0781 | 7.2118 | 6.923 | 8.6284 | 8.1952 | 8.0709 | 7.5761 |
| 213417_at | 6909 | TBX2 | T-box 2 | -0.08739 | 0.6487 | 0.9195 | 5.9795 | 5.9277 | 5.8474 | 5.9488 | 6.0231 | 6.0155 | 5.9102 | 6.0078 | 6.0275 | 5.7925 | 5.726 | 5.5982 |
| 221741_s_at | 54915 | YTHDF1 | YTH domain family, member 1 | -0.08747 | 0.7645 | 0.9685 | 6.4894 | 7.6365 | 6.7808 | 6.5758 | 6.5355 | 6.4307 | 6.5375 | 6.4589 | 7.5897 | 7.4463 | 7.4942 | 7.5528 |
| 205036_at | 11157 | LSM6 | LSM6 homolog, U6 small nuclear RNA associated (S. cerevisiae) | -0.09031 | 0.7804 | 0.9711 | 8.8613 | 8.8748 | 9.3333 | 8.6729 | 9.0383 | 8.9671 | 8.8492 | 9.3009 | 8.3822 | 9.0088 | 8.6284 | 8.8613 |
| 201855_s_at | 23300 | ATMIN | ATM interactor | -0.09074 | 0.7824 | 0.9711 | 6.5365 | 7.0252 | 6.3809 | 6.8978 | 6.4832 | 6.7033 | 6.4541 | 7.0859 | 6.7423 | 6.9865 | 6.6524 | 6.4515 |
| 205607_s_at | 57147 | SCYL3 | SCY1-like 3 (S. cerevisiae) | -0.09165 | 0.6707 | 0.933 | 6.1865 | 6.2473 | 6.282 | 6.0847 | 6.1608 | 3.955 | 5.7748 | 6.0359 | 5.432 | 5.938 | 5.7423 | 5.6868 |
| 201243_s_at | 481 | ATP1B1 | ATPase, Na+/K+ transporting, beta 1 polypeptide | -0.09176 | 0.7645 | 0.9685 | 9.8849 | 9.7719 | 9.995 | 10.2552 | 9.955 | 9.647 | 10.0321 | 9.8495 | 9.7015 | 9.5894 | 9.9531 | 9.3685 |
| 204313_s_at | 1385 | CREB1 | cAMP responsive element binding protein 1 | -0.09253 | 0.7824 | 0.9711 | 6.7758 | 6.8922 | 4.612 | 6.6825 | 4.5509 | 6.5425 | 5.1494 | 7.2343 | 7.0111 | 5.3755 | 6.645 | 6.486 |
| 213851_at | 375346 | TMEM110 | transmembrane protein 110 | -0.09266 | 0.9261 | 0.9845 | 6.8236 | 7.1012 | 7.0252 | 6.9161 | 6.73 | 7.3139 | 6.8842 | 6.6312 | 6.54 | 7.1012 | 6.7693 | 6.8675 |
| 201746_at | 7157 | TP53 | tumor protein p53 | -0.09278 | 0.7405 | 0.9683 | 7.9566 | 8.3107 | 8.8263 | 8.5894 | 8.5194 | 8.4002 | 8.115 | 8.7153 | 8.1207 | 8.6247 | 7.8165 | 8.3656 |
| 217758_s_at | 56889 | TM9SF3 | transmembrane 9 superfamily member 3 | -0.09425 | 0.7046 | 0.9479 | 7.7856 | 7.6629 | 9.2204 | 7.795 | 9.2876 | 8.9691 | 8.9691 | 8.9574 | 9.5238 | 8.1055 | 8.5324 | 7.9783 |
| 202659_at | 5699 | PSMB10 | proteasome (prosome, macropain) subunit, beta type, 10 | -0.09445 | 0.7924 | 0.9716 | 7.3089 | 7.3188 | 7.7351 | 7.3277 | 7.0381 | 7.3021 | 6.8406 | 7.7733 | 6.9893 | 7.9822 | 7.1474 | 7.1445 |
| 203201_at | 5373 | PMM2 | phosphomannomutase 2 | -0.09505 | 0.7246 | 0.9589 | 11.1291 | 10.1697 | 8.5665 | 9.7249 | 10.174 | 10.4499 | 12.2004 | 10.9533 | 7.6365 | 10.8682 | 11.1141 | 11.6409 |
| 211015_s_at | 3308 | HSPA4 | heat shock 70kDa protein 4 | -0.09582 | 0.7844 | 0.9711 | 11.8331 | 11.1378 | 10.9719 | 10.9184 | 10.9381 | 11.1661 | 10.9381 | 10.7981 | 12.4958 | 11.8331 | 12.1763 | 11.353 |
| 201125_s_at | 3693 | ITGB5 | integrin, beta 5 | -0.09648 | 0.8004 | 0.9716 | 6.7808 | 6.731 | 6.7251 | 6.8636 | 6.7904 | 6.5524 | 6.7205 | 6.5425 | 6.1242 | 6.3187 | 6.7967 | 6.7228 |
| 202545_at | 5580 | PRKCD | protein kinase C, delta | -0.09696 | 0.7465 | 0.9683 | 6.2549 | 6.1155 | 6.607 | 6.6599 | 6.5375 | 6.5054 | 6.6373 | 6.2911 | 6.3601 | 6.1727 | 6.5127 | 5.9211 |
| 209040_s_at | 5696 | PSMB8 | proteasome (prosome, macropain) subunit, beta type, 8 (large multifunctional peptidase 7) | -0.09794 | 0.7465 | 0.9683 | 5.1948 | 5.1612 | 5.294 | 5.1553 | 5.1965 | 6.475 | 5.4354 | 5.9795 | 5.1494 | 4.7131 | 6.2911 | 6.1155 |
| 202830_s_at | 2542 | SLC37A4 | solute carrier family 37 (glucose-6-phosphate transporter), member 4 | -0.09821 | 0.8942 | 0.9815 | 6.3872 | 6.3975 | 6.5758 | 6.3758 | 7.685 | 6.3601 | 6.3479 | 6.4589 | 6.0544 | 6.3433 | 6.2326 | 6.4408 |
| 203343_at | 7358 | UGDH | UDP-glucose 6-dehydrogenase | -0.09864 | 0.6747 | 0.9346 | 7.5692 | 7.5446 | 8.546 | 7.4188 | 8.546 | 8.0384 | 8.3197 | 7.3009 | 7.2881 | 8.4501 | 8.5388 | 8.0938 |
| 218898_at | 79850 | FAM57A | family with sequence similarity 57, member A | -0.09999 | 0.976 | 0.9921 | 6.8406 | 7.41 | 7.1528 | 7.2667 | 7.0597 | 7.4055 | 6.7251 | 7.3188 | 8.7157 | 7.3519 | 7.0745 | 7.1292 |
| 203627_at | 3480 | IGF1R | insulin-like growth factor 1 receptor | -0.1002 | 0.7545 | 0.9685 | 8.261 | 7.9783 | 9.2437 | 7.8745 | 7.9078 | 7.4911 | 7.6704 | 9.0523 | 7.7856 | 7.8733 | 7.499 | 7.1114 |
| 210754_s_at | 4067 | LYN | v-yes-1 Yamaguchi sarcoma viral related oncogene homolog | -0.1004 | 0.7365 | 0.9683 | 7.0381 | 6.9198 | 6.8236 | 7.0111 | 6.9488 | 7.6545 | 6.687 | 7.0623 | 7.3307 | 7.5001 | 6.8458 | 6.794 |
| 217140_s_at | 7416 | VDAC1 | voltage-dependent anion channel 1 | -0.1009 | 0.6846 | 0.9391 | 12.662 | 11.7662 | 12.3455 | 10.891 | 12.1067 | 12.4097 | 12.1763 | 11.7089 | 12.1763 | 12.5889 | 12.0594 | 12.2004 |
| 218845_at | 56940 | DUSP22 | dual specificity phosphatase 22 | -0.1026 | 0.6467 | 0.9193 | 7.0649 | 6.9296 | 8.7527 | 7.2967 | 7.0977 | 8.4622 | 8.3098 | 7.0623 | 8.3623 | 6.9965 | 8.3839 | 7.0284 |
| 202627_s_at | 5054 | SERPINE1 | serpin peptidase inhibitor, clade E (nexin, plasminogen activator inhibitor type 1), member 1 | -0.1034 | 0.7784 | 0.9711 | 5.294 | 5.8474 | 5.0985 | 5.5182 | 5.6556 | 4.9783 | 5.294 | 5.7166 | 5.5982 | 5.7835 | 6.0488 | 5.0263 |
| 200843_s_at | 2058 | EPRS | glutamyl-prolyl-tRNA synthetase | -0.1054 | 0.7066 | 0.9479 | 10.2024 | 10.2986 | 10.491 | 10.3927 | 10.1057 | 10.3927 | 10.5393 | 10.5015 | 10.0386 | 10.1489 | 10.2308 | 10.2308 |
| 211747_s_at | 23658 | LSM5 | LSM5 homolog, U6 small nuclear RNA associated (S. cerevisiae) | -0.106 | 0.8523 | 0.9716 | 12.4958 | 12.465 | 12.5889 | 12.5517 | 12.4097 | 9.2192 | 12.1067 | 12.2342 | 12.1067 | 12.465 | 12.465 | 12.3004 |
| 215945_s_at | 23321 | TRIM2 | tripartite motif containing 2 | -0.1065 | 0.7305 | 0.9655 | 8.6247 | 8.5324 | 8.7012 | 8.6476 | 9.4888 | 8.6171 | 8.4602 | 8.2128 | 9.9133 | 9.455 | 8.4612 | 8.5141 |
| 205811_at | 11232 | POLG2 | polymerase (DNA directed), gamma 2, accessory subunit | -0.1068 | 0.6527 | 0.9211 | 6.4515 | 7.7231 | 7.6234 | 5.7835 | 7.5692 | 7.4881 | 7.6742 | 7.5973 | 5.9915 | 7.5001 | 7.4889 | 7.0805 |
| 203575_at | 1459 | CSNK2A2 | casein kinase 2, alpha prime polypeptide | -0.107 | 0.7705 | 0.9698 | 8.8377 | 8.8561 | 8.7346 | 8.7346 | 8.6906 | 8.9622 | 8.9897 | 9.3333 | 8.9869 | 9.1265 | 8.446 | 8.633 |
| 203303_at | 6990 | DYNLT3 | dynein, light chain, Tctex-type 3 | -0.1077 | 0.8164 | 0.9716 | 7.4449 | 7.5973 | 7.6234 | 7.9704 | 7.7986 | 7.4255 | 7.4336 | 7.1528 | 7.4732 | 7.6666 | 7.657 | 7.1587 |
| 204000_at | 10681 | GNB5 | guanine nucleotide binding protein (G protein), beta 5 | -0.1085 | 0.7465 | 0.9683 | 5.9169 | 6.3138 | 6.051 | 6.0389 | 6.4053 | 6.2131 | 6.3872 | 5.938 | 5.8625 | 6.2691 | 6.1871 | 6.0073 |
| 208820_at | 5747 | PTK2 | PTK2 protein tyrosine kinase 2 | -0.1101 | 0.7545 | 0.9685 | 9.7997 | 10.8036 | 9.5117 | 10.8218 | 9.8495 | 9.8376 | 10.4772 | 9.9133 | 11.0543 | 9.9499 | 9.6875 | 9.7142 |
| 200622_x_at | 808 | CALM3 | calmodulin 3 (phosphorylase kinase, delta) | -0.1106 | 0.7126 | 0.9495 | 6.5365 | 6.8551 | 5.2393 | 6.2411 | 6.5993 | 6.7607 | 7.685 | 6.7607 | 7.49 | 6.4805 | 5.9813 | 6.5746 |
| 209364_at | 572 | BAD | BCL2-associated agonist of cell death | -0.1126 | 0.7565 | 0.9685 | 6.624 | 6.4268 | 6.4408 | 6.3308 | 6.5524 | 7.114 | 6.5301 | 6.7251 | 6.3575 | 6.7423 | 6.3809 | 7.0932 |
| 221539_at | 1978 | EIF4EBP1 | eukaryotic translation initiation factor 4E binding protein 1 | -0.1129 | 0.7405 | 0.9683 | 7.5337 | 7.685 | 7.8692 | 7.6612 | 8.1439 | 7.8786 | 7.7461 | 7.7535 | 7.6211 | 7.5266 | 7.9078 | 7.8094 |
| 206099_at | 5583 | PRKCH | protein kinase C, eta | -0.1137 | 0.6786 | 0.9361 | 5.1707 | 9.5009 | 5.1612 | 5.1982 | 4.9026 | 5.0985 | 5.0884 | 8.7574 | 5.0367 | 5.0107 | 5.116 | 8.5715 |
| 221641_s_at | 23597 | ACOT9 | acyl-CoA thioesterase 9 | -0.1139 | 0.7086 | 0.948 | 7.6442 | 7.7986 | 7.9882 | 8.0132 | 7.8928 | 7.6126 | 7.4732 | 8.0132 | 7.4003 | 7.5198 | 7.7772 | 7.6168 |
| 221531_at | 80349 | WDR61 | WD repeat domain 61 | -0.1155 | 0.6407 | 0.9161 | 9.2936 | 8.1475 | 8.7293 | 7.7856 | 7.594 | 9.0472 | 8.5919 | 8.1652 | 8.2735 | 8.8397 | 8.8263 | 8.581 |
| 205051_s_at | 3815 | KIT | v-kit Hardy-Zuckerman 4 feline sarcoma viral oncogene homolog | -0.1177 | 0.6707 | 0.933 | 5.9102 | 6.299 | 5.9873 | 6.0073 | 6.5894 | 7.3806 | 8.0943 | 7.6595 | 6.2011 | 5.5922 | 7.3307 | 7.872 |
| 202446_s_at | 5359 | PLSCR1 | phospholipid scramblase 1 | -0.1187 | 0.8782 | 0.9749 | 7.3841 | 7.5692 | 7.8155 | 7.8094 | 7.6005 | 7.4188 | 7.1768 | 7.461 | 7.3377 | 7.5337 | 7.5337 | 7.1114 |
| 203685_at | 596 | BCL2 | B-cell CLL/lymphoma 2 | -0.1195 | 0.6846 | 0.9391 | 5.5328 | 5.9102 | 5.7708 | 5.9592 | 5.7469 | 5.6619 | 6.7922 | 6.5127 | 6.0427 | 5.2037 | 5.9035 | 6.0914 |
| 201761_at | 10797 | MTHFD2 | methylenetetrahydrofolate dehydrogenase (NADP+ dependent) 2, methenyltetrahydrofolate cyclohydrolase | -0.1198 | 0.7066 | 0.9479 | 11.8924 | 11.8924 | 12.2244 | 11.9462 | 11.9863 | 11.9239 | 11.8756 | 12.2004 | 11.6409 | 12.1565 | 11.8563 | 11.9239 |
| 219492_at | 26511 | CHIC2 | cysteine-rich hydrophobic domain 2 | -0.1211 | 0.8643 | 0.9716 | 8.7079 | 8.6391 | 4.7565 | 8.6171 | 8.5096 | 8.3443 | 8.359 | 8.6135 | 8.446 | 8.7265 | 8.6562 | 8.2735 |
| 200768_s_at | 4144 | MAT2A | methionine adenosyltransferase II, alpha | -0.123 | 0.5908 | 0.8986 | 12.5517 | 12.3753 | 12.3753 | 12.4097 | 12.465 | 12.465 | 12.4958 | 12.3753 | 12.4511 | 12.4511 | 12.662 | 12.5517 |
| 202349_at | 1861 | TOR1A | torsin family 1, member A (torsin A) | -0.1244 | 0.6906 | 0.9421 | 5.2137 | 8.546 | 8.7822 | 8.4169 | 8.3408 | 8.3972 | 8.446 | 8.0672 | 8.2172 | 8.296 | 8.3972 | 8.1096 |
| 214086_s_at | 10038 | PARP2 | poly (ADP-ribose) polymerase 2 | -0.1258 | 0.7665 | 0.9685 | 9.7561 | 10.1184 | 10.0946 | 10.0094 | 5.7319 | 9.706 | 9.6908 | 10.1184 | 9.6827 | 9.9689 | 10.0612 | 9.8234 |
| 200602_at | 351 | APP | amyloid beta (A4) precursor protein | -0.1299 | 0.6946 | 0.9422 | 13.2279 | 13.363 | 13.2279 | 13.0626 | 13.2954 | 13.0626 | 13.2279 | 13.2279 | 13.2279 | 13.2279 | 13.2279 | 13.2279 |
| 205750_at | 670 | BPHL | biphenyl hydrolase-like (serine hydrolase) | -0.1318 | 0.7685 | 0.9698 | 7.6211 | 8.0874 | 7.6704 | 7.5528 | 7.7856 | 7.9661 | 7.7351 | 7.4255 | 10.3682 | 8.2633 | 8.7293 | 7.7535 |
| 205909_at | 5427 | POLE2 | polymerase (DNA directed), epsilon 2 (p59 subunit) | -0.1337 | 0.6208 | 0.9102 | 7.9071 | 8.8445 | 9.0383 | 8.9027 | 8.9454 | 8.7226 | 8.502 | 8.6517 | 8.4101 | 8.8182 | 9.1196 | 8.6808 |
| 215980_s_at | 3508 | IGHMBP2 | immunoglobulin mu binding protein 2 | -0.1343 | 0.8663 | 0.9716 | 6.1187 | 5.9009 | 5.736 | 5.831 | 5.7166 | 5.5548 | 5.5378 | 6.0078 | 5.6716 | 5.5446 | 8.5715 | 5.4195 |
| 206752_s_at | 1677 | DFFB | DNA fragmentation factor, 40kDa, beta polypeptide (caspase-activated DNase) | -0.1345 | 0.7206 | 0.9562 | 7.0745 | 7.4033 | 7.2641 | 7.0775 | 7.1528 | 6.9757 | 7.2032 | 7.1877 | 7.0851 | 7.2559 | 7.4715 | 7.1768 |
| 203218_at | 5601 | MAPK9 | mitogen-activated protein kinase 9 | -0.1351 | 0.5968 | 0.8996 | 10.045 | 10.4499 | 9.7285 | 10.3469 | 10.2552 | 10.4318 | 10.5113 | 10.6469 | 10.0005 | 10.584 | 10.5646 | 10.6154 |
| 217080_s_at | 9455 | HOMER2 | homer homolog 2 (Drosophila) | -0.1357 | 0.6407 | 0.9161 | 7.6629 | 6.6032 | 7.2118 | 7.1703 | 7.1088 | 6.5127 | 7.7198 | 7.5805 | 7.5897 | 7.795 | 6.7289 | 6.4991 |
| 210038_at | 5588 | PRKCQ | protein kinase C, theta | -0.1378 | 0.99 | 0.9951 | 6.7607 | 7.2091 | 7.2304 | 7.1703 | 6.8157 | 6.6276 | 7.1474 | 6.8082 | 6.8842 | 6.727 | 6.9591 | 6.7423 |
| 218450_at | 50865 | HEBP1 | heme binding protein 1 | -0.1386 | 0.6128 | 0.9066 | 8.0384 | 7.872 | 8.2216 | 7.9327 | 7.8826 | 6.8468 | 7.6442 | 7.044 | 7.8826 | 7.0843 | 8.0781 | 8.261 |
| 204999_s_at | 22809 | ATF5 | activating transcription factor 5 | -0.1387 | 0.5549 | 0.8876 | 7.7617 | 7.9445 | 7.9661 | 8.654 | 7.9661 | 7.9783 | 8.4049 | 7.7231 | 8.7913 | 7.9071 | 7.7072 | 8.4664 |
| 209380_s_at | 10057 | ABCC5 | ATP-binding cassette, sub-family C (CFTR/MRP), member 5 | -0.1389 | 0.9661 | 0.9921 | 5.7469 | 5.7242 | 5.803 | 5.938 | 5.6937 | 5.8904 | 5.3875 | 5.432 | 7.7968 | 5.3008 | 5.607 | 5.6326 |
| 219051_x_at | 79006 | METRN | meteorin, glial cell differentiation regulator | -0.1394 | 0.5449 | 0.8853 | 5.432 | 5.9693 | 5.8983 | 5.9035 | 5.8045 | 8.7293 | 5.7017 | 5.6276 | 9.0193 | 5.9813 | 5.7514 | 5.6633 |
| 201266_at | 7296 | TXNRD1 | thioredoxin reductase 1 | -0.142 | 0.5569 | 0.8876 | 9.7719 | 9.4092 | 9.2686 | 9.3908 | 9.6908 | 8.9748 | 9.3515 | 9.7373 | 9.3433 | 9.5414 | 9.4254 | 9.3209 |
| 219222_at | 64080 | RBKS | ribokinase | -0.1433 | 0.98 | 0.9921 | 4.9864 | 5.0618 | 5.4195 | 5.1023 | 5.2837 | 5.432 | 5.2037 | 4.8949 | 5.1062 | 4.8362 | 5.0263 | 5.0692 |
| 203565_s_at | 4331 | MNAT1 | menage a trois homolog 1, cyclin H assembly factor (Xenopus laevis) | -0.1439 | 0.6487 | 0.9195 | 8.2259 | 8.1402 | 9.4888 | 9.3433 | 8.5388 | 8.5966 | 9.1601 | 8.7157 | 8.3224 | 8.3822 | 9.2633 | 8.4172 |
| 202743_at | 8503 | PIK3R3 | phosphoinositide-3-kinase, regulatory subunit 3 (gamma) | -0.1449 | 0.6627 | 0.9298 | 5.3682 | 5.3085 | 5.3008 | 5.223 | 5.5378 | 5.5514 | 5.7423 | 5.5378 | 5.166 | 5.432 | 5.3457 | 5.8904 |
| 201817_at | 9690 | UBE3C | ubiquitin protein ligase E3C | -0.1459 | 0.6248 | 0.9127 | 8.1521 | 7.9974 | 9.0859 | 9.0383 | 7.9512 | 8.1348 | 8.8397 | 9.1512 | 7.6126 | 9.109 | 8.1103 | 8.546 |
| 208728_s_at | 998 | CDC42 | cell division cycle 42 (GTP binding protein, 25kDa) | -0.1472 | 0.7405 | 0.9683 | 12.3234 | 12.3455 | 12.4511 | 12.465 | 12.1301 | 8.231 | 12.1565 | 12.2771 | 12.1565 | 12.3234 | 12.3455 | 12.3234 |
| 218168_s_at | 56997 | ADCK3 | aarF domain containing kinase 3 | -0.1476 | 0.9182 | 0.9845 | 4.3929 | 4.1341 | 4.5509 | 4.151 | 4.2682 | 4.4649 | 3.955 | 4.1172 | 4.356 | 4.2311 | 4.0871 | 3.9141 |
| 203648_at | 9797 | TATDN2 | TatD DNase domain containing 2 | -0.1482 | 0.9042 | 0.9825 | 5.6326 | 5.3609 | 5.5446 | 5.6175 | 5.3457 | 5.0692 | 5.3503 | 5.473 | 5.1384 | 5.3131 | 5.2278 | 5.2837 |
| 200081_s_at | 6194 | RPS6 | ribosomal protein S6 | -0.1499 | 0.5409 | 0.8817 | 14.0921 | 14.0921 | 14.0921 | 13.6371 | 14.0921 | 14.0921 | 14.3602 | 14.0921 | 13.9353 | 13.9353 | 13.9353 | 14.0921 |
| 201697_s_at | 1786 | DNMT1 | DNA (cytosine-5-)-methyltransferase 1 | -0.15 | 0.5669 | 0.8876 | 12.3753 | 12.3234 | 12.3234 | 12.3455 | 12.3753 | 12.7071 | 12.5889 | 12.5889 | 12.7591 | 12.8313 | 12.9163 | 12.662 |
| 201841_s_at | 3315 | HSPB1 | heat shock 27kDa protein 1 | -0.1523 | 0.5828 | 0.8951 | 7.4911 | 8.0938 | 8.1263 | 7.3881 | 8.1808 | 8.7265 | 7.2733 | 7.7351 | 7.7351 | 8.0672 | 8.1918 | 8.167 |
| 201032_at | 10904 | BLCAP | bladder cancer associated protein | -0.1528 | 0.5689 | 0.8876 | 8.7155 | 9.032 | 9.6092 | 8.546 | 9.3908 | 9.0859 | 9.0023 | 9.4219 | 9.2204 | 8.8128 | 9.1386 | 9.0023 |
| 34408_at | 6253 | RTN2 | reticulon 2 | -0.1551 | 0.5489 | 0.8873 | 9.5376 | 8.9136 | 8.7191 | 8.6688 | 8.9748 | 9.3685 | 10.0005 | 9.1143 | 9.9689 | 9.8234 | 9.5537 | 10.0321 |
| 207540_s_at | 6850 | SYK | spleen tyrosine kinase | -0.1564 | 0.5649 | 0.8876 | 6.3975 | 6.3872 | 6.2057 | 6.0155 | 6.299 | 6.731 | 5.9693 | 6.299 | 6.413 | 6.9214 | 6.6204 | 5.7514 |
| 220127_s_at | 54850 | FBXL12 | F-box and leucine-rich repeat protein 12 | -0.1564 | 0.5768 | 0.8941 | 8.502 | 7.358 | 8.0943 | 7.4033 | 7.1397 | 7.0474 | 7.5337 | 8.7822 | 7.7351 | 7.7231 | 8.7226 | 8.8397 |
| 207163_s_at | 207 | AKT1 | v-akt murine thymoma viral oncogene homolog 1 | -0.1567 | 0.9062 | 0.9825 | 8.1402 | 8.3972 | 8.4339 | 8.6025 | 8.321 | 9.0193 | 8.3623 | 8.3516 | 7.9244 | 8.3857 | 8.2128 | 8.3656 |
| 201913_s_at | 80347 | COASY | CoA synthase | -0.1573 | 0.5409 | 0.8817 | 6.0155 | 5.7925 | 6.0004 | 6.8636 | 6.3895 | 6.5746 | 6.4669 | 6.1533 | 6.2911 | 5.641 | 6.3388 | 6.051 |
| 203726_s_at | 3909 | LAMA3 | laminin, alpha 3 | -0.1586 | 0.9501 | 0.9921 | 3.955 | 4.3929 | 4.5098 | 4.3799 | 4.4486 | 4.5225 | 4.2034 | 4.2682 | 8.0104 | 4.2996 | 4.052 | 3.7365 |
| 204711_at | 9851 | KIAA0753 | KIAA0753 | -0.1586 | 0.5808 | 0.8951 | 7.1088 | 7.9071 | 6.9462 | 6.9475 | 6.6638 | 7.7413 | 6.5942 | 7.6211 | 6.7968 | 7.0932 | 6.8922 | 7.8928 |
| 209337_at | 11168 | PSIP1 | PC4 and SFRS1 interacting protein 1 | -0.1588 | 0.521 | 0.8768 | 9.3022 | 9.6827 | 7.358 | 9.4483 | 7.4188 | 9.0263 | 9.5597 | 9.6038 | 6.2131 | 9.4345 | 9.4483 | 9.2323 |
| 203233_at | 3566 | IL4R | interleukin 4 receptor | -0.1593 | 0.5529 | 0.8876 | 7.0144 | 6.2911 | 7.0954 | 6.0004 | 5.7242 | 6.4805 | 6.6781 | 6.9591 | 7.358 | 6.9591 | 6.5355 | 6.7758 |
| 204451_at | 8321 | FZD1 | frizzled family receptor 1 | -0.1629 | 0.5529 | 0.8876 | 6.3919 | 6.2691 | 7.3223 | 6.3919 | 6.5054 | 6.2411 | 7.3307 | 6.4929 | 6.7904 | 7.3021 | 7.0859 | 7.241 |
| 208908_s_at | 831 | CAST | calpastatin | -0.164 | 0.521 | 0.8768 | 9.4219 | 9.0397 | 8.7978 | 9.0088 | 8.8935 | 8.78 | 8.8263 | 8.8492 | 11.353 | 9.7285 | 10.2552 | 9.1265 |
| 201671_x_at | 9097 | USP14 | ubiquitin specific peptidase 14 (tRNA-guanine transglycosylase) | -0.1647 | 0.5569 | 0.8876 | 6.8907 | 7.2524 | 7.5564 | 7.1528 | 7.436 | 7.6742 | 7.3188 | 7.9623 | 7.1528 | 7.2247 | 7.4033 | 7.5506 |
| 201984_s_at | 1956 | EGFR | epidermal growth factor receptor | -0.1673 | 0.6287 | 0.9127 | 4.9937 | 4.8576 | 4.8822 | 4.9229 | 4.9353 | 4.8362 | 5.3085 | 4.972 | 4.612 | 4.6373 | 4.7924 | 4.65 |
| 217872_at | 55011 | PIH1D1 | PIH1 domain containing 1 | -0.1684 | 0.7764 | 0.9711 | 7.2836 | 6.7968 | 6.9296 | 7.1215 | 6.873 | 7.1681 | 6.8236 | 7.0229 | 7.0229 | 6.9965 | 7.1528 | 6.8082 |
| 203616_at | 5423 | POLB | polymerase (DNA directed), beta | -0.1715 | 0.6307 | 0.9127 | 7.7522 | 7.8155 | 8.4166 | 7.685 | 7.6485 | 7.8326 | 7.249 | 7.7351 | 7.4688 | 7.8326 | 7.9768 | 7.9623 |
| 219082_at | 51005 | AMDHD2 | amidohydrolase domain containing 2 | -0.1721 | 0.489 | 0.8692 | 6.0914 | 5.7017 | 6.5968 | 6.217 | 6.4991 | 6.4307 | 6.2473 | 6.2034 | 6.1865 | 6.475 | 6.0544 | 6.5355 |
| 218961_s_at | 11284 | PNKP | polynucleotide kinase 3'-phosphatase | -0.1731 | 0.9741 | 0.9921 | 6.9712 | 6.9123 | 6.7423 | 7.1835 | 7.2601 | 7.1746 | 6.836 | 6.7472 | 6.9296 | 6.7758 | 6.6032 | 6.9098 |
| 205130_at | 5891 | MOK | MOK protein kinase | -0.1734 | 0.5569 | 0.8876 | 5.5982 | 5.7242 | 6.5245 | 5.4553 | 5.5182 | 5.8904 | 5.4947 | 5.1707 | 5.7204 | 5.5698 | 6.9298 | 5.6606 |
| 219270_at | 79094 | CHAC1 | ChaC, cation transport regulator homolog 1 (E. coli) | -0.1769 | 0.525 | 0.8768 | 8.5217 | 7.9566 | 8.296 | 8.2423 | 9.7249 | 9.6129 | 10.4396 | 9.5414 | 8.4339 | 8.2067 | 8.0728 | 9.7015 |
| 206254_at | 1950 | EGF | epidermal growth factor | -0.179 | 0.5729 | 0.8893 | 5.3682 | 4.9477 | 4.8469 | 5.0412 | 5.2182 | 4.7983 | 4.9864 | 4.972 | 5.4195 | 5.2037 | 4.9937 | 5.2278 |
| 208407_s_at | 1500 | CTNND1 | catenin (cadherin-associated protein), delta 1 | -0.1791 | 0.4511 | 0.8324 | 8.3408 | 7.7617 | 7.4732 | 7.6545 | 7.5761 | 7.7198 | 9.6129 | 8.4835 | 9.2437 | 8.4049 | 8.8613 | 9.3908 |
| 205899_at | 8900 | CCNA1 | cyclin A1 | -0.1823 | 0.7884 | 0.9711 | 5.5664 | 5.641 | 5.8589 | 5.8589 | 6.1155 | 5.5378 | 5.6606 | 5.7835 | 5.4862 | 5.8148 | 6.0231 | 7.6257 |
| 201087_at | 5829 | PXN | paxillin | -0.1828 | 0.491 | 0.8692 | 9.3568 | 9.8495 | 8.9351 | 9.1665 | 10.1184 | 10.8682 | 11.9984 | 10.2832 | 11.5842 | 10.7415 | 11.0543 | 9.9658 |
| 202716_at | 5770 | PTPN1 | protein tyrosine phosphatase, non-receptor type 1 | -0.1833 | 0.525 | 0.8768 | 8.6983 | 8.6562 | 8.1103 | 8.2633 | 8.9175 | 8.7781 | 8.6808 | 8.9136 | 8.9748 | 8.6391 | 8.6497 | 8.5852 |
| 217825_s_at | 51465 | UBE2J1 | ubiquitin-conjugating enzyme E2, J1, U | -0.1842 | 0.519 | 0.8768 | 8.1918 | 7.0859 | 6.7107 | 7.6781 | 7.5546 | 7.2621 | 8.6639 | 7.1364 | 8.6663 | 8.6476 | 7.657 | 7.9071 |
| 213150_at | 3206 | HOXA10 | homeobox A10 | -0.1842 | 0.517 | 0.8768 | 10.8682 | 10.6579 | 10.5015 | 10.4689 | 10.7518 | 10.6038 | 10.3258 | 10.9321 | 10.6622 | 10.7025 | 10.8682 | 10.7518 |
| 203639_s_at | 2263 | FGFR2 | fibroblast growth factor receptor 2 | -0.1843 | 0.5549 | 0.8876 | 6.8597 | 6.5676 | 5.7708 | 6.1187 | 6.6562 | 5.7879 | 6.7922 | 6.4053 | 8.3408 | 7.1215 | 7.1768 | 6.9556 |
| 208398_s_at | 9519 | TBPL1 | TBP-like 1 | -0.1851 | 0.523 | 0.8768 | 8.4735 | 8.1608 | 8.4002 | 8.9748 | 9.0023 | 8.7574 | 8.6808 | 8.2018 | 8.801 | 8.3972 | 9.0574 | 8.0321 |
| 202604_x_at | 102 | ADAM10 | ADAM metallopeptidase domain 10 | -0.1856 | 0.9661 | 0.9921 | 8.2886 | 8.4602 | 8.8397 | 8.446 | 8.6562 | 8.5919 | 8.524 | 8.4622 | 8.4338 | 8.2519 | 8.1402 | 8.028 |
| 217789_at | 58533 | SNX6 | sorting nexin 6 | -0.1866 | 0.5828 | 0.8951 | 9.4483 | 9.3515 | 9.6247 | 9.2633 | 9.4414 | 9.3768 | 9.3768 | 9.3308 | 9.1906 | 9.3099 | 9.2995 | 9.2437 |
| 211715_s_at | 622 | BDH1 | 3-hydroxybutyrate dehydrogenase, type 1 | -0.1875 | 0.5928 | 0.8989 | 4.9026 | 5.1828 | 5.3085 | 4.8262 | 5.2278 | 4.5613 | 5.4123 | 4.8613 | 5.001 | 5.4992 | 4.9864 | 5.0884 |
| 211919_s_at | 7852 | CXCR4 | chemokine (C-X-C motif) receptor 4 | -0.188 | 0.6228 | 0.9118 | 7.2032 | 7.8967 | 7.499 | 7.2967 | 7.3914 | 8.1652 | 8.4166 | 8.0384 | 7.3009 | 7.3009 | 7.6429 | 7.6704 |
| 202949_s_at | 2274 | FHL2 | four and a half LIM domains 2 | -0.1896 | 0.5369 | 0.8817 | 7.4873 | 7.114 | 7.3292 | 7.5198 | 7.436 | 6.9922 | 7.1331 | 6.9865 | 7.6761 | 7.4435 | 7.7867 | 7.4942 |
| 201631_s_at | 8870 | IER3 | immediate early response 3 | -0.1921 | 0.507 | 0.8748 | 5.609 | 5.7835 | 6.8703 | 6.6183 | 6.0877 | 6.0683 | 6.607 | 6.2411 | 6.1727 | 6.4478 | 6.0359 | 5.803 |
| 204679_at | 3775 | KCNK1 | potassium channel, subfamily K, member 1 | -0.1935 | 0.495 | 0.8692 | 5.8783 | 5.8825 | 9.9373 | 5.9035 | 6.1092 | 5.3457 | 9.2633 | 5.3755 | 8.8935 | 5.5446 | 8.9574 | 9.1906 |
| 213292_s_at | 23161 | SNX13 | sorting nexin 13 | -0.1936 | 0.5469 | 0.887 | 8.5732 | 8.5141 | 7.795 | 8.0157 | 8.4002 | 8.5852 | 8.8096 | 8.0555 | 9.954 | 8.9897 | 8.9415 | 8.4338 |
| 200886_s_at | 5223 | PGAM1 | phosphoglycerate mutase 1 (brain) | -0.1943 | 0.6028 | 0.9003 | 12.1763 | 12.3004 | 12.2342 | 12.2771 | 12.2342 | 12.5517 | 12.3004 | 12.4097 | 12.2244 | 12.1763 | 12.3234 | 12.2342 |
| 207238_s_at | 5788 | PTPRC | protein tyrosine phosphatase, receptor type, C | -0.1949 | 0.4172 | 0.8166 | 6.8907 | 6.0683 | 7.8401 | 6.4441 | 5.9009 | 7.2559 | 7.5013 | 7.6257 | 7.2064 | 7.5546 | 7.3841 | 7.6429 |
| 202388_at | 5997 | RGS2 | regulator of G-protein signaling 2, 24kDa | -0.1961 | 0.6028 | 0.9003 | 6.3706 | 6.0073 | 6.3975 | 6.4361 | 6.3388 | 5.8338 | 6.157 | 5.9795 | 6.0847 | 6.2326 | 6.2011 | 6.0329 |
| 202119_s_at | 8895 | CPNE3 | copine III | -0.1964 | 0.521 | 0.8768 | 11.8563 | 11.7318 | 11.9863 | 11.7662 | 11.6864 | 11.809 | 11.6409 | 11.8563 | 11.7662 | 11.9984 | 11.4966 | 11.7843 |
| 202732_at | 11142 | PKIG | protein kinase (cAMP-dependent, catalytic) inhibitor gamma | -0.1971 | 0.5928 | 0.8989 | 7.5761 | 7.5973 | 8.0555 | 7.848 | 7.795 | 9.2995 | 8.3443 | 8.9841 | 7.872 | 7.8826 | 7.9704 | 7.9728 |
| 209588_at | 2048 | EPHB2 | EPH receptor B2 | -0.1975 | 0.8703 | 0.9716 | 6.0004 | 5.244 | 5.8715 | 5.5514 | 5.5664 | 5.609 | 5.8983 | 5.6716 | 5.3933 | 5.7242 | 5.6226 | 5.4553 |
| 221779_at | 85377 | MICALL1 | MICAL-like 1 | -0.1982 | 0.7106 | 0.9494 | 5.452 | 5.3933 | 5.9102 | 5.3755 | 5.8045 | 6.0038 | 5.6276 | 5.7278 | 5.1948 | 5.4553 | 5.5278 | 5.5278 |
| 205379_at | 874 | CBR3 | carbonyl reductase 3 | -0.199 | 0.499 | 0.873 | 5.736 | 4.9657 | 4.8651 | 5.4641 | 5.6109 | 5.0204 | 4.8042 | 5.5982 | 5.831 | 4.7839 | 6.0155 | 5.7925 |
| 204825_at | 9833 | MELK | maternal embryonic leucine zipper kinase | -0.2003 | 0.6687 | 0.9329 | 10.0005 | 9.9373 | 9.955 | 9.9759 | 9.4264 | 9.8234 | 9.4305 | 9.9759 | 9.5376 | 9.9499 | 9.7997 | 9.995 |
| 221230_s_at | 51742 | ARID4B | AT rich interactive domain 4B (RBP1-like) | -0.2013 | 0.495 | 0.8692 | 7.9661 | 8.0375 | 7.6429 | 8.028 | 7.5546 | 7.8165 | 8.272 | 7.4942 | 7.4973 | 8.3723 | 7.5198 | 8.5869 |
| 201036_s_at | 3033 | HADH | hydroxyacyl-CoA dehydrogenase | -0.2015 | 0.4351 | 0.8275 | 9.1265 | 8.8182 | 9.032 | 9.032 | 8.2259 | 8.0219 | 8.7265 | 7.8144 | 8.7226 | 9.0023 | 8.7683 | 8.9136 |
| 203465_at | 9801 | MRPL19 | mitochondrial ribosomal protein L19 | -0.202 | 0.4411 | 0.8296 | 10.4772 | 10.5015 | 10.4499 | 10.1057 | 10.3927 | 10.2986 | 10.4579 | 10.5113 | 10.5248 | 10.4772 | 10.5765 | 10.4579 |
| 212851_at | 23142 | DCUN1D4 | DCN1, defective in cullin neddylation 1, domain containing 4 (S. cerevisiae) | -0.2028 | 0.5289 | 0.8768 | 7.8948 | 7.7986 | 8.0517 | 7.9783 | 7.6545 | 8.1402 | 8.7226 | 8.0104 | 7.8144 | 8.0145 | 8.0982 | 8.7293 |
| 200783_s_at | 3925 | STMN1 | stathmin 1 | -0.2029 | 0.4491 | 0.8324 | 9.6247 | 9.776 | 9.6092 | 9.863 | 9.8129 | 9.4768 | 9.8234 | 10.0593 | 8.8096 | 9.6247 | 9.8849 | 10.1782 |
| 202052_s_at | 26064 | RAI14 | retinoic acid induced 14 | -0.2038 | 0.5409 | 0.8817 | 9.9133 | 9.6648 | 9.3187 | 9.2192 | 9.8445 | 9.9689 | 9.7839 | 9.4483 | 11.3003 | 10.1416 | 10.4396 | 9.9421 |
| 205205_at | 5971 | RELB | v-rel reticuloendotheliosis viral oncogene homolog B | -0.2045 | 0.98 | 0.9921 | 5.8589 | 5.8625 | 6.1242 | 6.0847 | 5.5278 | 5.4641 | 5.5922 | 5.6716 | 5.7204 | 5.8474 | 6.1092 | 5.3503 |
| 218034_at | 51024 | FIS1 | fission 1 (mitochondrial outer membrane) homolog (S. cerevisiae) | -0.2063 | 0.507 | 0.8748 | 7.2559 | 6.2758 | 6.5245 | 6.3433 | 7.1681 | 6.2326 | 6.4832 | 6.1957 | 6.5245 | 6.496 | 6.7205 | 6.5054 |
| 202910_s_at | 976 | CD97 | CD97 molecule | -0.2107 | 0.7246 | 0.9589 | 5.9559 | 5.9211 | 6.1155 | 6.0847 | 5.9102 | 5.8589 | 6.0078 | 11.4763 | 5.6977 | 5.9915 | 5.7748 | 5.9795 |
| 207042_at | 1870 | E2F2 | E2F transcription factor 2 | -0.2127 | 0.9202 | 0.9845 | 5.1023 | 5.1062 | 5.0786 | 5.7017 | 5.3755 | 5.7928 | 4.9567 | 5.2336 | 5.2689 | 5.4641 | 5.2837 | 4.8651 |
| 212910_at | 57215 | THAP11 | THAP domain containing 11 | -0.215 | 0.4491 | 0.8324 | 7.3377 | 7.2925 | 7.4336 | 7.1397 | 7.3292 | 7.3841 | 7.241 | 7.2641 | 7.6442 | 7.0697 | 7.5446 | 7.249 |
| 207196_s_at | 10318 | TNIP1 | TNFAIP3 interacting protein 1 | -0.2156 | 0.3832 | 0.7956 | 5.0107 | 4.9864 | 6.5127 | 6.2473 | 5.0835 | 6.0544 | 5.7835 | 5.1707 | 6.1124 | 6.1488 | 6.3247 | 5.1494 |
| 202708_s_at | 8349 | HIST2H2BE | histone cluster 2, H2be | -0.216 | 0.5808 | 0.8951 | 6.299 | 6.095 | 5.6226 | 5.8338 | 5.6326 | 5.7104 | 5.5732 | 5.6937 | 7.2 | 6.6183 | 6.8978 | 6.2411 |
| 212614_at | 84159 | ARID5B | AT rich interactive domain 5B (MRF1-like) | -0.2207 | 0.6407 | 0.9161 | 6.4894 | 6.3975 | 6.3411 | 6.4014 | 5.9744 | 6.1865 | 6.299 | 6.2758 | 6.8157 | 6.4567 | 6.4567 | 6.1444 |
| 200816_s_at | 5048 | PAFAH1B1 | platelet-activating factor acetylhydrolase 1b, regulatory subunit 1 (45kDa) | -0.2213 | 0.493 | 0.8692 | 9.1143 | 8.8894 | 9.0411 | 8.7913 | 8.8527 | 8.7945 | 9.5363 | 9.3768 | 8.6882 | 8.9432 | 8.7945 | 9.2876 |
| 203521_s_at | 24149 | ZNF318 | zinc finger protein 318 | -0.2213 | 0.4491 | 0.8324 | 7.2396 | 8.3158 | 8.261 | 9.1265 | 10.5015 | 10.584 | 12.0594 | 11.3003 | 8.3723 | 8.3197 | 10.9381 | 12.3753 |
| 210145_at | 5321 | PLA2G4A | phospholipase A2, group IVA (cytosolic, calcium-dependent) | -0.2219 | 0.481 | 0.8616 | 10.1489 | 9.2316 | 7.9704 | 7.9945 | 8.1521 | 8.446 | 9.6056 | 8.5388 | 10.0573 | 10.1184 | 10.1602 | 9.6092 |
| 210761_s_at | 2886 | GRB7 | growth factor receptor-bound protein 7 | -0.2234 | 0.523 | 0.8768 | 10.5113 | 10.5327 | 9.0733 | 9.5496 | 9.5814 | 11.5374 | 10.4499 | 9.537 | 11.2078 | 12.5517 | 12.7952 | 10.6326 |
| 211725_s_at | 637 | BID | BH3 interacting domain death agonist | -0.2236 | 0.489 | 0.8692 | 5.2137 | 6.4832 | 5.3176 | 5.5664 | 6.4441 | 5.3457 | 5.3875 | 6.282 | 7.4055 | 6.7607 | 6.6183 | 6.3947 |
| 203360_s_at | 26292 | MYCBP | c-myc binding protein | -0.2248 | 0.3792 | 0.7942 | 11.3665 | 11.0907 | 11.4472 | 11.2801 | 11.353 | 11.353 | 11.1291 | 11.3348 | 11.0744 | 11.4966 | 11.4048 | 11.3003 |
| 204788_s_at | 5498 | PPOX | protoporphyrinogen oxidase | -0.233 | 0.6048 | 0.9003 | 9.455 | 8.7574 | 8.1652 | 8.5665 | 8.115 | 8.5506 | 8.8128 | 8.5096 | 10.7811 | 10.6225 | 9.6038 | 9.0733 |
| 218581_at | 63874 | ABHD4 | abhydrolase domain containing 4 | -0.233 | 0.992 | 0.9961 | 5.5863 | 5.4195 | 5.7017 | 5.5982 | 5.1965 | 5.6507 | 9.5597 | 5.1062 | 5.0367 | 4.9864 | 5.2689 | 5.3682 |
| 205161_s_at | 8800 | PEX11A | peroxisomal biogenesis factor 11 alpha | -0.2347 | 0.5908 | 0.8986 | 5.1384 | 5.0322 | 5.2092 | 4.9477 | 5.001 | 5.0786 | 5.2393 | 4.7924 | 5.3503 | 4.8651 | 5.1982 | 5.001 |
| 204256_at | 79071 | ELOVL6 | ELOVL fatty acid elongase 6 | -0.2349 | 0.4192 | 0.8166 | 7.1725 | 7.1228 | 7.4715 | 7.0775 | 6.9556 | 7.3609 | 7.2601 | 7.461 | 7.1012 | 7.3307 | 7.2142 | 7.3806 |
| 203285_s_at | 9653 | HS2ST1 | heparan sulfate 2-O-sulfotransferase 1 | -0.2366 | 0.4012 | 0.809 | 6.4578 | 7.0284 | 6.9865 | 6.9591 | 6.475 | 6.645 | 7.1012 | 7.0333 | 6.873 | 7.0284 | 6.9965 | 7.0851 |
| 202500_at | 3300 | DNAJB2 | DnaJ (Hsp40) homolog, subfamily B, member 2 | -0.2383 | 0.9062 | 0.9825 | 7.2247 | 7.1421 | 7.2064 | 7.2383 | 7.0284 | 7.5897 | 7.1088 | 7.105 | 6.8907 | 9.863 | 6.9757 | 10.1385 |
| 202458_at | 11098 | PRSS23 | protease, serine, 23 | -0.2397 | 0.4072 | 0.8101 | 6.4441 | 6.4929 | 6.3187 | 6.5474 | 6.3975 | 5.9592 | 6.5159 | 6.2691 | 6.5867 | 6.486 | 6.2473 | 6.5301 |
| 207034_s_at | 2736 | GLI2 | GLI family zinc finger 2 | -0.2412 | 0.5669 | 0.8876 | 5.0692 | 5.4947 | 4.9103 | 5.5278 | 5.2488 | 5.7879 | 6.0155 | 5.3047 | 5.7748 | 5.4992 | 4.9353 | 5.736 |
| 202423_at | 7994 | KAT6A | K(lysine) acetyltransferase 6A | -0.2416 | 0.4192 | 0.8166 | 8.6601 | 8.6025 | 8.7457 | 8.3158 | 9.2936 | 9.1143 | 8.9574 | 8.9027 | 8.4622 | 9.1962 | 9.1934 | 8.4501 |
| 201040_at | 2771 | GNAI2 | guanine nucleotide binding protein (G protein), alpha inhibiting activity polypeptide 2 | -0.2451 | 0.3752 | 0.7909 | 4.9657 | 4.612 | 4.8469 | 4.4955 | 4.8822 | 4.4407 | 4.3929 | 4.9103 | 4.9166 | 4.685 | 4.4407 | 5.2689 |
| 218457_s_at | 1788 | DNMT3A | DNA (cytosine-5-)-methyltransferase 3 alpha | -0.2452 | 0.3812 | 0.7956 | 8.6768 | 8.6729 | 7.3806 | 8.8671 | 7.8094 | 7.836 | 7.8826 | 7.5357 | 8.6729 | 9.0193 | 8.9841 | 8.8935 |
| 202868_s_at | 10775 | POP4 | processing of precursor 4, ribonuclease P/MRP subunit (S. cerevisiae) | -0.2476 | 0.3693 | 0.7868 | 8.6768 | 9.1036 | 9.1315 | 8.7683 | 9.0193 | 9.1512 | 8.8894 | 9.1962 | 8.4501 | 8.9454 | 9.0983 | 8.9622 |
| 202950_at | 1429 | CRYZ | crystallin, zeta (quinone reductase) | -0.2483 | 0.3733 | 0.7884 | 8.2886 | 8.1021 | 8.3408 | 8.3822 | 8.557 | 7.6005 | 7.9595 | 7.8877 | 8.3972 | 7.685 | 8.5665 | 8.2633 |
| 209263_x_at | 7106 | TSPAN4 | tetraspanin 4 | -0.2484 | 0.4331 | 0.8257 | 6.0231 | 5.6619 | 6.0193 | 6.6502 | 6.9025 | 6.295 | 6.5894 | 5.706 | 7.1877 | 6.3138 | 5.8589 | 6.519 |
| 201453_x_at | 6009 | RHEB | Ras homolog enriched in brain | -0.2488 | 0.4092 | 0.8101 | 12.2771 | 12.2004 | 11.9984 | 12.2244 | 12.3004 | 12.2342 | 12.019 | 12.019 | 12.2771 | 12.3004 | 12.2342 | 12.2244 |
| 201391_at | 10131 | TRAP1 | TNF receptor-associated protein 1 | -0.2495 | 0.3892 | 0.7964 | 8.9295 | 9.3568 | 9.5414 | 8.9398 | 9.1906 | 10.0946 | 9.0983 | 9.6171 | 9.2079 | 9.0733 | 9.3187 | 9.6092 |
| 205417_s_at | 1605 | DAG1 | dystroglycan 1 (dystrophin-associated glycoprotein 1) | -0.2497 | 0.4152 | 0.8166 | 4.3669 | 4.5808 | 4.5716 | 4.4147 | 4.3164 | 4.1172 | 4.151 | 4.8161 | 4.4147 | 4.1172 | 4.6951 | 4.3929 |
| 202550_s_at | 9217 | VAPB | VAMP (vesicle-associated membrane protein)-associated protein B and C | -0.2518 | 0.4671 | 0.8459 | 6.0078 | 6.0004 | 6.0683 | 6.1425 | 6.0818 | 6.1289 | 5.9813 | 6.095 | 6.2789 | 6.3758 | 6.095 | 5.8411 |
| 336_at | 6915 | TBXA2R | thromboxane A2 receptor | -0.2538 | 0.6347 | 0.9129 | 7.2641 | 7.2343 | 7.3377 | 7.1215 | 7.2167 | 7.4055 | 10.045 | 7.2167 | 8.3857 | 7.5854 | 7.5244 | 6.8338 |
| 203530_s_at | 6810 | STX4 | syntaxin 4 | -0.256 | 0.511 | 0.8767 | 8.4555 | 7.9822 | 7.7133 | 7.7811 | 7.6938 | 7.8575 | 7.7351 | 7.8745 | 9.0581 | 8.1881 | 8.4985 | 7.9208 |
| 203130_s_at | 3800 | KIF5C | kinesin family member 5C | -0.2561 | 0.9641 | 0.9921 | 6.4346 | 6.794 | 6.4408 | 6.5676 | 6.1406 | 6.3433 | 6.289 | 6.3758 | 10.7025 | 6.0231 | 6.0847 | 6.0718 |
| 218102_at | 51071 | DERA | deoxyribose-phosphate aldolase (putative) | -0.259 | 0.3214 | 0.743 | 9.0733 | 8.8096 | 6.7967 | 8.8397 | 8.9027 | 8.581 | 9.0765 | 8.7079 | 9.1315 | 8.9574 | 9.0472 | 8.8748 |
| 202720_at | 26136 | TES | testis derived transcript (3 LIM domains) | -0.2617 | 0.2635 | 0.7094 | 10.9381 | 6.3975 | 10.4357 | 10.6716 | 10.9929 | 10.9321 | 12.465 | 11.4048 | 12.3004 | 11.3911 | 11.6864 | 11.8924 |
| 209576_at | 2770 | GNAI1 | guanine nucleotide binding protein (G protein), alpha inhibiting activity polypeptide 1 | -0.2644 | 0.8164 | 0.9716 | 5.2488 | 5.4123 | 5.5606 | 5.4992 | 5.3085 | 5.2763 | 5.1612 | 5.2589 | 4.9937 | 7.8826 | 5.3176 | 5.0985 |
| 200830_at | 5708 | PSMD2 | proteasome (prosome, macropain) 26S subunit, non-ATPase, 2 | -0.2657 | 0.3313 | 0.7505 | 10.4579 | 10.0946 | 10.3359 | 10.3258 | 10.4012 | 10.4841 | 10.5327 | 10.7112 | 10.3359 | 10.491 | 10.2832 | 10.5802 |
| 204654_s_at | 7020 | TFAP2A | transcription factor AP-2 alpha (activating enhancer binding protein 2 alpha) | -0.2668 | 0.3872 | 0.7956 | 7.7522 | 8.0943 | 7.5727 | 7.9595 | 8.0384 | 7.9512 | 8.9351 | 8.1565 | 8.6346 | 8.3408 | 7.9623 | 7.8692 |
| 202986_at | 9915 | ARNT2 | aryl-hydrocarbon receptor nuclear translocator 2 | -0.267 | 0.4072 | 0.8101 | 11.1378 | 10.7518 | 8.6729 | 9.6092 | 9.6875 | 10.0688 | 10.491 | 9.6359 | 12.5889 | 11.7089 | 11.5626 | 11.2259 |
| 202093_s_at | 54623 | PAF1 | Paf1, RNA polymerase II associated factor, homolog (S. cerevisiae) | -0.2683 | 0.491 | 0.8692 | 8.1878 | 8.0555 | 7.9822 | 7.7198 | 7.9071 | 7.7878 | 7.4911 | 7.8285 | 8.3197 | 10.0502 | 7.9185 | 8.2084 |
| 209939_x_at | 8837 | CFLAR | CASP8 and FADD-like apoptosis regulator | -0.2685 | 0.5309 | 0.8786 | 5.9277 | 5.9693 | 5.9102 | 5.8045 | 5.9524 | 5.7423 | 6.0683 | 6.0466 | 6.1187 | 6.051 | 5.9559 | 5.7242 |
| 209682_at | 868 | CBLB | Cas-Br-M (murine) ecotropic retroviral transforming sequence b | -0.2706 | 0.4311 | 0.8252 | 5.9102 | 6.0116 | 5.6109 | 5.7166 | 5.6556 | 5.6619 | 5.9592 | 5.8045 | 6.0329 | 5.9102 | 6.3138 | 5.8625 |
| 202076_at | 329 | BIRC2 | baculoviral IAP repeat containing 2 | -0.2717 | 0.4691 | 0.848 | 6.8236 | 6.7607 | 7.0411 | 6.8786 | 7.0252 | 6.7758 | 6.9645 | 6.8675 | 6.8551 | 7.1557 | 7.0333 | 6.7033 |
| 208847_s_at | 128 | ADH5 | alcohol dehydrogenase 5 (class III), chi polypeptide | -0.272 | 0.3992 | 0.809 | 9.2633 | 9.1386 | 5.8338 | 9.3644 | 9.1143 | 9.1555 | 8.9136 | 9.4888 | 8.6562 | 8.7844 | 7.8512 | 9.2079 |
| 202208_s_at | 10123 | ARL4C | ADP-ribosylation factor-like 4C | -0.2733 | 0.4232 | 0.8179 | 7.249 | 6.9556 | 7.2641 | 6.836 | 7.0076 | 6.6183 | 7.0381 | 6.8551 | 7.6005 | 7.1474 | 7.7413 | 7.0275 |
| 218743_at | 79643 | CHMP6 | charged multivesicular body protein 6 | -0.2763 | 0.9182 | 0.9845 | 5.0618 | 4.8949 | 5.1948 | 4.6373 | 4.4955 | 4.8161 | 4.7487 | 4.4812 | 4.65 | 4.8161 | 4.5225 | 8.8182 |
| 212968_at | 5986 | RFNG | RFNG O-fucosylpeptide 3-beta-N-acetylglucosaminyltransferase | -0.2784 | 0.3832 | 0.7956 | 10.6225 | 9.1706 | 8.446 | 8.7781 | 8.9136 | 9.3908 | 11.1378 | 9.3603 | 11.2563 | 9.7498 | 10.2223 | 9.6716 |
| 200789_at | 1891 | ECH1 | enoyl CoA hydratase 1, peroxisomal | -0.283 | 0.3054 | 0.7309 | 8.3822 | 8.7356 | 8.3443 | 7.9595 | 8.4664 | 8.5113 | 8.6906 | 8.8377 | 9.3333 | 9.0472 | 8.9681 | 9.1555 |
| 212231_at | 23014 | FBXO21 | F-box protein 21 | -0.2845 | 0.2774 | 0.7236 | 6.7967 | 10.3781 | 6.9645 | 10.3997 | 10.1385 | 10.1602 | 10.3576 | 10.4012 | 10.6275 | 10.2552 | 7.9232 | 10.5113 |
| 201763_s_at | 1616 | DAXX | death-domain associated protein | -0.2851 | 0.3772 | 0.7934 | 8.4815 | 9.706 | 7.7733 | 7.9019 | 7.7617 | 7.9071 | 7.4336 | 7.6974 | 10.8832 | 9.3586 | 9.5715 | 8.5472 |
| 218619_s_at | 6839 | SUV39H1 | suppressor of variegation 3-9 homolog 1 (Drosophila) | -0.2858 | 0.3413 | 0.7552 | 8.2457 | 8.446 | 8.4664 | 8.6882 | 8.2735 | 8.2354 | 8.1878 | 8.4166 | 8.4555 | 8.3639 | 8.6346 | 8.0043 |
| 218910_at | 55129 | ANO10 | anoctamin 10 | -0.2877 | 0.3872 | 0.7956 | 5.5514 | 5.5378 | 6.0275 | 5.7242 | 5.9831 | 5.7278 | 5.7604 | 5.5863 | 9.3022 | 5.3817 | 5.6109 | 5.9069 |
| 215884_s_at | 29978 | UBQLN2 | ubiquilin 2 | -0.2881 | 0.4172 | 0.8166 | 8.094 | 8.0384 | 8.0375 | 8.0709 | 7.9386 | 7.9566 | 8.1918 | 8.3408 | 8.2423 | 7.9185 | 7.9974 | 8.0157 |
| 209262_s_at | 2063 | NR2F6 | nuclear receptor subfamily 2, group F, member 6 | -0.2907 | 0.2834 | 0.7295 | 9.3433 | 8.502 | 8.9058 | 8.9883 | 7.848 | 8.8671 | 11.2563 | 9.776 | 7.2667 | 9.2633 | 9.5376 | 9.8445 |
| 217931_at | 10695 | CNPY3 | canopy 3 homolog (zebrafish) | -0.2925 | 0.4371 | 0.8275 | 6.7904 | 6.6502 | 6.1444 | 6.9475 | 6.5758 | 7.5805 | 8.0938 | 7.9232 | 6.731 | 6.7808 | 6.9865 | 6.937 |
| 212485_at | 23131 | GPATCH8 | G patch domain containing 8 | -0.2943 | 0.8204 | 0.9716 | 7.1557 | 7.4889 | 7.7535 | 7.594 | 7.2836 | 7.4385 | 7.3974 | 7.4033 | 7.1877 | 7.1725 | 7.4732 | 7.1292 |
| 216913_s_at | 23223 | RRP12 | ribosomal RNA processing 12 homolog (S. cerevisiae) | -0.2944 | 0.3273 | 0.748 | 7.2667 | 7.2621 | 7.3845 | 8.249 | 7.6629 | 7.3944 | 8.296 | 7.4255 | 7.3824 | 7.2396 | 8.4501 | 8.3158 |
| 218435_at | 29103 | DNAJC15 | DnaJ (Hsp40) homolog, subfamily C, member 15 | -0.295 | 0.3333 | 0.7505 | 7.4055 | 7.3307 | 7.5506 | 7.1768 | 7.1474 | 7.0824 | 8.4172 | 7.358 | 7.9882 | 7.5612 | 7.5357 | 7.3277 |
| 202602_s_at | 27336 | HTATSF1 | HIV-1 Tat specific factor 1 | -0.2964 | 0.3034 | 0.7309 | 11.7318 | 11.9984 | 11.8756 | 11.8924 | 12.0594 | 12.2004 | 12.5517 | 12.5517 | 12.3455 | 11.9863 | 12.2244 | 12.465 |
| 215903_s_at | 23139 | MAST2 | microtubule associated serine/threonine kinase 2 | -0.2971 | 0.3952 | 0.8052 | 7.1877 | 7.2396 | 7.1421 | 7.4255 | 7.249 | 6.9618 | 7.2641 | 7.2032 | 7.7413 | 7.375 | 7.0977 | 6.8907 |
| 203213_at | 983 | CDK1 | cyclin-dependent kinase 1 | -0.2992 | 0.3653 | 0.7817 | 11.2801 | 10.9381 | 11.0543 | 10.9381 | 11.1141 | 10.9381 | 10.7631 | 10.9929 | 10.6038 | 11.1291 | 11.2078 | 11.0744 |
| 201967_at | 10180 | RBM6 | RNA binding motif protein 6 | -0.3002 | 0.521 | 0.8768 | 8.0481 | 8.115 | 8.1918 | 8.2354 | 8.3656 | 8.0375 | 8.4735 | 8.2886 | 8.0132 | 8.0943 | 9.5094 | 9.1386 |
| 212180_at | 1399 | CRKL | v-crk sarcoma virus CT10 oncogene homolog (avian)-like | -0.3019 | 0.505 | 0.8748 | 10.1184 | 9.7015 | 9.5715 | 9.5894 | 9.5496 | 9.7142 | 9.7213 | 9.863 | 11.4334 | 10.6716 | 10.6716 | 9.9759 |
| 204587_at | 9016 | SLC25A14 | solute carrier family 25 (mitochondrial carrier, brain), member 14 | -0.304 | 0.3074 | 0.7309 | 6.0914 | 7.3415 | 7.3974 | 7.3467 | 7.0805 | 6.1957 | 7.2881 | 7.4873 | 6.5245 | 6.1336 | 7.4435 | 7.6781 |
| 219968_at | 51385 | ZNF589 | zinc finger protein 589 | -0.3086 | 0.521 | 0.8768 | 7.0042 | 6.836 | 7.1254 | 7.0275 | 7.4732 | 7.1215 | 6.8938 | 8.6135 | 7.2343 | 7.3258 | 7.1587 | 6.9488 |
| 219184_x_at | 29928 | TIMM22 | translocase of inner mitochondrial membrane 22 homolog (yeast) | -0.3124 | 0.3214 | 0.743 | 8.2084 | 7.8692 | 8.4622 | 8.2259 | 8.0672 | 8.3639 | 7.7072 | 8.3972 | 7.9208 | 8.2067 | 8.3248 | 8.205 |
| 222217_s_at | 11000 | SLC27A3 | solute carrier family 27 (fatty acid transporter), member 3 | -0.3133 | 0.3074 | 0.7309 | 8.9158 | 8.1874 | 7.6429 | 7.7856 | 7.9232 | 8.8821 | 9.1906 | 8.5141 | 10.3781 | 9.2274 | 9.647 | 9.2274 |
| 203494_s_at | 9702 | CEP57 | centrosomal protein 57kDa | -0.3174 | 0.3573 | 0.7765 | 7.6416 | 7.3307 | 7.2524 | 7.2641 | 7.0843 | 7.0176 | 7.4511 | 7.2654 | 7.4873 | 7.9232 | 7.461 | 7.2967 |
| 205120_s_at | 6443 | SGCB | sarcoglycan, beta (43kDa dystrophin-associated glycoprotein) | -0.3199 | 0.2735 | 0.717 | 6.4376 | 5.8045 | 6.5054 | 6.5023 | 6.413 | 6.1187 | 6.2658 | 6.56 | 6.0847 | 6.1608 | 6.2131 | 6.2034 |
| 205498_at | 2690 | GHR | growth hormone receptor | -0.3203 | 0.3253 | 0.7469 | 6.1444 | 6.0718 | 6.1092 | 6.2326 | 6.1957 | 6.1155 | 5.9592 | 6.217 | 5.8537 | 6.299 | 5.8825 | 5.9559 |
| 219711_at | 54807 | ZNF586 | zinc finger protein 586 | -0.321 | 0.1637 | 0.6157 | 5.3933 | 5.3755 | 5.5982 | 5.6937 | 5.1321 | 5.3682 | 10.9929 | 5.6507 | 5.7637 | 5.607 | 5.4195 | 5.9069 |
| 212943_at | 9847 | KIAA0528 | KIAA0528 | -0.3216 | 0.507 | 0.8748 | 11.2259 | 11.3665 | 11.2801 | 11.1141 | 11.0292 | 6.6204 | 10.8218 | 11.2801 | 11.1141 | 11.4048 | 11.3234 | 10.8448 |
| 219821_s_at | 54438 | GFOD1 | glucose-fructose oxidoreductase domain containing 1 | -0.3231 | 0.5629 | 0.8876 | 6.0527 | 6.2268 | 5.9211 | 6.4578 | 6.1871 | 6.3138 | 5.9169 | 6.3947 | 10.8448 | 6.3348 | 6.3048 | 9.2754 |
| 201234_at | 3611 | ILK | integrin-linked kinase | -0.3253 | 0.2575 | 0.7049 | 8.9897 | 9.4264 | 9.5496 | 9.0574 | 8.9432 | 9.0983 | 8.9158 | 9.2274 | 8.7079 | 9.41 | 9.6609 | 9.5009 |
| 201932_at | 10489 | LRRC41 | leucine rich repeat containing 41 | -0.3256 | 0.2635 | 0.7094 | 10.8448 | 10.8407 | 10.7277 | 10.9533 | 9.7373 | 10.5646 | 11.1141 | 10.8754 | 11.237 | 10.8535 | 11.2801 | 11.0907 |
| 213689_x_at | 388650 | FAM69A | family with sequence similarity 69, member A | -0.3259 | 0.2495 | 0.6992 | 6.923 | 7.1557 | 9.2516 | 9.1996 | 9.776 | 9.9331 | 10.5886 | 9.7561 | 6.1877 | 9.647 | 10.1385 | 10.3074 |
| 213995_at | 27109 | ATP5S | ATP synthase, H+ transporting, mitochondrial Fo complex, subunit s (factor B) | -0.326 | 0.2695 | 0.7161 | 6.731 | 7.4255 | 6.8938 | 6.8786 | 7.27 | 6.9198 | 6.6276 | 6.1092 | 7.6595 | 7.838 | 7.8401 | 6.6619 |
| 202889_x_at | 9053 | MAP7 | microtubule-associated protein 7 | -0.3261 | 0.8144 | 0.9716 | 5.1023 | 4.9229 | 5.3933 | 5.3008 | 5.5863 | 5.294 | 11.617 | 5.294 | 5.473 | 5.2689 | 5.0739 | 5.1982 |
| 219061_s_at | 8270 | LAGE3 | L antigen family, member 3 | -0.3278 | 0.3373 | 0.7532 | 5.8059 | 5.4862 | 6.1336 | 5.9102 | 5.9744 | 6.0038 | 5.609 | 6.1155 | 5.6368 | 6.2758 | 5.8045 | 5.8749 |
| 212694_s_at | 5096 | PCCB | propionyl CoA carboxylase, beta polypeptide | -0.3309 | 0.2595 | 0.7049 | 9.3187 | 10.6225 | 10.4357 | 9.9373 | 10.0554 | 10.1416 | 10.7415 | 10.7518 | 10.6275 | 10.0188 | 9.8521 | 10.3359 |
| 200814_at | 5720 | PSME1 | proteasome (prosome, macropain) activator subunit 1 (PA28 alpha) | -0.3338 | 0.2575 | 0.7049 | 8.748 | 9.0581 | 9.0983 | 9.1601 | 8.9671 | 9.4092 | 9.0193 | 8.9167 | 8.7265 | 9.0581 | 9.238 | 8.9351 |
| 218807_at | 10451 | VAV3 | vav 3 guanine nucleotide exchange factor | -0.3396 | 0.2735 | 0.717 | 5.6716 | 5.452 | 5.7278 | 6.1242 | 5.7835 | 5.4862 | 5.5278 | 5.2589 | 6.051 | 5.9559 | 6.2131 | 8.0943 |
| 205467_at | 843 | CASP10 | caspase 10, apoptosis-related cysteine peptidase | -0.3399 | 0.2735 | 0.717 | 6.2473 | 6.7693 | 5.8589 | 6.0302 | 6.3433 | 6.8468 | 7.7576 | 6.8316 | 6.7968 | 6.2473 | 6.5676 | 7.3695 |
| 200053_at | 9552 | SPAG7 | sperm associated antigen 7 | -0.3447 | 0.2136 | 0.6689 | 8.4002 | 8.272 | 8.2886 | 8.1688 | 8.4172 | 8.7157 | 8.8195 | 8.524 | 8.557 | 8.6639 | 8.7265 | 8.5388 |
| 203279_at | 9695 | EDEM1 | ER degradation enhancer, mannosidase alpha-like 1 | -0.3461 | 0.2295 | 0.6759 | 7.7133 | 7.4188 | 7.4188 | 7.4715 | 7.3089 | 7.7072 | 7.6938 | 7.5761 | 7.5564 | 7.6365 | 7.6781 | 7.9445 |
| 200966_x_at | 226 | ALDOA | aldolase A, fructose-bisphosphate | -0.3472 | 0.2575 | 0.7049 | 5.5732 | 5.736 | 6.0078 | 5.9126 | 6.7205 | 5.5182 | 6.1796 | 6.289 | 6.3809 | 6.7693 | 5.9277 | 6.1877 |
| 202908_at | 7466 | WFS1 | Wolfram syndrome 1 (wolframin) | -0.3483 | 0.2315 | 0.6759 | 6.8515 | 7.1557 | 6.8938 | 6.7205 | 7.044 | 7.0024 | 6.8126 | 6.7423 | 7.1474 | 7.1557 | 7.3055 | 7.3974 |
| 218215_s_at | 7376 | NR1H2 | nuclear receptor subfamily 1, group H, member 2 | -0.3492 | 0.2455 | 0.696 | 6.6399 | 6.8157 | 6.6183 | 6.519 | 6.8907 | 7.1877 | 6.5894 | 6.8468 | 7.2881 | 7.3377 | 7.2559 | 7.0597 |
| 204542_at | 10610 | ST6GALNAC2 | ST6 (alpha-N-acetyl-neuraminyl-2,3-beta-galactosyl-1,3)-N-acetylgalactosaminide alpha-2,6-sialyltransferase 2 | -0.3497 | 0.7745 | 0.971 | 5.3933 | 5.3176 | 5.4947 | 5.4354 | 5.473 | 5.2763 | 5.2037 | 5.0322 | 5.3008 | 5.0884 | 5.3875 | 12.3455 |
| 207416_s_at | 4775 | NFATC3 | nuclear factor of activated T-cells, cytoplasmic, calcineurin-dependent 3 | -0.3506 | 0.6427 | 0.9163 | 7.1241 | 7.0111 | 7.0252 | 6.9161 | 6.7808 | 7.1746 | 6.9591 | 6.9475 | 6.9073 | 7.0111 | 11.9462 | 7.4435 |
| 202801_at | 5566 | PRKACA | protein kinase, cAMP-dependent, catalytic, alpha | -0.3533 | 0.2176 | 0.6689 | 8.5506 | 8.8935 | 8.7683 | 8.7293 | 8.9058 | 9.8495 | 9.9331 | 9.9658 | 10.0573 | 9.2754 | 9.7719 | 9.5537 |
| 209515_s_at | 5873 | RAB27A | RAB27A, member RAS oncogene family | -0.3536 | 0.2295 | 0.6759 | 7.5013 | 7.1924 | 5.831 | 7.4435 | 7.3021 | 7.6704 | 7.9752 | 7.9185 | 7.3188 | 7.2654 | 7.3258 | 7.8575 |
| 201774_s_at | 9918 | NCAPD2 | non-SMC condensin I complex, subunit D2 | -0.3545 | 0.1597 | 0.6077 | 8.7226 | 7.6938 | 8.6247 | 8.7012 | 8.7913 | 8.7012 | 9.0859 | 8.9681 | 9.032 | 8.9351 | 9.1555 | 9.3187 |
| 202738_s_at | 5257 | PHKB | phosphorylase kinase, beta | -0.3545 | 0.2295 | 0.6759 | 6.8907 | 6.519 | 6.6399 | 6.5365 | 6.7607 | 6.4541 | 6.5023 | 6.7808 | 6.8479 | 6.8406 | 6.9965 | 6.7289 |
| 203067_at | 8050 | PDHX | pyruvate dehydrogenase complex, component X | -0.3554 | 0.4451 | 0.8324 | 8.7781 | 8.8613 | 5.0618 | 8.654 | 8.4735 | 8.8492 | 8.9454 | 8.8671 | 8.5715 | 8.4602 | 8.7457 | 8.7844 |
| 200636_s_at | 5792 | PTPRF | protein tyrosine phosphatase, receptor type, F | -0.3572 | 0.2535 | 0.7049 | 7.6527 | 7.6704 | 7.441 | 6.9922 | 6.937 | 7.7522 | 8.1748 | 7.8575 | 9.5496 | 8.0348 | 8.7153 | 8.2259 |
| 217478_s_at | 3108 | HLA-DMA | major histocompatibility complex, class II, DM alpha | -0.3577 | 0.2355 | 0.6835 | 4.612 | 4.7565 | 4.308 | 4.766 | 4.5899 | 4.9657 | 4.685 | 4.7052 | 4.5225 | 4.8576 | 4.7409 | 4.9166 |
| 202703_at | 8446 | DUSP11 | dual specificity phosphatase 11 (RNA/RNP complex 1-interacting) | -0.3577 | 0.2275 | 0.6759 | 9.9658 | 9.6609 | 9.7917 | 9.3308 | 9.7719 | 9.4483 | 9.4008 | 9.4254 | 9.7957 | 9.6908 | 9.7142 | 9.9162 |
| 89476_r_at | 79716 | NPEPL1 | aminopeptidase-like 1 | -0.3578 | 0.2495 | 0.6992 | 6.5301 | 6.7423 | 6.7289 | 6.9803 | 6.5942 | 6.9522 | 7.6126 | 6.8978 | 7.0932 | 6.7968 | 6.794 | 6.9098 |
| 206109_at | 2523 | FUT1 | fucosyltransferase 1 (galactoside 2-alpha-L-fucosyltransferase, H blood group) | -0.3581 | 0.2435 | 0.696 | 6.9298 | 6.3601 | 5.1062 | 5.4862 | 5.8229 | 5.7669 | 6.7922 | 5.9102 | 7.6416 | 7.1924 | 6.9488 | 7.0111 |
| 204579_at | 2264 | FGFR4 | fibroblast growth factor receptor 4 | -0.3602 | 0.2495 | 0.6992 | 5.6977 | 5.7242 | 6.0073 | 5.8589 | 5.9626 | 6.2691 | 6.1242 | 6.2624 | 5.7879 | 5.803 | 5.6799 | 6.0193 |
| 209747_at | 7043 | TGFB3 | transforming growth factor, beta 3 | -0.3615 | 0.2495 | 0.6992 | 6.0683 | 6.1242 | 5.6125 | 5.6175 | 5.7669 | 5.641 | 6.9757 | 6.6524 | 6.5867 | 6.2624 | 5.9813 | 6.7968 |
| 201469_s_at | 6464 | SHC1 | SHC (Src homology 2 domain containing) transforming protein 1 | -0.3616 | 0.3234 | 0.7441 | 8.094 | 7.7856 | 7.2667 | 7.3974 | 7.7351 | 7.872 | 8.2354 | 7.4255 | 8.8613 | 10.3682 | 8.4825 | 10.5327 |
| 209875_s_at | 6696 | SPP1 | secreted phosphoprotein 1 | -0.3616 | 0.1317 | 0.5804 | 3.5495 | 3.5495 | 3.641 | 3.5495 | 3.641 | 3.5495 | 3.641 | 3.4398 | 3.5495 | 3.7081 | 3.7081 | 3.4398 |
| 213343_s_at | 81544 | GDPD5 | glycerophosphodiester phosphodiesterase domain containing 5 | -0.3619 | 0.3094 | 0.7309 | 6.5425 | 6.6183 | 6.6524 | 6.6638 | 6.6502 | 7.0024 | 7.0024 | 6.731 | 6.8432 | 6.5921 | 6.8922 | 6.6619 |
| 210719_s_at | 10362 | HMG20B | high mobility group 20B | -0.3628 | 0.2315 | 0.6759 | 9.203 | 9.4008 | 9.3209 | 9.623 | 9.5894 | 9.7917 | 9.3258 | 9.7015 | 9.7101 | 9.3515 | 9.6213 | 9.6875 |
| 218290_at | 55111 | PLEKHJ1 | pleckstrin homology domain containing, family J member 1 | -0.3644 | 0.2295 | 0.6759 | 8.8209 | 8.5966 | 8.8096 | 8.6099 | 8.7116 | 8.7366 | 8.7929 | 8.8935 | 9.4483 | 8.654 | 8.5919 | 8.9058 |
| 202599_s_at | 8204 | NRIP1 | nuclear receptor interacting protein 1 | -0.3656 | 0.1537 | 0.6012 | 6.9865 | 6.8922 | 7.8928 | 7.0954 | 7.6629 | 7.7351 | 8.0118 | 6.7726 | 7.6629 | 7.7617 | 7.836 | 7.7733 |
| 201847_at | 3988 | LIPA | lipase A, lysosomal acid, cholesterol esterase | -0.3671 | 0.2196 | 0.6689 | 9.0263 | 9.0574 | 9.2754 | 9.0263 | 9.1196 | 8.6284 | 8.7781 | 9.2204 | 8.9691 | 8.9622 | 9.1512 | 8.9398 |
| 207700_s_at | 8202 | NCOA3 | nuclear receptor coactivator 3 | -0.3684 | 0.2335 | 0.6797 | 7.4688 | 7.5013 | 7.3914 | 7.7617 | 7.4973 | 7.0995 | 8.6476 | 7.9704 | 7.8692 | 7.9752 | 8.0132 | 8.0104 |
| 211758_x_at | 10190 | TXNDC9 | thioredoxin domain containing 9 | -0.3695 | 0.1517 | 0.5982 | 4.4407 | 7.2265 | 7.1634 | 7.2032 | 7.0977 | 6.7808 | 7.1703 | 6.9645 | 7.4336 | 7.4044 | 7.685 | 7.2304 |
| 203464_s_at | 22905 | EPN2 | epsin 2 | -0.3701 | 0.2994 | 0.7309 | 7.9232 | 8.3822 | 8.1688 | 8.1021 | 8.5324 | 9.1623 | 9.1996 | 8.3088 | 8.6171 | 8.1874 | 8.6858 | 8.6688 |
| 213419_at | 323 | APBB2 | amyloid beta (A4) precursor protein-binding, family B, member 2 | -0.3706 | 0.1657 | 0.6184 | 6.5942 | 6.2624 | 6.0818 | 5.9795 | 5.9126 | 6.3524 | 7.0284 | 6.3121 | 6.9803 | 6.5769 | 6.4053 | 6.1289 |
| 221610_s_at | 55620 | STAP2 | signal transducing adaptor family member 2 | -0.3709 | 0.479 | 0.8596 | 5.4553 | 5.0204 | 5.1982 | 4.9937 | 4.6246 | 5.1948 | 4.9567 | 4.8042 | 10.1184 | 5.1707 | 5.3047 | 4.9901 |
| 213324_at | 6714 | SRC | v-src sarcoma (Schmidt-Ruppin A-2) viral oncogene homolog (avian) | -0.3715 | 0.4511 | 0.8324 | 3.8487 | 3.7081 | 3.8487 | 3.955 | 3.9141 | 4.2178 | 4.2034 | 4.151 | 3.641 | 3.641 | 3.8487 | 3.9983 |
| 202996_at | 57804 | POLD4 | polymerase (DNA-directed), delta 4 | -0.3719 | 0.1956 | 0.6485 | 5.7278 | 5.7104 | 5.9873 | 5.5732 | 5.3875 | 5.5182 | 6.1187 | 5.9277 | 5.9488 | 5.7604 | 5.7925 | 5.706 |
| 204757_s_at | 9854 | C2CD2L | C2CD2-like | -0.3721 | 0.2315 | 0.6759 | 5.4641 | 5.4553 | 5.4641 | 5.3609 | 5.8229 | 6.3919 | 5.5037 | 5.5664 | 5.4388 | 5.6109 | 5.9277 | 5.609 |
| 216598_s_at | 6347 | CCL2 | chemokine (C-C motif) ligand 2 | -0.3734 | 0.4212 | 0.8172 | 5.641 | 5.6507 | 5.3503 | 5.4992 | 5.4159 | 5.4123 | 5.4641 | 5.4123 | 6.0231 | 5.4388 | 5.3875 | 7.8826 |
| 203566_s_at | 178 | AGL | amylo-alpha-1, 6-glucosidase, 4-alpha-glucanotransferase | -0.3754 | 0.2974 | 0.7309 | 7.499 | 7.7811 | 7.9566 | 7.7133 | 8.0536 | 7.844 | 7.9232 | 7.8706 | 8.1918 | 7.7351 | 8.7366 | 7.6365 |
| 219352_at | 55008 | HERC6 | hect domain and RLD 6 | -0.3767 | 0.2874 | 0.7309 | 5.5548 | 5.5548 | 5.2182 | 5.3176 | 5.5037 | 5.3176 | 5.8552 | 5.8229 | 5.7879 | 5.8625 | 5.3609 | 5.3817 |
| 218902_at | 4851 | NOTCH1 | notch 1 | -0.3769 | 0.04192 | 0.4361 | 7.657 | 7.2621 | 6.9556 | 7.0474 | 7.3258 | 7.0932 | 7.6365 | 7.3881 | 7.4144 | 7.4732 | 7.3377 | 7.6025 |
| 212875_s_at | 25966 | C2CD2 | C2 calcium-dependent domain containing 2 | -0.3808 | 0.0519 | 0.4614 | 4.2178 | 3.955 | 4.2444 | 4.0871 | 4.3669 | 4.4147 | 4.4407 | 4.052 | 4.4812 | 4.3929 | 4.5509 | 4.3164 |
| 202635_s_at | 5440 | POLR2K | polymerase (RNA) II (DNA directed) polypeptide K, 7.0kDa | -0.3816 | 0.2076 | 0.6613 | 11.9351 | 12.0594 | 12.0363 | 11.9984 | 11.9239 | 12.1067 | 11.8331 | 12.0594 | 11.7089 | 12.1301 | 12.0917 | 12.1301 |
| 221060_s_at | 7099 | TLR4 | toll-like receptor 4 | -0.3817 | 0.03992 | 0.4338 | 4.151 | 4.6437 | 4.9229 | 4.2682 | 4.6246 | 4.5098 | 5.0655 | 4.3452 | 4.8651 | 4.4407 | 5.0263 | 4.8362 |
| 201849_at | 664 | BNIP3 | BCL2/adenovirus E1B 19kDa interacting protein 3 | -0.3853 | 0.1796 | 0.6322 | 7.7986 | 7.9232 | 8.4172 | 7.6723 | 7.7461 | 7.9131 | 8.0157 | 8.0517 | 7.4688 | 8.0446 | 8.1986 | 7.9945 |
| 202637_s_at | 3383 | ICAM1 | intercellular adhesion molecule 1 | -0.3854 | 0.05788 | 0.4718 | 4.8613 | 4.6951 | 4.3929 | 4.65 | 4.685 | 4.5899 | 4.7924 | 4.8822 | 4.9864 | 4.9937 | 4.8651 | 4.7052 |
| 202942_at | 2109 | ETFB | electron-transfer-flavoprotein, beta polypeptide | -0.3881 | 0.2076 | 0.6613 | 8.7878 | 8.9671 | 8.3723 | 8.4735 | 9.266 | 9.2204 | 8.581 | 9.4517 | 8.9175 | 9.232 | 8.4664 | 9.2316 |
| 202454_s_at | 2065 | ERBB3 | v-erb-b2 erythroblastic leukemia viral oncogene homolog 3 (avian) | -0.3896 | 0.04391 | 0.4402 | 4.4147 | 4.8362 | 4.2178 | 4.3308 | 4.1889 | 4.2996 | 4.8651 | 4.7131 | 4.7052 | 4.4812 | 11.5189 | 4.8822 |
| 203709_at | 5261 | PHKG2 | phosphorylase kinase, gamma 2 (testis) | -0.3905 | 0.2136 | 0.6689 | 7.5612 | 7.3258 | 7.2641 | 7.3258 | 7.436 | 7.2343 | 7.0805 | 7.5064 | 8.3656 | 7.6595 | 7.7291 | 7.3549 |
| 203911_at | 5909 | RAP1GAP | RAP1 GTPase activating protein | -0.3928 | 0.2575 | 0.7049 | 6.9123 | 6.8978 | 6.8703 | 7.358 | 6.9198 | 6.923 | 6.7539 | 7.0805 | 6.9645 | 6.9335 | 7.9019 | 6.9922 |
| 218407_x_at | 29937 | NENF | neudesin neurotrophic factor | -0.3942 | 0.1836 | 0.6346 | 10.9321 | 10.8407 | 11.1378 | 11.0744 | 10.8682 | 11.2563 | 10.9321 | 11.2259 | 10.7112 | 11.0744 | 11.1378 | 11.1661 |
| 205039_s_at | 10320 | IKZF1 | IKAROS family zinc finger 1 (Ikaros) | -0.3969 | 0.02395 | 0.411 | 4.3452 | 4.1341 | 4.151 | 4.2178 | 4.0871 | 4.0871 | 4.3452 | 4.2996 | 3.9141 | 4.2682 | 4.4486 | 4.5367 |
| 218113_at | 23670 | TMEM2 | transmembrane protein 2 | -0.3997 | 0.1577 | 0.6071 | 5.7604 | 5.6799 | 5.9524 | 5.7925 | 5.9009 | 5.5982 | 6.0073 | 5.6125 | 5.9277 | 6.0073 | 5.7104 | 6.1877 |
| 204809_at | 10845 | CLPX | ClpX caseinolytic peptidase X homolog (E. coli) | -0.4038 | 0.2016 | 0.6529 | 7.6044 | 7.7878 | 7.9071 | 8.3388 | 8.4501 | 7.5446 | 8.3408 | 7.7856 | 7.4003 | 7.8692 | 8.5506 | 8.3107 |
| 203456_at | 11230 | PRAF2 | PRA1 domain family, member 2 | -0.4044 | 0.1776 | 0.6322 | 8.1348 | 7.9185 | 8.1986 | 7.8285 | 7.9882 | 7.7522 | 7.8826 | 8.4339 | 7.7856 | 7.8575 | 8.3178 | 7.8745 |
| 204756_at | 5607 | MAP2K5 | mitogen-activated protein kinase kinase 5 | -0.4068 | 0.2754 | 0.7203 | 7.7878 | 7.7072 | 7.3519 | 7.7382 | 7.5612 | 7.7072 | 7.836 | 7.7811 | 12.0363 | 7.5692 | 7.6429 | 7.5692 |
| 203919_at | 6919 | TCEA2 | transcription elongation factor A (SII), 2 | -0.4075 | 0.1996 | 0.6529 | 7.5805 | 7.5337 | 7.3292 | 7.2836 | 7.5064 | 7.6595 | 8.654 | 8.0728 | 8.4602 | 7.8733 | 7.5564 | 8.4735 |
| 203659_s_at | 10206 | TRIM13 | tripartite motif containing 13 | -0.4098 | 0.1996 | 0.6529 | 7.0859 | 6.6562 | 7.4511 | 6.8458 | 6.9298 | 7.2784 | 8.9794 | 8.0157 | 7.6704 | 7.5357 | 7.2654 | 8.3273 |
| 209163_at | 1534 | CYB561 | cytochrome b-561 | -0.412 | 0.2954 | 0.7309 | 6.3187 | 6.5942 | 6.5758 | 6.4578 | 6.4376 | 6.4991 | 6.5375 | 6.6183 | 6.3706 | 6.496 | 6.6032 | 6.2789 |
| 202466_at | 11044 | PAPD7 | PAP associated domain containing 7 | -0.4121 | 0.1936 | 0.6441 | 8.7945 | 8.2259 | 7.4255 | 7.5357 | 8.0043 | 7.8786 | 9.706 | 8.9454 | 9.9192 | 9.2815 | 9.1036 | 9.5238 |
| 217809_at | 28969 | BZW2 | basic leucine zipper and W2 domains 2 | -0.4126 | 0.2116 | 0.6675 | 8.6688 | 9.0193 | 8.6099 | 8.8748 | 8.8642 | 8.5167 | 8.5096 | 8.5869 | 9.1265 | 8.7012 | 8.7844 | 8.9691 |
| 209615_s_at | 5058 | PAK1 | p21 protein (Cdc42/Rac)-activated kinase 1 | -0.4143 | 0.2435 | 0.696 | 6.5769 | 6.1984 | 6.3919 | 6.5993 | 6.5127 | 6.8842 | 6.6599 | 6.5931 | 6.3388 | 11.4472 | 6.3809 | 6.3706 |
| 203054_s_at | 6988 | TCTA | T-cell leukemia translocation altered gene | -0.4152 | 0.1477 | 0.5982 | 10.3927 | 9.5894 | 10.5393 | 10.5327 | 9.863 | 10.0005 | 10.6909 | 10.6716 | 10.6154 | 10.4237 | 9.8325 | 10.9184 |
| 202507_s_at | 6616 | SNAP25 | synaptosomal-associated protein, 25kDa | -0.4161 | 0.2655 | 0.7094 | 4.5098 | 4.4147 | 3.9983 | 4.2996 | 3.7648 | 4.7565 | 4.6601 | 3.9983 | 5.2182 | 10.0688 | 4.6702 | 4.3669 |
| 221562_s_at | 23410 | SIRT3 | sirtuin 3 | -0.4169 | 0.1896 | 0.6395 | 3.955 | 3.8487 | 3.7081 | 3.7648 | 3.955 | 3.7081 | 4.2839 | 3.9141 | 4.1889 | 3.8487 | 3.641 | 4.0695 |
| 222103_at | 466 | ATF1 | activating transcription factor 1 | -0.4186 | 0.1198 | 0.5686 | 8.3224 | 8.1103 | 8.4602 | 8.2172 | 8.0375 | 8.2457 | 8.0874 | 8.5665 | 7.9623 | 8.1543 | 8.4555 | 8.4612 |
| 212403_at | 89910 | UBE3B | ubiquitin protein ligase E3B | -0.4207 | 0.2295 | 0.6759 | 7.0474 | 6.7033 | 7.0007 | 6.9073 | 7.0007 | 7.2396 | 7.3089 | 7.4715 | 7.0649 | 7.0597 | 6.937 | 7.7413 |
| 202310_s_at | 1277 | COL1A1 | collagen, type I, alpha 1 | -0.4212 | 0.1697 | 0.6261 | 4.052 | 4.5808 | 4.9657 | 4.727 | 5.8625 | 4.8651 | 4.3164 | 4.6246 | 5.5378 | 5.9211 | 5.7017 | 5.7278 |
| 203186_s_at | 6275 | S100A4 | S100 calcium binding protein A4 | -0.4224 | 0.1577 | 0.6071 | 7.3009 | 5.938 | 6.1871 | 6.2911 | 6.2691 | 7.1528 | 6.731 | 6.0818 | 7.0851 | 7.1088 | 6.7107 | 7.0851 |
| 221142_s_at | 55825 | PECR | peroxisomal trans-2-enoyl-CoA reductase | -0.4258 | 0.1118 | 0.5521 | 7.4033 | 7.4942 | 7.3845 | 7.5001 | 7.4044 | 7.6629 | 7.594 | 7.8706 | 7.2383 | 7.2064 | 7.4942 | 7.3021 |
| 208690_s_at | 9124 | PDLIM1 | PDZ and LIM domain 1 | -0.4271 | 0.1796 | 0.6322 | 8.3368 | 8.9622 | 8.5919 | 8.6808 | 8.8642 | 8.3158 | 8.4835 | 8.581 | 8.5869 | 8.801 | 8.6768 | 9.0574 |
| 203120_at | 7159 | TP53BP2 | tumor protein p53 binding protein, 2 | -0.4282 | 0.1896 | 0.6395 | 9.7373 | 9.6875 | 9.5814 | 9.4888 | 9.3433 | 9.2492 | 9.6785 | 10.0321 | 10.4499 | 9.6827 | 10.1416 | 9.4219 |
| 218529_at | 51293 | CD320 | CD320 molecule | -0.4317 | 0.1776 | 0.6322 | 10.9184 | 10.9664 | 10.9184 | 10.9664 | 10.6716 | 10.8448 | 10.7112 | 11.1291 | 10.7415 | 11.1661 | 10.8365 | 10.9381 |
| 212124_at | 57178 | ZMIZ1 | zinc finger, MIZ-type containing 1 | -0.4319 | 0.2016 | 0.6529 | 8.8128 | 8.6517 | 8.2519 | 8.3088 | 8.8311 | 8.6099 | 9.6827 | 8.5919 | 9.9373 | 8.7945 | 9.0383 | 8.78 |
| 219581_at | 80746 | TSEN2 | tRNA splicing endonuclease 2 homolog (S. cerevisiae) | -0.4331 | 0.02794 | 0.427 | 8.2633 | 8.446 | 8.359 | 8.5324 | 8.1688 | 8.546 | 8.6391 | 8.6906 | 8.5356 | 8.4002 | 8.5852 | 8.6983 |
| 205085_at | 4998 | ORC1 | origin recognition complex, subunit 1 | -0.4353 | 0.2814 | 0.7282 | 10.2832 | 9.8546 | 8.7978 | 8.7844 | 9.1706 | 9.2815 | 9.1706 | 9.0193 | 11.3911 | 10.3414 | 12.1301 | 9.9421 |
| 56197_at | 57048 | PLSCR3 | phospholipid scramblase 3 | -0.4359 | 0.2096 | 0.6655 | 5.7469 | 5.4686 | 5.5732 | 5.5548 | 5.5732 | 5.6175 | 5.7204 | 5.4388 | 6.2411 | 5.7166 | 6.157 | 5.7423 |
| 202847_at | 5106 | PCK2 | phosphoenolpyruvate carboxykinase 2 (mitochondrial) | -0.4371 | 0.1397 | 0.5865 | 6.56 | 6.3601 | 7.0176 | 6.7251 | 6.3895 | 6.0329 | 6.2473 | 6.645 | 6.8636 | 6.9645 | 7.2142 | 6.6032 |
| 203755_at | 701 | BUB1B | budding uninhibited by benzimidazoles 1 homolog beta (yeast) | -0.4423 | 0.1816 | 0.6322 | 9.5814 | 9.863 | 9.4092 | 9.6875 | 9.9192 | 9.9373 | 11.3118 | 10.2086 | 9.9759 | 9.995 | 10.7025 | 11.0139 |
| 204812_at | 9183 | ZW10 | ZW10, kinetochore associated, homolog (Drosophila) | -0.4431 | 0.1098 | 0.5521 | 8.0043 | 8.1688 | 8.3972 | 8.1348 | 9.0694 | 8.9855 | 7.5371 | 9.1265 | 9.1623 | 8.9748 | 9.0859 | 8.9432 |
| 205153_s_at | 958 | CD40 | CD40 molecule, TNF receptor superfamily member 5 | -0.4454 | 0.2455 | 0.696 | 8.2547 | 7.9752 | 6.8236 | 7.6211 | 7.5854 | 7.5897 | 7.795 | 7.2733 | 10.8365 | 9.2127 | 9.0922 | 8.5715 |
| 202891_at | 4817 | NIT1 | nitrilase 1 | -0.4464 | 0.1397 | 0.5865 | 8.6906 | 8.6099 | 8.5919 | 8.2547 | 8.502 | 8.4049 | 8.8981 | 8.7913 | 9.3099 | 9.0383 | 9.0411 | 9.2198 |
| 213384_x_at | 5331 | PLCB3 | phospholipase C, beta 3 (phosphatidylinositol-specific) | -0.448 | 0.07585 | 0.5012 | 7.1557 | 6.6684 | 6.8938 | 7.0007 | 7.0859 | 7.3467 | 7.3841 | 7.0843 | 7.044 | 7.2064 | 11.3348 | 7.2304 |
| 218149_s_at | 55893 | ZNF395 | zinc finger protein 395 | -0.4517 | 0.1577 | 0.6071 | 8.7155 | 8.6639 | 7.685 | 8.6338 | 8.495 | 8.2704 | 9.6875 | 8.7945 | 12.8313 | 11.1962 | 11.6409 | 9.1831 |
| 205573_s_at | 51375 | SNX7 | sorting nexin 7 | -0.453 | 0.1756 | 0.6322 | 8.115 | 8.2578 | 7.5131 | 7.3806 | 8.231 | 7.6781 | 11.3348 | 9.6056 | 9.2516 | 8.7913 | 8.6768 | 9.7213 |
| 212255_s_at | 27032 | ATP2C1 | ATPase, Ca++ transporting, type 2C, member 1 | -0.4539 | 0.1437 | 0.593 | 9.2452 | 8.6338 | 8.2332 | 9.7373 | 9.3515 | 10.045 | 8.9058 | 9.8973 | 12.2004 | 10.4133 | 10.6622 | 9.647 |
| 212206_s_at | 94239 | H2AFV | H2A histone family, member V | -0.4548 | 0.1916 | 0.644 | 7.7382 | 7.9882 | 7.499 | 7.836 | 8.0536 | 8.1543 | 8.3972 | 8.6284 | 8.2547 | 7.8155 | 9.3099 | 8.9454 |
| 201069_at | 4313 | MMP2 | matrix metallopeptidase 2 (gelatinase A, 72kDa gelatinase, 72kDa type IV collagenase) | -0.4587 | 0.1377 | 0.5865 | 6.687 | 6.3187 | 5.5138 | 5.7104 | 5.7017 | 5.803 | 7.5761 | 6.5769 | 6.9123 | 6.7904 | 7.2 | 7.3415 |
| 204106_at | 7016 | TESK1 | testis-specific kinase 1 | -0.462 | 0.1357 | 0.5865 | 8.7457 | 8.7226 | 7.7214 | 8.0384 | 8.5665 | 8.6688 | 10.3682 | 9.3515 | 10.4396 | 9.4092 | 9.7373 | 10.0688 |
| 201052_s_at | 9491 | PSMF1 | proteasome (prosome, macropain) inhibitor subunit 1 (PI31) | -0.4621 | 0.1437 | 0.593 | 7.3848 | 6.8316 | 6.5524 | 7.1088 | 7.4873 | 7.2784 | 7.4911 | 6.7922 | 8.4172 | 7.9704 | 8.0043 | 7.4942 |
| 203546_at | 9670 | IPO13 | importin 13 | -0.4643 | 0.09581 | 0.5294 | 8.7265 | 7.4463 | 7.3009 | 8.524 | 8.3754 | 8.8128 | 9.4305 | 8.5852 | 9.7213 | 9.4483 | 9.3022 | 8.5506 |
| 211729_x_at | 644 | BLVRA | biliverdin reductase A | -0.4651 | 0.1118 | 0.5521 | 8.6368 | 8.7913 | 8.9574 | 9.5009 | 9.3568 | 9.0023 | 9.2467 | 9.3209 | 9.2316 | 9.203 | 9.2754 | 9.3515 |
| 212955_s_at | 5438 | POLR2I | polymerase (RNA) II (DNA directed) polypeptide I, 14.5kDa | -0.467 | 0.1397 | 0.5865 | 9.3258 | 9.1906 | 9.5094 | 9.3644 | 9.2192 | 9.5105 | 9.0263 | 9.4264 | 9.0574 | 9.5009 | 9.5009 | 9.1196 |
| 201136_at | 5355 | PLP2 | proteolipid protein 2 (colonic epithelium-enriched) | -0.4691 | 0.1517 | 0.5982 | 6.6781 | 6.2411 | 5.3817 | 5.736 | 6.0466 | 6.0427 | 6.9488 | 6.3433 | 8.0375 | 6.8938 | 7.1397 | 6.9296 |
| 221552_at | 57406 | ABHD6 | abhydrolase domain containing 6 | -0.4737 | 0.08782 | 0.5174 | 6.9573 | 6.7156 | 6.9335 | 6.8938 | 6.7107 | 7.1215 | 7.1474 | 7.249 | 7.3639 | 6.687 | 7.2641 | 6.9618 |
| 202447_at | 1666 | DECR1 | 2,4-dienoyl CoA reductase 1, mitochondrial | -0.4751 | 0.1038 | 0.5428 | 6.3138 | 6.475 | 6.4991 | 6.4805 | 6.2442 | 6.2624 | 6.0427 | 8.028 | 6.5867 | 8.6025 | 6.4778 | 6.3187 |
| 203675_at | 4925 | NUCB2 | nucleobindin 2 | -0.4791 | 0.1697 | 0.6261 | 8.1878 | 8.2423 | 8.3224 | 8.1874 | 8.3623 | 8.1055 | 8.3368 | 8.47 | 8.1439 | 8.1652 | 8.3178 | 8.1918 |
| 201102_s_at | 5211 | PFKL | phosphofructokinase, liver | -0.4804 | 0.06188 | 0.4765 | 5.7748 | 5.7559 | 6.095 | 5.7423 | 5.8552 | 5.8589 | 5.8474 | 5.8338 | 13.0053 | 5.6977 | 6.0073 | 5.7925 |
| 212765_at | 23271 | CAMSAP2 | calmodulin regulated spectrin-associated protein family, member 2 | -0.4844 | 0.2176 | 0.6689 | 9.4649 | 9.1623 | 8.6071 | 8.5919 | 8.5919 | 8.6517 | 9.2965 | 9.0055 | 10.491 | 9.8376 | 10.2496 | 10.518 |
| 218744_s_at | 29763 | PACSIN3 | protein kinase C and casein kinase substrate in neurons 3 | -0.4872 | 0.06587 | 0.4807 | 5.9831 | 6.1533 | 5.9795 | 6.157 | 6.1406 | 6.3348 | 6.1092 | 6.2473 | 8.6025 | 6.1871 | 6.1214 | 6.3308 |
| 203258_at | 10589 | DRAP1 | DR1-associated protein 1 (negative cofactor 2 alpha) | -0.4917 | 0.1477 | 0.5982 | 7.1171 | 7.0252 | 7.3223 | 7.5783 | 7.3639 | 6.9335 | 7.1397 | 7.0275 | 7.3258 | 7.685 | 7.5854 | 7.2641 |
| 202887_s_at | 54541 | DDIT4 | DNA-damage-inducible transcript 4 | -0.4987 | 0.1058 | 0.5474 | 6.519 | 8.1521 | 6.355 | 6.6204 | 6.607 | 6.8703 | 7.5013 | 7.1768 | 7.9208 | 7.0381 | 7.2 | 7.4222 |
| 209109_s_at | 7105 | TSPAN6 | tetraspanin 6 | -0.5012 | 0.08583 | 0.5118 | 10.8036 | 10.7112 | 10.8535 | 10.8535 | 10.7789 | 10.5015 | 10.6225 | 10.9664 | 10.3074 | 10.6622 | 10.8754 | 10.9321 |
| 218170_at | 51015 | ISOC1 | isochorismatase domain containing 1 | -0.5019 | 0.1697 | 0.6261 | 9.1831 | 9.2516 | 9.3644 | 9.1906 | 9.2995 | 9.0574 | 10.1954 | 9.7213 | 10.9664 | 9.5496 | 9.3209 | 10.0094 |
| 202016_at | 4232 | MEST | mesoderm specific transcript homolog (mouse) | -0.5074 | 0.1198 | 0.5686 | 11.0907 | 10.9184 | 9.8546 | 10.2832 | 11.1661 | 11.4334 | 13.363 | 12.4958 | 12.4097 | 12.0594 | 12.5517 | 13.0626 |
| 212624_s_at | 1123 | CHN1 | chimerin (chimaerin) 1 | -0.5127 | 0.1297 | 0.5804 | 7.5288 | 6.9462 | 7.3089 | 7.4385 | 7.0597 | 6.8082 | 7.0024 | 7.1474 | 7.9822 | 7.7834 | 7.8245 | 7.1901 |
| 209464_at | 9212 | AURKB | aurora kinase B | -0.5165 | 0.1277 | 0.5784 | 8.9622 | 8.5388 | 8.6517 | 8.7116 | 8.6284 | 9.1315 | 10.9691 | 9.9373 | 9.0411 | 9.0922 | 9.5455 | 9.7997 |
| 205897_at | 4776 | NFATC4 | nuclear factor of activated T-cells, cytoplasmic, calcineurin-dependent 4 | -0.518 | 0.1337 | 0.5839 | 6.2411 | 5.8783 | 5.4536 | 5.223 | 5.4454 | 5.0884 | 5.7922 | 5.5138 | 7.1703 | 6.1187 | 6.3919 | 5.9873 |
| 208510_s_at | 5468 | PPARG | peroxisome proliferator-activated receptor gamma | -0.5182 | 0.1856 | 0.637 | 6.1289 | 6.2131 | 6.282 | 6.217 | 5.8825 | 6.1727 | 6.0914 | 6.3247 | 10.6909 | 6.1488 | 5.9559 | 10.7631 |
| 204517_at | 5480 | PPIC | peptidylprolyl isomerase C (cyclophilin C) | -0.5185 | 0.1098 | 0.5521 | 7.4449 | 7.2733 | 7.1634 | 7.4144 | 7.3841 | 7.375 | 7.044 | 7.4463 | 7.7509 | 7.9882 | 7.461 | 7.9539 |
| 201995_at | 2131 | EXT1 | exostosin 1 | -0.5235 | 0.06587 | 0.4807 | 6.9416 | 9.9133 | 8.9175 | 8.9883 | 9.0765 | 9.5105 | 11.0907 | 9.8849 | 10.9381 | 10.3414 | 10.7518 | 9.955 |
| 216347_s_at | 23368 | PPP1R13B | protein phosphatase 1, regulatory subunit 13B | -0.5241 | 0.1138 | 0.5563 | 6.7251 | 6.5474 | 6.3706 | 6.607 | 6.5301 | 7.1587 | 8.2172 | 7.1241 | 7.4511 | 7.1856 | 9.203 | 7.5244 |
| 203208_s_at | 9650 | MTFR1 | mitochondrial fission regulator 1 | -0.5246 | 0.1337 | 0.5839 | 9.7015 | 9.2936 | 8.9295 | 9.3198 | 9.4092 | 9.2274 | 9.3603 | 9.2815 | 10.1385 | 9.7142 | 9.9331 | 9.8376 |
| 203038_at | 5796 | PTPRK | protein tyrosine phosphatase, receptor type, K | -0.5264 | 0.1178 | 0.5646 | 8.7366 | 9.8973 | 8.8671 | 10.0612 | 9.1555 | 9.2316 | 9.4649 | 10.4772 | 10.7518 | 10.1385 | 10.6154 | 9.2079 |
| 201649_at | 9246 | UBE2L6 | ubiquitin-conjugating enzyme E2L 6 | -0.535 | 0.08383 | 0.5118 | 8.0104 | 7.7509 | 6.7922 | 7.3021 | 7.8544 | 8.1899 | 9.3187 | 8.3656 | 8.9869 | 8.4555 | 8.9295 | 8.8311 |
| 208950_s_at | 501 | ALDH7A1 | aldehyde dehydrogenase 7 family, member A1 | -0.5394 | 0.06986 | 0.488 | 6.8675 | 6.8703 | 7.1331 | 6.9296 | 6.3809 | 6.157 | 7.0111 | 7.6781 | 7.0775 | 7.7834 | 6.9073 | 6.93 |
| 213721_at | 6657 | SOX2 | SRY (sex determining region Y)-box 2 | -0.5454 | 0.09381 | 0.5294 | 7.0843 | 7.0954 | 6.7968 | 6.937 | 6.8479 | 6.8907 | 7.114 | 7.3377 | 7.7675 | 7.2784 | 7.5288 | 7.2667 |
| 204656_at | 6461 | SHB | Src homology 2 domain containing adaptor protein B | -0.5457 | 0.1218 | 0.5725 | 7.9244 | 7.836 | 6.8938 | 6.9645 | 7.2654 | 7.5013 | 7.7878 | 8.0828 | 9.5067 | 8.3107 | 9.1265 | 8.7346 |
| 218571_s_at | 29082 | CHMP4A | charged multivesicular body protein 4A | -0.5467 | 0.08184 | 0.5118 | 8.0321 | 8.1881 | 8.0157 | 7.9882 | 8.3098 | 7.844 | 8.5506 | 8.1881 | 8.094 | 8.3639 | 8.1878 | 8.2886 |
| 205633_s_at | 211 | ALAS1 | aminolevulinate, delta-, synthase 1 | -0.5532 | 0.09381 | 0.5294 | 10.3576 | 10.5248 | 10.2455 | 10.3576 | 10.4396 | 10.3781 | 12.4511 | 11.5626 | 11.2259 | 11.1378 | 10.7811 | 11.5374 |
| 219461_at | 56924 | PAK6 | p21 protein (Cdc42/Rac)-activated kinase 6 | -0.5534 | 0.07385 | 0.5012 | 3.7648 | 4.2178 | 3.9141 | 4.052 | 3.7081 | 4.2444 | 3.8814 | 3.641 | 3.9983 | 4.0871 | 4.3452 | 4.2682 |
| 204420_at | 8061 | FOSL1 | FOS-like antigen 1 | -0.5555 | 0.06387 | 0.4805 | 6.1124 | 6.0116 | 5.7922 | 5.8148 | 6.1187 | 5.9102 | 6.1336 | 6.1865 | 5.9211 | 5.7423 | 6.0275 | 6.2131 |
| 201980_s_at | 6251 | RSU1 | Ras suppressor protein 1 | -0.5616 | 0.07585 | 0.5012 | 6.4053 | 6.5091 | 6.295 | 6.282 | 6.3247 | 6.2758 | 8.1652 | 7.1445 | 6.6204 | 6.6399 | 6.4778 | 7.4688 |
| 221567_at | 8996 | NOL3 | nucleolar protein 3 (apoptosis repressor with CARD domain) | -0.5654 | 0.03792 | 0.4313 | 7.7811 | 7.8245 | 7.836 | 7.9661 | 8.0104 | 7.9752 | 8.9295 | 8.2704 | 8.5194 | 7.7986 | 8.272 | 8.7457 |
| 212717_at | 9842 | PLEKHM1 | pleckstrin homology domain containing, family M (with RUN domain) member 1 | -0.5673 | 0.0978 | 0.5314 | 9.2995 | 9.2127 | 8.0781 | 8.3388 | 8.6135 | 8.5665 | 10.1416 | 9.1601 | 10.2455 | 9.4254 | 9.6961 | 10.1697 |
| 202678_at | 2958 | GTF2A2 | general transcription factor IIA, 2, 12kDa | -0.5678 | 0.0499 | 0.4561 | 10.4318 | 10.3927 | 10.7112 | 10.5113 | 10.4318 | 10.4579 | 10.2552 | 10.4237 | 10.3106 | 10.6038 | 10.8218 | 10.7869 |
| 200825_s_at | 10525 | HYOU1 | hypoxia up-regulated 1 | -0.5696 | 0.05988 | 0.4761 | 8.0874 | 8.231 | 7.6629 | 7.8928 | 7.9728 | 7.8512 | 8.6284 | 7.7133 | 8.7527 | 8.2735 | 11.6611 | 8.296 |
| 221856_s_at | 55793 | FAM63A | family with sequence similarity 63, member A | -0.5702 | 0.02794 | 0.427 | 6.475 | 6.4567 | 6.2658 | 6.3872 | 6.4589 | 6.413 | 8.7346 | 6.4589 | 6.1124 | 6.4361 | 9.995 | 6.4929 |
| 209531_at | 2954 | GSTZ1 | glutathione transferase zeta 1 | -0.5707 | 0.1417 | 0.5898 | 7.5897 | 7.6742 | 7.9945 | 7.5312 | 7.7535 | 7.7811 | 7.3549 | 8.7012 | 8.9136 | 7.7535 | 8.5869 | 8.5194 |
| 219127_at | 79170 | PRR15L | proline rich 15-like | -0.5709 | 0.08982 | 0.5198 | 5.5182 | 5.6125 | 4.9824 | 5.1023 | 5.3176 | 5.5037 | 6.2658 | 5.6276 | 6.6276 | 5.9813 | 6.4441 | 5.9488 |
| 218856_at | 27242 | TNFRSF21 | tumor necrosis factor receptor superfamily, member 21 | -0.5727 | 0.03593 | 0.4285 | 4.6373 | 8.0517 | 7.5131 | 7.49 | 7.5506 | 7.9327 | 9.0351 | 8.0321 | 9.6092 | 8.6906 | 8.8209 | 9.8129 |
| 203927_at | 4794 | NFKBIE | nuclear factor of kappa light polypeptide gene enhancer in B-cells inhibitor, epsilon | -0.5774 | 0.04192 | 0.4361 | 5.8411 | 5.6226 | 5.5138 | 5.5378 | 5.6799 | 5.7604 | 8.1521 | 6.645 | 6.2268 | 6.0078 | 6.1336 | 6.673 |
| 202117_at | 392 | ARHGAP1 | Rho GTPase activating protein 1 | -0.5797 | 0.1277 | 0.5784 | 6.5746 | 6.8082 | 6.7922 | 6.3524 | 6.3105 | 6.3872 | 6.4307 | 6.6032 | 7.2559 | 7.044 | 7.044 | 6.5375 |
| 204949_at | 3385 | ICAM3 | intercellular adhesion molecule 3 | -0.5808 | 0.0479 | 0.442 | 4.9026 | 4.7924 | 4.9477 | 5.0739 | 4.8651 | 4.7409 | 4.9783 | 5.0985 | 4.9657 | 4.5716 | 5.0985 | 4.972 |
| 204191_at | 3454 | IFNAR1 | interferon (alpha, beta and omega) receptor 1 | -0.5815 | 0.05589 | 0.4718 | 7.3258 | 7.2247 | 6.7726 | 6.8907 | 6.8104 | 7.2142 | 7.8165 | 7.9244 | 8.5919 | 7.7133 | 7.3009 | 7.657 |
| 204369_at | 5290 | PIK3CA | phosphoinositide-3-kinase, catalytic, alpha polypeptide | -0.5831 | 0.07385 | 0.5012 | 8.8671 | 8.3368 | 7.4511 | 7.8634 | 7.9822 | 8.8377 | 9.7285 | 8.7226 | 11.1291 | 9.6213 | 9.9373 | 9.6038 |
| 217995_at | 58472 | SQRDL | sulfide quinone reductase-like (yeast) | -0.5834 | 0.03194 | 0.4285 | 5.6175 | 5.9693 | 5.6507 | 5.607 | 5.8375 | 5.4536 | 9.5067 | 10.4499 | 5.7928 | 5.7104 | 5.9915 | 5.5514 |
[truncated: 212,074 more chars]
